# The Novel Chinese Medicine JY5 Formula Alleviates Hepatic Fibrosis by Inhibiting the Notch Signaling Pathway

**DOI:** 10.3389/fphar.2021.671152

**Published:** 2021-09-22

**Authors:** Yadong Fu, Zhun Xiao, Xiaoting Tian, Wei Liu, Zhou Xu, Tao Yang, Yonghong Hu, Xiaoxi Zhou, Jing Fang, Siqi Gao, Dingqi Zhang, Yongping Mu, Hua Zhang, Yiyang Hu, Chenggang Huang, Jiamei Chen, Ping Liu

**Affiliations:** ^1^ Key Laboratory of Liver and Kidney Diseases (Ministry of Education), Institute of Liver Diseases, Shuguang Hospital Affiliated to Shanghai University of Traditional Chinese Medicine, Shanghai, China; ^2^ Institute of Interdisciplinary Integrative Medicine Research, Shanghai University of Traditional Chinese Medicine, Shanghai, China; ^3^ Shanghai Key Laboratory of Traditional Chinese Clinical Medicine, Shanghai, China; ^4^ Shanghai Institute of Materia Medica, Chinese Academy of Sciences, Shanghai, China; ^5^ Department of Cardiology, Cardiovascular Research Institute, Shuguang Hospital Affiliated to Shanghai University of Traditional Chinese Medicine, Shanghai, China

**Keywords:** traditional Chinese medicine, JY5 formula, fuzheng huayu, hepatic fibrosis, notch signaling pathway

## Abstract

Advanced liver fibrosis can lead to cirrhosis, resulting in an accelerated risk of hepatocellular carcinoma and liver failure. Fuzheng Huayu formula (FZHY) is a traditional Chinese medicine formula treated liver fibrosis in China approved by a Chinese State Food and Drug Administration (NO: Z20050546), composed of *Salvia Miltiorrhiza* bge., *Prunus davidiana* (Carr.) Franch., cultured *Cordyceps sinensis* (BerK.) Sacc. Mycelia, *Schisandra chinensis* (Turcz.) Baill., *Pinus massoniana* Lamb., and *Gynostemma pentaphyllum* (Thunb.) Makino. However, the main active substances and mechanism of FZHY are unclear. The aim of this study is to identify a novel anti-fibrotic compound, which consists of the main active ingredients of FZHY, and investigate its mechanism of pharmacological action. The main active ingredients of FZHY were investigated by quantitative analysis of FZHY extracts and FZHY-treated plasma and liver in rats. The anti-fibrotic composition of the main active ingredients was studied through uniform design *in vivo*, and its mechanism was evaluated in carbon tetrachloride (CCl_4_)- and bile duct ligation (BDL)-induced liver fibrosis models in rats and mice, and transforming growth factor beta 1-induced LX-2 cell activation model *in vitro*. A novel Chinese medicine, namely JY5 formula, consisting of salvianolic acid B, schisantherin A, and amygdalin, the main active ingredients of FZHY, significantly alleviated hepatic hydroxyproline content and collagen deposition in CCl_4_-and BDL-induced fibrotic liver in rats and mice. In addition, JY5 inhibited the activation of hepatic stellate cells (HSCs) by inactivating Notch signaling *in vitro* and *in vivo*. In this study, we found a novel JY5 formula, which exerted anti-hepatic fibrotic effects by inhibiting the Notch signaling pathway, consequently suppressing HSCs activation. These results provide an adequate scientific basis for clinical research and application of the JY5 formula, which may be a potential novel therapeutic candidate for liver fibrosis.

## Introduction

Liver fibrosis is a common pathological feature of chronic liver diseases including chronic viral hepatitis, metabolic-associated fatty liver disease, and cholestatic liver diseases. It is a consequence of an abnormal wound healing response, characterized by excessive deposition of extracellular matrix (ECM). If the injury persists, liver fibrosis can progress to cirrhosis and hepatocellular carcinoma, ultimately leading to liver failure (Friedman, 2015). The effective treatment for liver fibrosis is to address the root cause and prevent progression. Some clinical studies (Lee et al., 2015) have shown that liver fibrosis, even early cirrhosis, is reversible, providing reliable evidence for conducting clinical studies on anti-hepatic fibrosis drugs. With several types of animal models of liver fibrosis becoming increasingly mature, there has been a greater understanding of the pathogenesis of liver fibrosis in the last few decades (Cordero-Espinoza and Huch, 2018). A number of anti-hepatic fibrosis drug studies have been conducted in recent years, some of which have been researched in clinical trials (Lemoinne and Friedman, 2019). However, to date, there are no clinically approved by FDA or effective medical therapies aimed specifically towards hepatic fibrosis.

Traditional Chinese medicine (TCM) has marked clinical effects on the treatment of liver fibrosis. Among these, Fuzheng Huayu (FZHY) formula which composed of *Salvia miltiorrhiza* Bunge (Dansen), *Prunus davidiana* (CarriŠre) Franch (Taoren), cultured *Cordyceps sinensis* (BerK.) Sacc. Mycelia (Chongcao), *Schisandra chinensis* (Turcz.) Baill (Wuweizi), *Pinus massoniana* Lamb. (Songhuafen), and *Gynostemma pentaphyllum* (Thunb.) Makino (Jiaogulan), is a complex preparation to treat liver fibrosis in China approved by a Chinese State Food and Drug Administration (NO: Z20050546). The anti-fibrotic effect has been confirmed in a phase II clinical trial of hepatic fibrosis post-hepatitis C in the United States (Zhang and Schuppan, 2014). However, there are some challenges with TCM, such as its multi-component, multi-target, ill-defined active ingredients and mechanisms, which limit their clinical application. In recent years, our team has conducted a large amount of research to elucidate the mechanisms underlying the role of FZHY in the prevention and treatment of chronic liver diseases, including inhibiting the inflammatory response, protecting hepatocytes (to relieve hepatocyte damage and inhibit hepatocyte apoptosis), inhibiting hepatic stellate cell (HSC) activation, reducing collagen deposition, inhibiting Kupffer cell (KC) activation, inhibiting liver sinusoidal endothelial cell (LSEC) capillarization and angiogenesis, and promoting liver regeneration (Liu et al., 2009; Chen et al., 2019). We further studied the anti-fibrosis effects of different compounds of FZHY. Phenolic acids in *Salvia miltiorrhiza* play a prominent role in inhibiting the inflammatory response, protecting hepatocytes, and inhibiting HSC activation (Wang et al., 2012; Tao et al., 2013; Kan et al., 2014; Yan et al., 2017; Wang et al., 2019). Amygdalin, as one of the major active compounds of *Peach kernel*, has the main role in inhibiting the inflammatory response, reducing collagen deposition, and inhibiting HSC activation (Luo et al., 2016; Luo et al., 2018). The lignan compounds from *Schisandrae* play an important role in protecting hepatocytes and inhibiting HSC activation (Kang et al., 2019; He et al., 2020). These findings suggest that the related bioactive ingredients in FZHY may have anti-fibrotic effects.

Notch signaling is a highly conservative pathway during evolution. Notch receptors interact with ligands on the surface of adjacent cells, then cleave inside the cell membrane, translocate into the nucleus, and regulate the transcription of multiple target genes. Previous studies (Adams and Jafar-Nejad, 2019) have shown that as an important intercellular or intracellular signaling pathway, Notch plays an important role in liver development and pathophysiology. Notch has a great impact on the occurrence and development of hepatic fibrosis and can interact with transforming growth factor beta (TGF-β), Hedgehog, and Hippo signaling pathways to mediate cell–cell interactions. Activation of HSCs is a critical cellular event in liver fibrosis. HSCs transdifferentiate into myofibroblasts, accompanied by activation of Notch signaling pathway (Zhang et al., 2015). After Notch activity levels are suppressed, this process can be reversed. In addition, with the progression of liver fibrosis induced by carbon tetrachloride (CCl_4_) and bile duct ligation (BDL), the Notch signaling pathway is significantly activated. Efficient inhibition of the Notch pathway can significantly mitigate liver fibrosis and reduce hepatocyte apoptosis (Chen et al., 2012). Thus, targeting the Notch signaling pathway can regulate the activation of HSCs, thereby suppressing the occurrence and progression of hepatic fibrosis.

In this study, we found that salvianolic acid B, schisantherin A, and amygdalin were the main active ingredients of FZHY formula by quantitative analysis of FZHY extracts and FZHY-treated plasma and liver in rats. A novel TCM formula, namely JY5, was obtained through uniform design. The anti-hepatic fibrosis efficacy of JY5 was comparable to that of FZHY in CCl_4_-induced hepatic fibrosis in rats (Supplementary Figures S3 and S4). Further studies demonstrated that JY5 alleviated liver fibrosis by inhibiting the activation of HSCs via inhibition of the Notch signaling pathway.

## Materials and Methods

### Animals

Adult Wistar or Sprague-Dawley male rats (160–180 g, specific pathogen-free [SPF] grade) were purchased from Shanghai Xipuer-Bikai Experimental Animal Co., Ltd (Shanghai, China) and fed in the Laboratory Animal Center at School of Pharmacy, Fudan University (Shanghai, China). Adult male C57/BL6 mice (aged 6–8 weeks, 18–20 g, SPF grade) were purchased from Shanghai Southern Model Biotechnology Co., Ltd (Shanghai, China) and maintained in the Shanghai Research Center of the Southern Model Organisms (Shanghai, China). Rats and mice were housed under constant conditions (ambient temperature 25 ± 2°C, relative humidity 40–60%, and 12/12 h light-dark cycle) with free access to standard diet and water. All rat experiments were reviewed and approved by the Experimental Animal Ethics Committee of School of Pharmacy, Fudan University (Approval No. 2018–07-SZYD-LP-01). All mice experiments were approved by the Institutional Animal Care and Use Committee (IACUC) at Shanghai Research Center of the Southern Model Organisms (Approval No. 2019–0031).

### Drugs

Reference standards: salvianolic acid B, salvianic acid, salvianic acid A, rosmarinic acid, gypenoside XLIX, ginsenoside Rb3, amygdalin, schisantherin A, schisandrol A, schisandrol B, deoxyschizandrin, schisandrin B, tanshinone, cryptotanshinone, adenosine, and cordycepin were purchased from Shanghai Standard Technology Co., Ltd (Shanghai, China). The purity of all standards was more than 98%. FZHY decoction: mixture of *Salvia miltiorrhiza* Bunge at 533 g, *Prunus davidiana* (CarriŠre) Franch. at 133 g, and *Gynostemma pentaphyllum* (Thunb.) Makino at 400 g, was heated to boiling with water for 2 h for the first time and for 1.5 h for the second time. The combined decoction was filtered, and concentrated to a relative density at 1.20 g/ml (50–55°C). After cooling down, the decoction was precipitated by adding 70% alcohol, and then filtrate 1 was generated after filtration and concentration. A combination of cultured *Cordyceps sinensis* (BerK.) Sacc. Mycelia at 267 g and *Schisandra chinensis* (Turcz.) Baill. at 133 g was heated with 70% alcohol for 2 h for the first time and 1.5 h for the second time, and the combined decoction was filtered and concentrated as filtrate 2. *Pinus massoniana* Lamb. at 133 g was infiltrated with 50% alcohol for 4 h for the first time and 2 h for the second time, and the combined decoction was filtered and concentrated as filtrate 3. Filtrates 1–3 were combined and concentrated to 800 ml at 2 g raw drug/mL. The voucher specimens, *Salvia miltiorrhiza* Bunge (No. 1600001), *Prunus davidiana* (CarriŠre) Franch (No. 1600002), *Gynostemma pentaphyllum* (Thunb.) Makino (No. 1600003), cultured *Cordyceps sinensis* (BerK.) Sacc. Mycelia (No. 1600004), *Schisandra chinensis* (Turcz.) Baill (No. 1600005), and *Pinus massoniana* Lamb. (No. 1600006) were deposited in the Shanghai Institute of Materia Medica.

### Pharmacokinetic Study of Fuzheng Huayu Decoction in Rats

This study was conducted according to the guidelines of the IACUC of the Shanghai Institute of Materia Medica, Chinese Academy of Sciences (Shanghai, China). The experimental protocol is shown in Supplementary Material: Materials and Methods 1.1.

### Instrumentation

There were 16 compounds determined in various biosamples by the UltiMate 3000 ultra-high-performance liquid chromatograph linked to the active quadrupole electrostatic field orbital trap high-resolution mass spectrometer, connected to an electrospray ionization source (Thermo Fisher Scientific, Waltham, MA, United States). The operating parameters were set as shown in Supplementary Material: Materials and Methods 1.2.

### Experimental Liver Fibrosis Models

CCl_4_-induced liver fibrosis rat model, BDL-induced liver fibrosis rat model, and CCl_4_-induced liver fibrosis mouse model were used in this study. The experimental protocols are shown in Supplementary Material: Materials and Methods 1.3.

### Cell Culture

The immortalized human hepatic stellate cell line (LX-2) was provided by the Institute of Liver diseases, Shanghai University of Traditional Chinese Medicine (Shanghai, China). LX-2 cells (1.25 × 10^5^) were seeded in 6 cm dishes or 6-well plates and maintained in Dulbecco’s Modified Eagle Medium containing 1% penicillin/streptomycin and 10% fetal bovine serum at 37°C and 5% CO_2_. After post-inoculation for 24 h, all LX-2 cells except the control group were treated with TGF-β1 (5 ng/ml), and simultaneously treated with different concentrations of JY5 as follows: low-dose group (6.587 μg/ml) (salvianolic acid B 8 μM, amygdalin 0.25 μM, schisantherin A 1 μM), medium-dose group (13.174 μg/ml) (salvianolic acid B 16 μM, amygdalin 0.5 μM, schisantherin A 2 μM), and high-dose group (26.348 μg/ml) (salvianolic acid B 32 μM, amygdalin 1 μM, schisantherin A 4 μM). SB431542 (10 μM), a TGF-β receptor inhibitor, was used as a positive control. After incubation for 24 h, LX-2 cells were lysed and collected for Western blotting and quantitative PCR (qPCR) analysis.

### Serum Biochemistry Analysis

Serum alanine aminotransferase (ALT), aspartate aminotransferase (AST), alkaline phosphatase (ALP), total bilirubin (TBil), direct bilirubin (DBil), and total bile acid (TBA) levels were measured using the TBA-40FR automatic biochemistry analyzer (Toshiba Medical, Tokyo, Japan) at the Science and Technology Experiment Center, Shanghai University of TCM.

### Histopathological and Immunohistochemical Analysis

Liver injury and fibrosis were assessed with hematoxylin and eosin (H&E) and Sirius Red (SR) staining using 4 μm thick paraffin-embedded liver sections. Immunohistochemistry (IHC) staining of collagen type I (Col-I), Col-IV, *a*-smooth muscle actin (α-SMA), and desmin was performed. The detailed protocols are shown in Supplementary Material: Materials and Methods 1.4.

### Hepatic Hydroxyproline Content Assay

According to the kit instructions, hydroxyproline (Hyp) content in liver tissue was detected using the Hydroxyproline Testing Kit-Alkaline Hydrolysis Method (Cat No. A030-2; Nanjing Jiancheng Bioengineering Institute, Nanjing, China).

### Western Blot Analysis

Total protein in liver tissues or cells was extracted using RIPA lysis buffer containing proteinase and phosphatase inhibitor (Cat No. P0013B; Biyuntian Biotechnology Co., Ltd., Shanghai, China). Total protein concentration was determined using a BCA protein assay kit (Lot TD265229; Thermo Fisher Scientific). Proteins (30–50 μg) were denatured at 100°C for 5  min, and then separated by 8% or 10% sodium dodecyl sulfate–polyacrylamide gel electrophoresis. The proteins were electrotransferred onto polyvinylidene fluoride (PVDF) membranes. The membranes were blocked in 5% BSA at room temperature for 60 min, and then incubated with primary antibody (Supplementary Table S1) overnight at 4°C. The following day, the membranes were incubated in the dark for 1 h at room temperature with fluorescence-labeled secondary antibody (Supplementary Table S1). The PVDF membranes were scanned using the Odyssey 2.1 software of Odyssey infrared scanner (LI-COR Biosciences, Lincoln, NE, United States). After scanning, target protein bands were cut out according to the molecular weight of target protein without any edit. The greyscale values relative to GAPDH of the target proteins were analyzed using ImageJ software.

### Quantitative PCR Analysis

Total RNA in liver tissues or cells was extracted and reverse transcribed using a nucleic acid purification kit (Code: NPK-201F, Lot. 742,100; Toyobo Co., Ltd., Osaka, Japan) and the ReverTra Ace qPCR RT Kit (Code: FSQ-301, Lot. 616,800; Toyobo Co., Ltd., Osaka, Japan). The qPCR primer sequence information is listed in Supplementary Table S2. The PCR cycling program was 95°C for 60 s, 40 cycles of 95°C for 15 s, and 60°C for 60 s, followed by melting curve analysis. The GAPDH gene was used as the internal reference for normalization of the target genes. The relative mRNA expression of each group was calculated using the 2^−ΔΔCt^ method.

### Dual-Luciferase Reporter Assay

The transcriptional activity of Notch was measured using RBP-кβ luciferase reporter plasmid constructed by Shanghai Jikai Gene Chemical Technology Co. Ltd. following the supplier’s instructions. The RBP-кB-Luc vector were engineered in GV238 backbone vector. The target gene sequence of RBP-кB (NM_005,349-promoter-1) was amplified by PCR using the primers as follows: forward: 5′-CTA​GCC​TAG​GCG​ACA​GAG​CAA​G-3’; reverse: 5′-CTT​TAT​GTT​TTT​GGC​GTC​TTC​CA-3’. The amplicons were inserted into the cloning sites of KpnI and XhoI located upstream of the firefly luciferase gene. and then a dual-luciferase reporter assay was performed using Dual-Lumi™ luciferase reporter gene assay kit (RG088S, Beyotime Biotechnology, China). The transiently co-transfected LX-2 cells with the corresponding transfection mix containing 200 ng RBP-кB-Luc plasmid (firefly) and 20 ng pRL-TK control vector (renilla) using Lip8000TM (C0533, Beyotime Biotechnology, China) were treated with TGF-β1 (5 ng/ml), and simultaneously treated with salvianolic acid B (32 μM), amygdalin (1 μM), schisantherin A (4 μM) or JY5 (26.348 μg/ml) for 24 h, respectively. RBP-кB luciferase activity was detectedby Dual-Lumi™ luciferase reporter gene assay kit following the manufacturer’s instructions. With renilla luciferase as the internal control in each transfection, the relative luciferase activity was calculated as the ratio of firefly-to-renilla luciferaseactivity.

### Statistical Analysis

All data were analyzed using the SPSS 21.0 software package. All data are expressed as the mean ± standard deviation (SD). Comparisons between multiple groups were analyzed by the one-way analysis of variance, followed by the least significant difference test. *p* < 0.05 was considered statistically significant. In addition, the pharmacokinetic (PK) parameters were calculated by noncompartmental analysis in WinNonlin software with a sparse sampling algorithm (Pharsight 6.2, Cary, NC, United States).

## Result

### Quantitative Analysis of Fuzheng Huayu Decoction and Fuzheng Huayu Biological Samples

**FIGURE 1:**
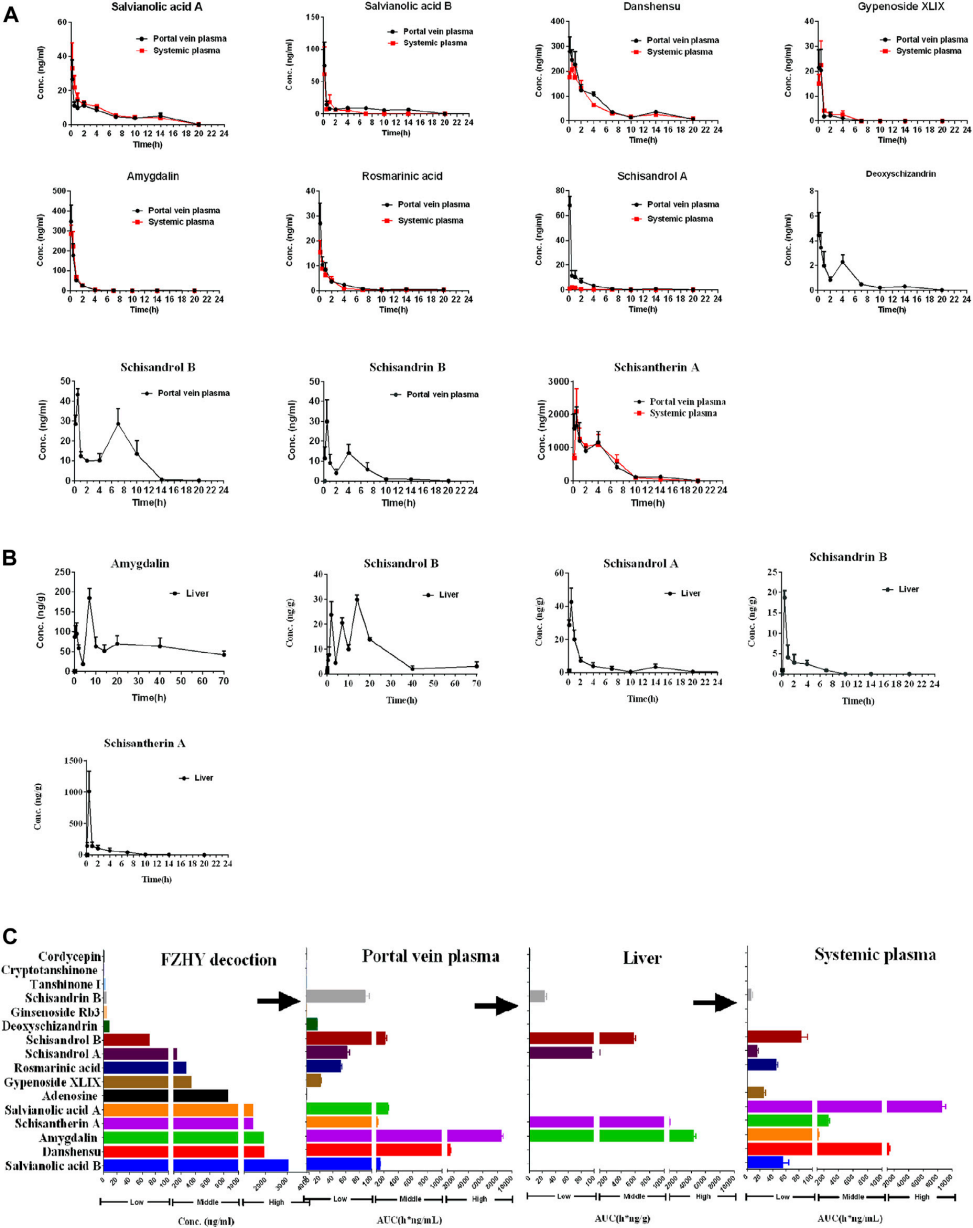
Quantitative analysis of FZHY decoction and FZHY biological samples. Concentration–time curves of compounds in the plasma **(A)** and liver **(B)** after oral administration of FZHY decoction (20 g/kg) in rats (*n* = 5). **(C)** Summary of the contents and AUC of compounds in the FZHY decoction or FZHY biological samples.

The chemical structure and concentration of 16 compounds in the FZHY decoction (2 g/ml) are shown in Supplementary Figures S1, S2 and Supplementary Table S3 respectively. The concentration–time curves of compounds in the plasma and liver after oral administration of FZHY decoction (20 g/kg) in rats are shown in Figures 1A,B, and the corresponding PK parameters are shown in Table 1. The summary of the contents and area under the curve (AUC) of compounds in the FZHY decoction or FZHY biological samples are shown in Figure 1C. There were 16 compounds determined in the FZHY decoction, including six derived from *Salvia miltiorrhiza*, five derived from *Schisandra chinensis*, two derived from *Gynostemma pentaphylla*, two from *Cordyceps mycelium*, and one compound from *Gynostemma pentaphylla*. Among the compounds, salvianolic acid B had the highest content in the FZHY decoction, followed by danshensu, amygdalin, schisantherin A, and salvianolic acid A (concentration≥1000 μg/ml). Adenosine, gypenoside XLIX, rosmarinic acid, and schisandrol A were in the middle content group (100 μg/ml < concentration<1000 μg/ml), and the concentrations of the remaining compounds, including schisandrol B, deoxyschizandrin, ginsenoside Rb3, schisandrin B, tanshinone I, cryptotanshinone, and cordycepin, were very low (concentration≤100 μg/ml). The portal vein blood is the first site after gut absorption but before hepatic disposition, which is responsible for transferring the substances to the liver post-dose. Following oral administration of FZHY in rats, there were 11 compounds accurately detected in the portal vein plasma, of which tanshinone I, cryptotanshinone, cordycepin, ginsenoside Rb3, and adenosine were undetected, probably due to the low content in the formula or poor physicochemical property. T_max_s of all compounds were within 0.5 h in portal vein plasma, indicating fast absorption. Schisantherin A exhibited maximum exposure in the portal vein plasma, followed by danshensu (AUC≥1000 h*ng/mL). Four compounds, including amygdalin, schisandrol B, salvianolic acid B, and salvianolic acid A, belonged to the middle exposure group (100 h*ng/mL < concentration <1000 h*ng/mL). The remaining five compounds were in the low exposure group (AUC≤100 h*ng/mL). After hepatic disposition, the compounds were transported to the systemic plasma, which is responsible for delivering substances to the other organs, except the liver. Compared to those in the portal vein plasma, there were eight compounds determined in the systemic plasma, of which schisandrol A, schisandrin B, and deoxyschizandrin were undetected. Similar to those in the portal vein plasma, the absorption of those compounds was quick; schisantherin A and danshensu were in the high exposure group (AUC≥1000 h*ng/mL), and schisantherin A had the highest exposure in the systemic plasma. The middle exposure group (100 h*ng/mL < AUC <1000 h*ng/mL) included amygdalin and salvianolic acid A; the remaining compounds belonged to the low exposure group (AUC≤100 h*ng/mL). Following oral administration of FZHY decoction, only five compounds were detected in the liver. T_max_s of schisandrol A, schisandrin B, and schisantherin A was 0.5 h, similar to that in the plasma. By contrast, T_max_s of schisandrol B and amygdalin was 14 and 7 h, respectively, consistent with their multi-peak phenomenon in the concentration-time curves. By contrast to levels in the plasma, amygdalin had the highest hepatic exposure, followed by schisantherin A, whose exposure was >1000 h*ng/g. Schisantherin A was in the middle exposure group (100 h*ng/g < AUC <1000 h*ng/g), and the hepatic exposure of schisandrol A and schisandrin B was very low (AUC≤100 h*ng/g). Finally, salvianolic acid B, schisantherin A, and amygdalin, which had the highest content in the FZHY decoction, plasma, and liver, respectively, were selected to evaluate the anti-hepatic fibrosis effects *in vivo*.

**TABLE 1:** The PK parameters of 11 compounds in the portal vein plasma, systemic plasma and liver, following oral administration of FZHY decoction in rats.

Compounds	Portal vein plasma			Systemic plasma		
	T_max_(h)	C_max_ (ng/ml)	AUC(h*ng/ml)	T_max_(h)	C_max_ (ng/ml)	AUC(h*ng/ml)
Salvianolic acid A	0.167	26.7 ± 11.2	108.7 ± 9.1	0.167	33.1 ± 14.9	122.3 ± 5.7
Salvianolic acid B	0.167	74.8 ± 36.4	144.4 ± 7.3	0.167	61.5 ± 42.1	55.9 ± 18.6
Danshensu	0.167	279.8 ± 58.8	1515.9 ± 64.6	0.5	206.6 ± 41.6	1362 ± 95
Gypenoside XLIX	0.5	21.5 ± 7.3	21.3 ± 3.5	0.5	22.4 ± 9.8	26.4 ± 3.8
Amygdain	0.167	347.7 ± 82.5	254.7 ± 23.2	0.167	286.2 ± 44.2	262.8 ± 23.9
Rosmarinic acid	0.167	27.1 ± 8.1	51.9 ± 4	0.167	15.4 ± 4.9	45.5 ± 4.7
Schisandrol A	0.167	68.2 ± 7.2	62 ± 8.6			
Schisandrol B	0.5	43.4 ± 3	215.1 ± 49.3	0.5	35.9 ± 0.6	84.3 ± 19.8
Deoxyschizandrin	0.167	4.4 ± 1.8	15.6 ± 1.1			
Schisandrin B	0.5	29.9 ± 11	89.4 ± 15.7			
Schisantherin A	0.5	1657.7 ± 584.3	8542.1 ± 566.2	0.5	2094.4 ± 681	8672.6 ± 1093.6

Compouds	Liver		
	T_max_(h)	C_max_ (ng/g)	AUC(h*ng/g)
Amygdain	7	185 ± 24.1	4423.2 ± 599.8			
Schisandrol A	0.5	42.5 ± 8.4	96.4 ± 13.7			
Schisandrol B	14	29.9 ± 1.8	578.7 ± 50.3			
Schisandrin B	0.5	18.7 ± 1.7	24.1 ± 4.6			
Schisantherin A	0.5	1011.1 ± 322.6	1070.3 ± 103.4			

### JY5 Significantly Alleviates Hepatic Injury and Collagen Deposition in CCl_4_-Induced Rat and Mouse Liver Fibrosis

**FIGURE 2:**
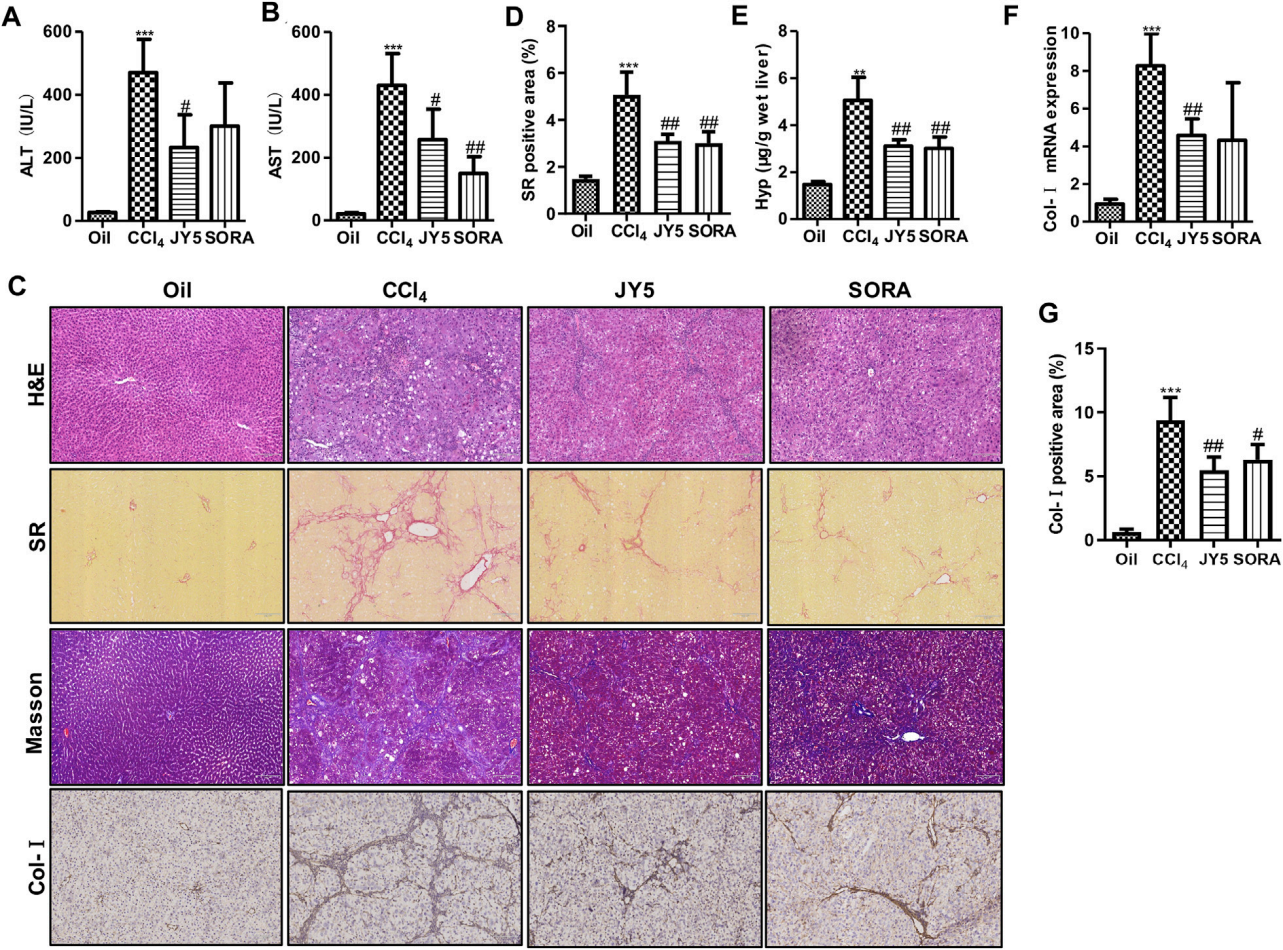
JY5 significantly alleviates hepatic inflammatory injury and collagen deposition in CCl_4_-induced rat liver fibrosis**.** Serum levels of ALT **(A)** and AST **(B)** were measured in each group. **(C)** H&E staining (100×), SR staining (100×), Masson staining (100×) and IHC staining of Col-I (100×), and semi-quantitative analysis **(D)** of collagen disposition (%) in SR-stained liver sections **(E)** Hyp content in wet liver tissue was detected by alkaline hydrolysis. **(F)** The mRNA levels of *Col-I* in liver tissue were analyzed by qPCR. **(G)** quantitative analysis of immunohistochemical staining for Col-I. **p < 0.01, ***p < 0.001 vs the control group, ^#^ *p* < 0.05, ^##^ *p* < 0.01 vs the CCl_4_ group. Oil: control group.

**FIGURE 3:**
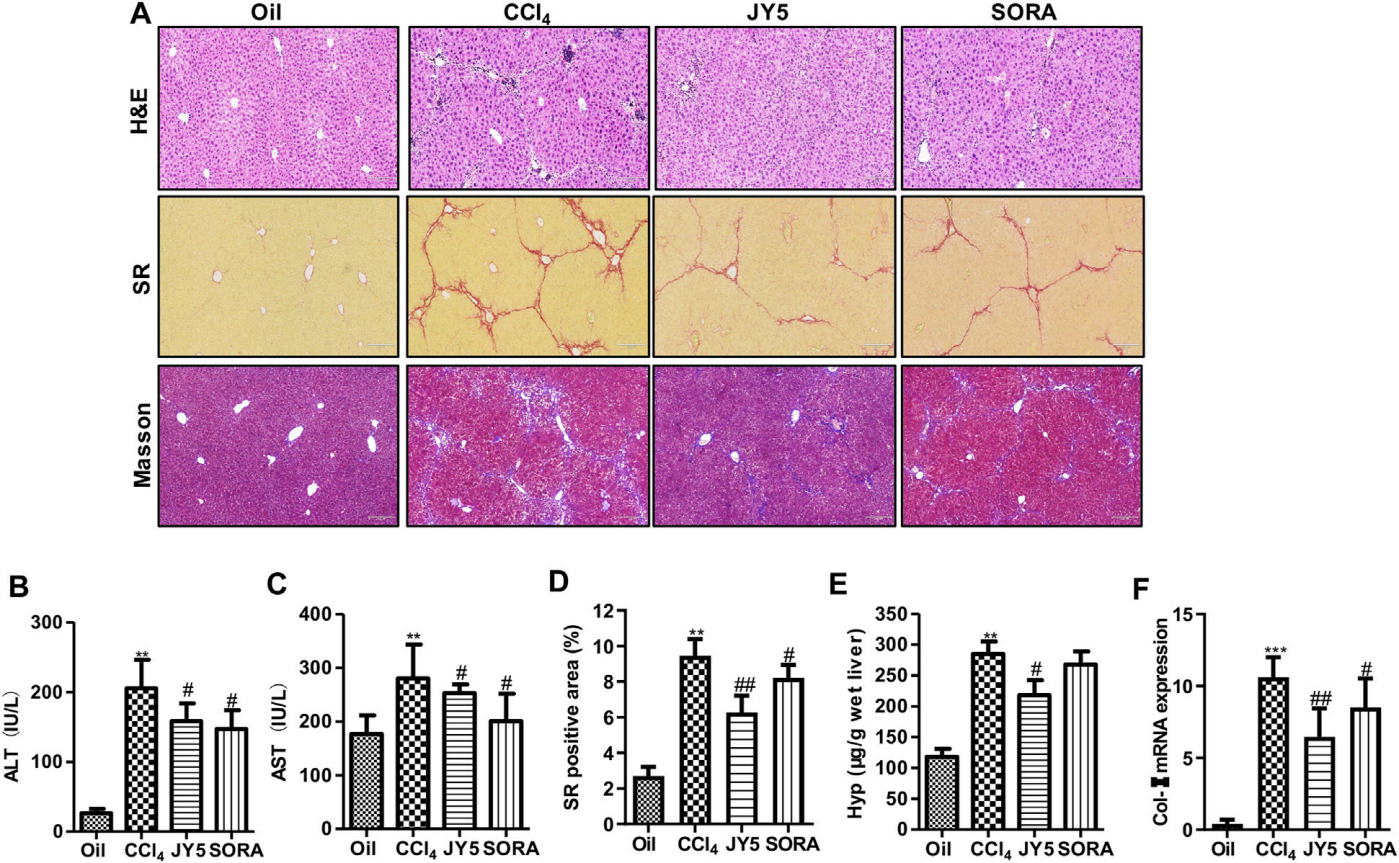
JY5 significantly alleviates hepatic inflammatory injury and collagen deposition in CCl_4_-induced mouse liver fibrosis. **(A)** H&E staining (100×), SR staining (100×) and Masson staining (100×) in liver tissue. Serum levels of ALT **(B)** and AST **(C)** were measured in each group. **(D)** Semi-quantitative analysis of collagen disposition (%) in SR-stained liver sections. **(E)** Hyp content in wet liver tissue was detected by alkaline hydrolysis. **(F)** The mRNA expression of *Col-I* in mice liver tissue was analyzed by qPCR. **p < 0.01, ***p < 0.001 vs the control group, ^#^ *p* < 0.05, ^##^ *p* < 0.01 vs the CCl_4_ group. Oil: control group.

Compared with the control group (Oil), the levels of serum ALT and AST were significantly increased in the CCl_4_ group. After treatment with JY5 (salvianolic acid: B 16 mg/kg, amygdalin: 0.5 mg/kg, schisantherin A: 2 mg/kg) or FZHY (2 g/kg), the levels of ALT and AST were significantly decreased (Figures 2A,B and Supplementary Figures S3D,E). The serum AST level was decreased in the sorafenib group (SORA, 5 mg/kg) compared with the CCl_4_ group (Figure 2B). H&E staining showed that the hepatic lobular structure was severely collapsed with formation of more complete pseudo-lobules in the CCl_4_ group. As the fibrous tissue became denser, the hepatocytes were disordered and ballooning degeneration occurred. There was a large number of inflammatory cells infiltration surrounding the hepatic sinusoid, central vein, and portal tract. The above lesions were obviously attenuated with less pseudo-lobules and inflammatory cells infiltration, after treatment with JY5 or FZHY or SORA (Supplementary Figure S3A and Figure 2C, upper panel). SR and Masson staining showed that compared to the control group, collagen deposition was obviously increased in the CCl_4_ group. The fibrotic septum became significantly widened and distributed from the portal tract to the periphery in a reticular manner, forming pseudo-lobules with varying sizes. By contrast, collagen deposition was obviously decreased, the fibrotic septum became narrower, and pseudo-lobules structures were observed less in the JY5-, FZHY-, or SORA-treated groups (Supplementary Figure S3A, lower panel and Figure 2C, middle panel). Both the hepatic Hyp content and collagen deposition were significantly increased in the CCl_4_ group compared to the control group. The above indicators were significantly reduced after intervention with JY5, FZHY, or SORA (Supplementary Figures S3B,C and Figures 2D,E). These results demonstrated that JY5 formula had significant anti-hepatic fibrosis effects. IHC staining showed that compared with the control group, Col-I expression was visible in the fibrotic septum in the CCl_4_ group. By contrast, JY5 and SORA significantly reduced Col-I expression in the liver tissue (Figure 2C, lower panel and Figure 2G). In addition, qPCR results showed that *Col-I* mRNA expression was significantly more elevated in the CCl_4_ group than in the control group. However, compared to the CCl_4_ group, *Col-I* mRNA expression was significantly reduced in the JY5-treated group (Figure 2F). Consistent with the CCl_4_-induced rat liver fibrosis model, JY5 (salvianolic acid B, 22.4 mg/kg amygdalin, 0.7 mg/kg schisantherin A, 2.8 mg/kg) significantly alleviated hepatic injury and collagen deposition in CCl_4_-induced liver fibrosis in mice (Figure 3).

### JY5 Significantly Alleviates Hepatic Injury and Collagen Deposition in Bile Duct Ligation-Induced Rat Liver Fibrosis

**FIGURE 4:**
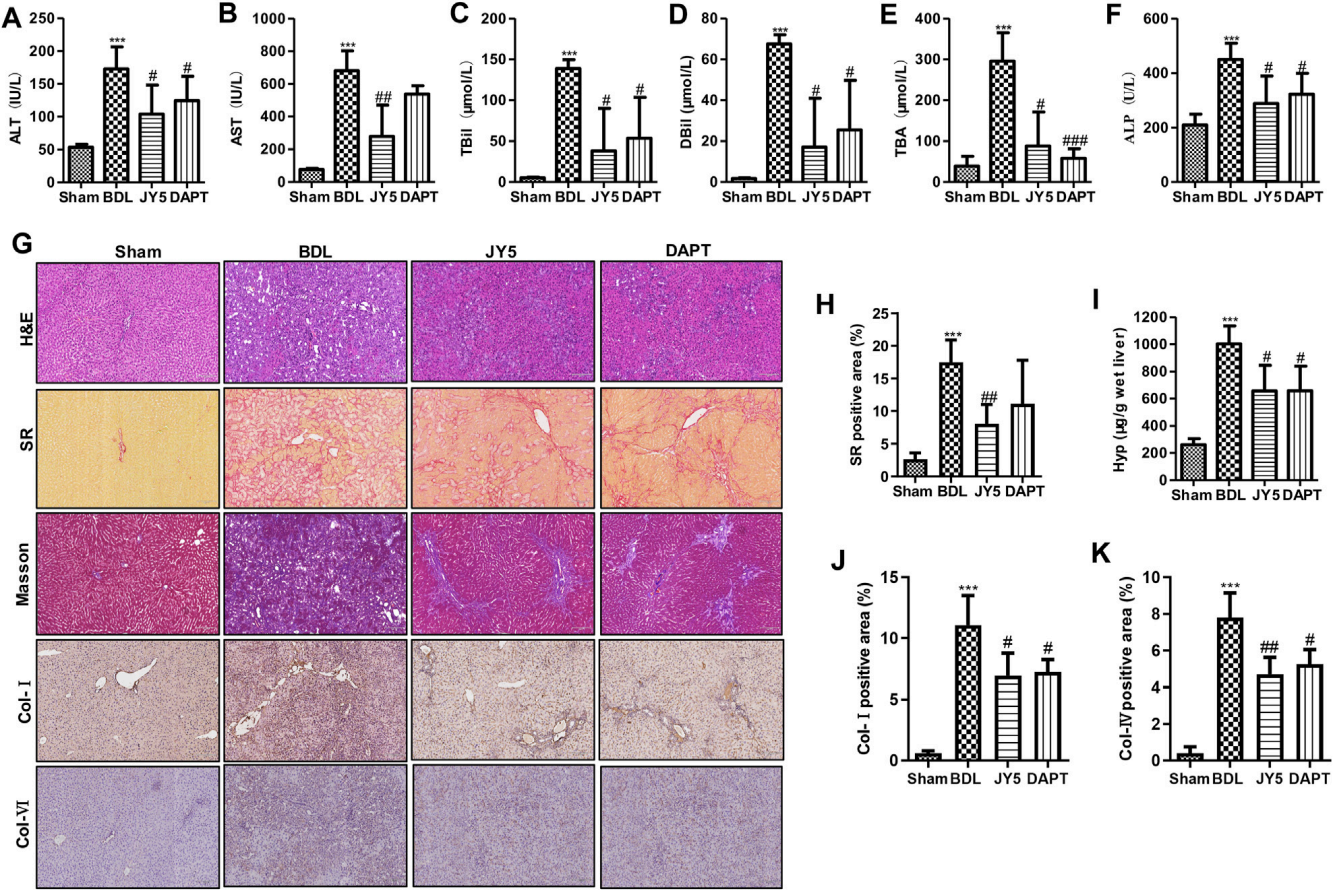
JY5 significantly alleviates hepatic inflammatory injury and collagen deposition in BDL-induced rat liver fibrosis. The levels of serum ALT **(A)**, AST **(B)**, TBil **(C)**, DBil **(D**), TBA **(E)**, and ALP **(F)** were measured in each group. **(G)** H&E staining (100×), SR staining (100×), Masson staining (100×) and IHC for Col-I and Col-IV (100×) staining, and semi-quantitative analysis **(H)** of collagen disposition (%) in SR-stained liver sections. **(I)** Hyp content in wet liver tissue was detected by alkaline hydrolysis. Quantitative analysis of immunohistochemical staining for Col-I **(J)** and Col-IV **(K)**. ***p < 0.001 vs the sham group, ^#^ *p* < 0.05, ^##^ *p* < 0.01 vs the BDL group.

Compared with the sham group, the levels of ALT, AST, TBil, DBil, TBA, and ALP were significantly increased in the BDL group. After JY5 (salvianolic acid B, 16 mg/kg amygdalin, 0.5 mg/kg schisantherin A, 2 mg/kg) or DAPT (30 mg/kg) treatment, the levels of ALT, AST, TBil, DBil, TBA, and ALP were significantly decreased (Figures 4A–F). Consistent with CCl_4_-induced liver fibrosis, JY5 reduced hepatic Hyp content and collagen deposition, and downregulated the expression of Col-I and Col-IV in BDL-induced rat liver fibrosis (Figures 4G–K). These results suggest that JY5 significantly alleviates hepatic injury and collagen deposition in BDL-induced liver fibrosis.

### JY5 Significantly Represses the Activation of Hepatic Stellate Cells *In Vivo*

**FIGURE 5:**
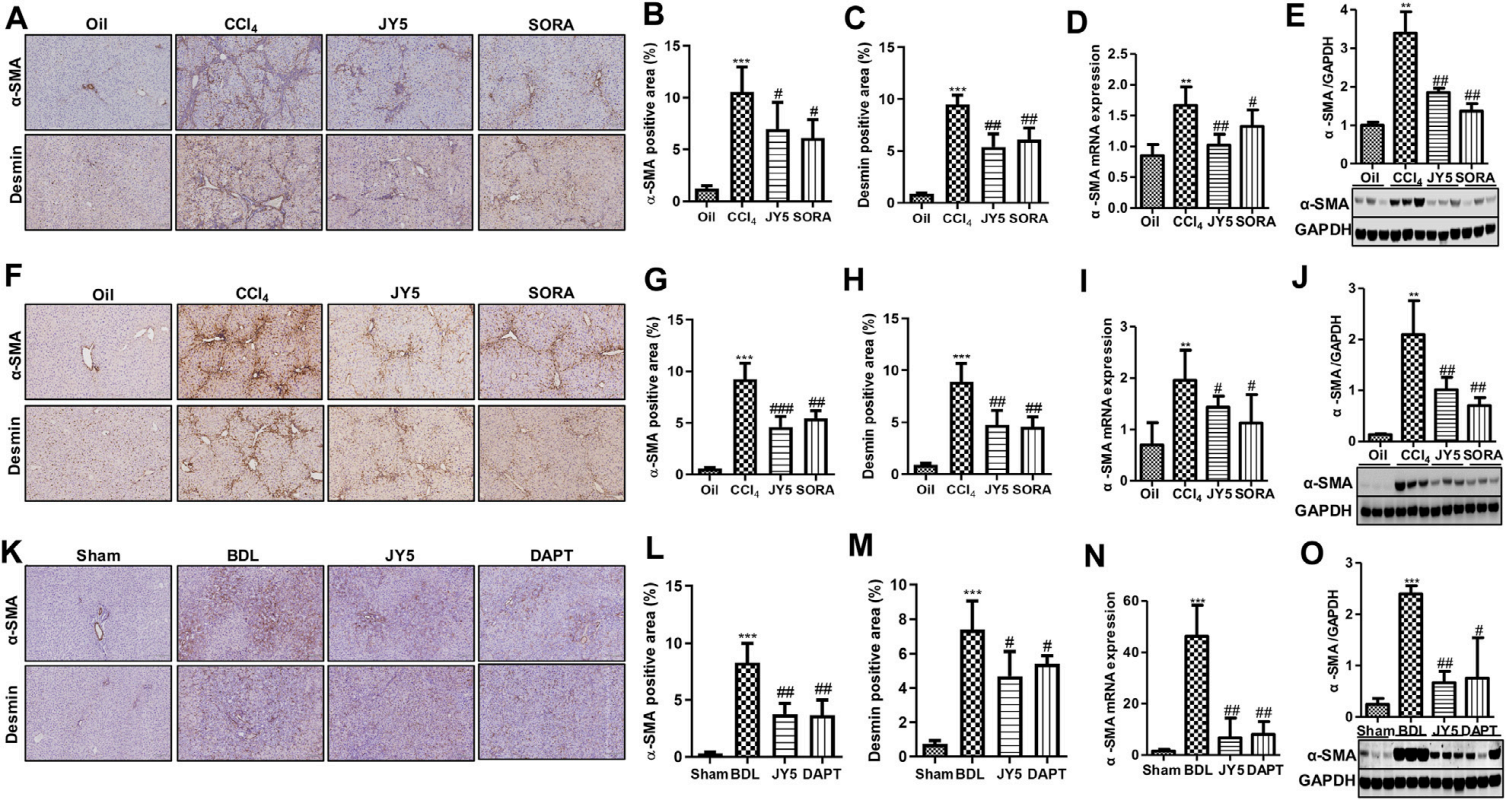
JY5 inhibits the activation of HSCs ***in vivo***. **(A)** In the CCl_4_-induced rat liver fibrosis experiment, representative images of IHC (100×) staining for *a*-SMA and desmin. Quantitative analysis of immunohistochemical staining for *a*-SMA **(B)** and desmin. **(C)** The protein and mRNA levels of *a*-SMA were, respectively, analyzed by qPCR **(D)** and Western blotting **(E)**. **(F)** In the CCl_4_-induced liver fibrosis mice experiment, representative images of IHC (100×) staining for *a*-SMA and desmin in liver tissue from mice treated with the various treatments. Quantitative analysis of immunohistochemical staining for *a*-SMA **(G)** and desmin. **(H)** The protein and mRNA levels of *a*-SMA in mice liver tissue were, respectively, analyzed by qPCR **(I)** and Western blotting **(J)**. **(K)** In the BDL-induced rat liver fibrosis experiment, representative images of IHC (100×) staining for *a*-SMA and desmin in liver tissue. Quantitative analysis of immunohistochemical staining for *a*-SMA **(L)**and desmin **(M)**. The protein and mRNA expression of *a*-SMA was, respectively, analyzed by qPCR **(N)** and Western blotting **(O)**. **p < 0.01, ***p < 0.001 vs the control group or sham group, ^#^ *p* < 0.05, ^##^ *p* < 0.01 vs the CCl_4_ group or BDL group. Oil: control group.

In both the CCl_4_-induced rat and mouse liver fibrosis experiments, IHC staining showed that high *a*-SMA and desmin was expressed in the fibrotic septum in the CCl_4_ group. By contrast, both *a*-SMA^(+)^ cells and desmin^(+)^ cells were decreased in the JY5- and SORA-treated group (Figures 5A–C,F–H). Western blot analysis and qPCR showed that *a*-SMA expression was significantly elevated compared to the control group. However, compared to the CCl_4_ group, both *a*-SMA mRNA and protein expression was significantly reduced in the JY5- and SORA-treated groups (Figures 5D,E,I,J). Similarly, in the BDL-induced liver fibrosis experiment, the treatment effect of JY5 was consistent with the results in the CCl_4_-induced liver fibrosis experiments (Figures 5K–O). These results demonstrate that JY5 significantly represses the activation of HSCs in CCl_4_ -and BDL-induced liver fibrosis.

**FIGURE 6:**
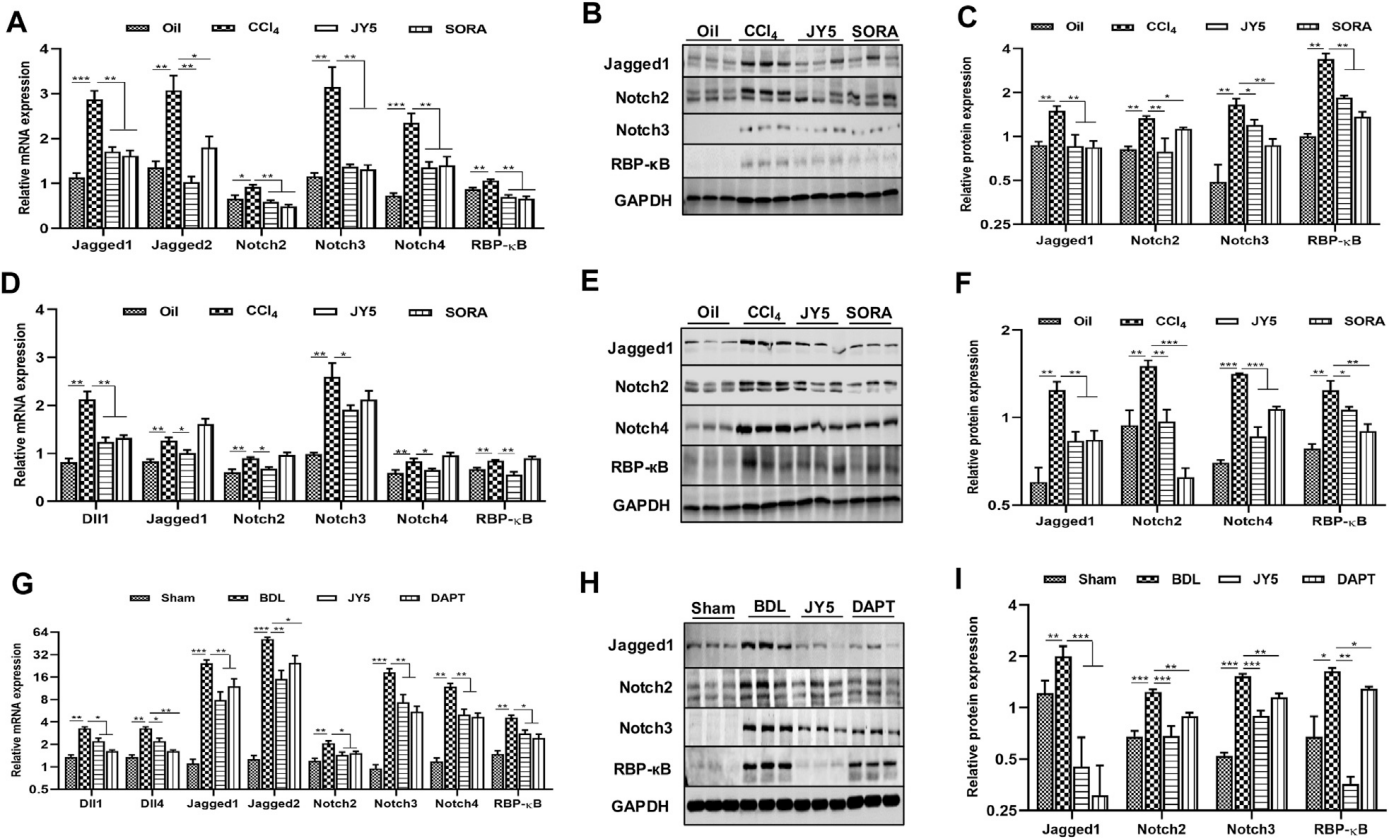
JY5 may ameliorate liver fibrosis by inhibiting the Notch signaling pathway ***in vivo***. **(A)** In the CCl_4_-induced rat liver fibrosis experiment, the mRNA levels of *Jagged1, Jagged2, Notch2, Notch3, Notch4* and *RBP-κB* were measured by qPCR. Western blotting **(B)** and quantitative analysis **(C)** of Jagged1, Notch2, Notch3 and RBP-κB protein. **(D)** In the CCl_4_-induced liver fibrosis mice experiment, the mRNA levels of *Dll1, Jagged1, Notch2, Notch3, Notch4* and *RBP-κB* were measured by qPCR. Western blotting **(E)** and quantitative analysis **(F)** of Jagged1, Notch2, Notch4 and RBP-κB protein. **(G)** In the BDL-induced rat liver fibrosis experiment, the mRNA levels of *Dll1, Dll4, Jagged1, Jagged2, Notch2, Notch3, Notch4* and *RBP-κB* were measured by qPCR. Western blotting **(H)** and quantitative analysis **(I)** of Jagged1, Notch2, Notch3 and RBP-κB protein. *p < 0.05, **p < 0.01, ***p < 0.001 vs the control group or sham group, ^#^ *p* < 0.05, ^##^ *p* < 0.01, ^###^ *p* < 0.001 vs the CCl_4_ group or BDL group. Oil: control group.

### JY5 Significantly Inhibits Activation of the Notch Signaling Pathway *In Vivo*

In the CCl_4_-induced liver fibrosis rat experiment, qPCR showed that the mRNA expressions of *Jagged1, Jagged2, Notch2, Notch3, Notch4* and *recombination signal binding protein-κB (RBP-κB)* were significantly more upregulated in the CCl_4_ group than those in the control group. However, compared to the CCl_4_ group, the mRNA expressions of *Jagged1, Jagged2, Notch2, Notch3, Notch4,* and *RBP-κB* were significantly reduced in the JY5- and SORA-treated groups (Figure 6A). Western blotting showed that the protein expression of Jagged1, Notch2, Notch3 and RBP-κB was significantly increased in the CCl_4_ group, compared to the control group. Above these proteins expression was significantly more reduced in the JY5- and SORA-treated groups than in the CCl_4_ group (Figures 6B,C). While in CCl_4_-induced liver fibrosis mice experiment, JY5 not only decreased the mRNA expressions of *Jagged1, Notch2, Notch3, Notch4* and *RBP-κB,* but also downregulated the expression of *Dll1* (Figure 6D). Western blotting showed that the proteins expression of Jagged1, Notch2, Notch4 and RBP-κB was significantly more reduced in the JY5- and SORA-treated groups than in the CCl_4_ group (Figures 6E,F). Consistent with the CCl_4_-induced rat liver fibrosis model, JY5 decreased the expressions of *Jagged1, Notch2, Notch3, Notch4* and *RBP-κB* in the BDL-induced liver fibrosis model. In addition, the mRNA expressions of *Dll1, Dll4* and *Jagged two* were significantly decreased after treatment with JY5 (Figures 6G–I). These results suggest that JY5 can significantly inhibit the activation of Notch signaling pathway in CCl_4_-and BDL-induced liver fibrosis.

### JY5 may Inhibit the Activation of LX-2 Cells Induced by TGF-β1 by Regulating the Notch Signaling Pathway

**FIGURE 7:**
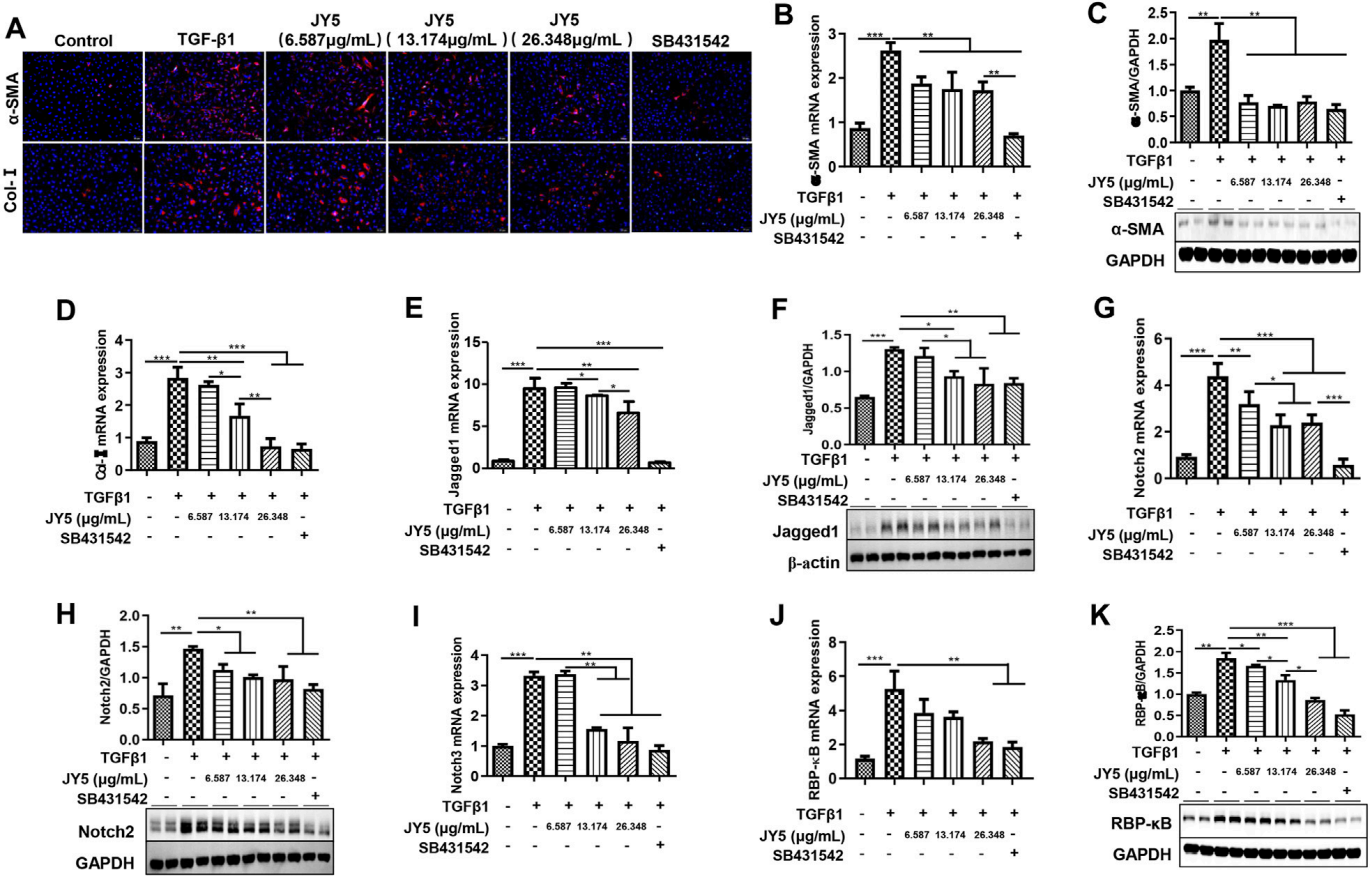
JY5 inhibits the activation of LX-2 cells induced by TGF-β1 by repressing the Notch signaling pathway. **(A)** Representative images of Immunofluorescence staining (100×) for *a*-SMA and Col-Ⅰ. The mRNA expressions of *α-SMA* **(B)** *, Col-I* **(D)** *, Jagged1* **(E)** *, Notch2* **(G)** *, Notch3* **(I)** and *RBP-κB* **(J)** were measured by qPCR. Western blotting and quantitative analysis of *a*-SMA (**C**), Jagged1 **(F)**, Notch2 **(H)** and RBP-κB **(K)** protein in cell lysate. *p < 0.05, **p < 0.01, and ***p < 0.001.

LX-2 cells were activated by TGF-β1 to observe the effect of JY5 at various concentrations *in vitro*. Immunofluorescence showed that both *a*-SMA^(+)^ cells and Col-Ⅰ^(+)^ cells were significantly elevated in TGF-β1-treated cells compared to the control cells. However, these positive cells were significantly reduced after treatment with various concentrations of JY5 and SB431542 (Figure 7A). qPCR showed that the mRNA levels of *α-SMA, Col-I, Jagged1, Notch2, Notch3* and *RBP-кB* were significantly elevated in TGF-β1-treated cells compared to the control cells. However, above these genes mRNA expression were significantly reduced after treatment with various concentrations of JY5. *Col-I, Jagged1, Notch2* and *Notch3* mRNA expressions were significantly decreased in the high-dose JY5 group, compared to the low-dose JY5 group (Figures 7B,D,E,G,I,J). Western blot analysis showed that the protein expressions of a-SMA, Jaggeed1, Nothc2 and RBP-κB were significantly increased after treatment with TGF-β1. Compared with the TGF-β1 group, above these proteins expression were significantly reduced in the JY5-treated groups. Of these, the protein expression of Jagged1 and RBP-κB was significantly reduced in the high-dose JY5 group, compared to the low-dose JY5 groups (Figures 7C,F,H,K). These results suggest that JY5 may inhibit the activation of LX-2 cells induced by TGF-β1 by regulating the Notch signaling pathway.

### Molecular Docking and molecular Dynamics Simulations

**FIGURE 8:**
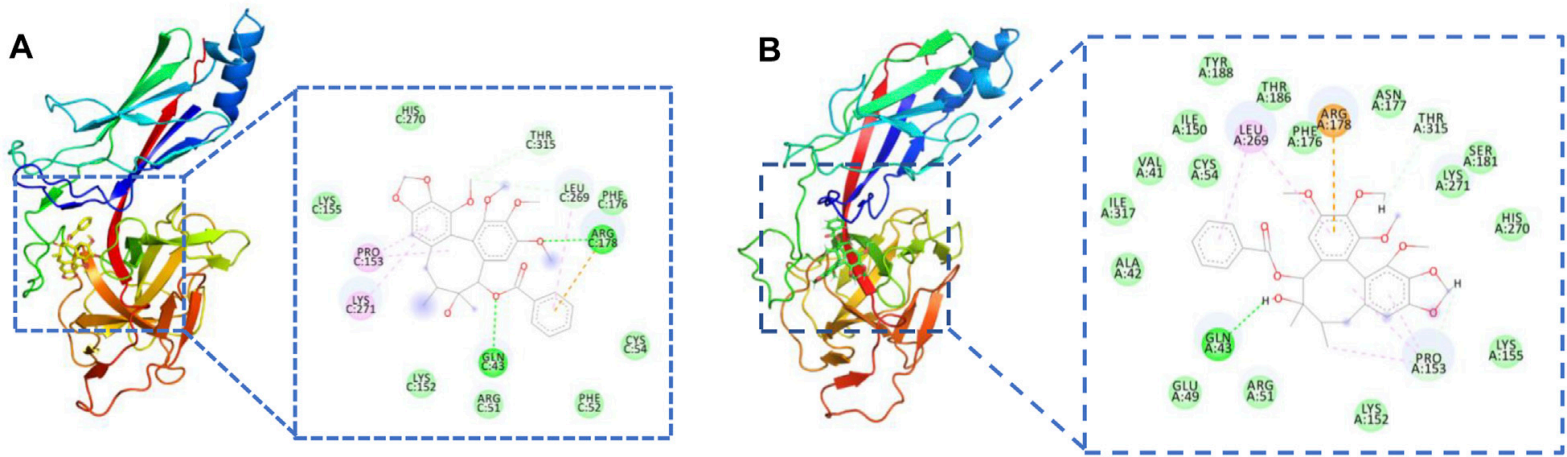
Molecular interactions between schisantherin A and the key residues of RBP-κB. **(A)** Molecular docking. **(B)** The molecular interactions after molecular dynamics simulations.

To investigate the binding mechanism of JY5 towards Jagged1, Notch2 and RBP-κB, molecular docking and molecular dynamics simulations was conducted. The lowest binding energy docking module was schisantherin A interacted with RBP-κB (Supplementary Table S4), and the intermolecular interactions have shown that schisantherin A has formed hydrogen bond with residues Gln43 and Arg178 of RBP-κB respectively (Figure 8A), and the results of molecular dynamics simulations also shown that schisantherin A has formed hydrogen bond with residues Gln43 (Supplementary Figure S5, Figure 8B), which suggested that RBP-κB may be the specific target by which JY5 regulated the Notch pathway.

**FIGURE 9:**
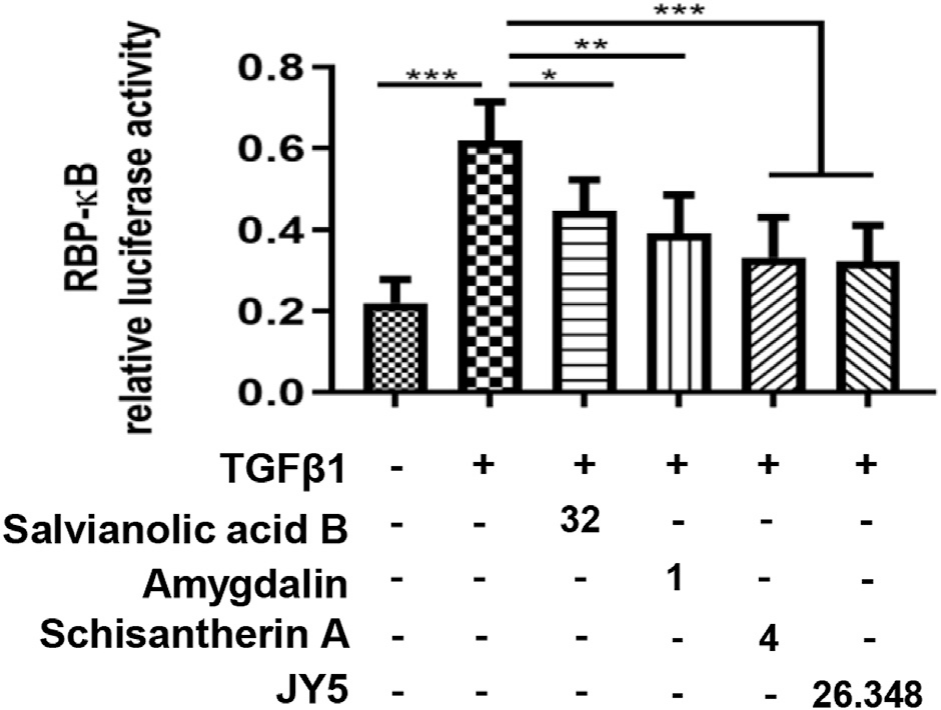
RBP-кB luciferase reporter activity. RBP-кB luciferase reporter activity in LX-2 cells after 24 h treatment with salvianolic acid B (32 μM), amygdalin (1 μM), schisantherin A (4 μM) or JY5 (26.348 μg/ml). *p < 0.05, **p < 0.01, ***p < 0.001.

We proceeded to test whether JY5-mediated RBP-κB was accompanied by transcriptional activation of RBP-κB using luciferase reporter assay. Exposure of LX-2 cells to TGF-β1 for 24 h resulted in a statistically significant increase in RBP-κB luciferase reporter activity (Figure 9), however, after simultaneously treated with salvianolic acid B, amygdalin, schisantherin A or JY5 for 24 h (Figure 9), the RBP-κB luciferase reporter activity was reduced, which further suggested that RBP-κB may be the target by which JY5 regulated the Notch pathway.

## Discussion

Liver fibrosis is an abnormal repair response to tissue damage, characterized by excessive deposition of ECM, leading to the persistence and development of pathological scar. Hepatic fibrosis is common in most chronic liver diseases process, which is a clinically important problem. To develop effective anti-hepatic fibrotic drugs, researchers have conducted a large number of basic and clinical studies. Despite achieving certain results in recent years, most of these drugs are in the preclinical or clinical trials stage, and some have failed clinical trials due to severe toxic side effects (Yoon et al., 2016). TCM has marked clinical effects on the treatment of liver fibrosis, which are closely correlated with its characteristics of multi-ingredients compatibility and multi-targets. However, the components of TCM are complex, and their mechanisms are not very clear, which somewhat increases the complexity of TCM studies. Regarding the intensive development of multidisciplinary crossover study, active ingredients screening, extraction, and purification from Chinese herbs provide a new approach for TCM formula research.

Salvianolic acid B is the main water-soluble phenolic acid compound. Numerous studies (Yan et al., 2010; Wang et al., 2012; Tao et al., 2013; Yan et al., 2017) have indicated that salvianolic acid B exerts significant anti-hepatic fibrosis effects through the following mechanisms: inhibiting the activation of HSCs by downregulating the TGF-β1/SMAD signaling pathway, protecting hepatocytes from apoptosis via inhibiting death receptor pathway, and stabilizing the mitochondrial membrane and regulating NF-κB/IκBα signaling pathway. Amygdalin is the major ingredient of peach kernel. Amygdalin can inhibit the activation of HSCs by downregulating the TGF-β/CTGF signaling pathway and induce activated HSC apoptosis by upregulating Bax gene expression, subsequently exerting anti-hepatic fibrotic effects (Luo et al., 2016; Luo et al., 2018). Lignans are the main bioactive components of Schisandrae. Studies have shown that these lignans can suppress inflammation, protect hepatocytes, and inhibit the activation of HSCs by downregulating the TGFβ/SMAD and MAPK signaling pathways (He et al., 2020).

In this study, we measured the content of various compounds in FZHY extract, plasma, and liver in rats after intragastric administration of FZHY. We obtained the three main bioactive ingredients of FZHY: salvianolic acid B, which had the highest content in FZHY extract; schisantherin A, which had maximum exposure in plasma; and amygdalin, which had highest hepatic exposure in the liver. Then we conducted uniform design and validation experiments to explore their composition and determine the best ratio for treating the rat hepatic fibrosis model. We obtained a new formula, namely JY5, which had anti-fibrotic effects comparable to that of FZHY. Further studies have shown that JY5 can significantly decrease serum ALT and AST levels and inhibit inflammation reaction while reducing collagen deposition in CCl_4_-or BDL-induced liver fibrosis models.

The activation of HSCs is a pivotal event in liver fibrosis. Under persistent stimulation from CCl_4_ and BDL, HSCs are largely activated and transformed into myofibroblasts, which cause excessive ECM accumulated in the liver, eventually leading to hepatic fibrosis formation. Activated HSCs, as one of the main sources of hepatic ECM, can secrete Col*-*I and Col*-*III proteins. *a*-SMA is a specific marker of activated HSCs. This study found that JY5 significantly reduced the mRNA and protein expression of *a*-SMA, and decreased *Col-I* mRNA expression in CCl_4_-and BDL-induced liver fibrosis in rats and mice. The results were further confirmed in TGF-β1-induced LX-2 cell activation. JY5 significantly downregulated the expressions of *a*-SMA and *Col-I* in activated LX-2 cells induced by TGF-β1. These results suggest that JY5 significantly inhibits the activation of HSCs.

Notch signaling is a highly conserved pathway evolutionarily, which influences intercellular signal transduction and cell fate decisions, and regulates the growth and development homeostasis of multiple tissues and organs, and in particular, the progression and development of diseases (Siebel and Lendahl, 2017). The Notch signaling pathway mainly consists of four Notch receptors (Notch1, Notch2, Notch3, Notch4), five Notch ligands (Jagged1, Jagged2, Dll1, Dll3, Dll4), and the transcriptional regulatory elements of downstream signals (D’Souza et al., 2010). Previous studies (Ni et al., 2018) have shown that Notch plays an important role in the progress and development of hepatic fibrosis, which can interact with other signaling pathways such as TGF-β, Hedgehog, and Hippo. TGF-β1 can promote the proliferation and activation of HSC-T25 cells by regulating the Notch signaling pathway (Aimaiti et al., 2019). The Jagged1 gene was successfully knocked down by using rAAV1-Jagged1-shRNA in CCl_4_-induced liver fibrosis, resulting in alleviation of liver fibrosis (Tang et al., 2017). In addition, knockout of the RBP-κB gene, which is considered a key transcription factor in the Notch pathway, can inhibit the proliferation and activation of HSCs to alleviate CCl_4_-induced liver fibrosis in mice (He et al., 2015). Blockade of the Notch pathway can effectively inhibit the activation of HSCs, which in turn attenuates liver fibrosis. In this study, the expressions of Jagged1, Jagged2, Notch2, Notch3, Notch4, and RBP-κB were significantly increased in CCl_4_-and BDL-induced liver fibrosis in rats and mice. While after intervention with JY5, the expressions of these Notch-related genes and proteins were significantly decreased. This was further confirmed by LX-2 cell activation induced by TGF-β1 experiments *in vitro*. These results suggest that JY5 might exert anti-fibrotic effects by regulating the Notch signaling pathway to inhibit the activation of HSCs.

In this study, we used SORA and DAPT as positive controls. SORA, as a multi-receptor tyrosine kinase inhibitor that can inhibit the proliferation of multiple tumor cells and promote cell apoptosis, is commonly used for the treatment of hepatocellular carcinoma clinically (Keating, 2017). DAPT, as a γ-secretase inhibitor, can block the release of the Notch intracellular domain, thereby inhibiting activation of the Notch signaling pathway (Ni et al., 2018). In accordance with previous studies (Deng et al., 2013; Zhang et al., 2016), our results show that both SORA and DAPT have significant anti-hepatic fibrosis effects. Interestingly, SORA also can significantly downregulate the mRNA expressions of *Jagged1, Jagged2, Notch2, Notch3, Notch4* and *RBP-κB*, and the protein expression of RBP-κB. We speculate that SORA exerts anti-hepatic fibrosis effects, which might be related to the regulation of the Notch pathway; however, the precise mechanism needs to be further investigated. In addition, DAPT inhibits liver fibrosis by blocking the Notch pathway (Chen et al., 2012). This conclusion was further confirmed in our study. However, it is important to note that *γ*-secretase inhibitors, as a nonspecific Notch blocker, have shown severe side effects in clinical trials (Ni et al., 2018). So the clinical application of DAPT may be limited. Compared with SORA and DAPT, JY5 may have synergistic anti-fibrosis effects via multiple pathways, not specifically blocking a particular target or pathway, which confers relatively higher safety and more effectiveness.

The results of molecular docking, molecular dynamics simulations and RBP-кB luciferase reporter assay suggested that RBP-κB may be the specific target by which JY5 regulated the Notch pathway. In addition, given that JY5, as a component of TCM compounds, has shown good efficacy in two different liver fibrosis models, we speculate that JY5 might alleviate hepatic fibrosis through other mechanisms. In response to these issues, a series of related studies to elaborate upon the compatibility mechanisms of anti-liver fibrosis effect of JY5 will be conducted. These studies will provide an adequate scientific basis for clinical research and application of JY5 formula.

## Conclusion

**FIGURE 10:**
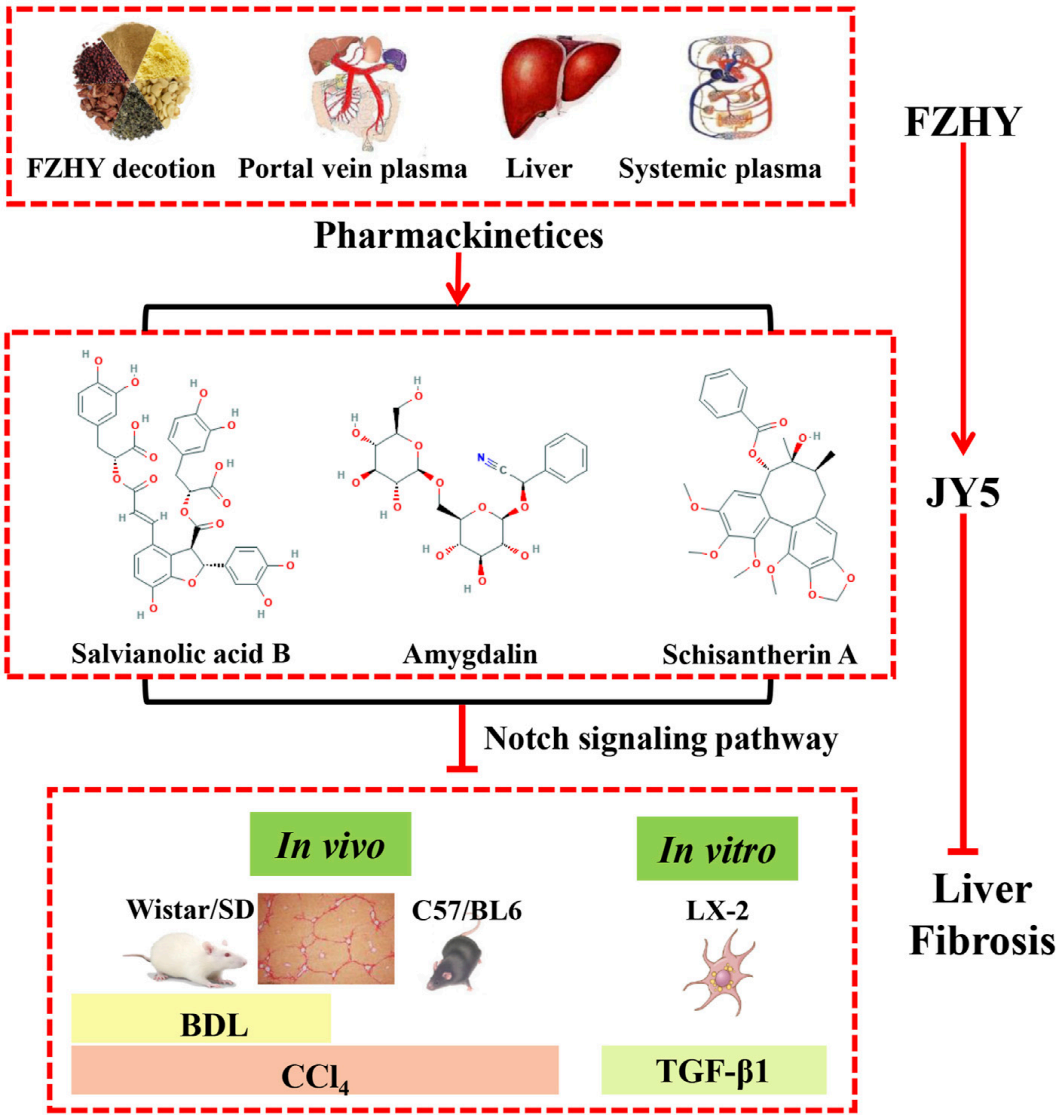
A brief diagram of this study was shown. JY5 formula, which consists of the main active ingredients of FZHY, could exert anti-hepatic fibrotic effect through regulating Notch signaling pathway and inhibiting the activation of HSCs.

In summary, we obtained a novel anti-hepatic fibrosis component of TCM compounds, namely, JY5, through uniform design and validation experiments *in vivo*, and explored its part of mechanisms for the first time. Our study showed that JY5 may exert anti-hepatic fibrotic effects by regulating the Notch signaling pathway to inhibit the activation of HSCs (Figure 10). Thus, the JY5 formula may be a potential novel therapeutic candidate for liver fibrosis.

## Data Availability

The raw data supporting the conclusion of this article will be made available by the authors, without undue reservation.

## Abbreviations

FZHY, fuzheng huayu formula; SORA, sorafenib; HSCs, hepatic stellate cells; ECM, extracellular matrix; TCM, traditional Chinese medicine; KCs, kupffer cells; LSECs, liver sinusoidal endothelial cells; CCl4, carbon tetrachloride; BDL, bile duct of ligation; ALT, alanine aminotransferase; AST, aspartate aminotransferase; ALP, alkaline phosphatase; TBil, total bilirubin; DBil, direct bilirubin; TBA, total bile acids; Hyp, hydroxyproline; IHC, immunohistochemical; H&E, hematoxylin & eosin; SR, sirius red; α-SMA , α-smooth muscle actin; RBP-κB, recombination signal binding protein-κB; HCC, hepatocellular carcinoma; NICD, notch intracellular domain.
