# Correction: The novel Chinese medicine JY5 formula alleviates hepatic fibrosis by inhibiting the notch signaling pathway

**DOI:** 10.3389/fphar.2026.1854159

**Published:** 2026-05-18

**Authors:** Yadong Fu, Zhun Xiao, Xiaoting Tian, Wei Liu, Zhou Xu, Tao Yang, Yonghong Hu, Xiaoxi Zhou, Jing Fang, Siqi Gao, Dingqi Zhang, Yongping Mu, Hua Zhang, Yiyang Hu, Chenggang Huang, Jiamei Chen, Ping Liu

**Affiliations:** 1 Key Laboratory of Liver and Kidney Diseases (Ministry of Education), Institute of Liver Diseases, Shuguang Hospital Affiliated to Shanghai University of Traditional Chinese Medicine, Shanghai, China; 2 Institute of Interdisciplinary Integrative Medicine Research, Shanghai University of Traditional Chinese Medicine, Shanghai, China; 3 Shanghai Key Laboratory of Traditional Chinese Clinical Medicine, Shanghai, China; 4 Shanghai Institute of Materia Medica, Chinese Academy of Sciences, Shanghai, China; 5 Department of Cardiology, Cardiovascular Research Institute, Shuguang Hospital Affiliated to Shanghai University of Traditional Chinese Medicine, Shanghai, China

**Keywords:** Fuzheng Huayu, hepatic fibrosis, JY5 formula, notch signaling pathway, traditional Chinese medicine

There was a mistake in Figure 4 as published. Figure 4G presents the Col-IV immunohistochemistry results. During data organization and figure preparation, a clerical oversight in file naming led to the image intended for the JY5 group being inadvertently replaced with that of the DAPT group. This error occurred purely by accident during figure assembly. This error affected only the final placement of the image; all quantitative analyses were performed using accurate, original data. Therefore, the correction does not alter any of the study’s results, scientific conclusions, or data interpretations. The corrected Figure 4 appears below.

**FIGURE 4:**
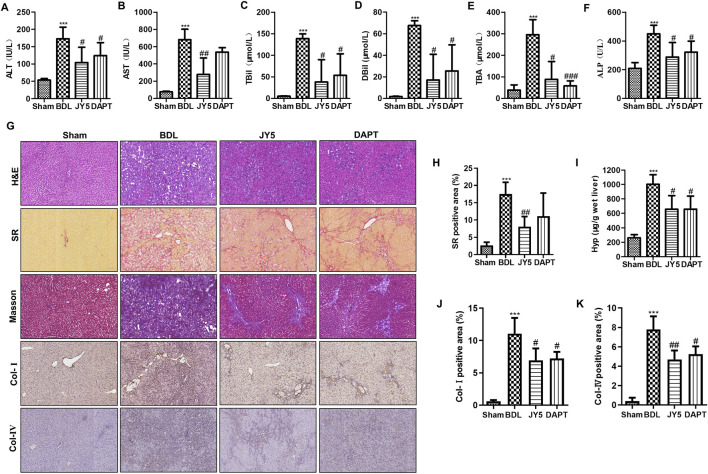
JY5 significantly alleviates hepatic inflammatory injury and collagen deposition in BDL-induced rat liver fibrosis. The levels of serum ALT **(A)**, AST **(B)**, TBil **(C)**, DBil **(D)**, TBA **(E)**, and ALP **(F)** were measured in each group. **(G)** H&E staining (100×), SR staining (100×), Masson staining (100×) and IHC for Col-I and Col-IV (100×) staining, and semi-quantitative analysis **(H)** of collagen disposition (%) in SR-stained liver sections. **(I)** Hyp content in wet liver tissue was detected by alkaline hydrolysis. Quantitative analysis of immunohistochemical staining for Col-I **(J)** and Col-IV **(K)**. ***p < 0.001 vs the sham group, # p < 0.05, ##p < 0.01 vs the BDL group.

The original article has been updated.

