# Supplementary figures and images for: The Novel Chinese Medicine JY5 Formula Alleviates Hepatic Fibrosis by Inhibiting the Notch Signaling Pathway (part 1 of 2)
Source: Front Pharmacol. 2021 Sep 22;12:671152. doi: 10.3389/fphar.2021.671152 (PMC8493219; doi:10.3389/fphar.2021.671152)

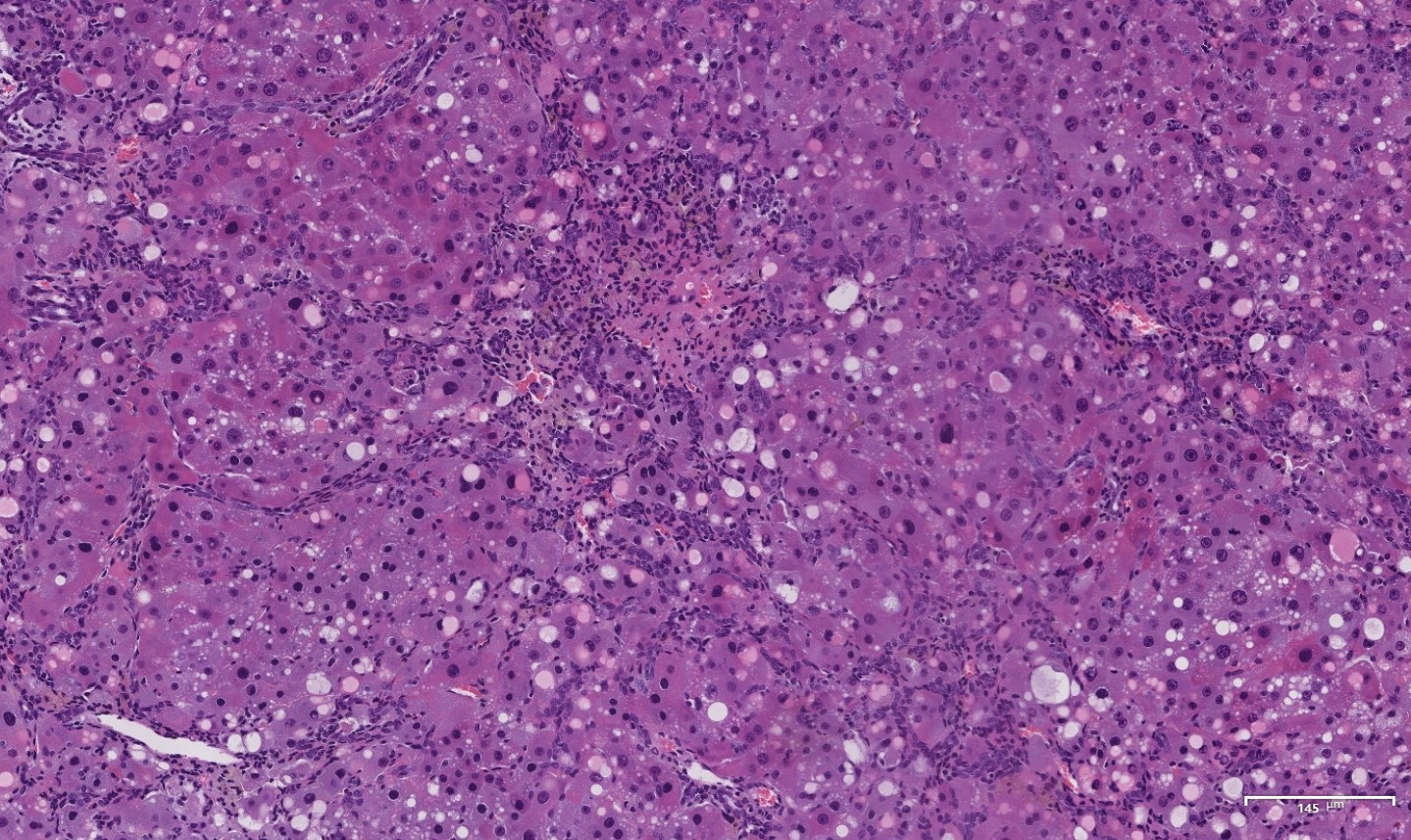

Supplement: Supplementary file 1 [file DataSheet3.ZIP › the original source data of Figures 1-4/Fig. 2/Fig. 2C (Rat-CCl4)/H&E/CCl4.jpg]

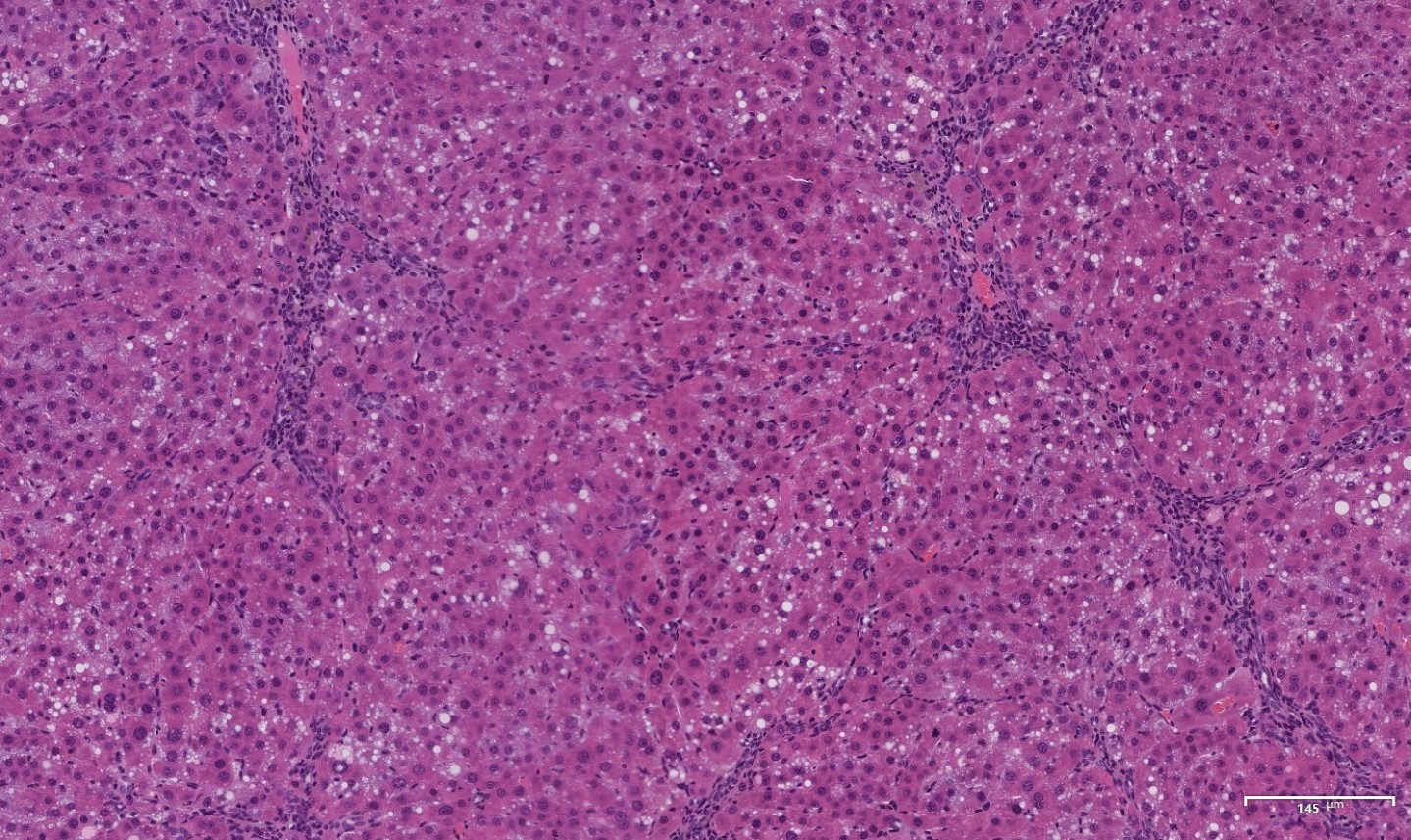

Supplement: Supplementary file 1 [file DataSheet3.ZIP › the original source data of Figures 1-4/Fig. 2/Fig. 2C (Rat-CCl4)/H&E/JY5.jpg]

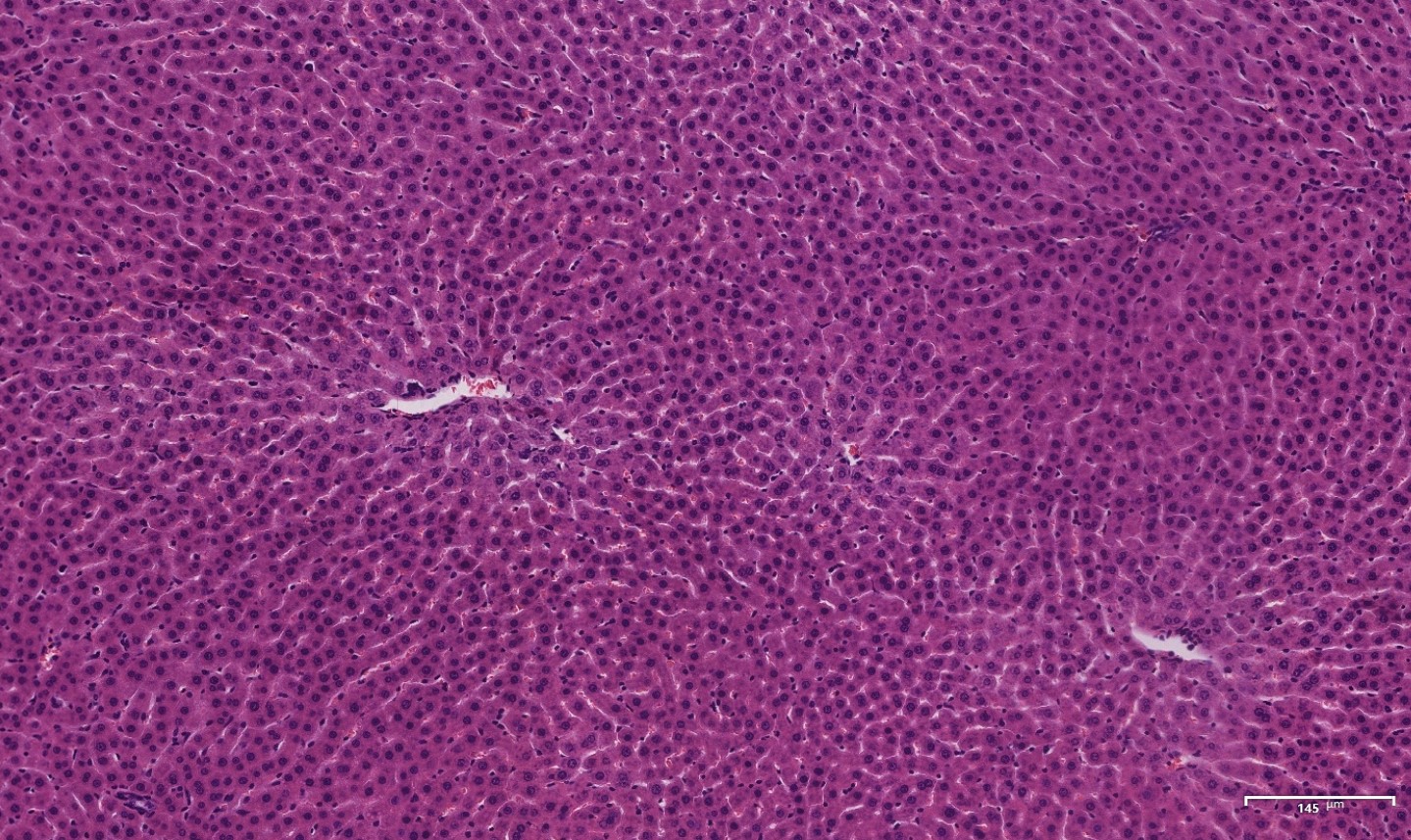

Supplement: Supplementary file 1 [file DataSheet3.ZIP › the original source data of Figures 1-4/Fig. 2/Fig. 2C (Rat-CCl4)/H&E/Oil.jpg]

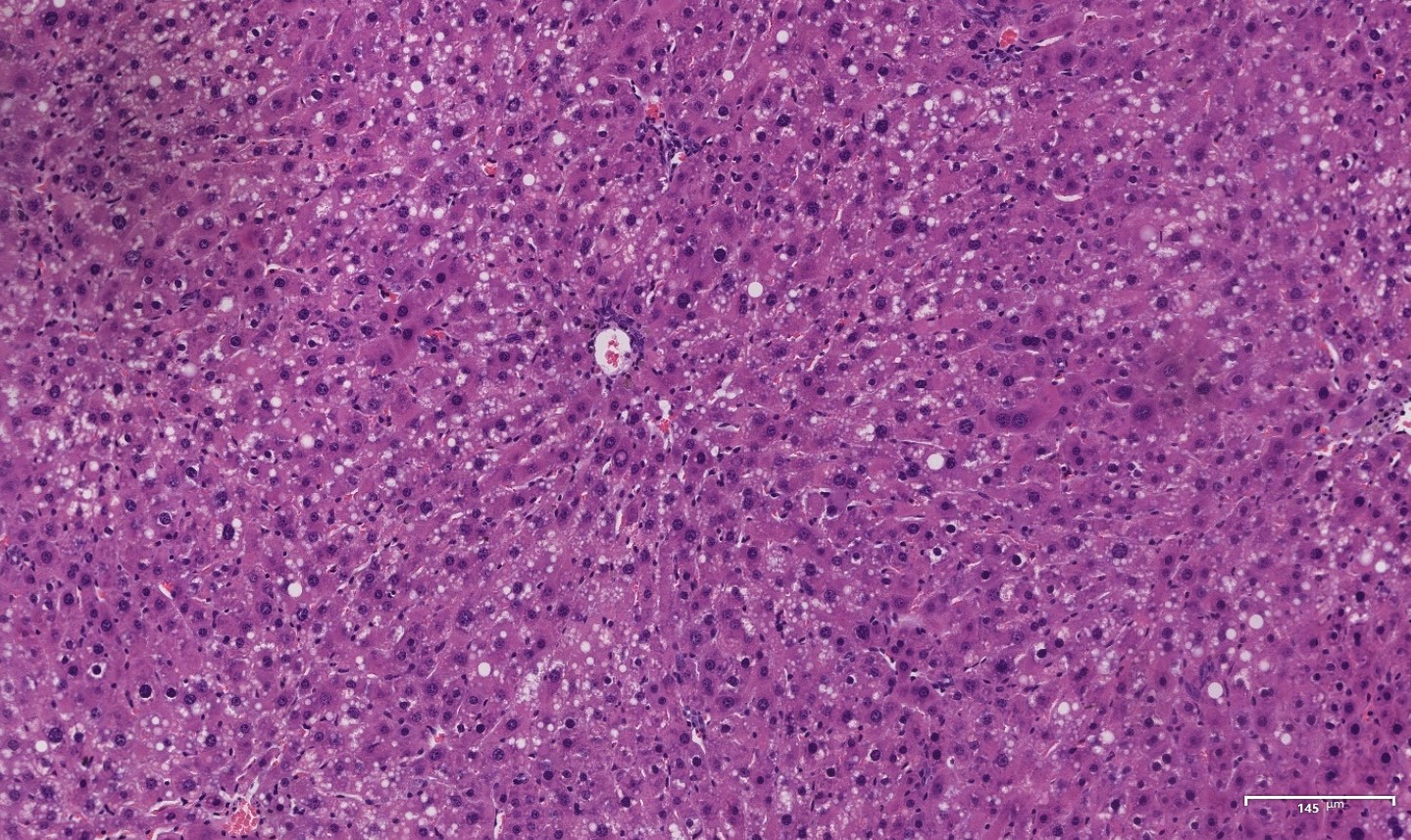

Supplement: Supplementary file 1 [file DataSheet3.ZIP › the original source data of Figures 1-4/Fig. 2/Fig. 2C (Rat-CCl4)/H&E/SORA.jpg]

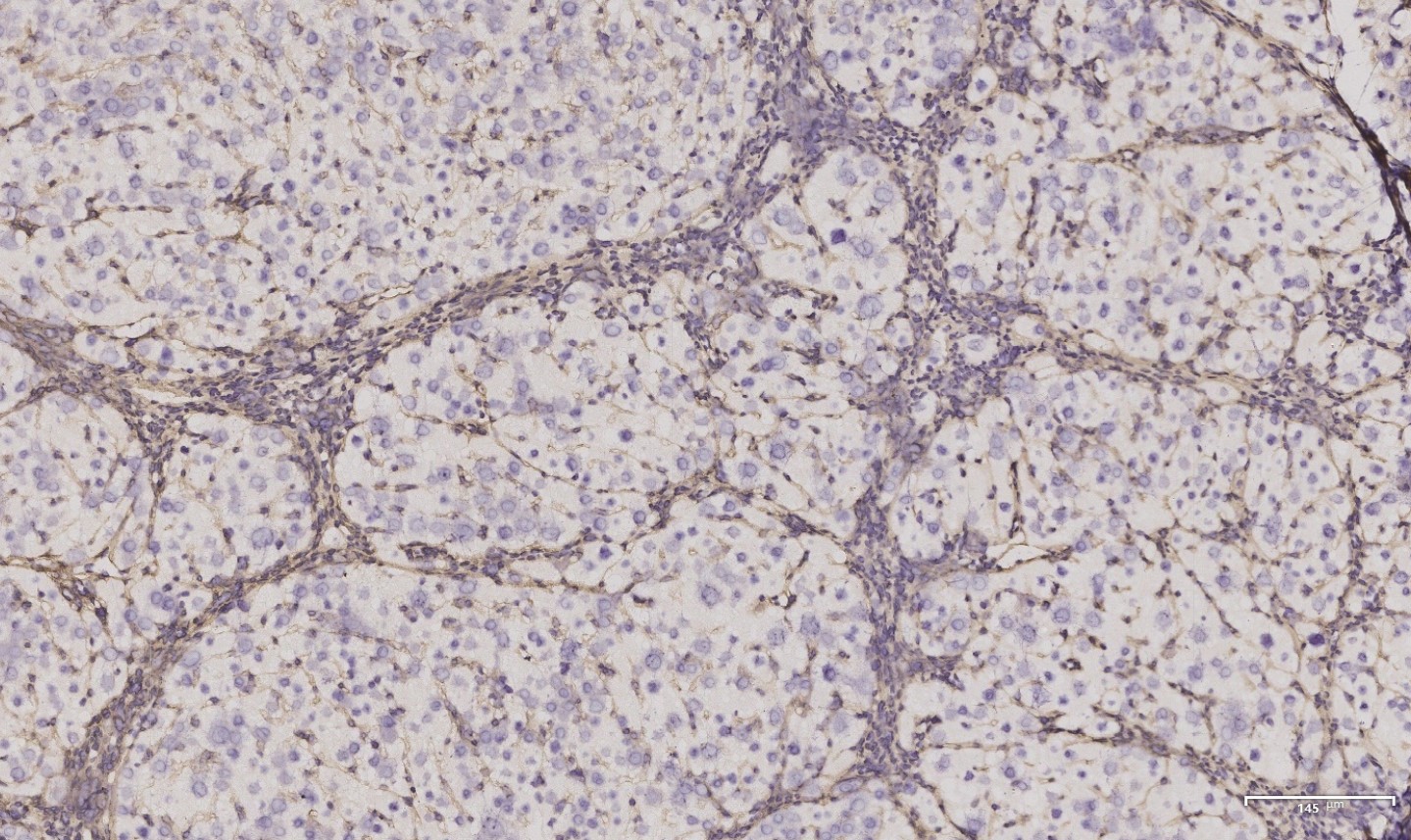

Supplement: Supplementary file 1 [file DataSheet3.ZIP › the original source data of Figures 1-4/Fig. 2/Fig. 2C (Rat-CCl4)/IHC-Col-Γàá/CCl4.jpg]

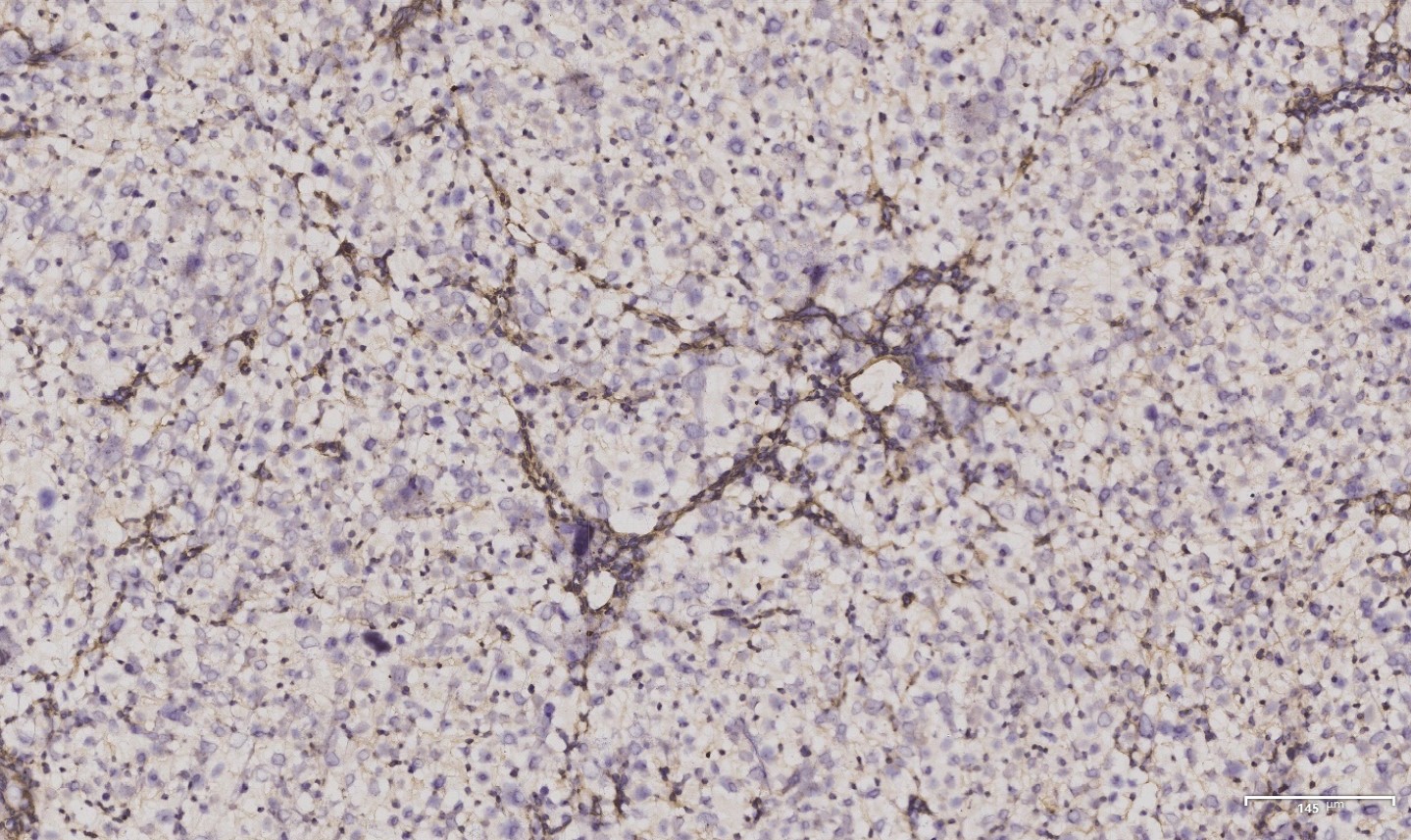

Supplement: Supplementary file 1 [file DataSheet3.ZIP › the original source data of Figures 1-4/Fig. 2/Fig. 2C (Rat-CCl4)/IHC-Col-Γàá/JY5.jpg]

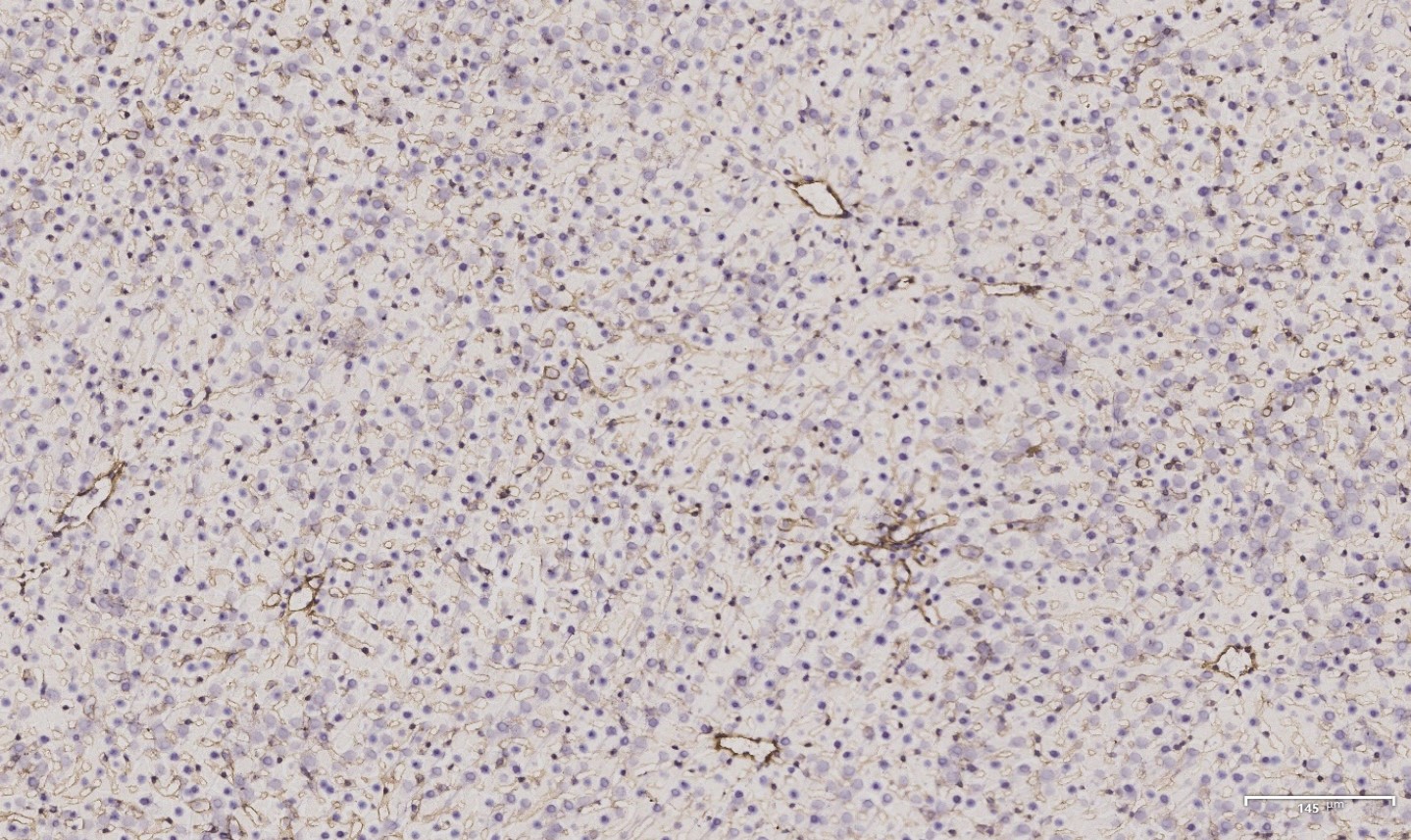

Supplement: Supplementary file 1 [file DataSheet3.ZIP › the original source data of Figures 1-4/Fig. 2/Fig. 2C (Rat-CCl4)/IHC-Col-Γàá/Oil.jpg]

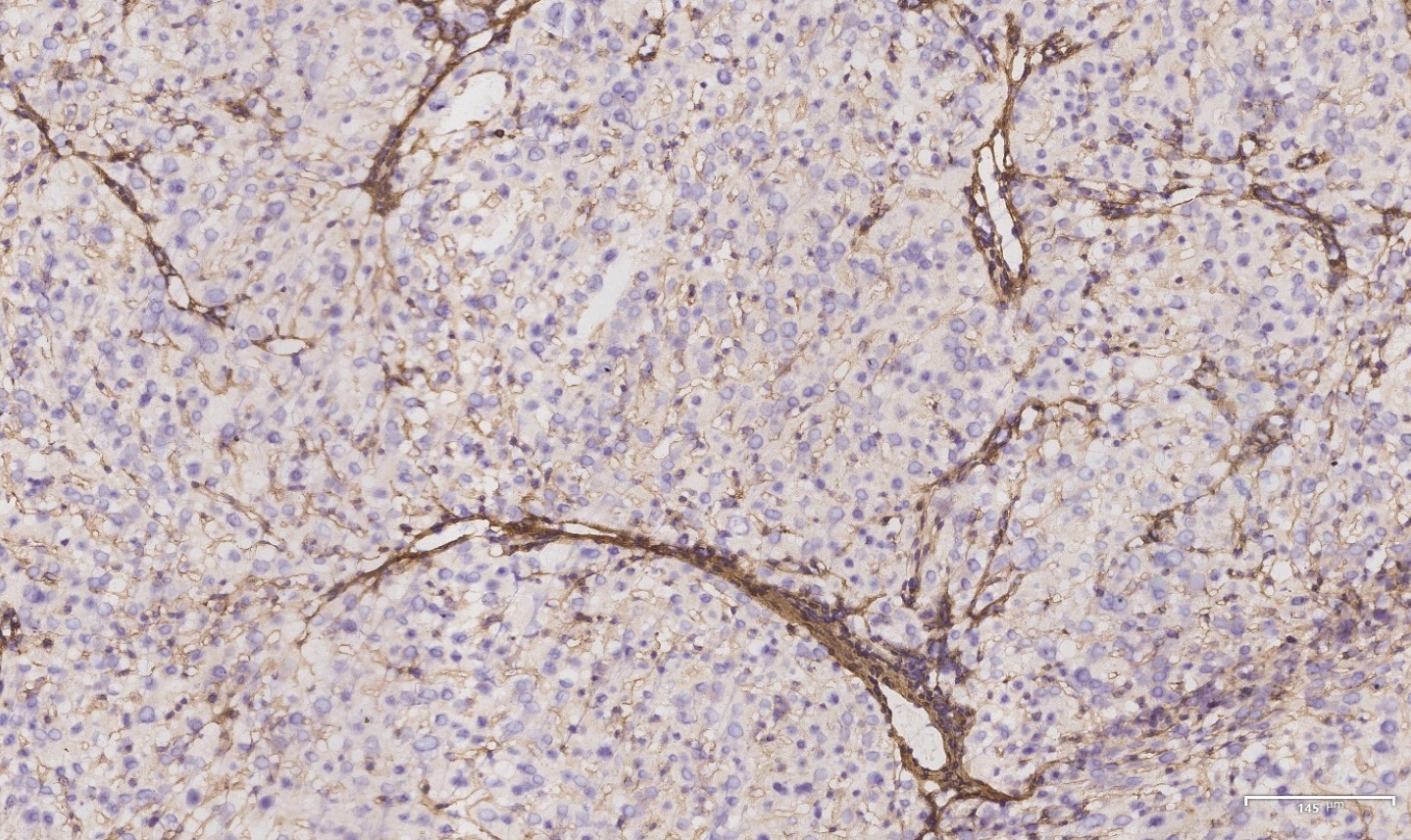

Supplement: Supplementary file 1 [file DataSheet3.ZIP › the original source data of Figures 1-4/Fig. 2/Fig. 2C (Rat-CCl4)/IHC-Col-Γàá/SORA.jpg]

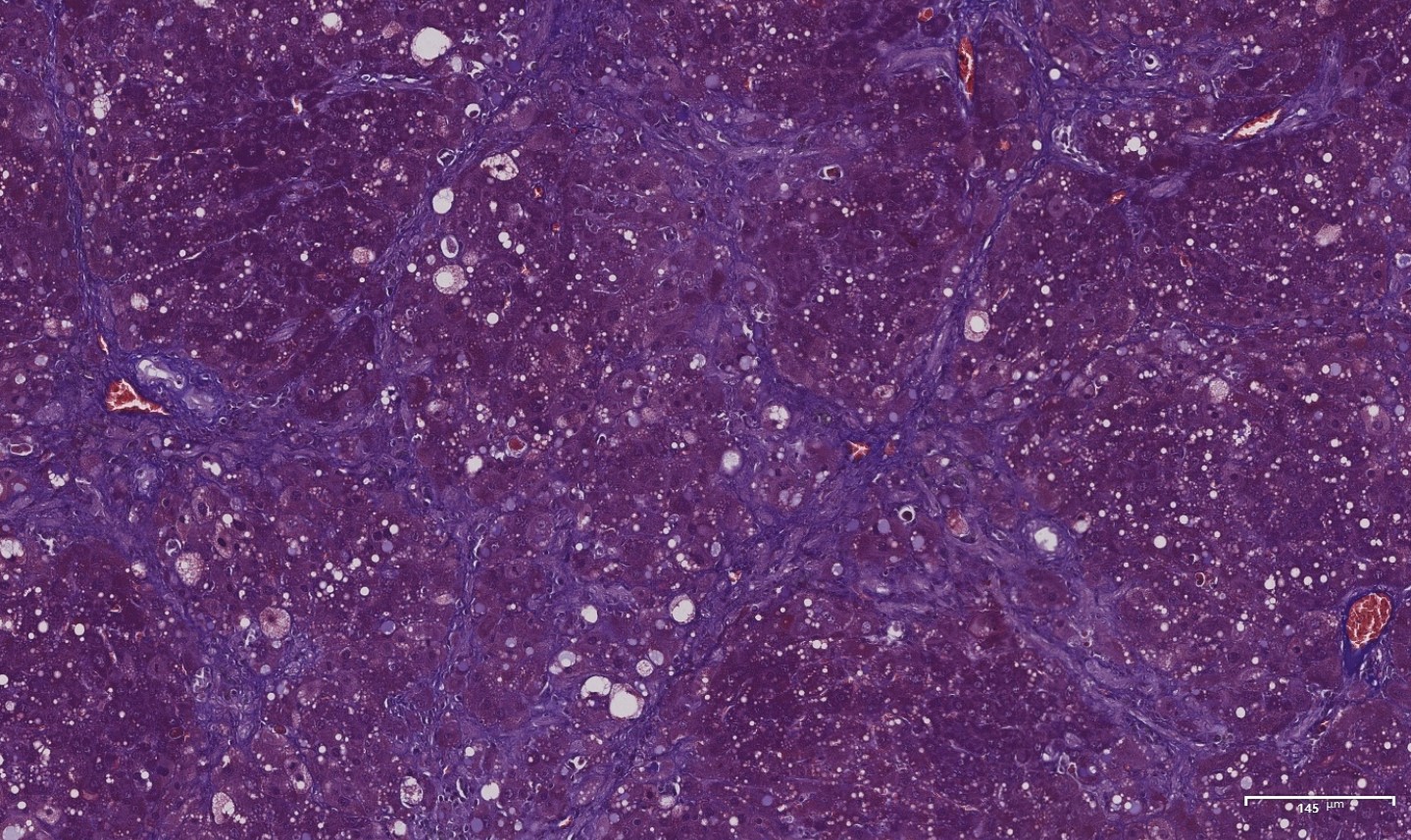

Supplement: Supplementary file 1 [file DataSheet3.ZIP › the original source data of Figures 1-4/Fig. 2/Fig. 2C (Rat-CCl4)/Masson/CCl4.jpg]

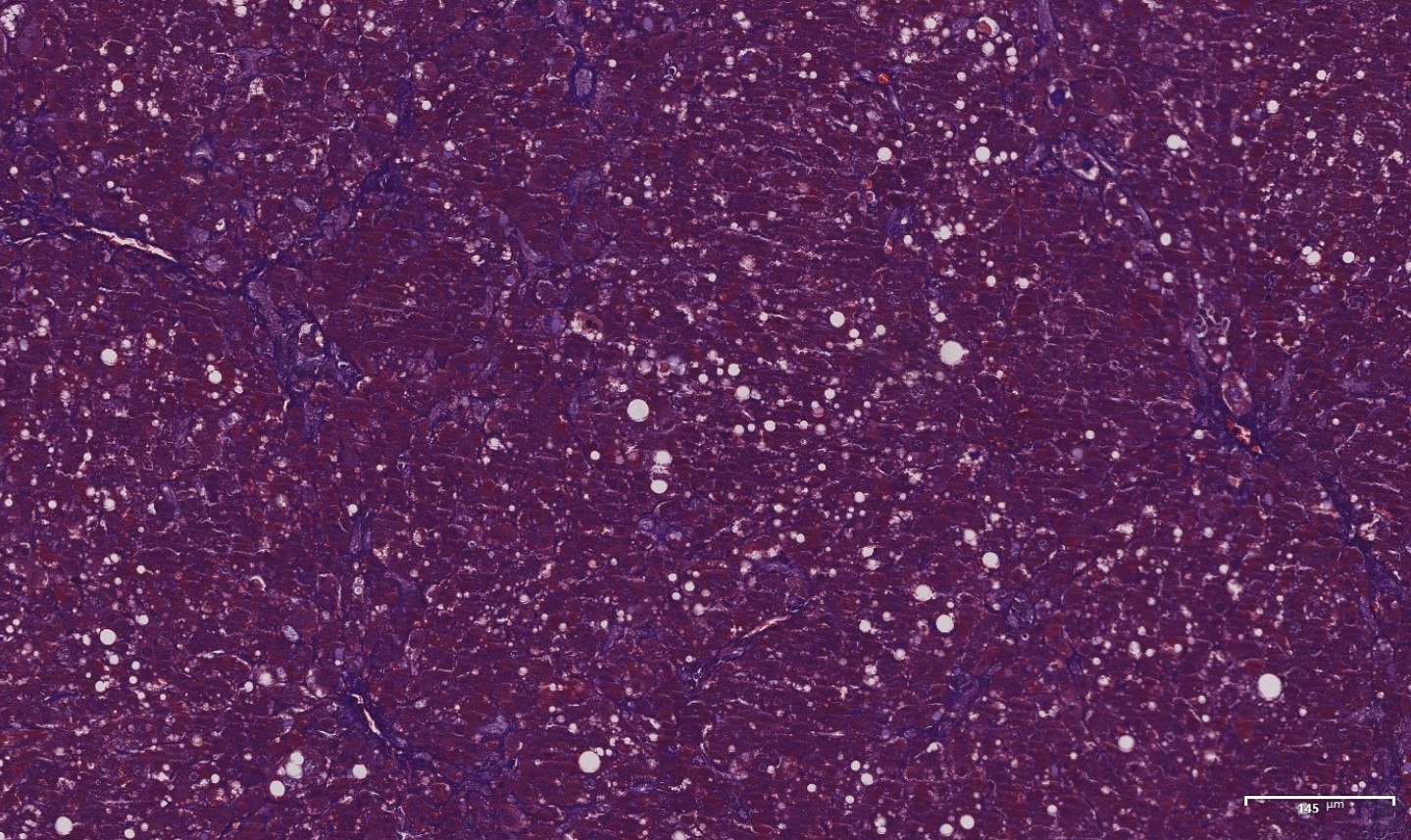

Supplement: Supplementary file 1 [file DataSheet3.ZIP › the original source data of Figures 1-4/Fig. 2/Fig. 2C (Rat-CCl4)/Masson/JY5.jpg]

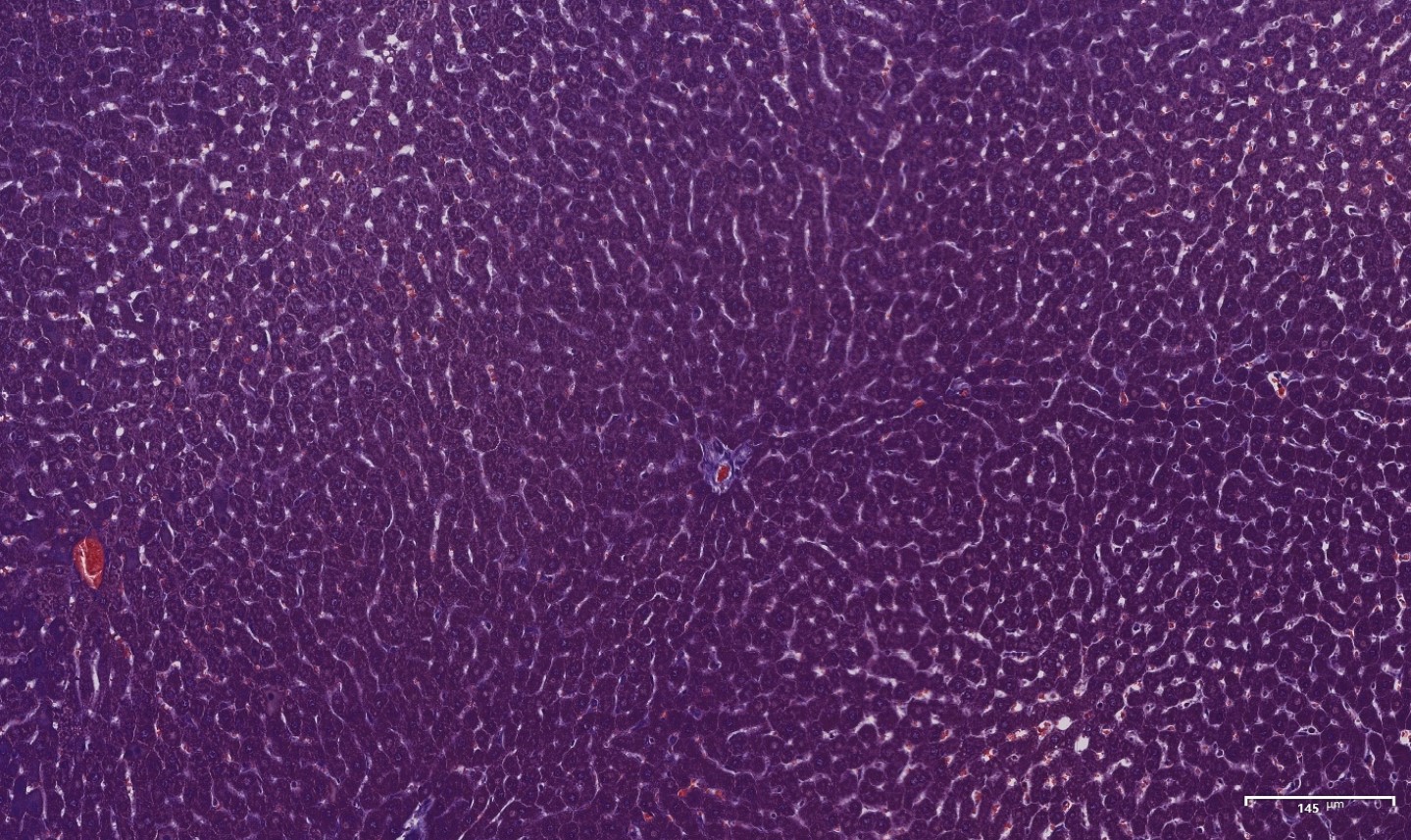

Supplement: Supplementary file 1 [file DataSheet3.ZIP › the original source data of Figures 1-4/Fig. 2/Fig. 2C (Rat-CCl4)/Masson/Oil.jpg]

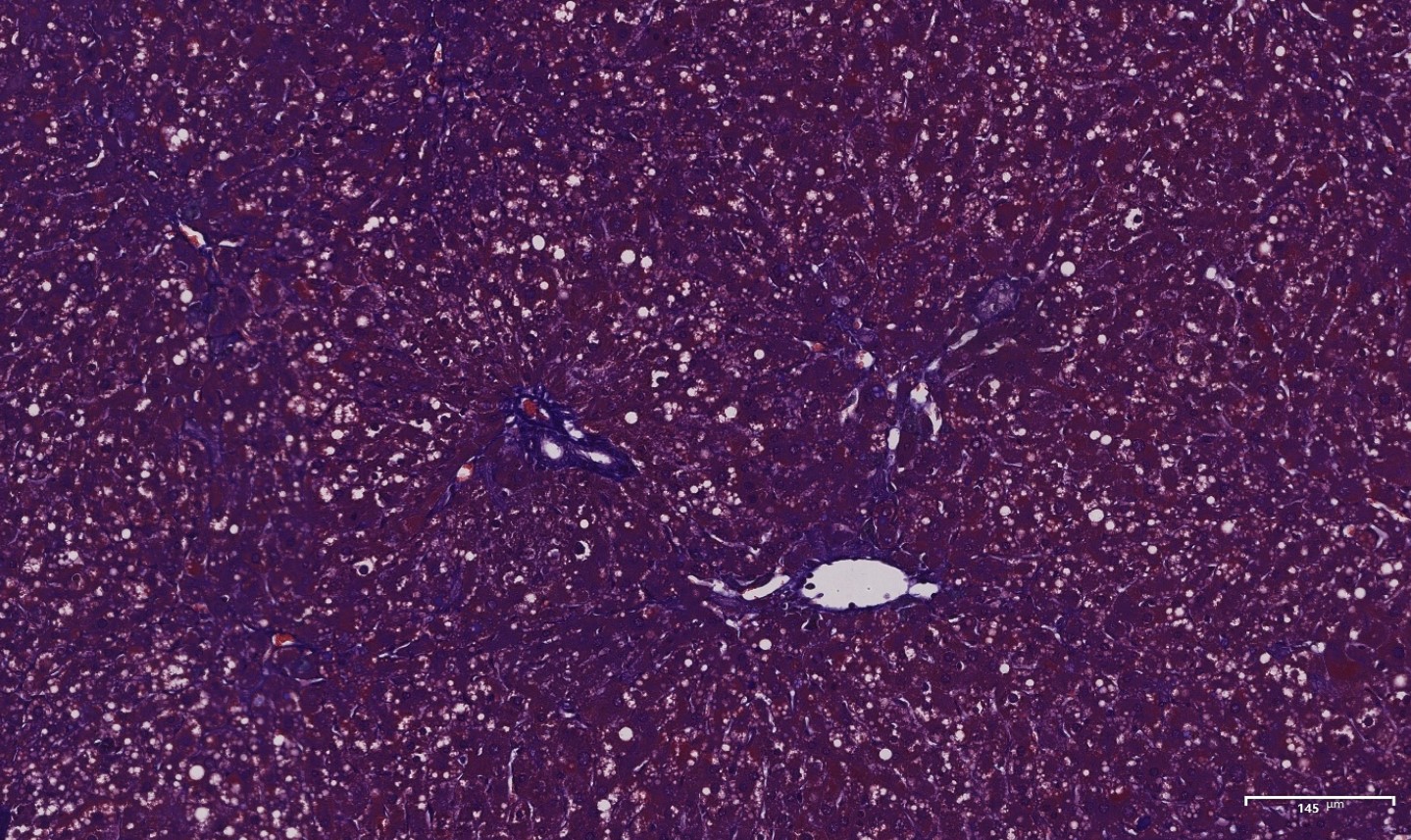

Supplement: Supplementary file 1 [file DataSheet3.ZIP › the original source data of Figures 1-4/Fig. 2/Fig. 2C (Rat-CCl4)/Masson/SORA.jpg]

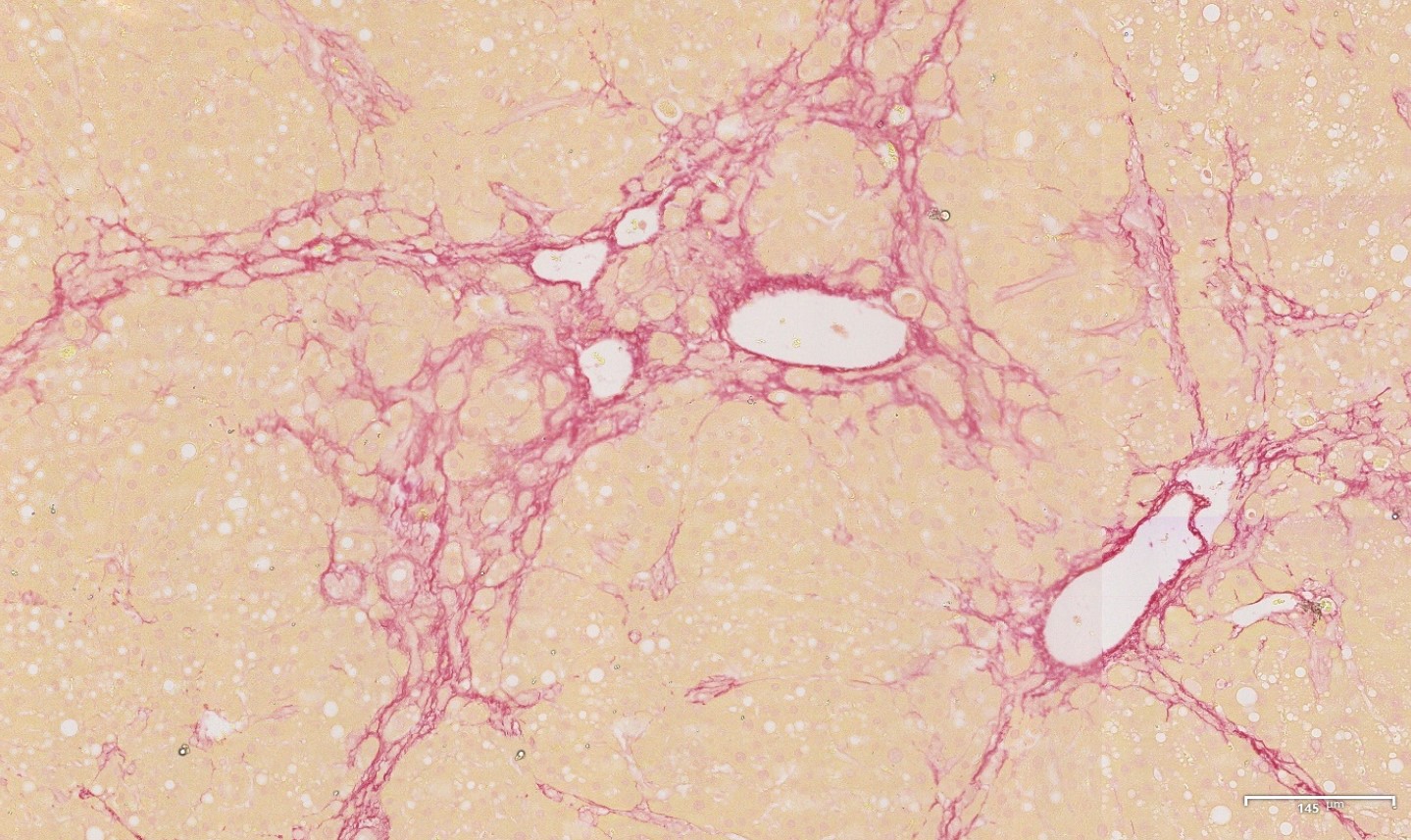

Supplement: Supplementary file 1 [file DataSheet3.ZIP › the original source data of Figures 1-4/Fig. 2/Fig. 2C (Rat-CCl4)/SR/CCl4.jpg]

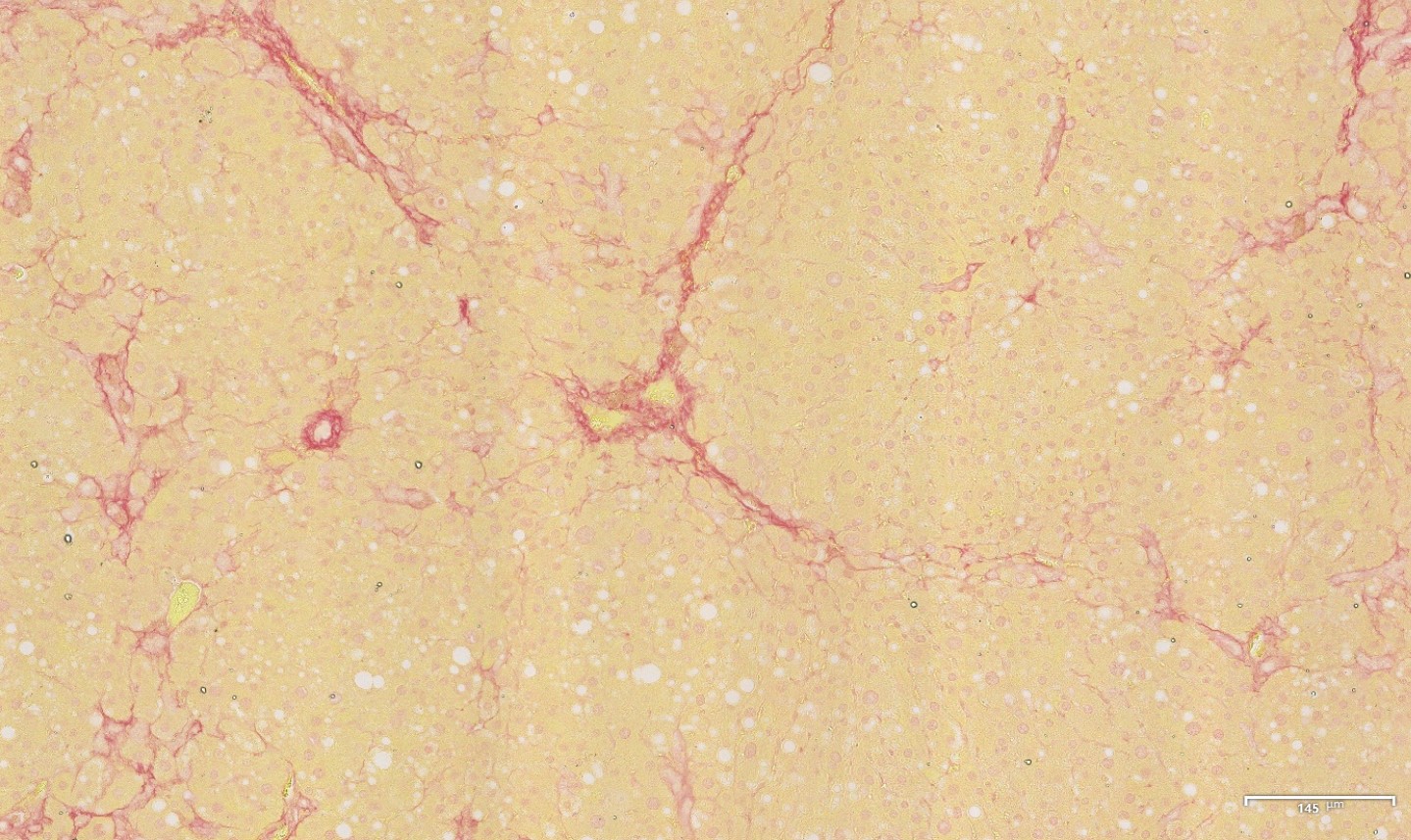

Supplement: Supplementary file 1 [file DataSheet3.ZIP › the original source data of Figures 1-4/Fig. 2/Fig. 2C (Rat-CCl4)/SR/JY5.jpg]

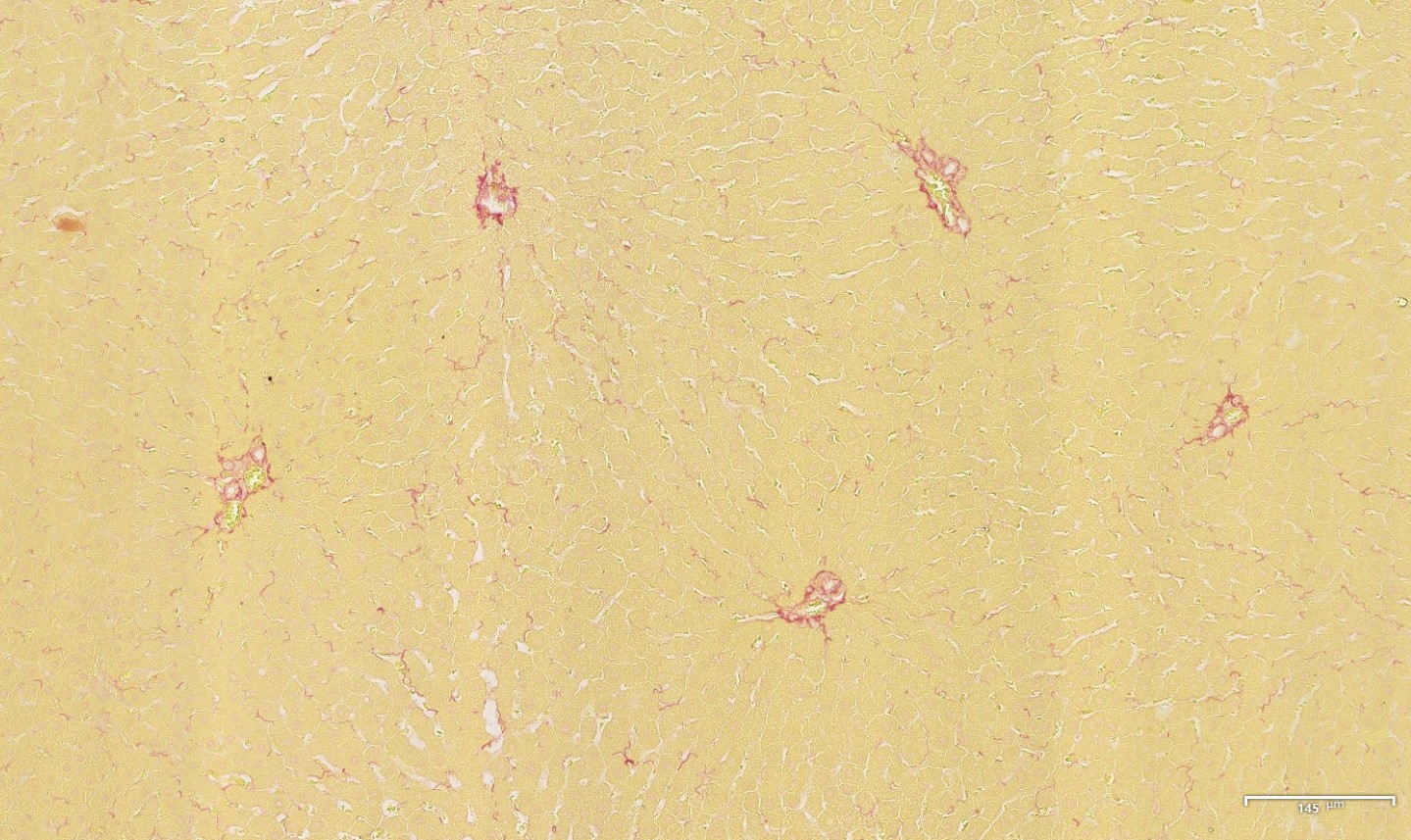

Supplement: Supplementary file 1 [file DataSheet3.ZIP › the original source data of Figures 1-4/Fig. 2/Fig. 2C (Rat-CCl4)/SR/Oil.jpg]

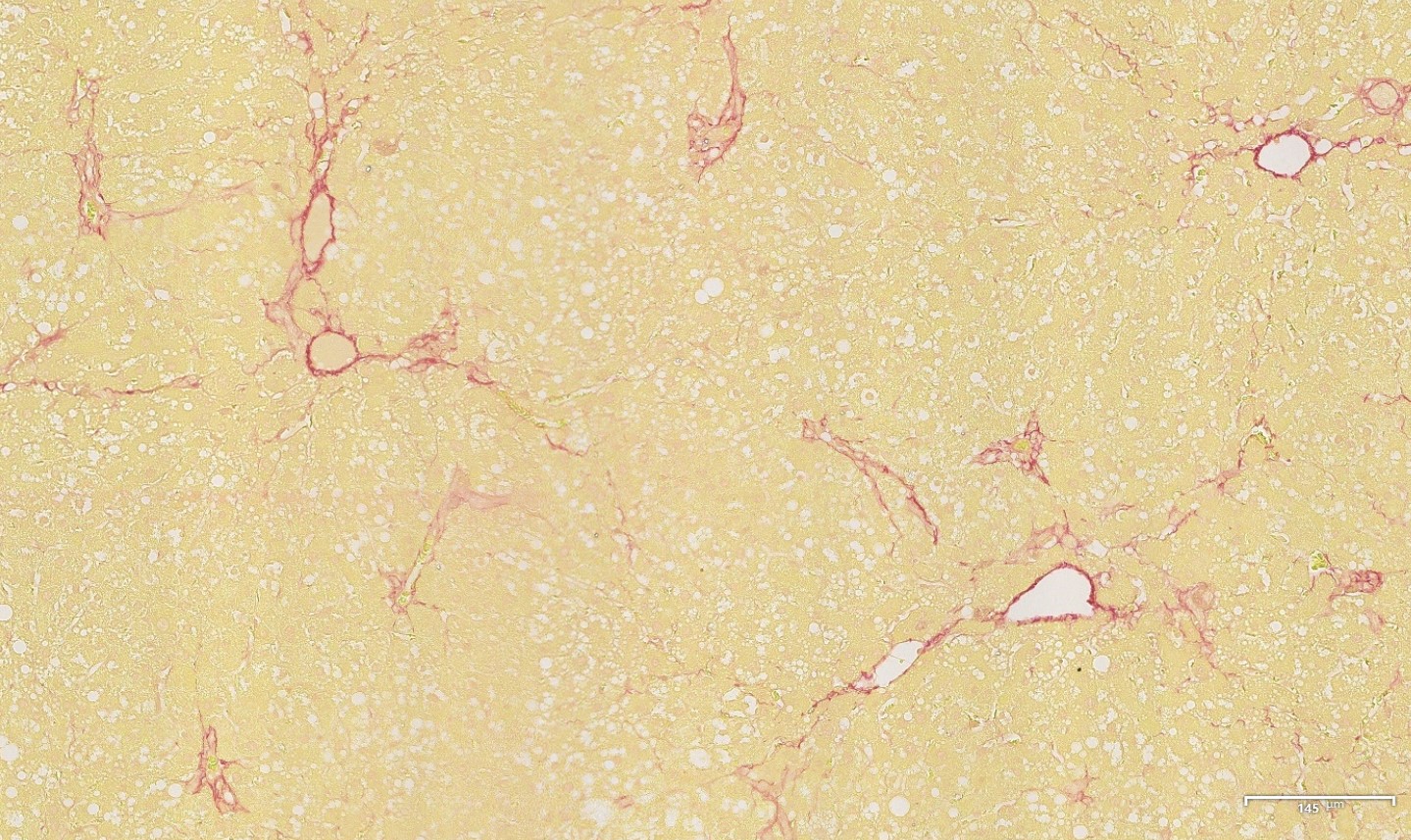

Supplement: Supplementary file 1 [file DataSheet3.ZIP › the original source data of Figures 1-4/Fig. 2/Fig. 2C (Rat-CCl4)/SR/SORA.jpg]

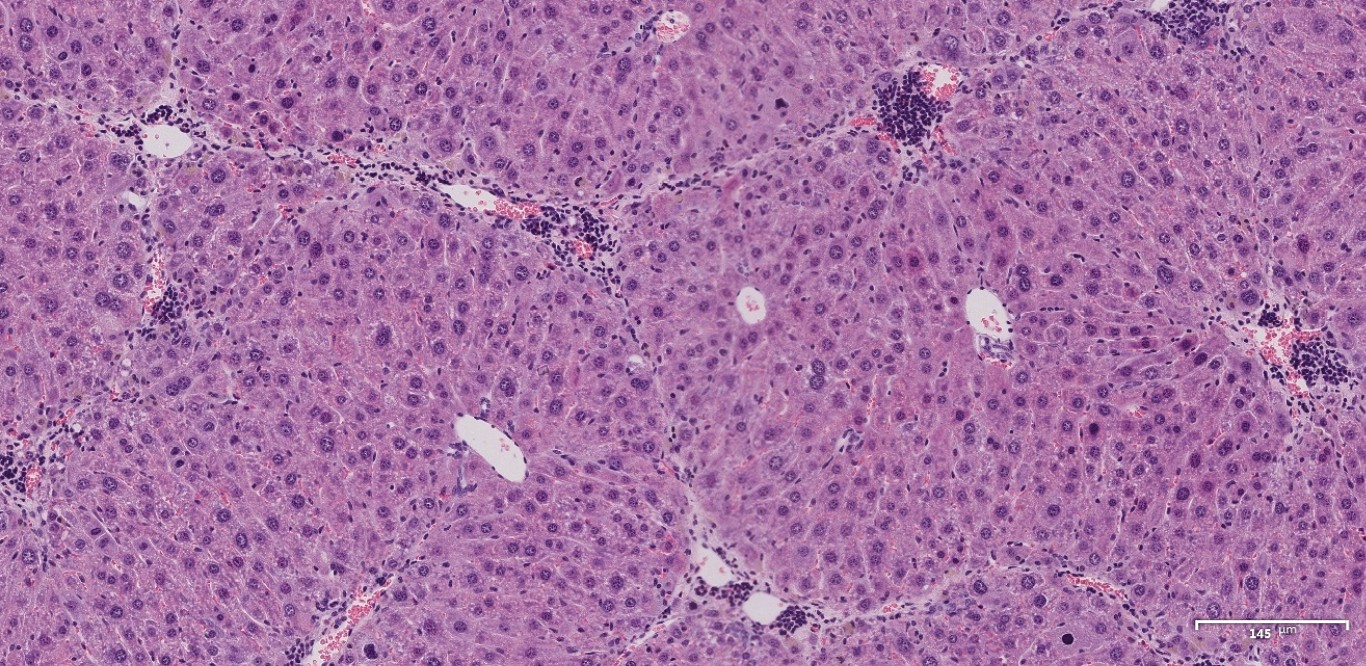

Supplement: Supplementary file 1 [file DataSheet3.ZIP › the original source data of Figures 1-4/Fig. 3/Fig. 3A (Mice-CCl4)/H&E/CCl4.jpg]

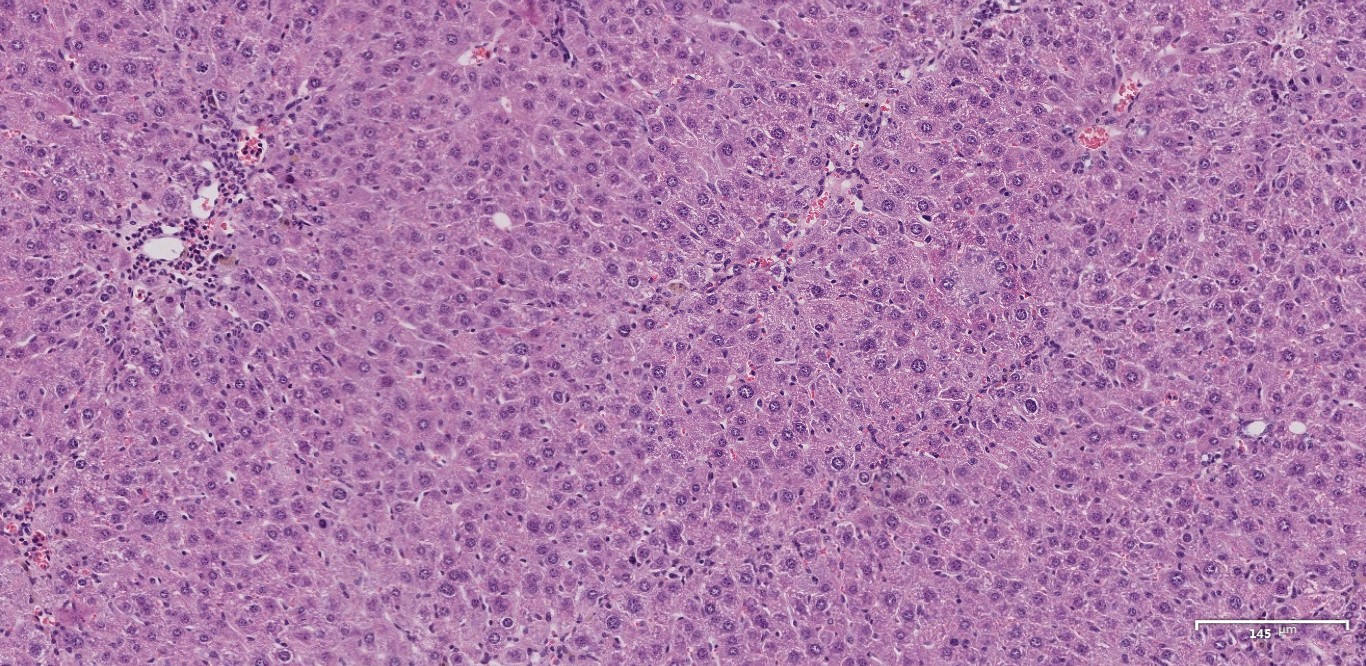

Supplement: Supplementary file 1 [file DataSheet3.ZIP › the original source data of Figures 1-4/Fig. 3/Fig. 3A (Mice-CCl4)/H&E/JY5.jpg]

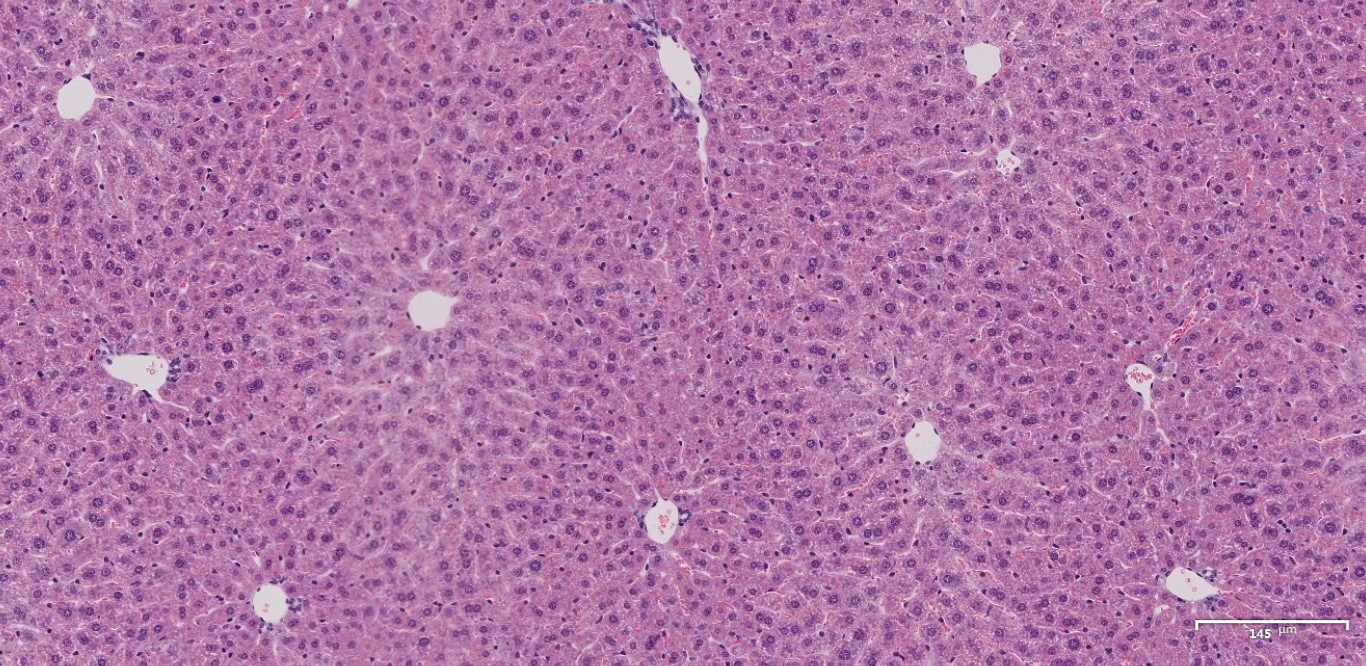

Supplement: Supplementary file 1 [file DataSheet3.ZIP › the original source data of Figures 1-4/Fig. 3/Fig. 3A (Mice-CCl4)/H&E/Oil.jpg]

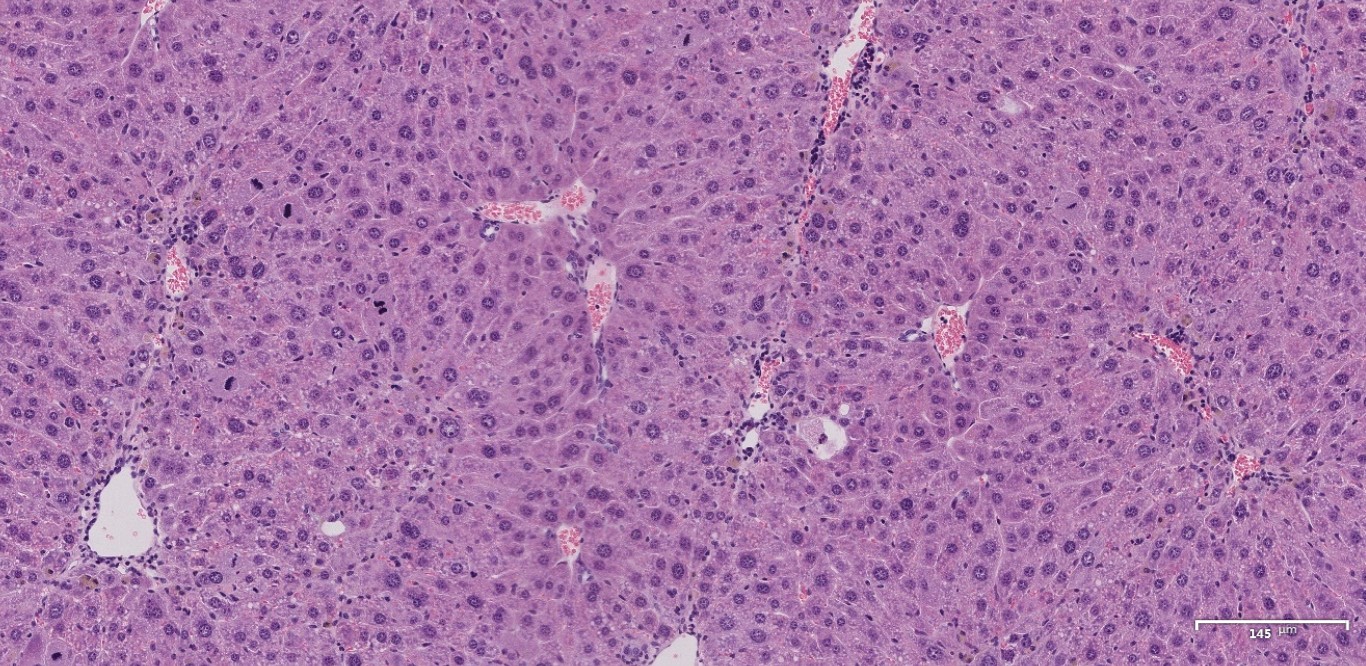

Supplement: Supplementary file 1 [file DataSheet3.ZIP › the original source data of Figures 1-4/Fig. 3/Fig. 3A (Mice-CCl4)/H&E/SORA.jpg]

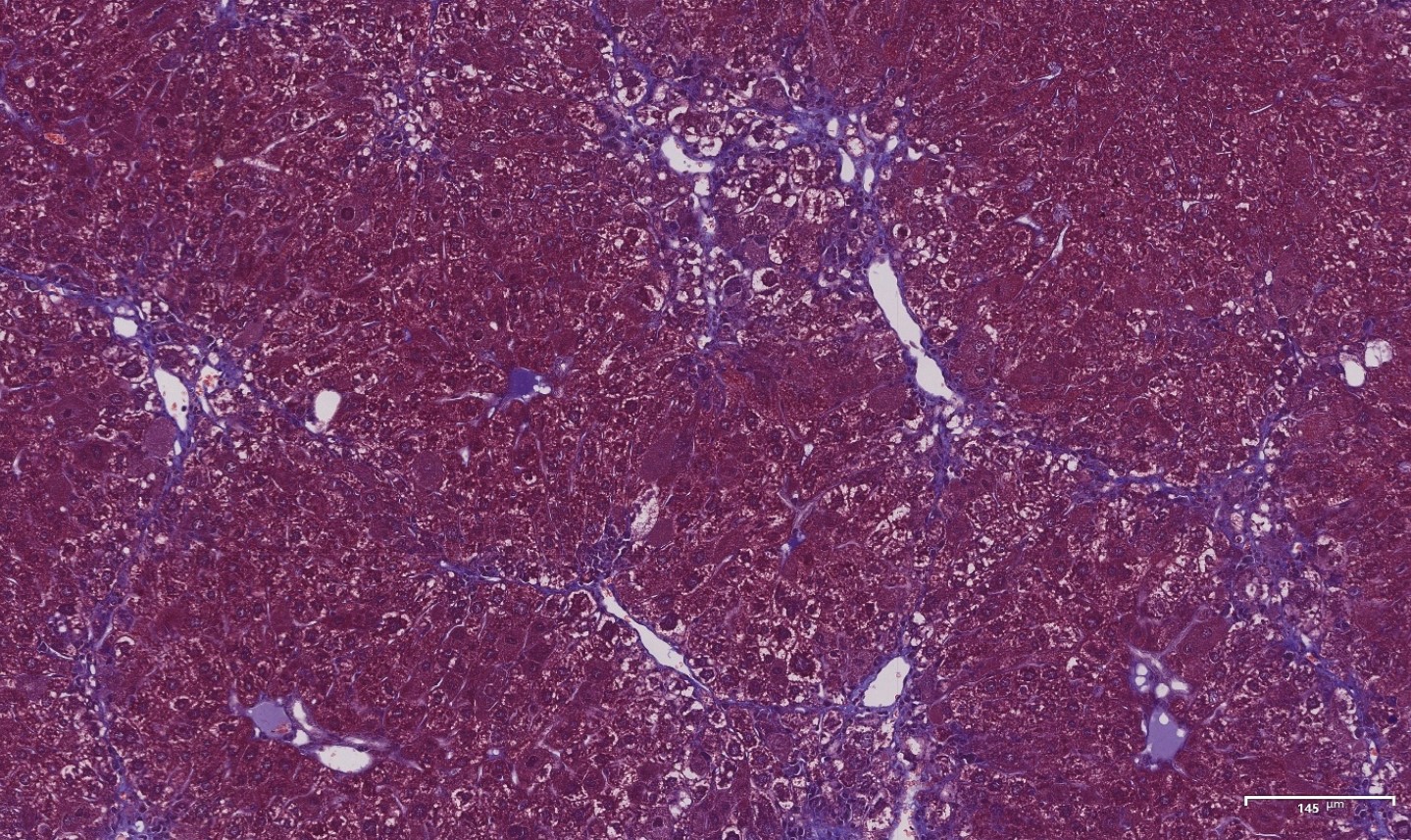

Supplement: Supplementary file 1 [file DataSheet3.ZIP › the original source data of Figures 1-4/Fig. 3/Fig. 3A (Mice-CCl4)/Masson/CCl4.jpg]

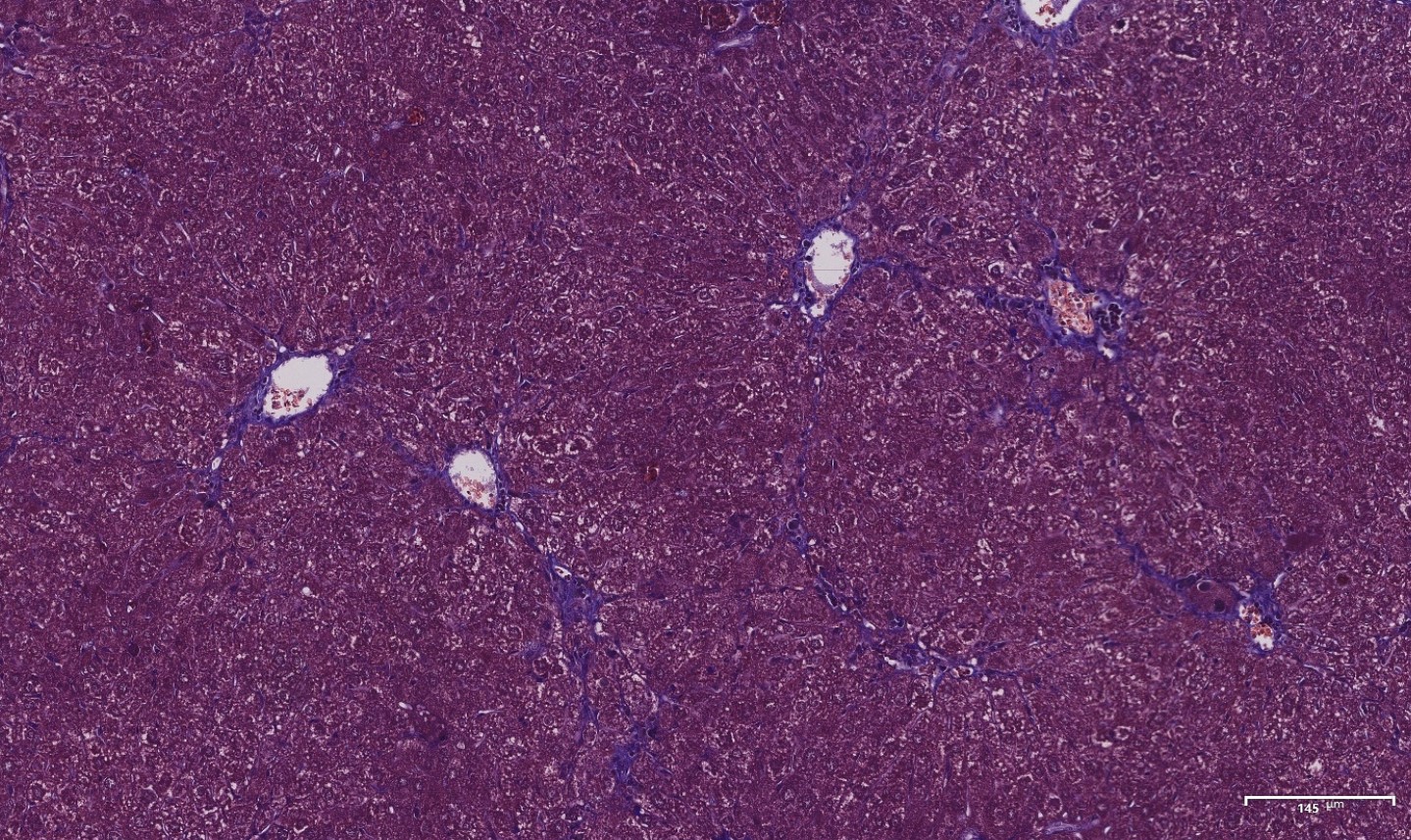

Supplement: Supplementary file 1 [file DataSheet3.ZIP › the original source data of Figures 1-4/Fig. 3/Fig. 3A (Mice-CCl4)/Masson/JY5.jpg]

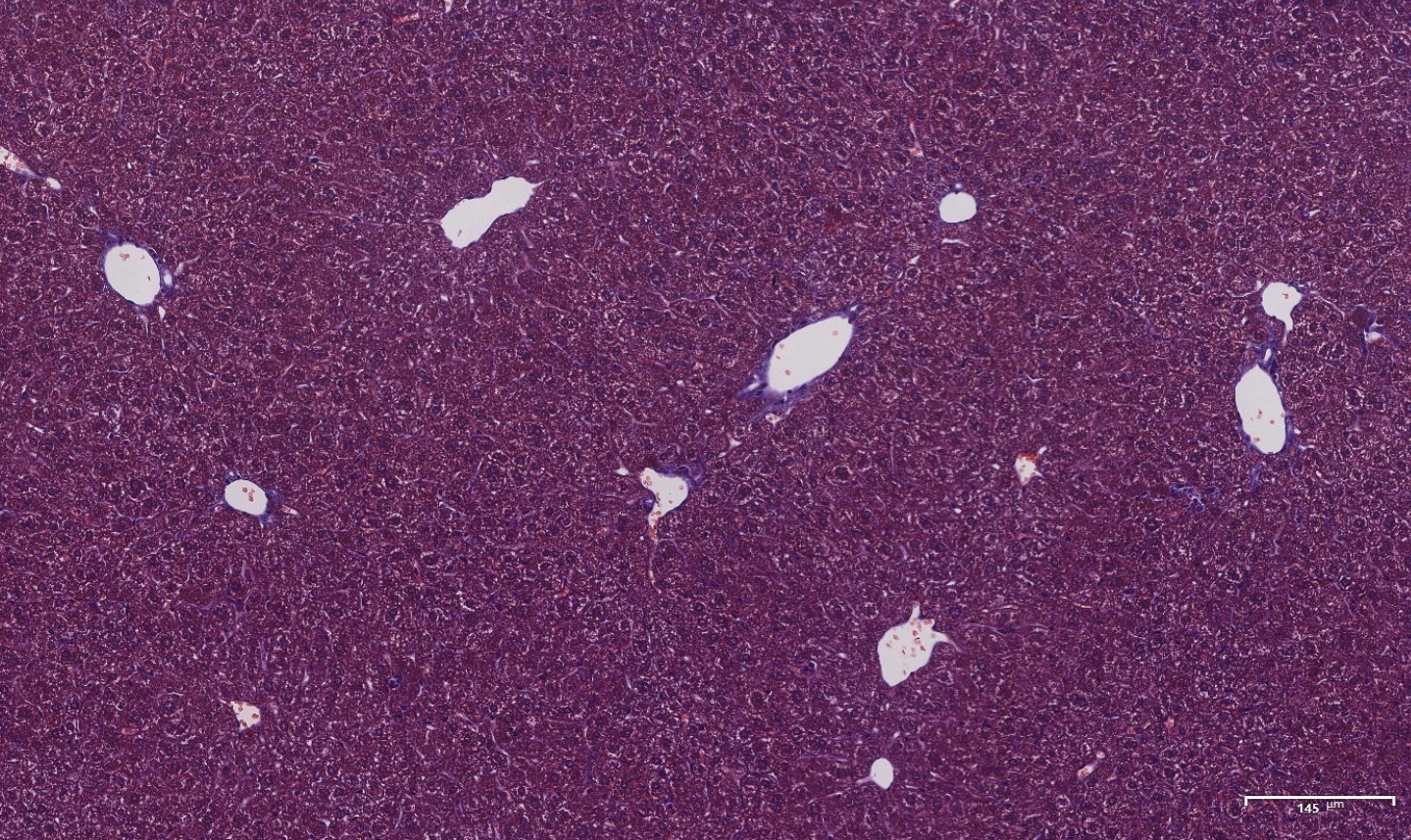

Supplement: Supplementary file 1 [file DataSheet3.ZIP › the original source data of Figures 1-4/Fig. 3/Fig. 3A (Mice-CCl4)/Masson/Oil.jpg]

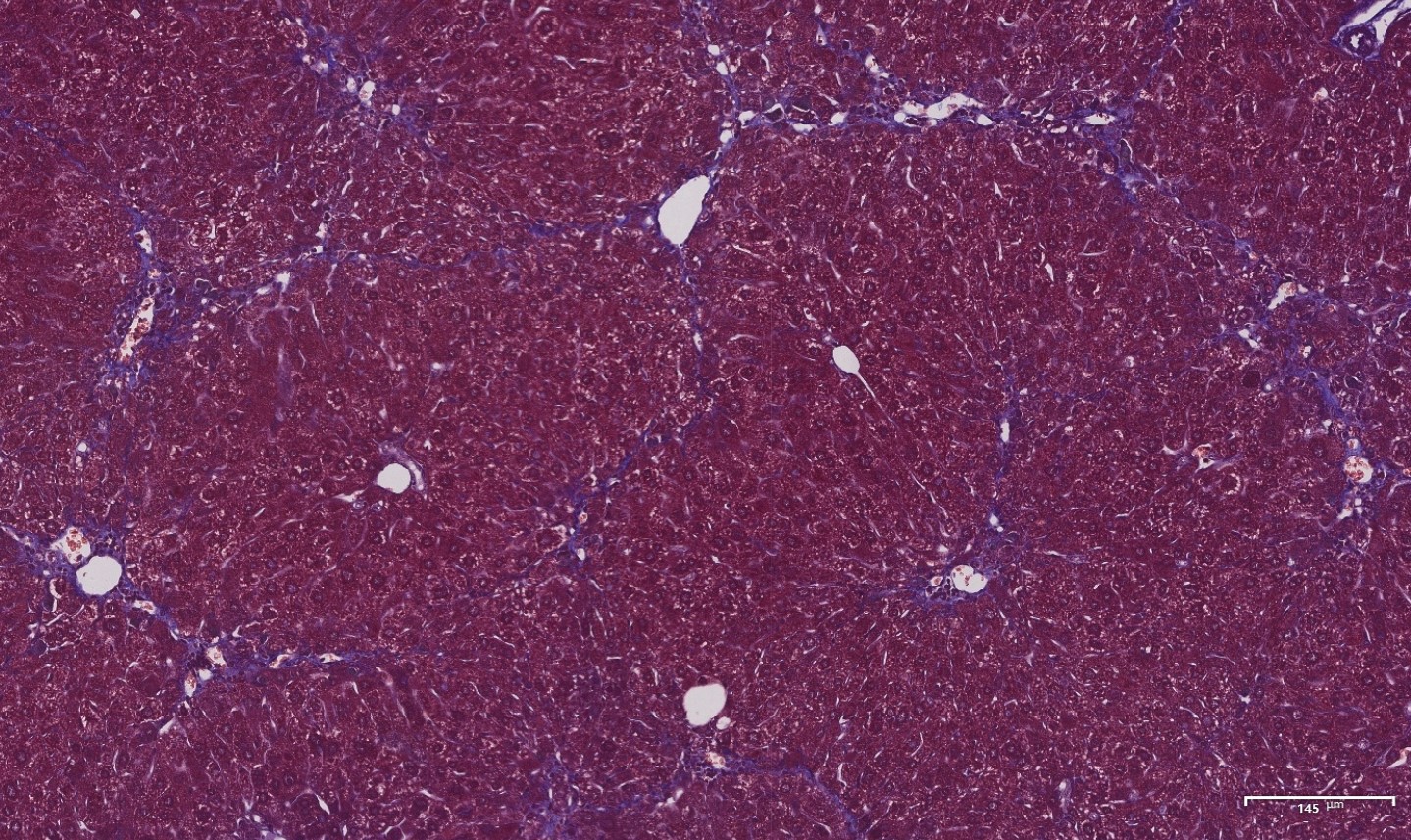

Supplement: Supplementary file 1 [file DataSheet3.ZIP › the original source data of Figures 1-4/Fig. 3/Fig. 3A (Mice-CCl4)/Masson/SORA.jpg]

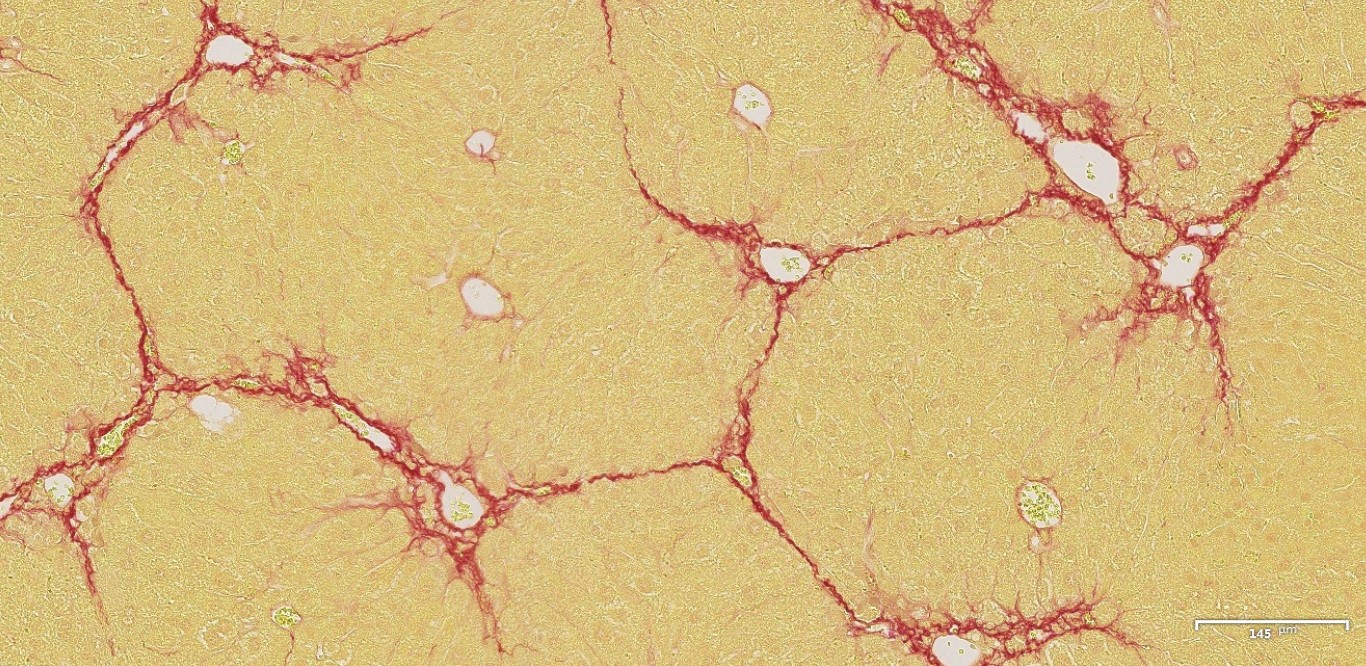

Supplement: Supplementary file 1 [file DataSheet3.ZIP › the original source data of Figures 1-4/Fig. 3/Fig. 3A (Mice-CCl4)/SR/CCl4.jpg]

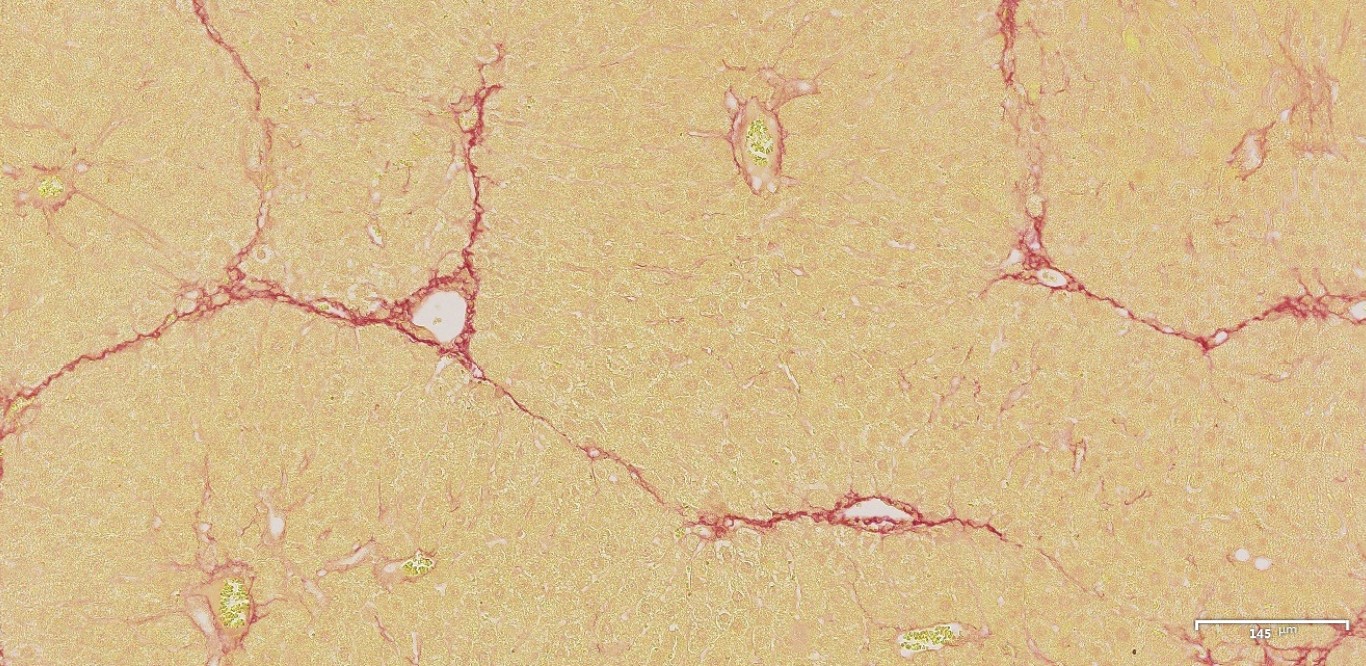

Supplement: Supplementary file 1 [file DataSheet3.ZIP › the original source data of Figures 1-4/Fig. 3/Fig. 3A (Mice-CCl4)/SR/JY5.jpg]

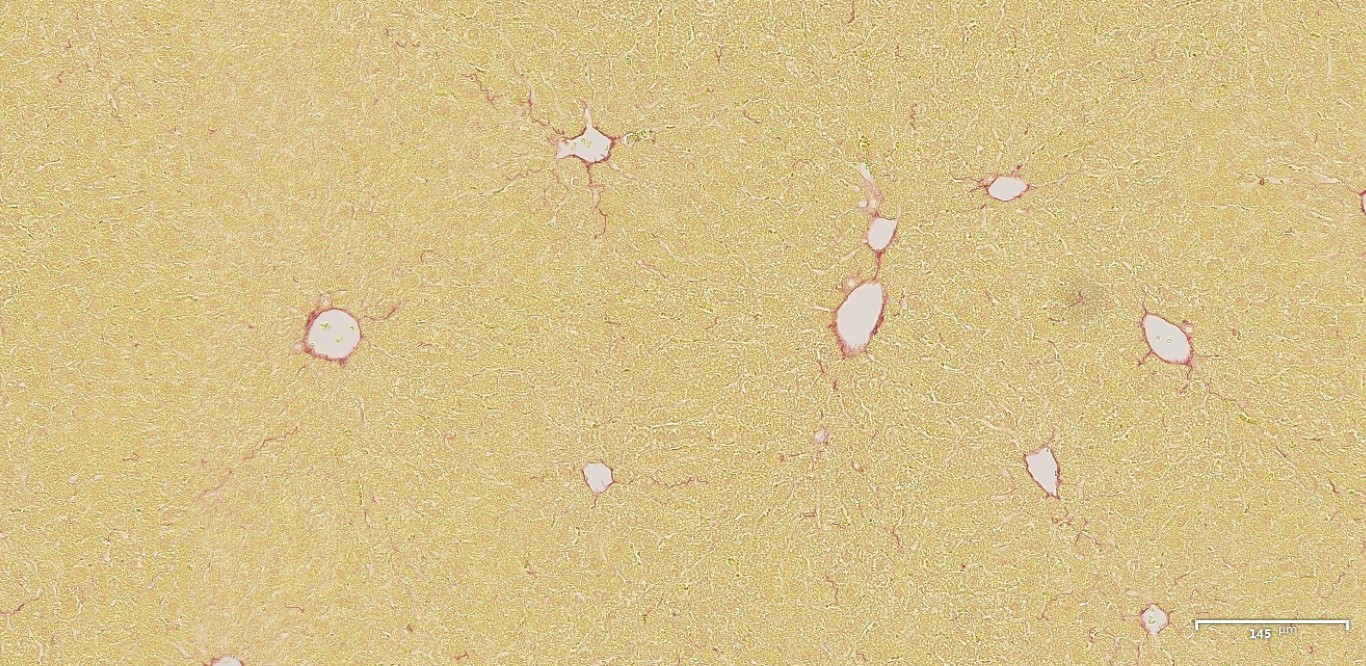

Supplement: Supplementary file 1 [file DataSheet3.ZIP › the original source data of Figures 1-4/Fig. 3/Fig. 3A (Mice-CCl4)/SR/Oil.jpg]

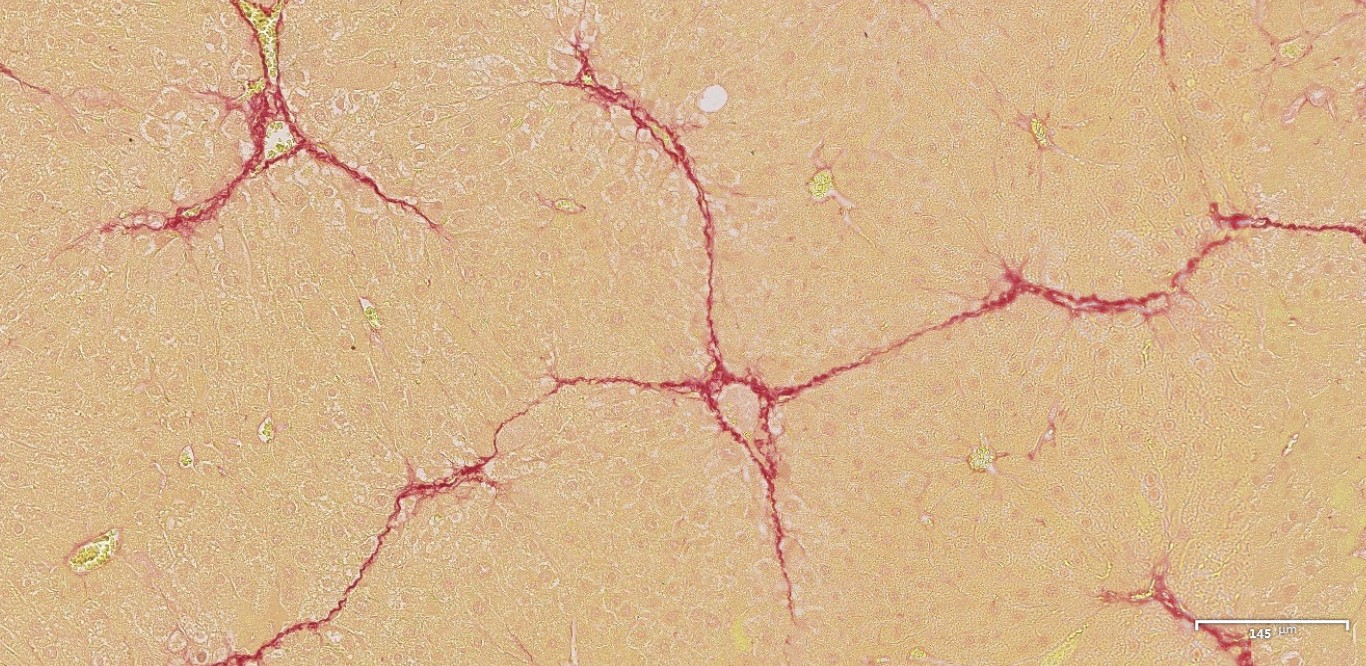

Supplement: Supplementary file 1 [file DataSheet3.ZIP › the original source data of Figures 1-4/Fig. 3/Fig. 3A (Mice-CCl4)/SR/SORA.jpg]

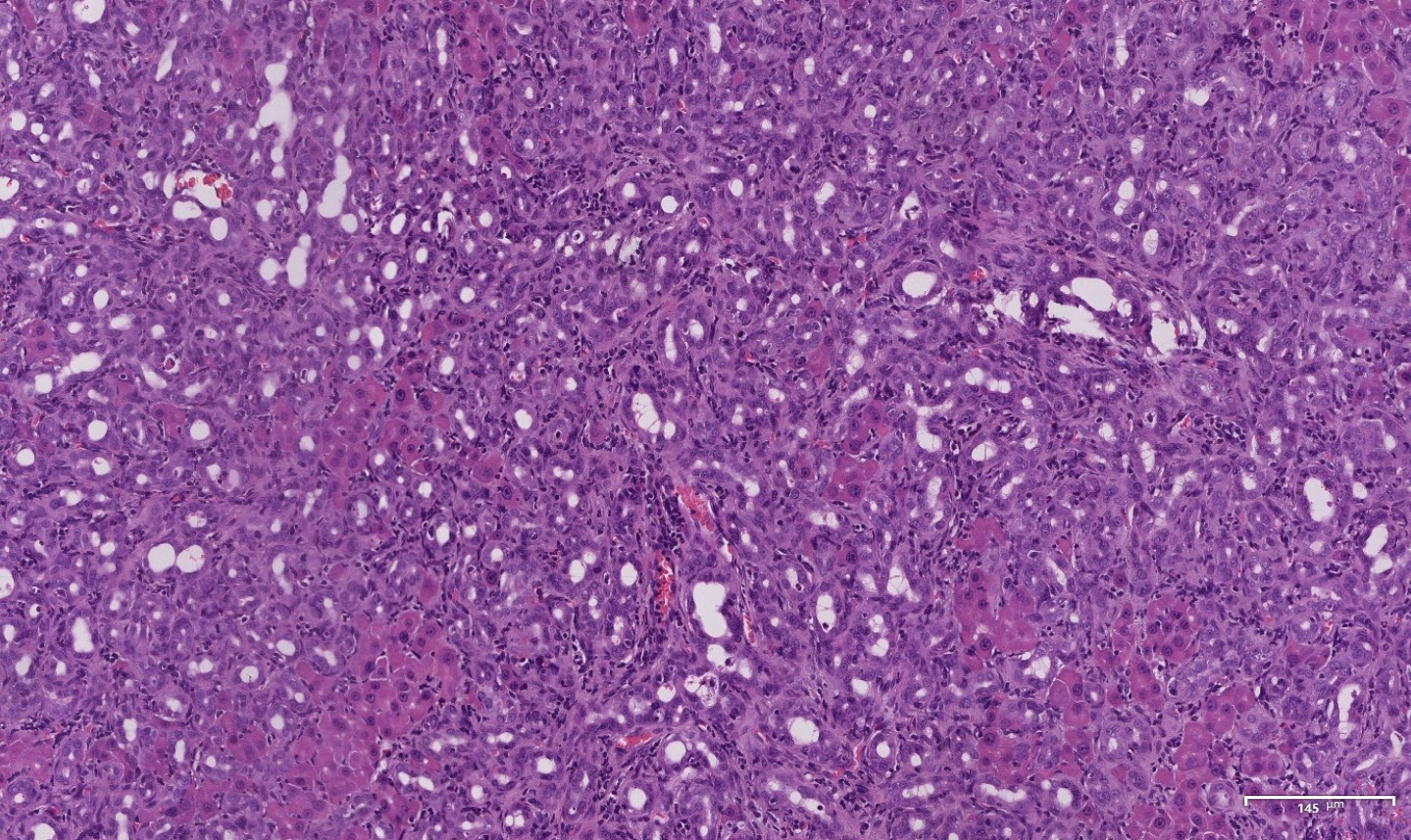

Supplement: Supplementary file 1 [file DataSheet3.ZIP › the original source data of Figures 1-4/Fig. 4/Fig. 4G (Rat-BDL)/H&E/BDL.jpg]

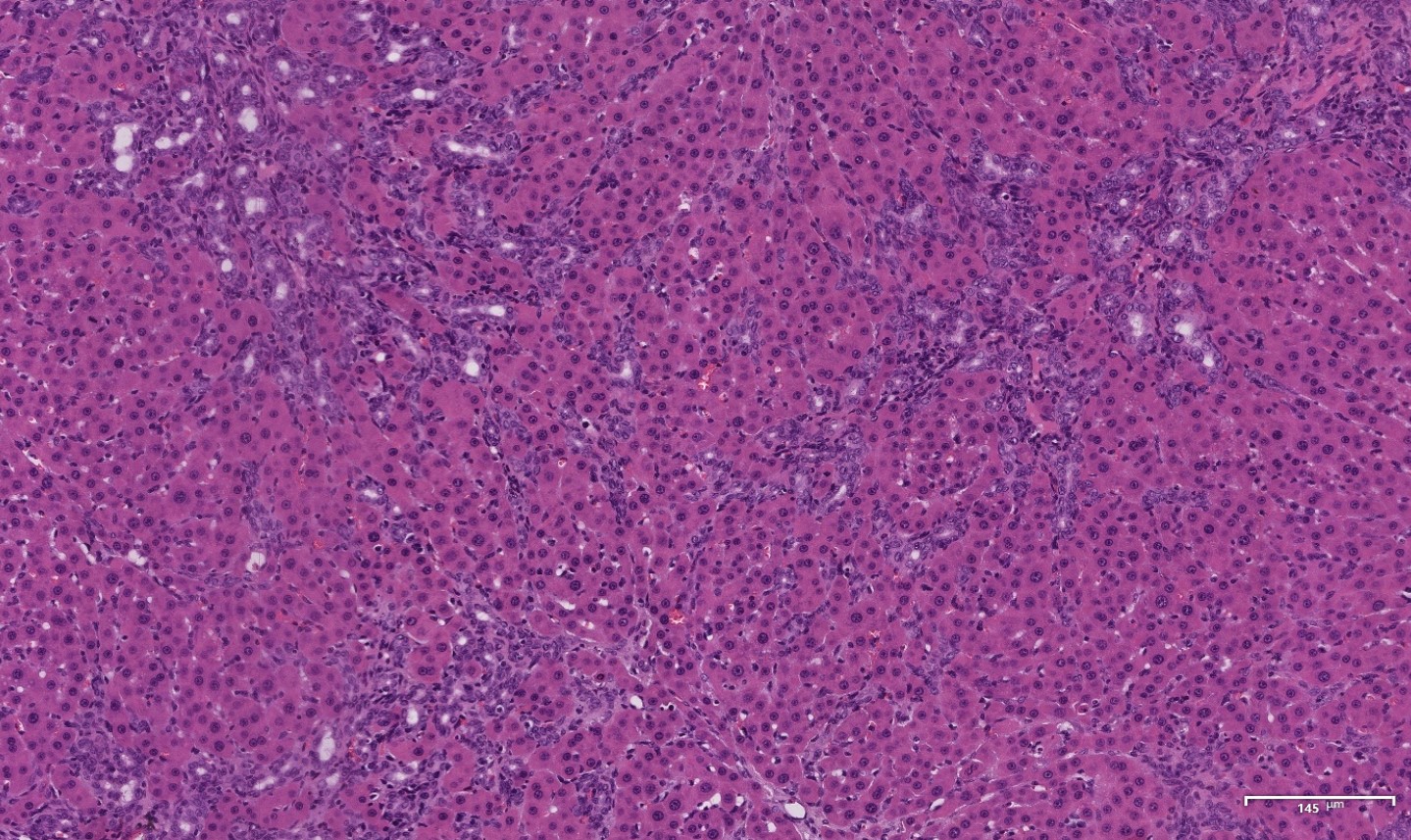

Supplement: Supplementary file 1 [file DataSheet3.ZIP › the original source data of Figures 1-4/Fig. 4/Fig. 4G (Rat-BDL)/H&E/DAPT.jpg]

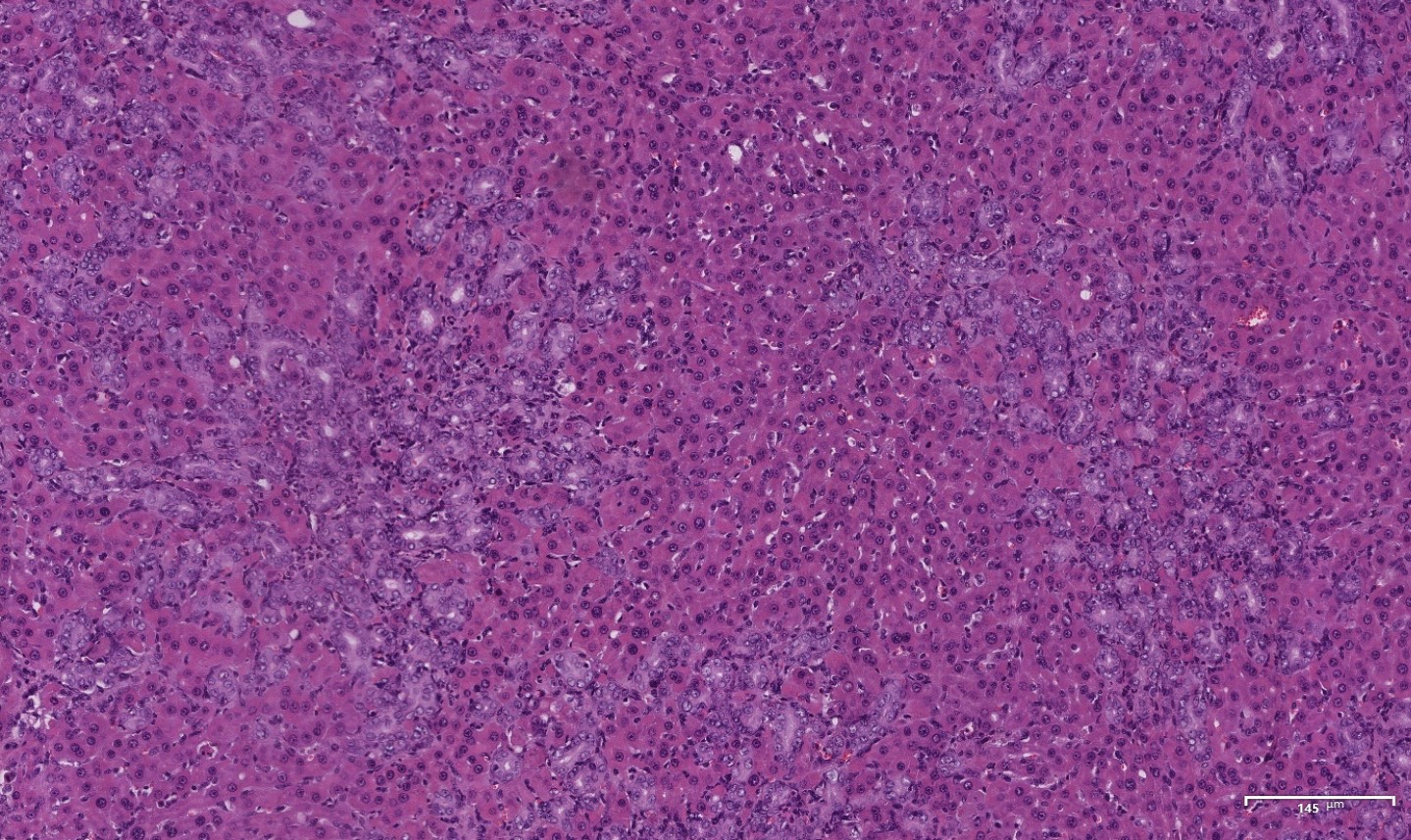

Supplement: Supplementary file 1 [file DataSheet3.ZIP › the original source data of Figures 1-4/Fig. 4/Fig. 4G (Rat-BDL)/H&E/JY5.jpg]

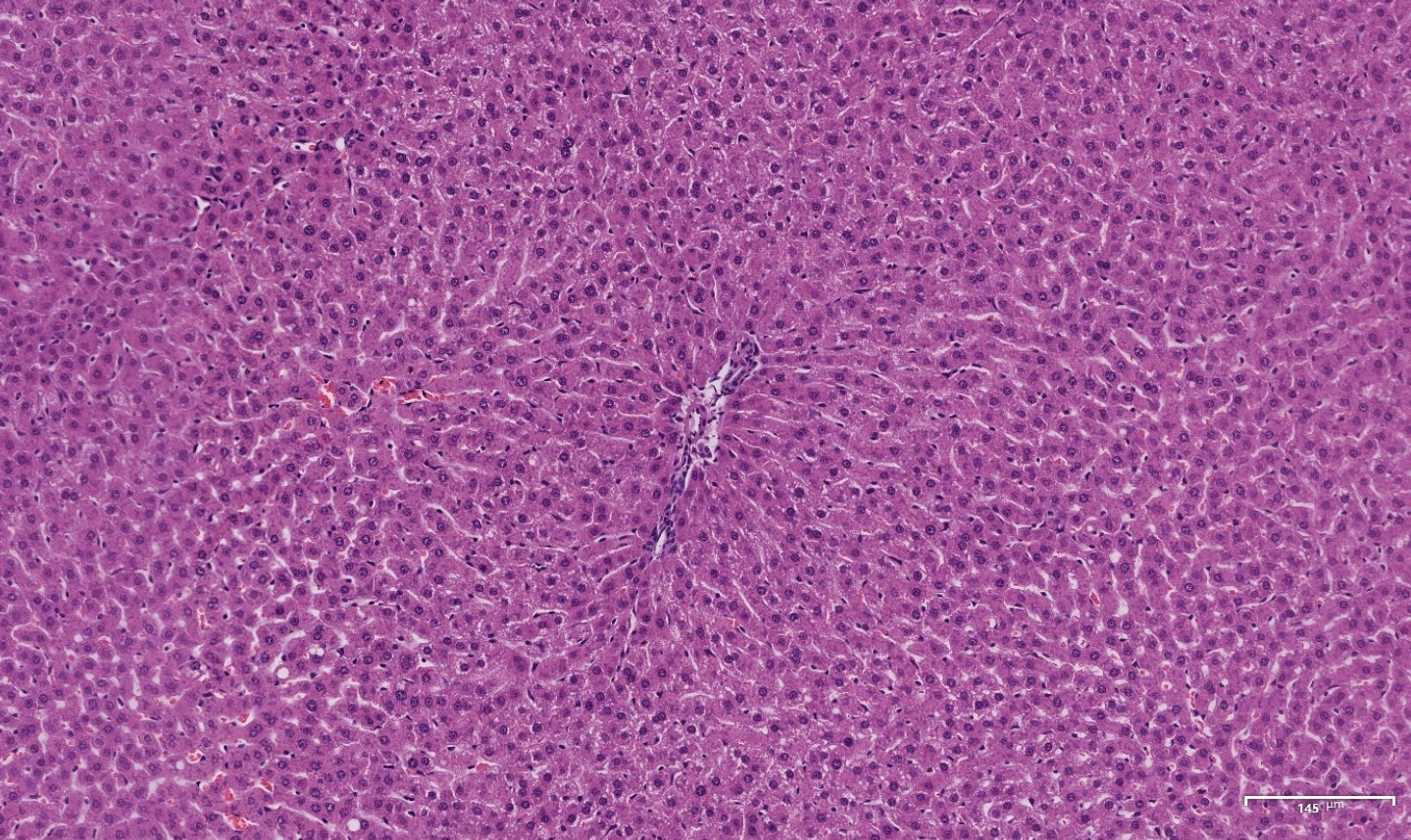

Supplement: Supplementary file 1 [file DataSheet3.ZIP › the original source data of Figures 1-4/Fig. 4/Fig. 4G (Rat-BDL)/H&E/Sham.jpg]

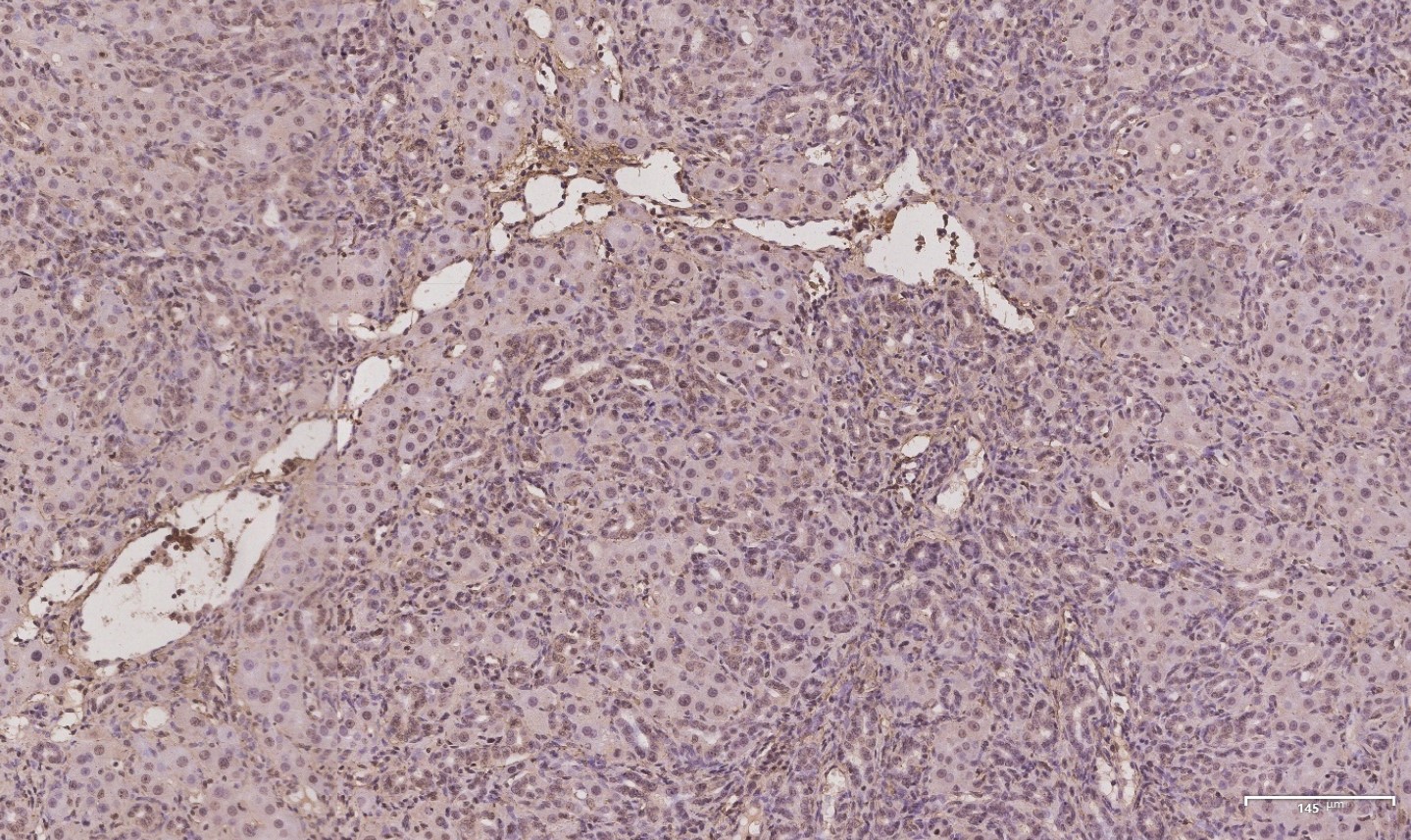

Supplement: Supplementary file 1 [file DataSheet3.ZIP › the original source data of Figures 1-4/Fig. 4/Fig. 4G (Rat-BDL)/IHC-Col-I/BDL.jpg]

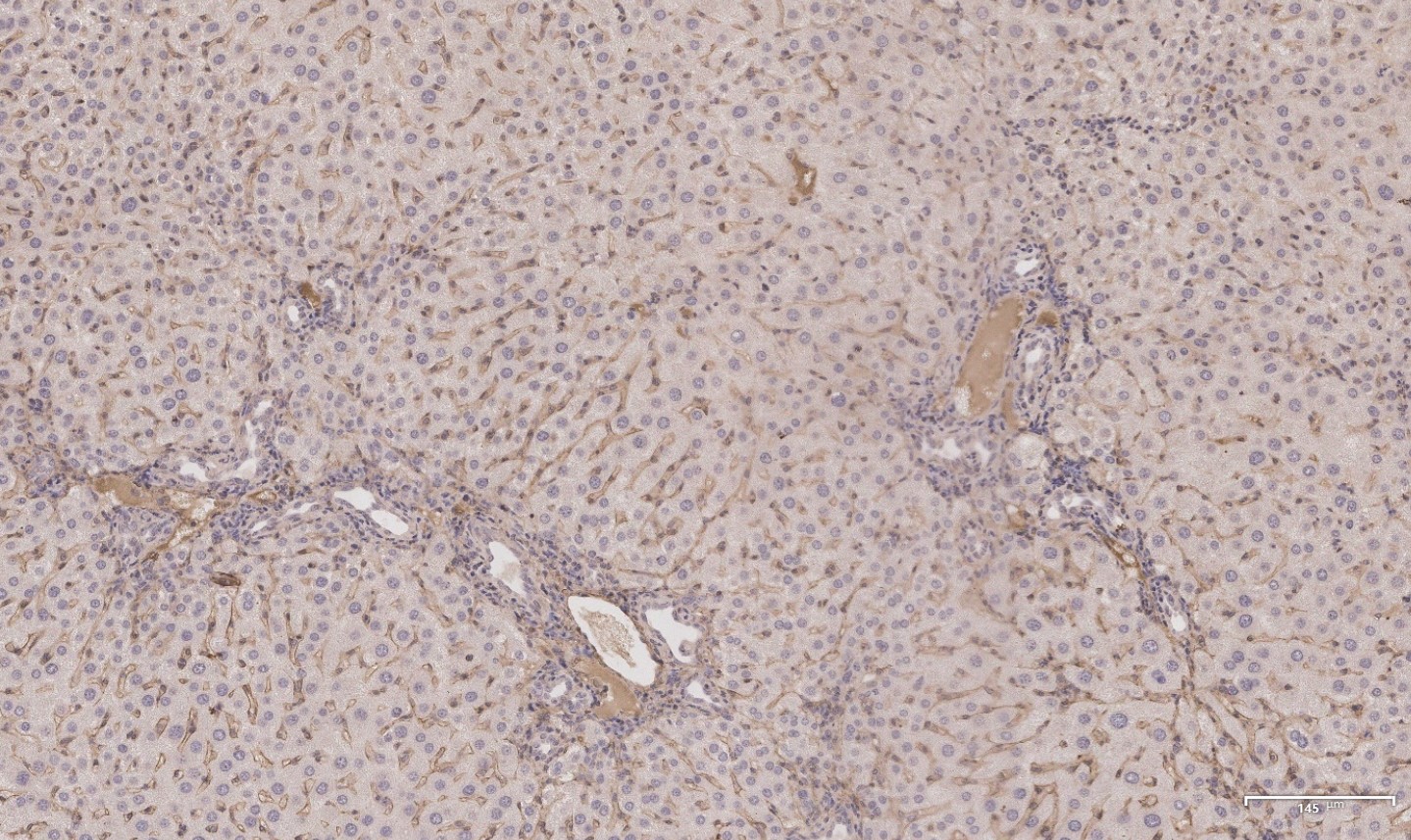

Supplement: Supplementary file 1 [file DataSheet3.ZIP › the original source data of Figures 1-4/Fig. 4/Fig. 4G (Rat-BDL)/IHC-Col-I/DAPT.jpg]

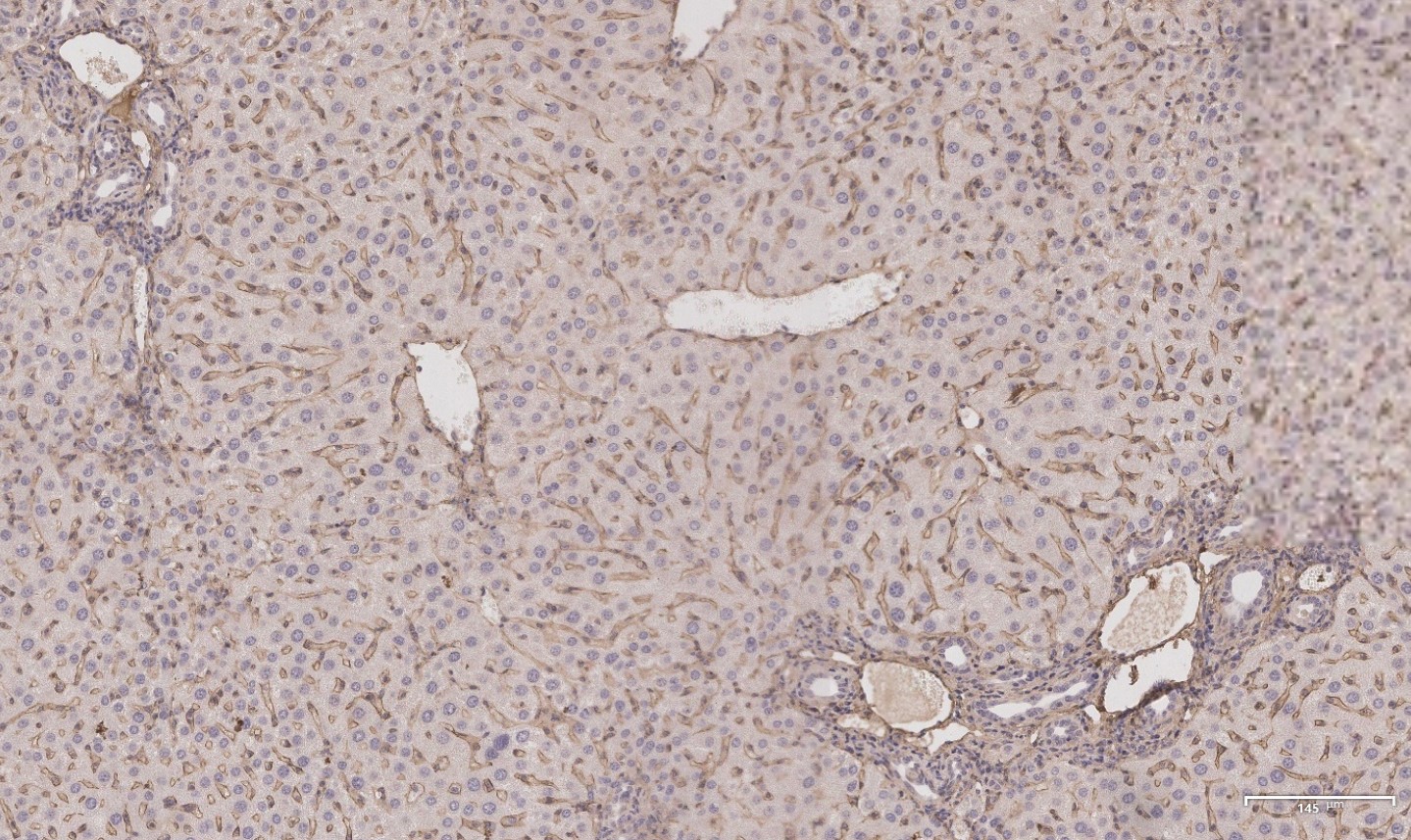

Supplement: Supplementary file 1 [file DataSheet3.ZIP › the original source data of Figures 1-4/Fig. 4/Fig. 4G (Rat-BDL)/IHC-Col-I/JY5.jpg]

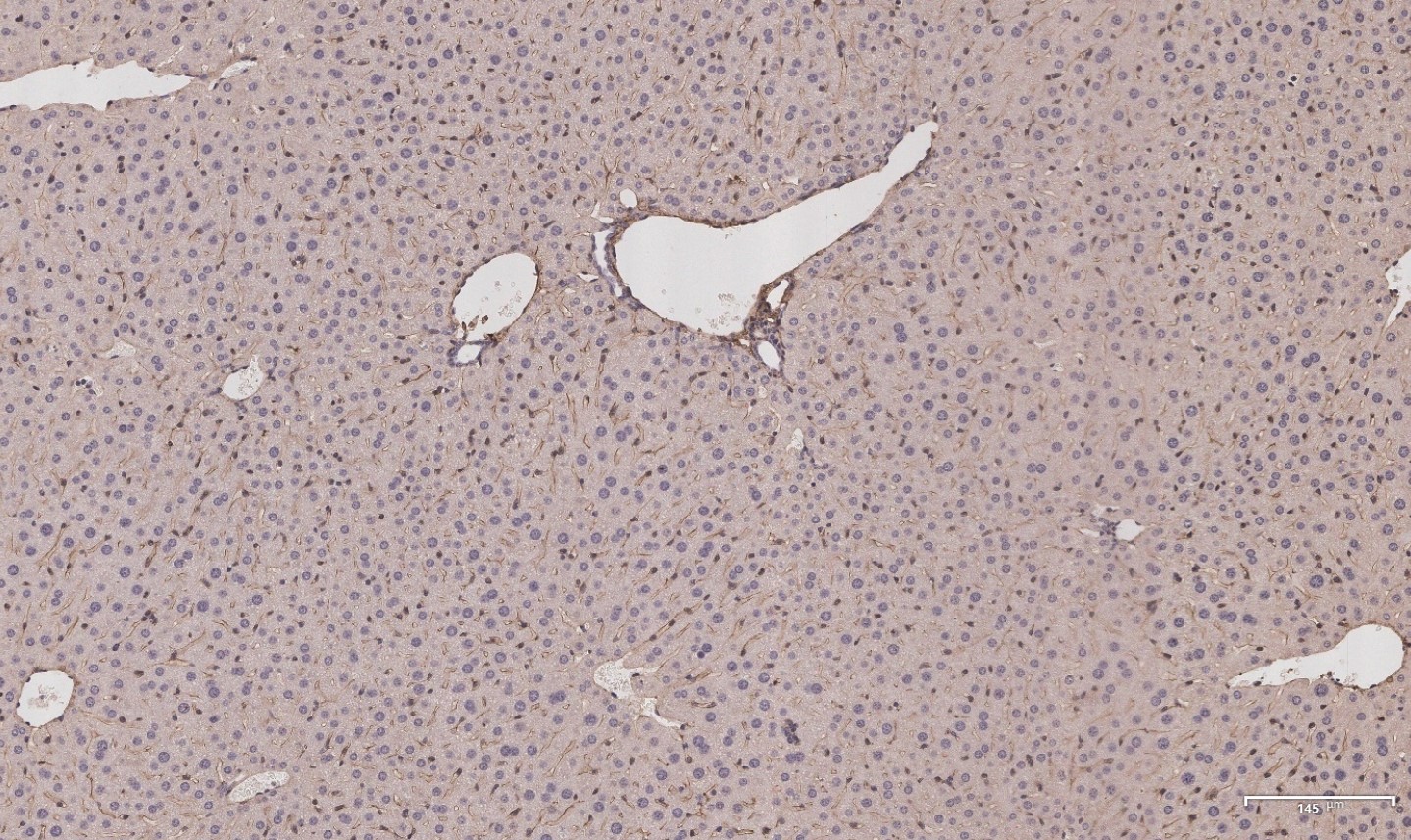

Supplement: Supplementary file 1 [file DataSheet3.ZIP › the original source data of Figures 1-4/Fig. 4/Fig. 4G (Rat-BDL)/IHC-Col-I/Sham.jpg]

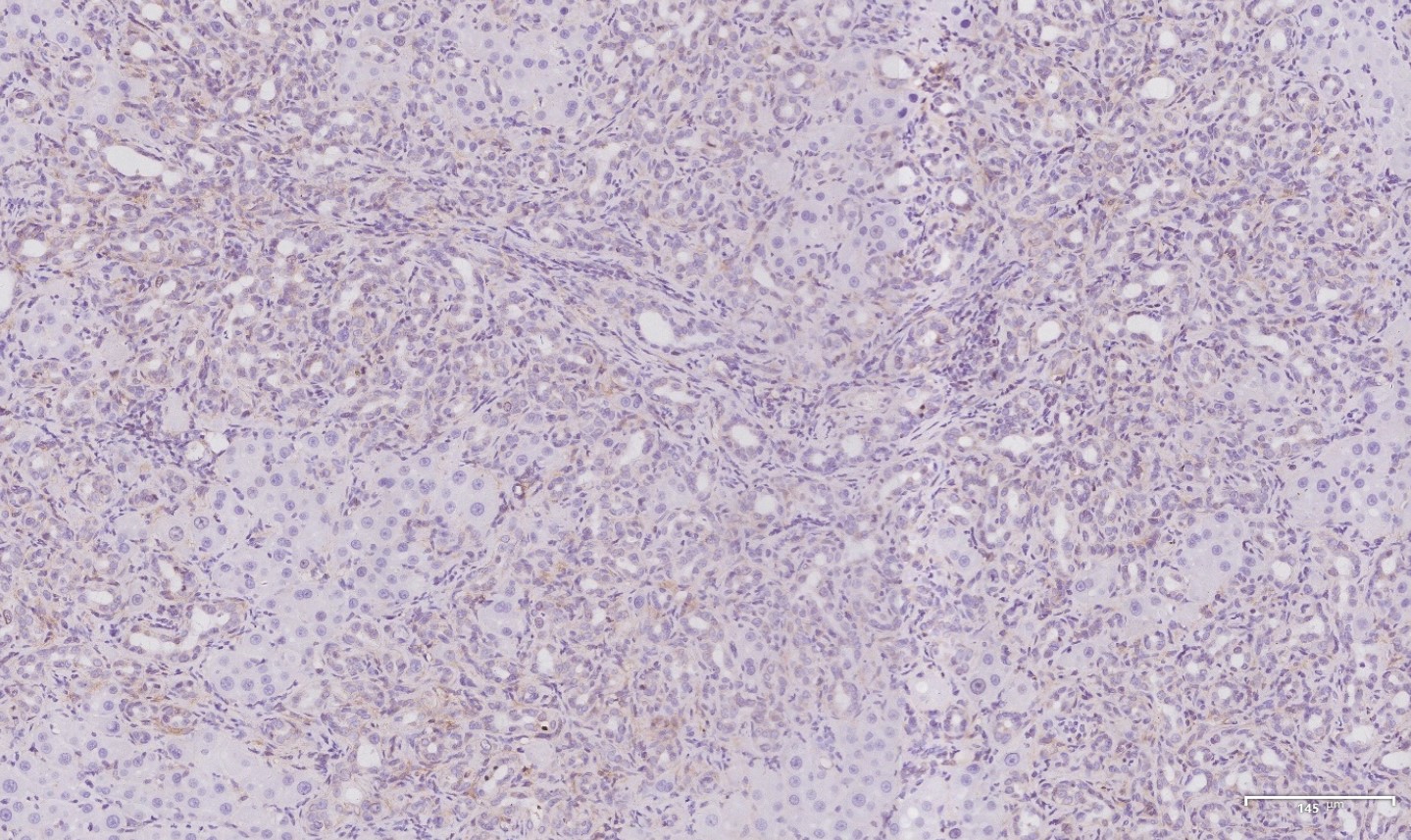

Supplement: Supplementary file 1 [file DataSheet3.ZIP › the original source data of Figures 1-4/Fig. 4/Fig. 4G (Rat-BDL)/IHC-Col-IV/BDL.jpg]

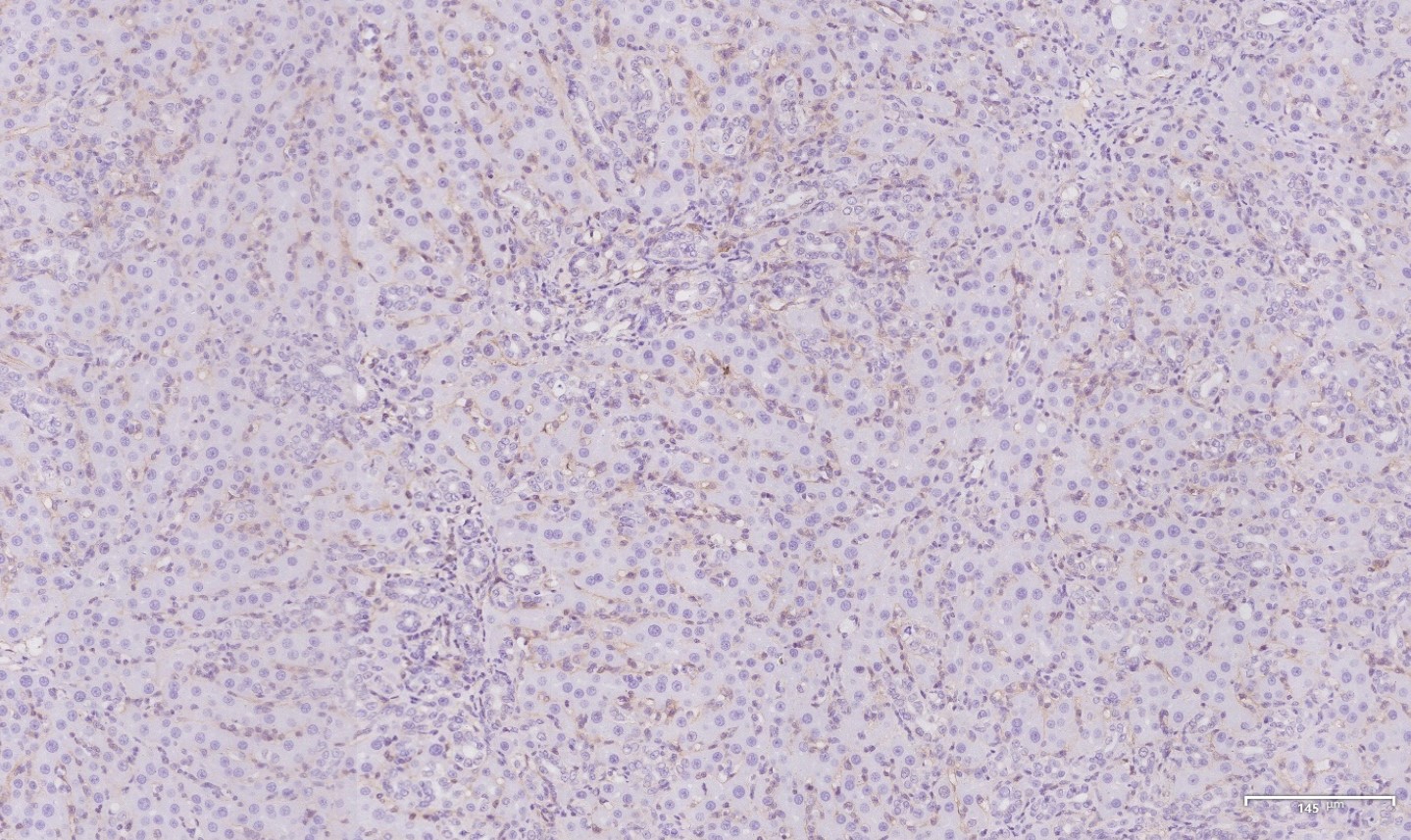

Supplement: Supplementary file 1 [file DataSheet3.ZIP › the original source data of Figures 1-4/Fig. 4/Fig. 4G (Rat-BDL)/IHC-Col-IV/DAPT.jpg]

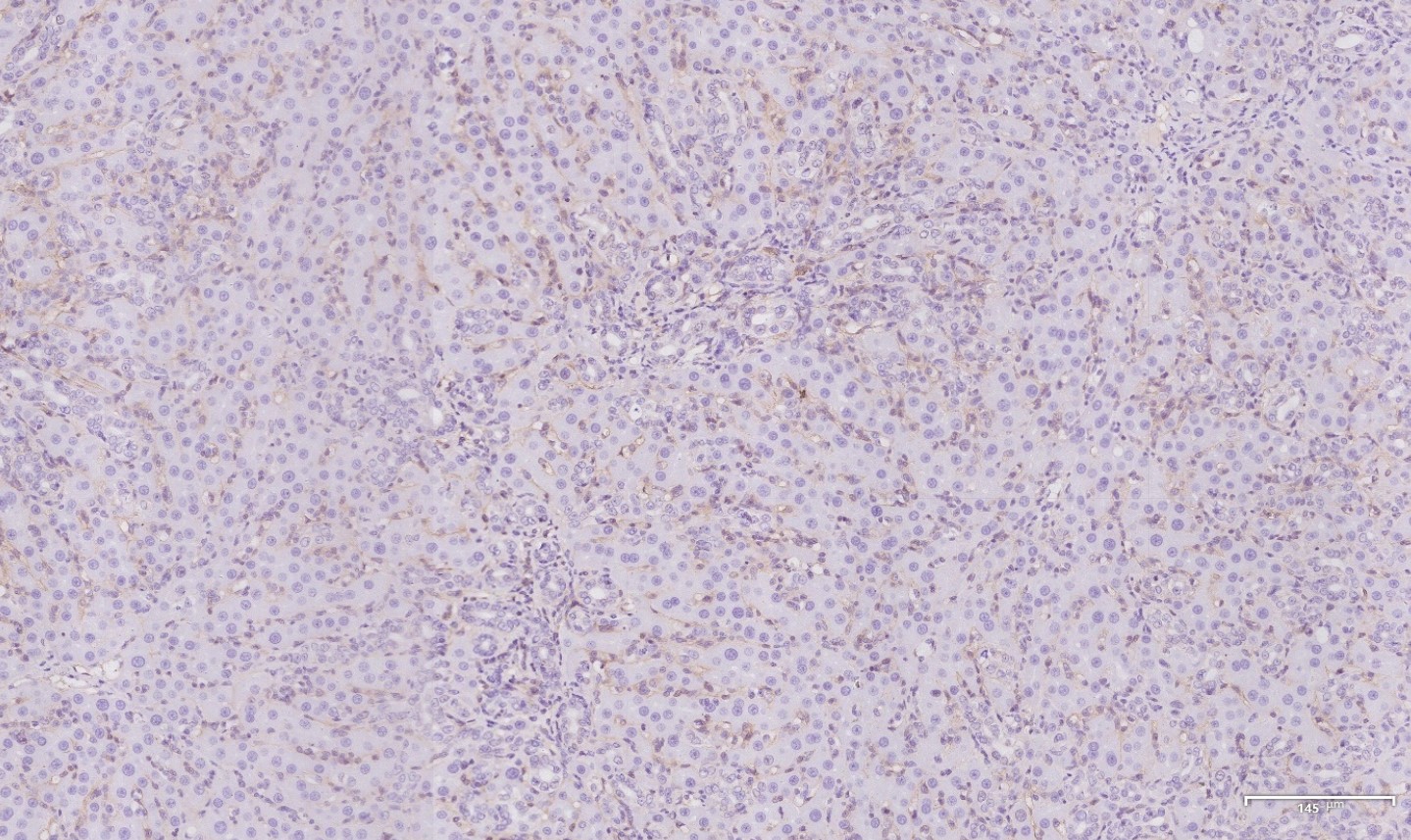

Supplement: Supplementary file 1 [file DataSheet3.ZIP › the original source data of Figures 1-4/Fig. 4/Fig. 4G (Rat-BDL)/IHC-Col-IV/JY5.jpg]

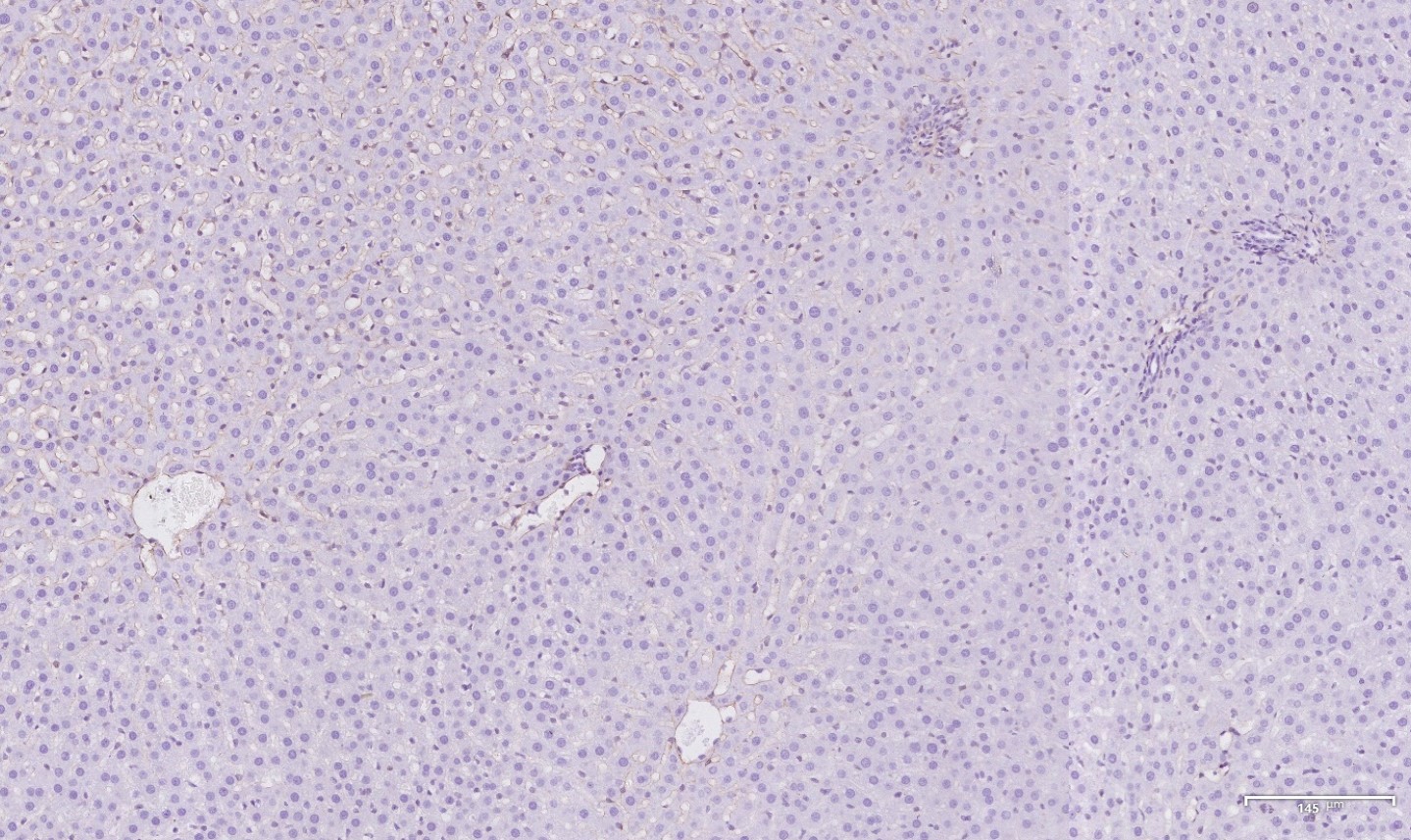

Supplement: Supplementary file 1 [file DataSheet3.ZIP › the original source data of Figures 1-4/Fig. 4/Fig. 4G (Rat-BDL)/IHC-Col-IV/Sham.jpg]

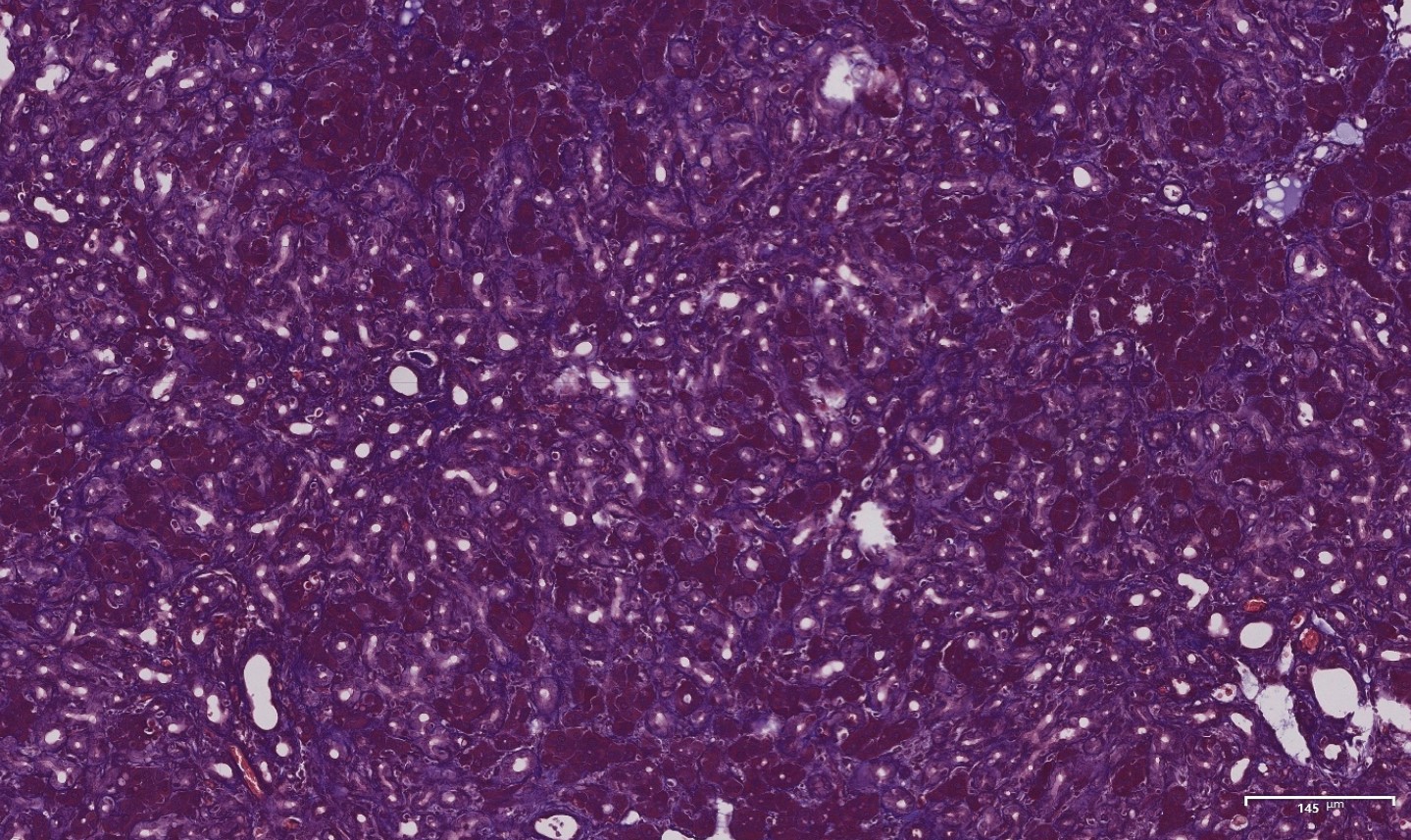

Supplement: Supplementary file 1 [file DataSheet3.ZIP › the original source data of Figures 1-4/Fig. 4/Fig. 4G (Rat-BDL)/Masson/BDL.jpg]

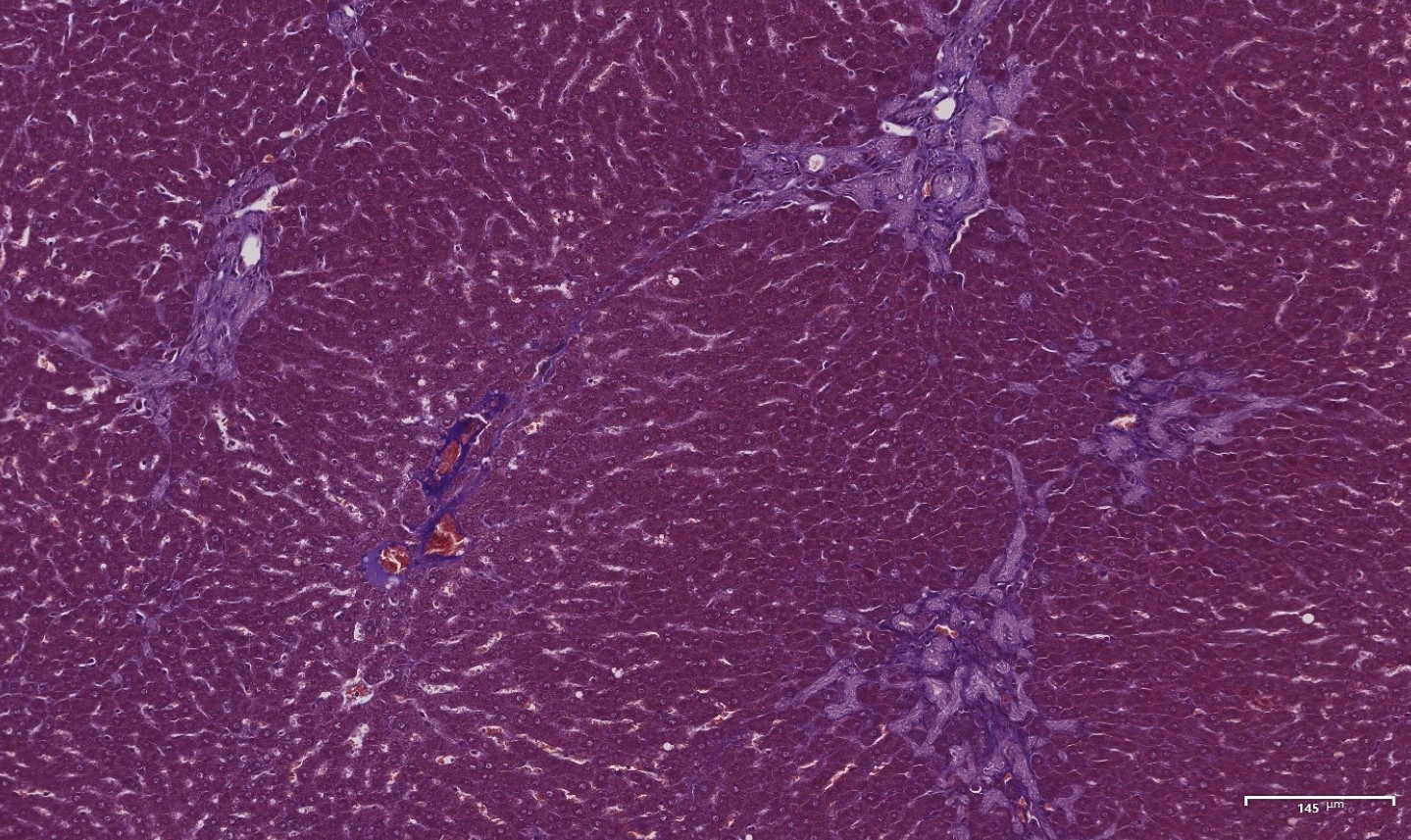

Supplement: Supplementary file 1 [file DataSheet3.ZIP › the original source data of Figures 1-4/Fig. 4/Fig. 4G (Rat-BDL)/Masson/DAPT.jpg]

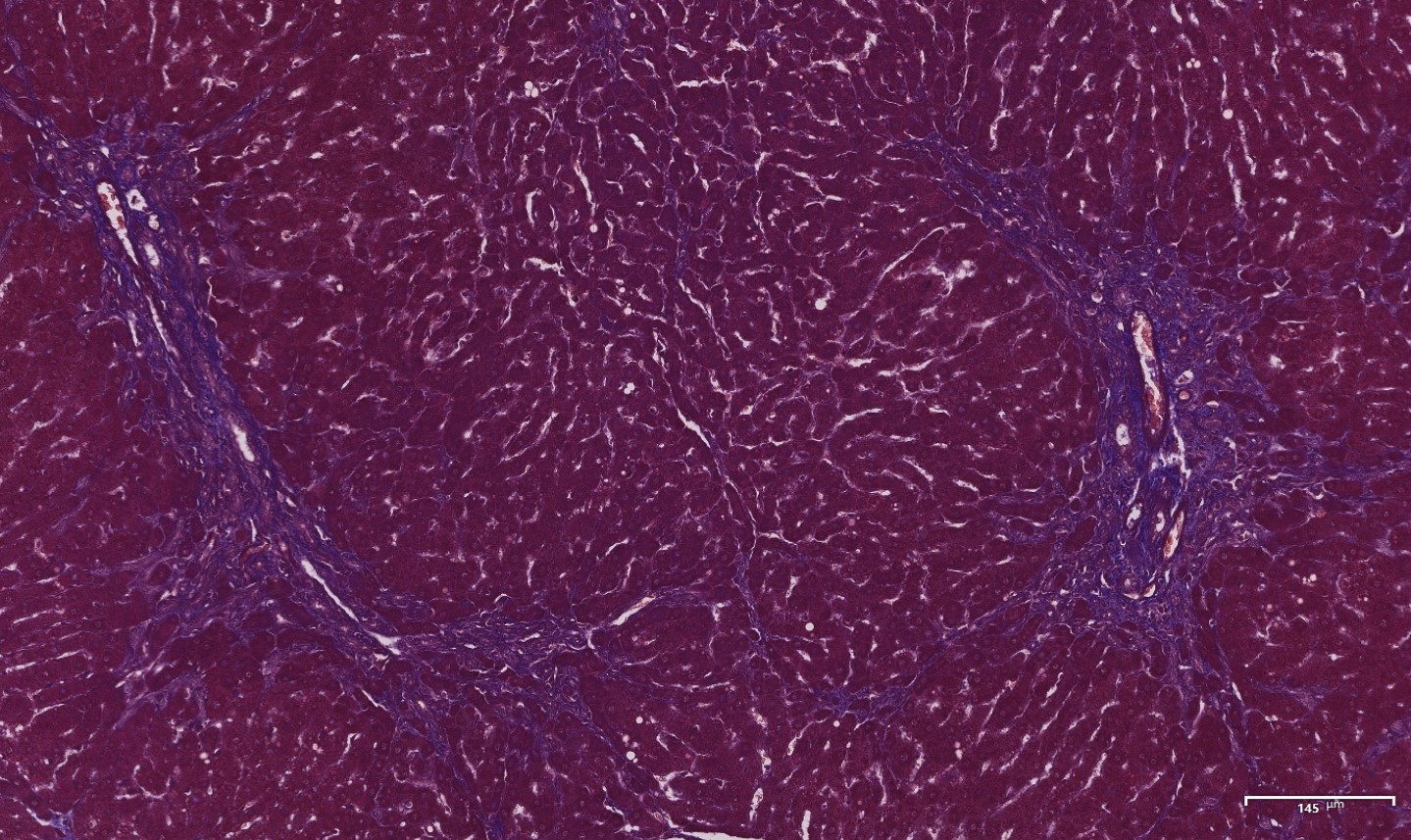

Supplement: Supplementary file 1 [file DataSheet3.ZIP › the original source data of Figures 1-4/Fig. 4/Fig. 4G (Rat-BDL)/Masson/JY5.jpg]

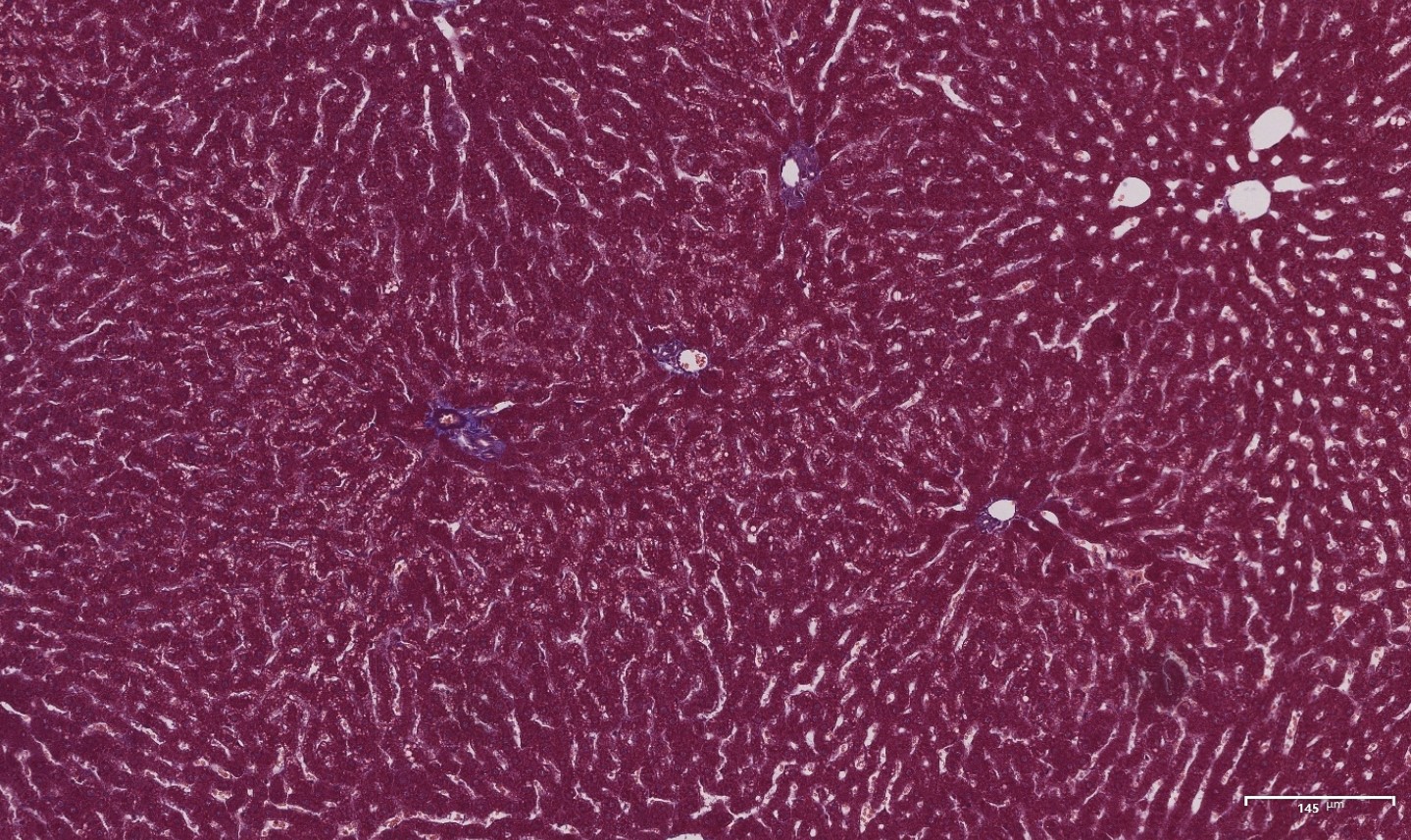

Supplement: Supplementary file 1 [file DataSheet3.ZIP › the original source data of Figures 1-4/Fig. 4/Fig. 4G (Rat-BDL)/Masson/Sham.jpg]

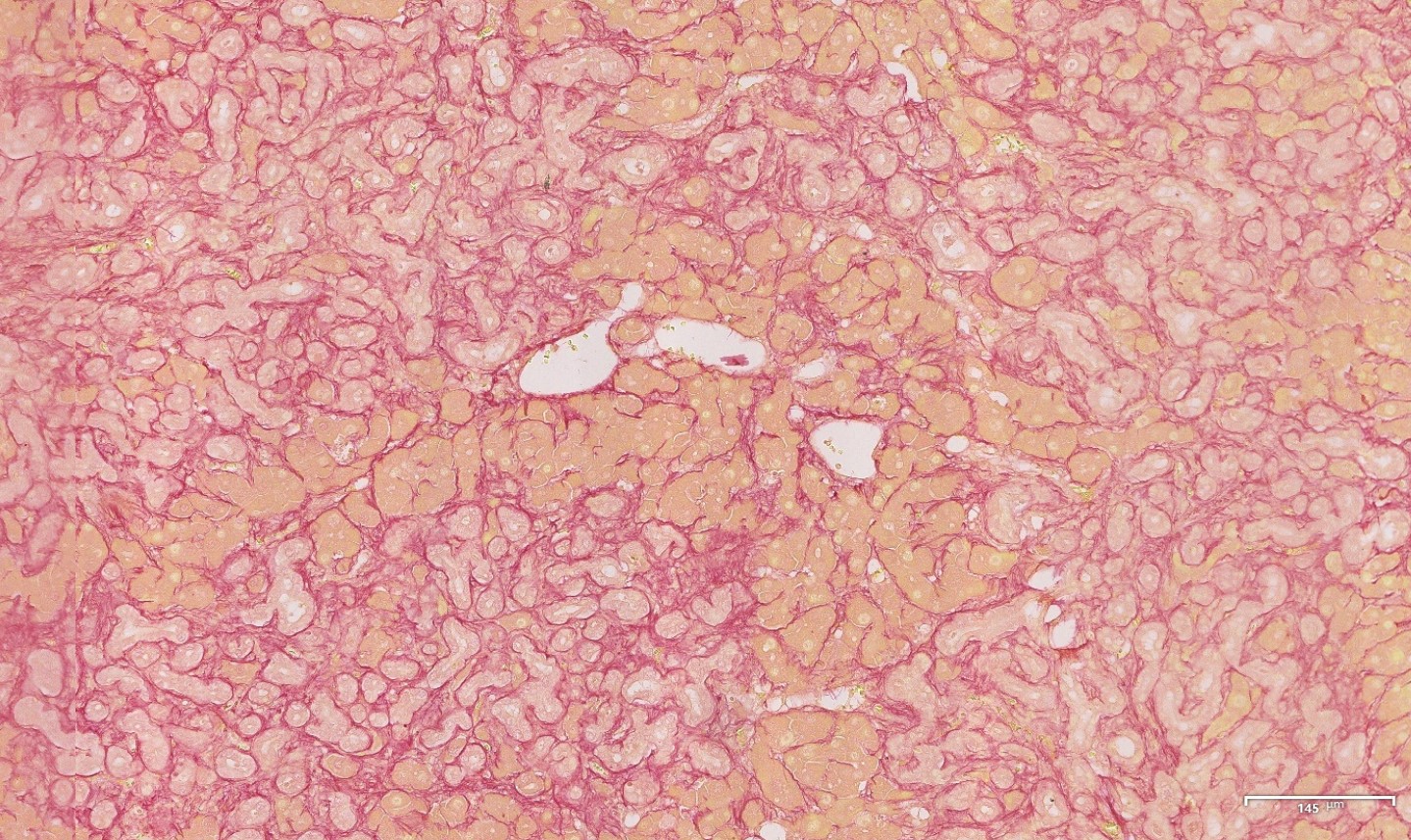

Supplement: Supplementary file 1 [file DataSheet3.ZIP › the original source data of Figures 1-4/Fig. 4/Fig. 4G (Rat-BDL)/SR/BDL.jpg]

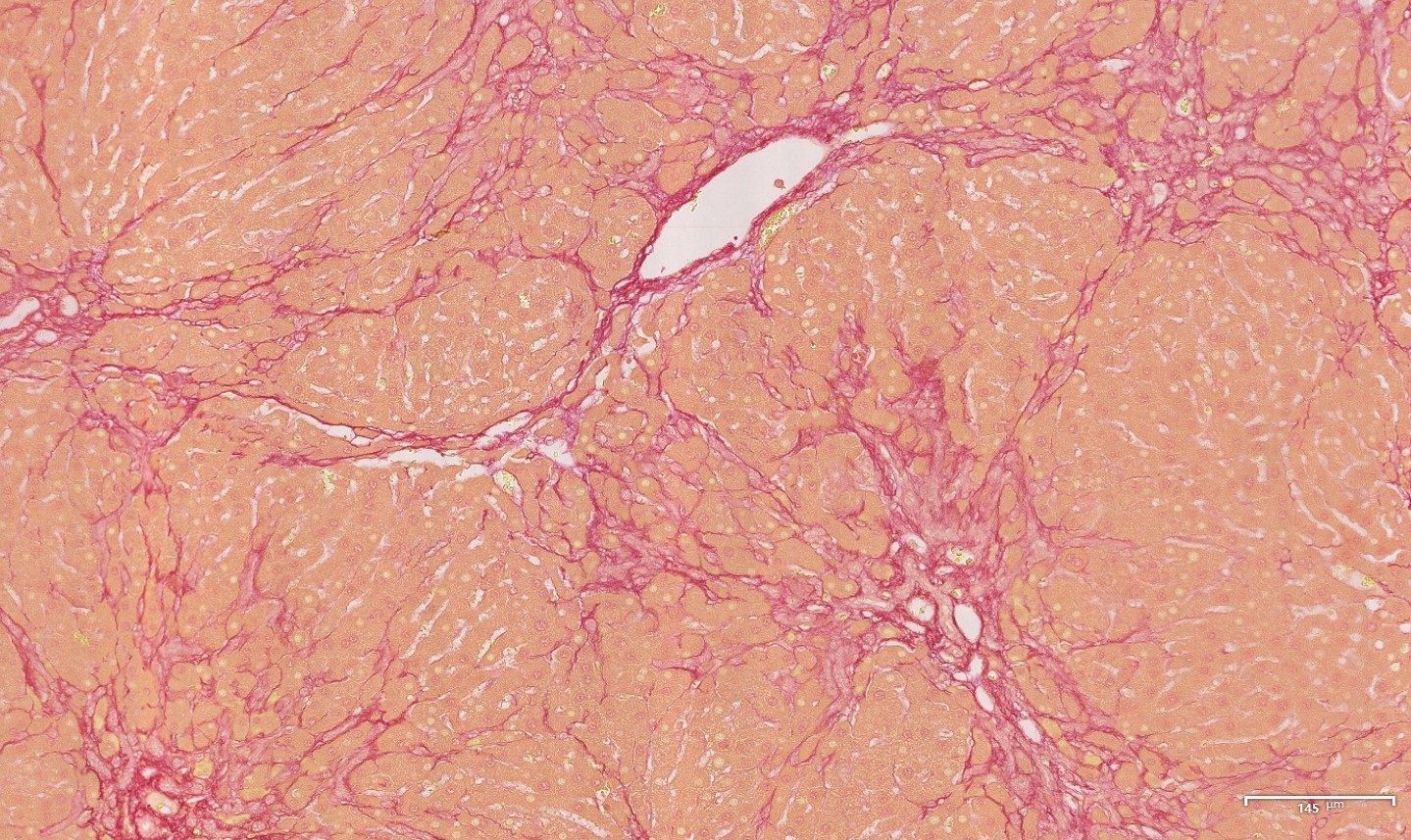

Supplement: Supplementary file 1 [file DataSheet3.ZIP › the original source data of Figures 1-4/Fig. 4/Fig. 4G (Rat-BDL)/SR/DAPT.jpg]

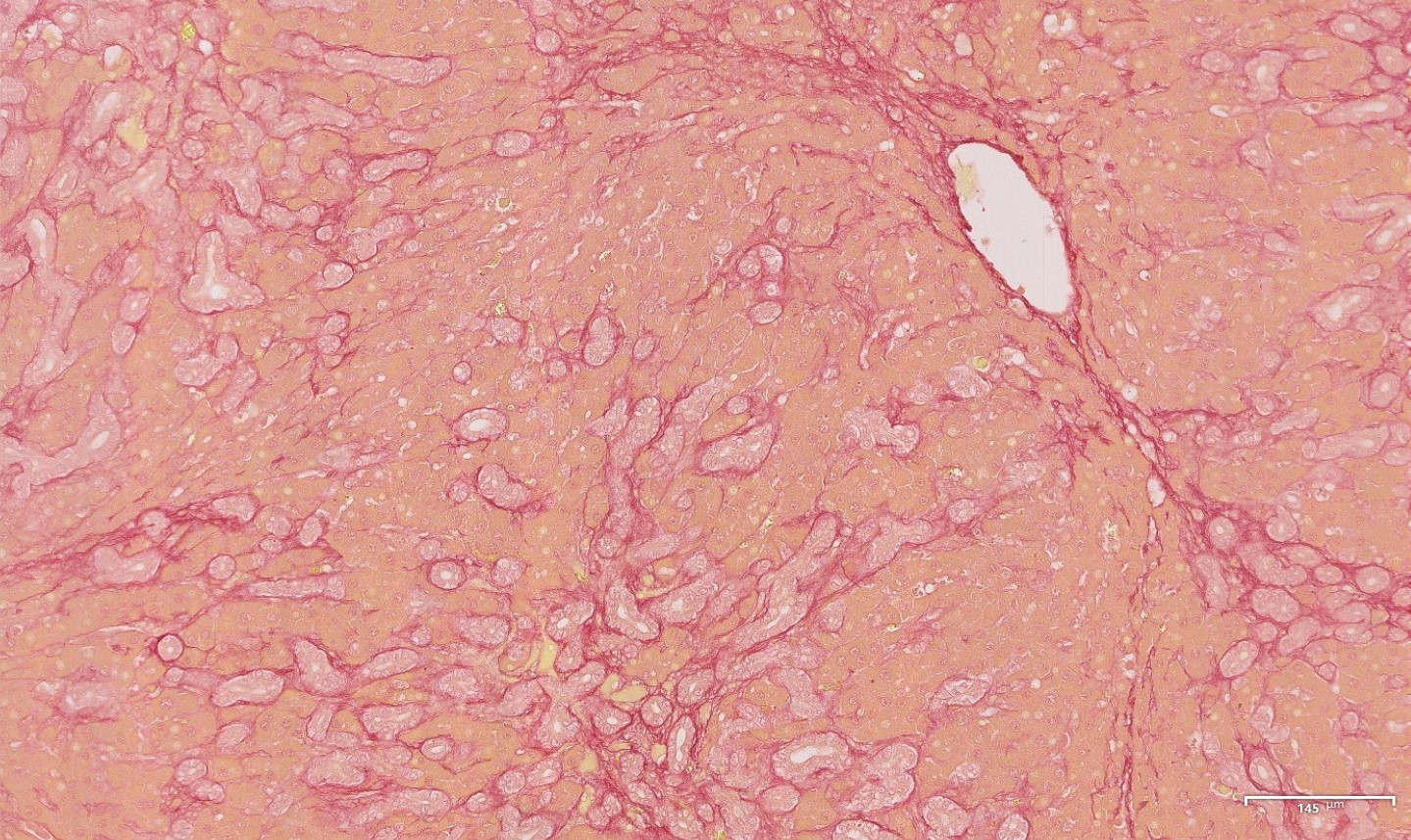

Supplement: Supplementary file 1 [file DataSheet3.ZIP › the original source data of Figures 1-4/Fig. 4/Fig. 4G (Rat-BDL)/SR/JY5.jpg]

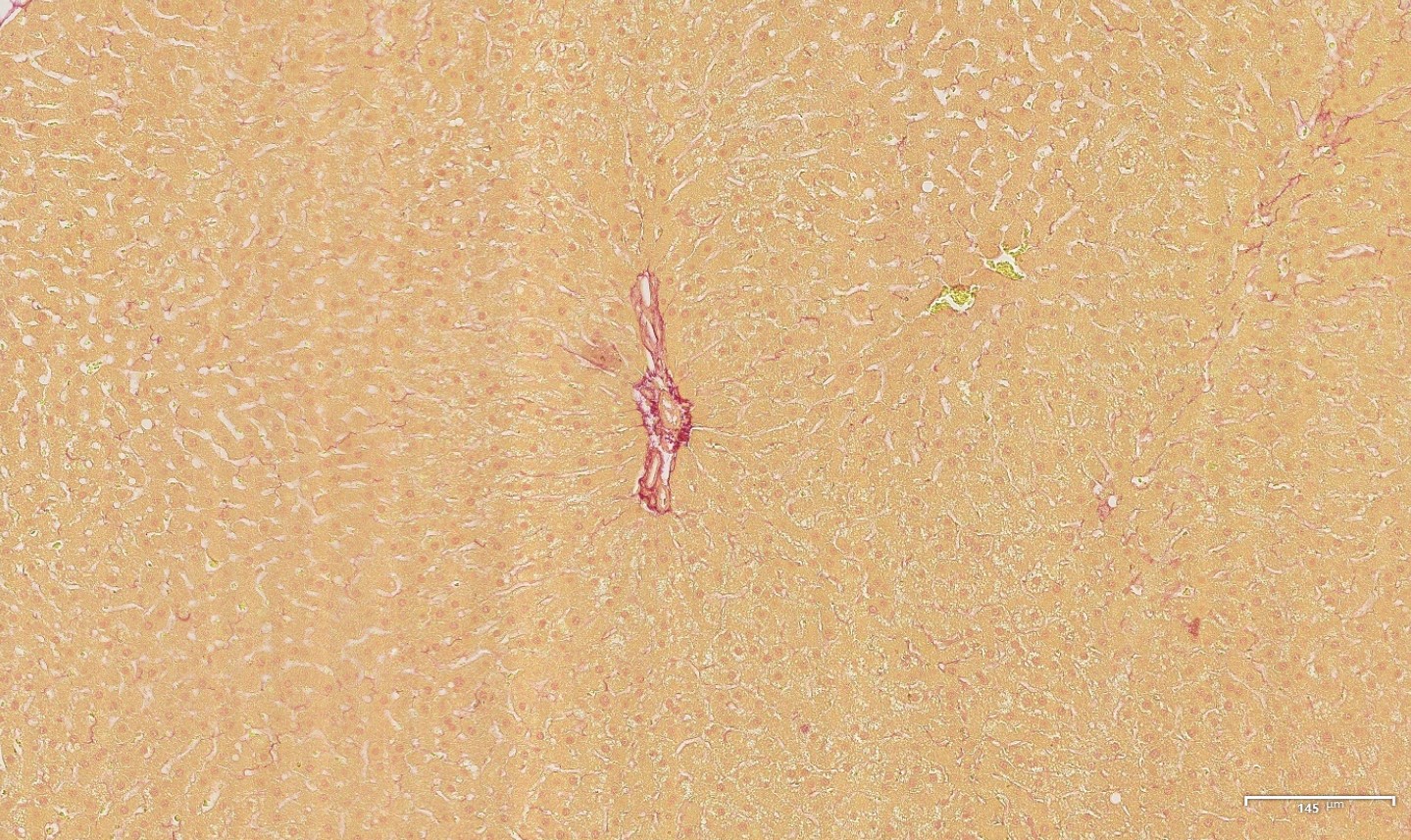

Supplement: Supplementary file 1 [file DataSheet3.ZIP › the original source data of Figures 1-4/Fig. 4/Fig. 4G (Rat-BDL)/SR/Sham.jpg]

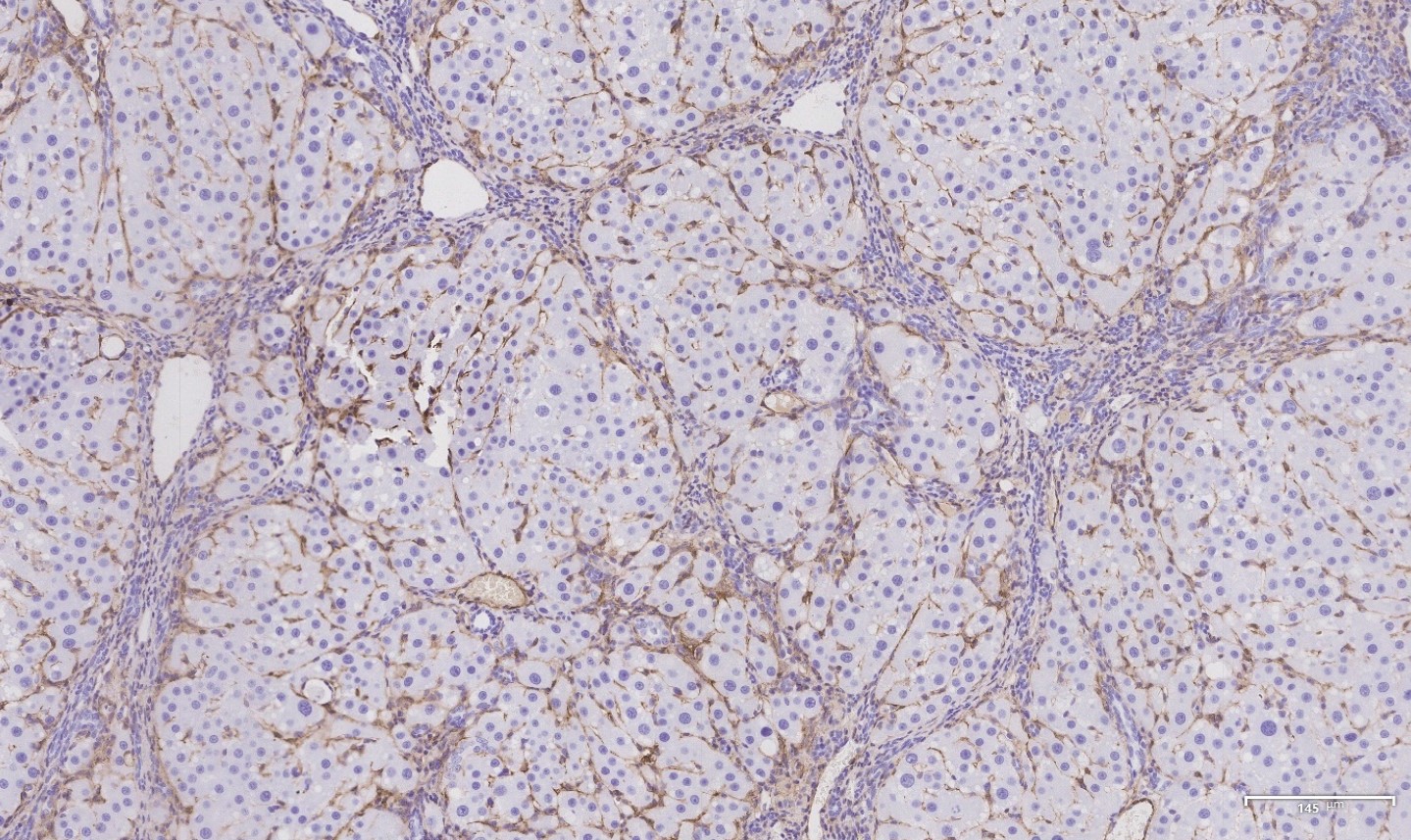

Supplement: Supplementary file 3 [file DataSheet4.ZIP › the original source data of Figures 5-7/Fig. 5/IHC/Fig. 5A (Rat-CCl4)/a-SMA/CCl4.jpg]

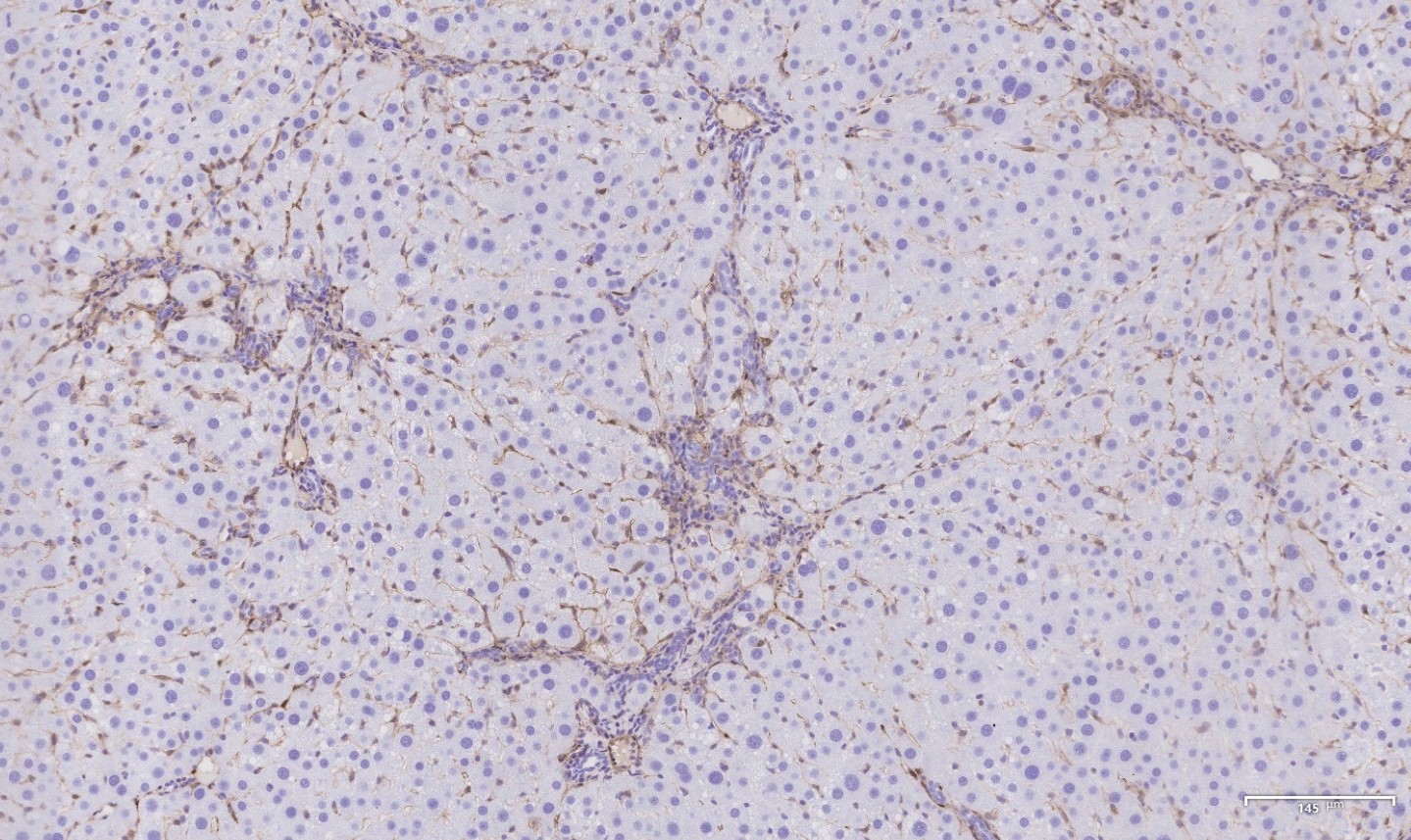

Supplement: Supplementary file 3 [file DataSheet4.ZIP › the original source data of Figures 5-7/Fig. 5/IHC/Fig. 5A (Rat-CCl4)/a-SMA/JY5.jpg]

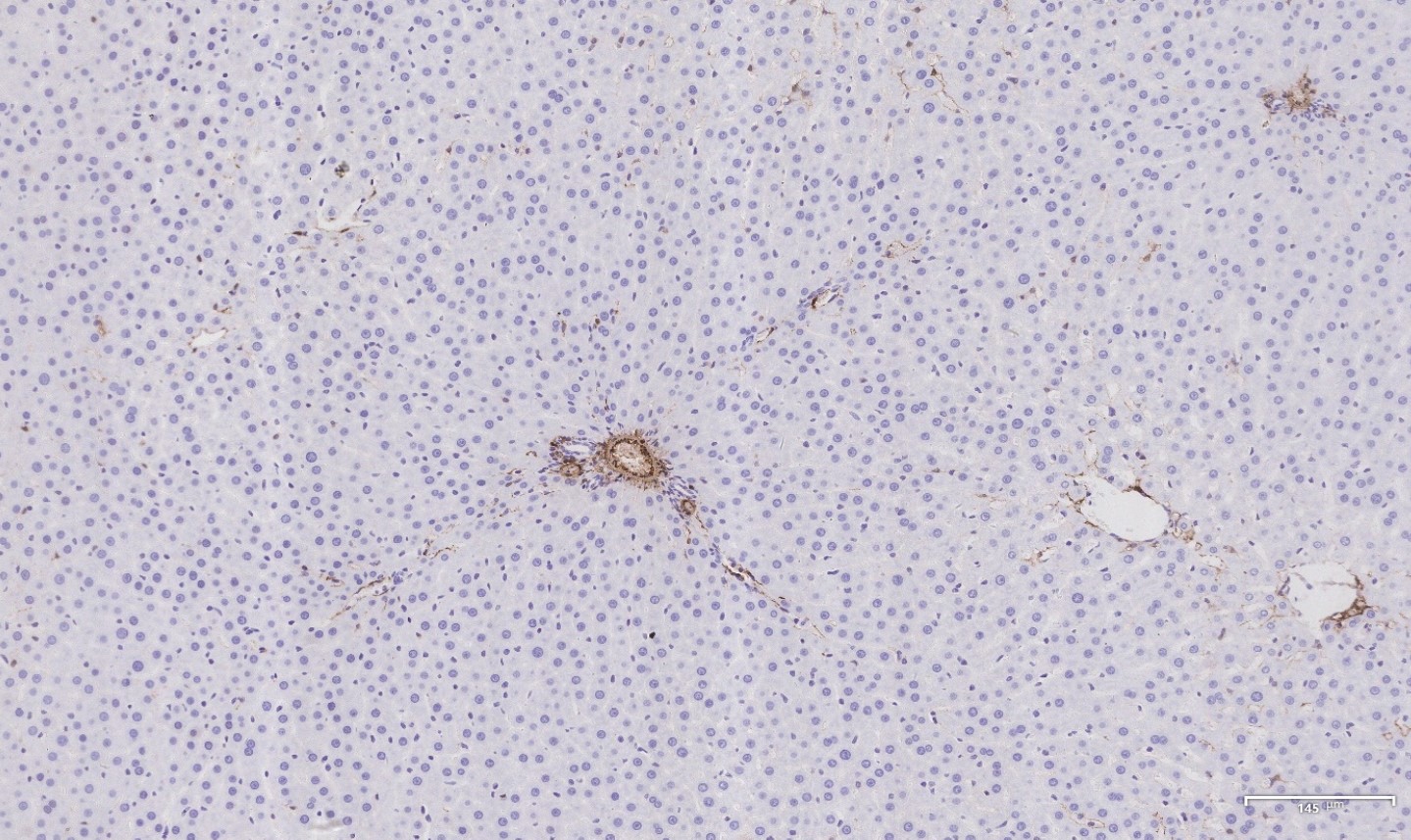

Supplement: Supplementary file 3 [file DataSheet4.ZIP › the original source data of Figures 5-7/Fig. 5/IHC/Fig. 5A (Rat-CCl4)/a-SMA/Oil.jpg]

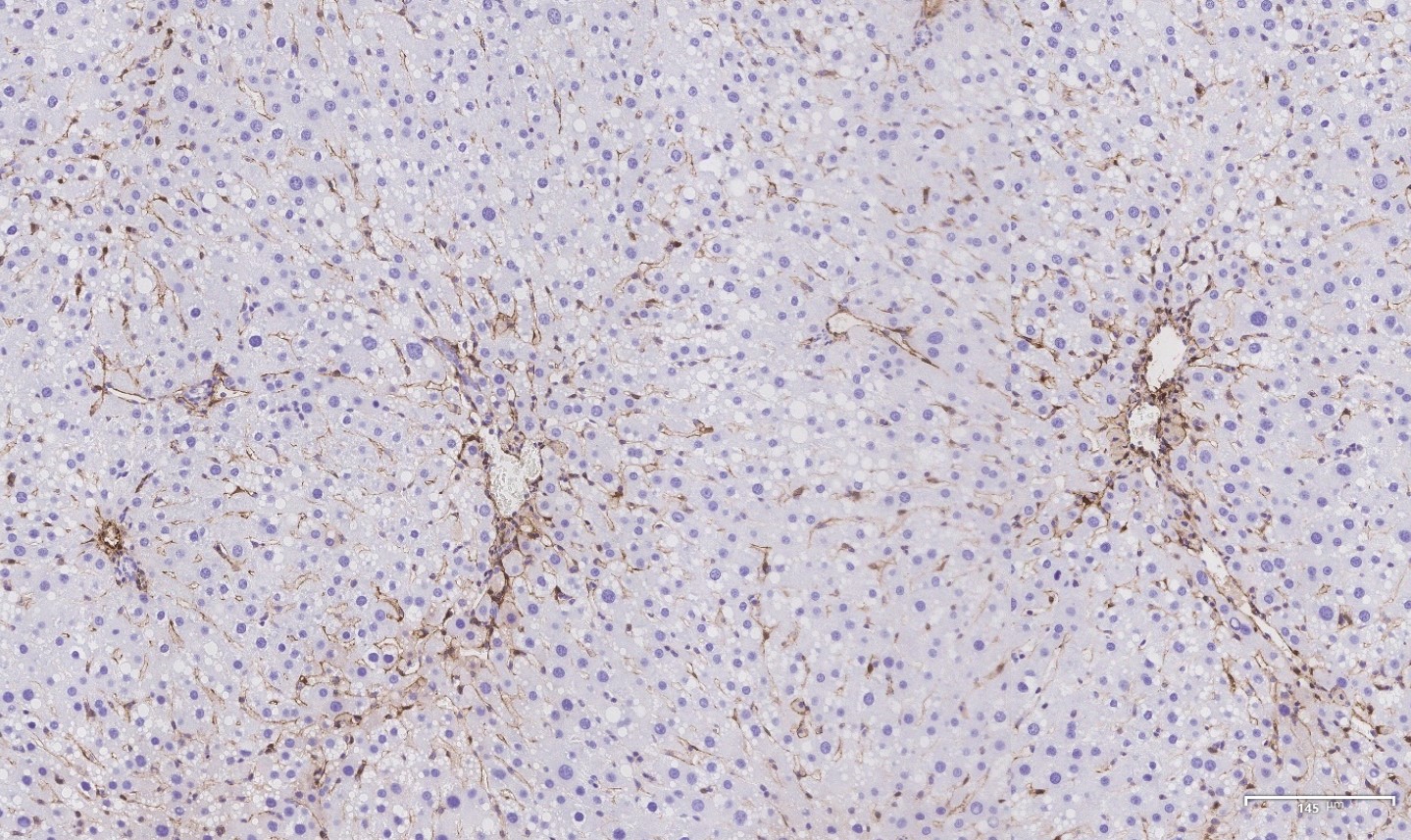

Supplement: Supplementary file 3 [file DataSheet4.ZIP › the original source data of Figures 5-7/Fig. 5/IHC/Fig. 5A (Rat-CCl4)/a-SMA/SORA.jpg]

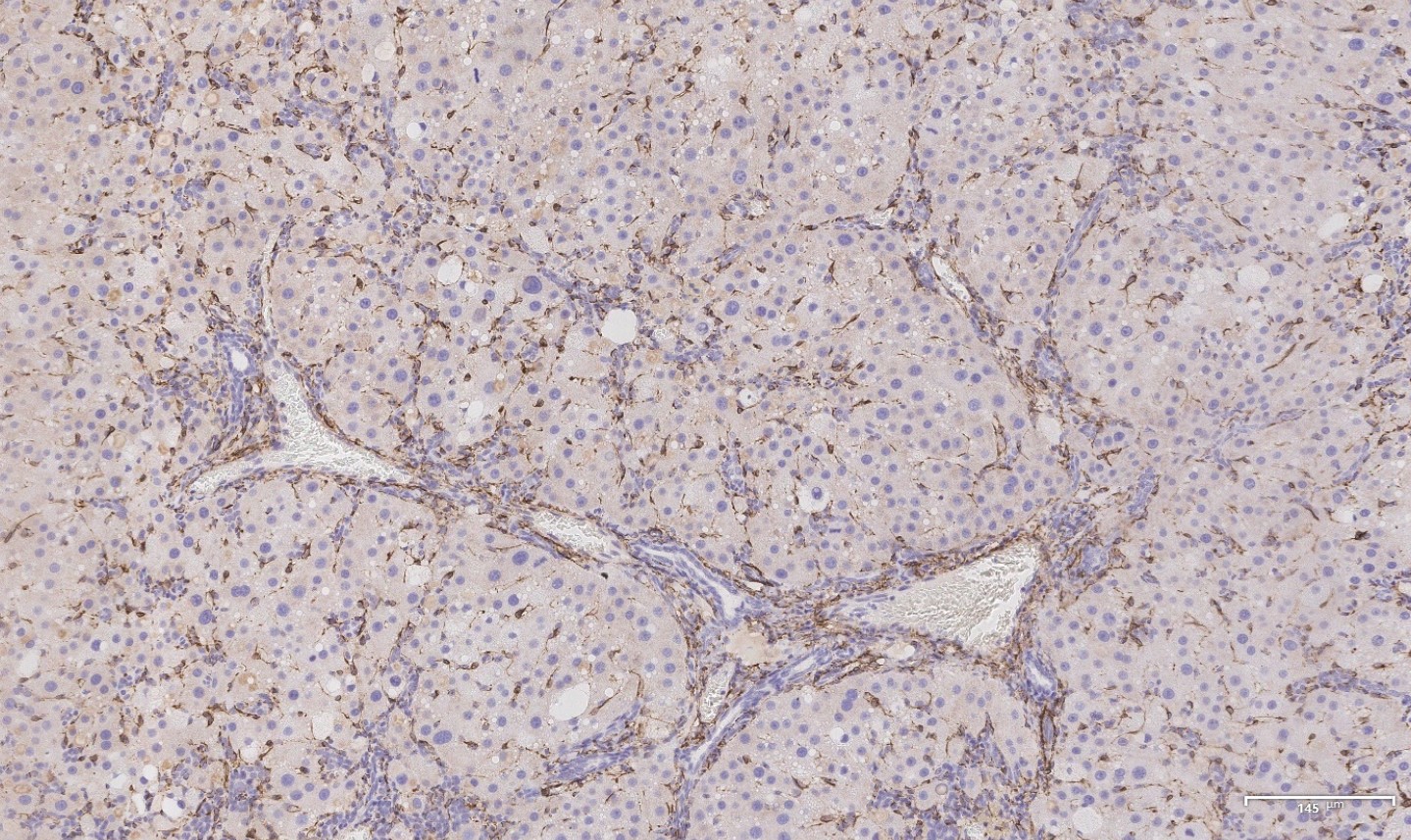

Supplement: Supplementary file 3 [file DataSheet4.ZIP › the original source data of Figures 5-7/Fig. 5/IHC/Fig. 5A (Rat-CCl4)/Desmin/CCl4.jpg]

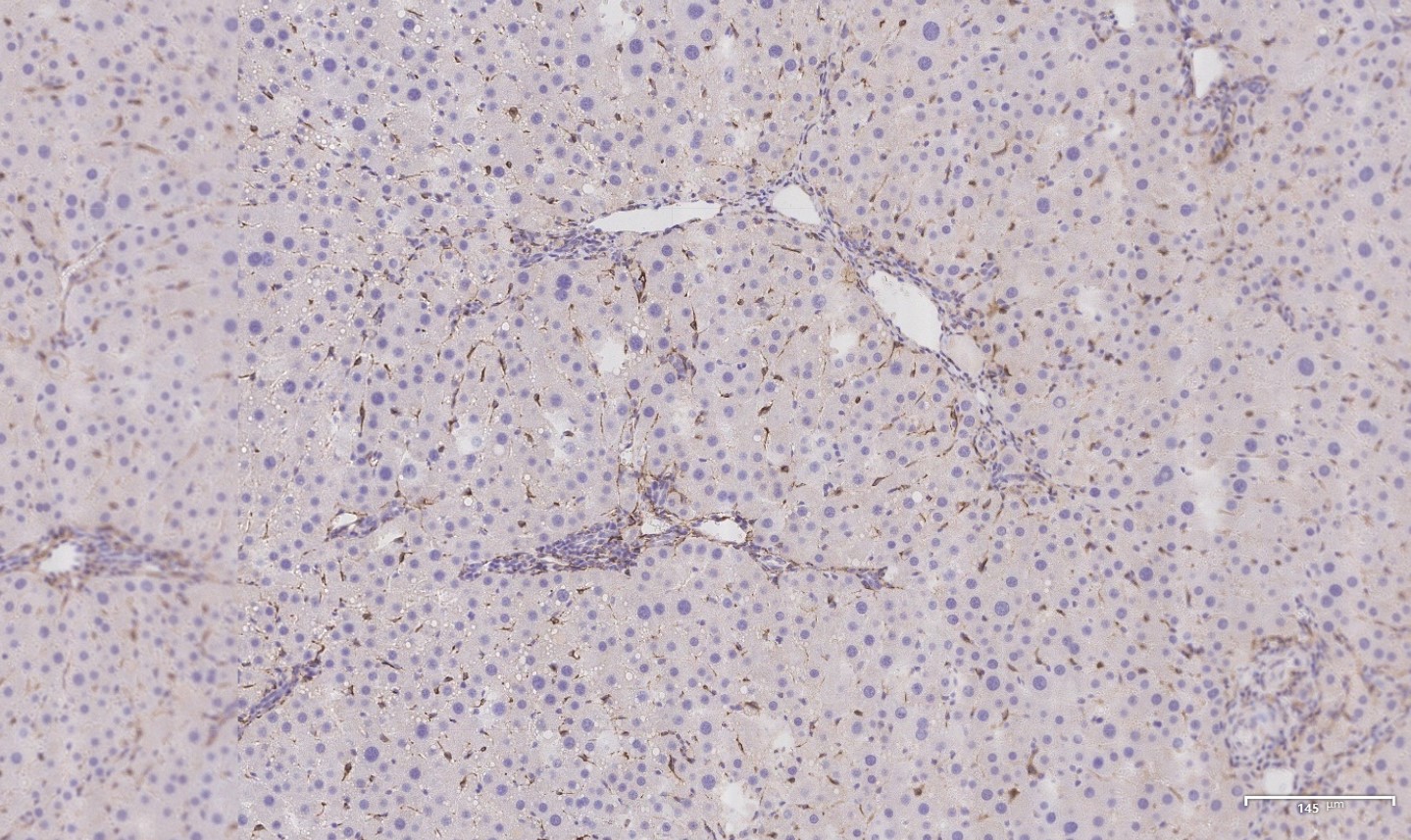

Supplement: Supplementary file 3 [file DataSheet4.ZIP › the original source data of Figures 5-7/Fig. 5/IHC/Fig. 5A (Rat-CCl4)/Desmin/JY5.jpg]

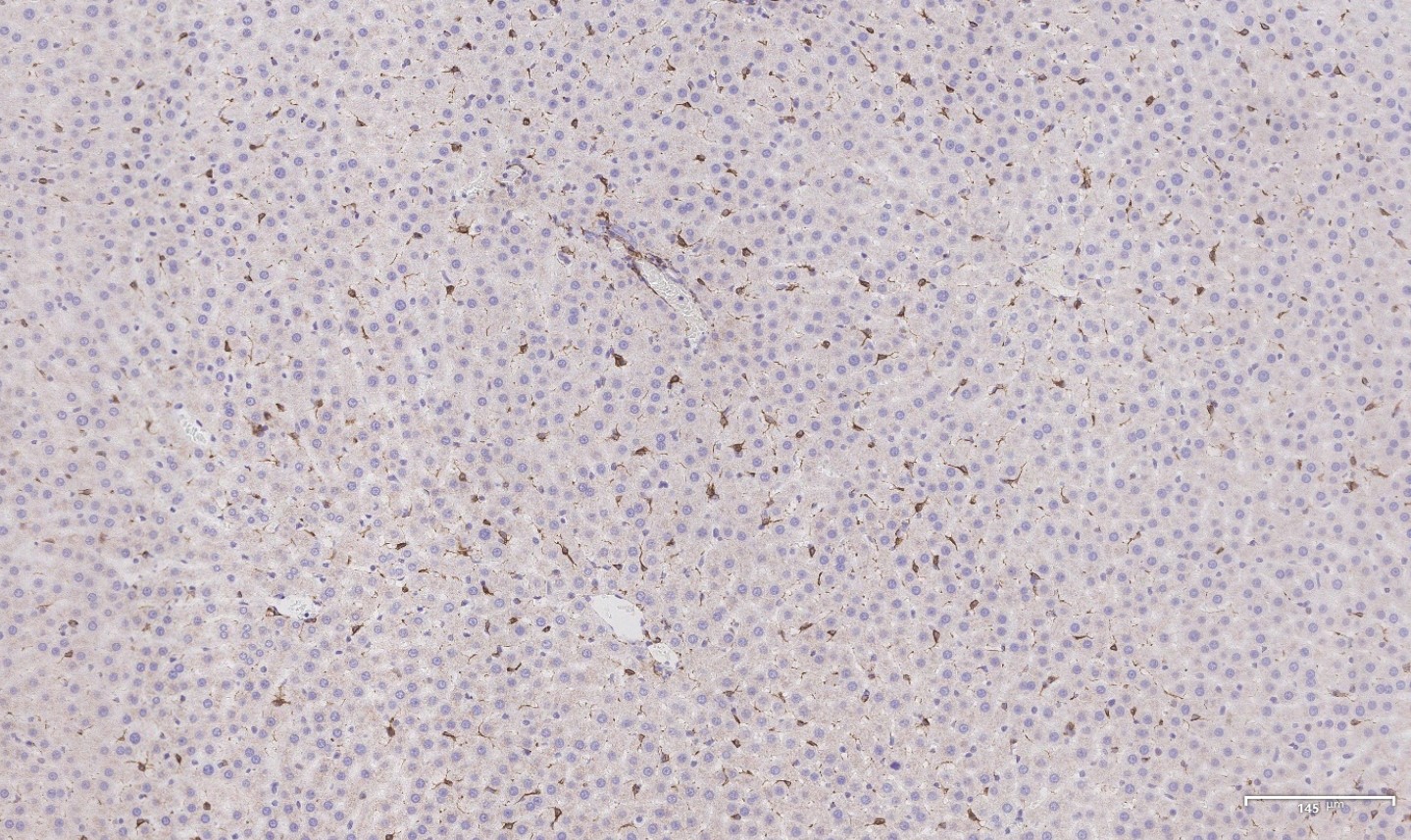

Supplement: Supplementary file 3 [file DataSheet4.ZIP › the original source data of Figures 5-7/Fig. 5/IHC/Fig. 5A (Rat-CCl4)/Desmin/Oil.jpg]

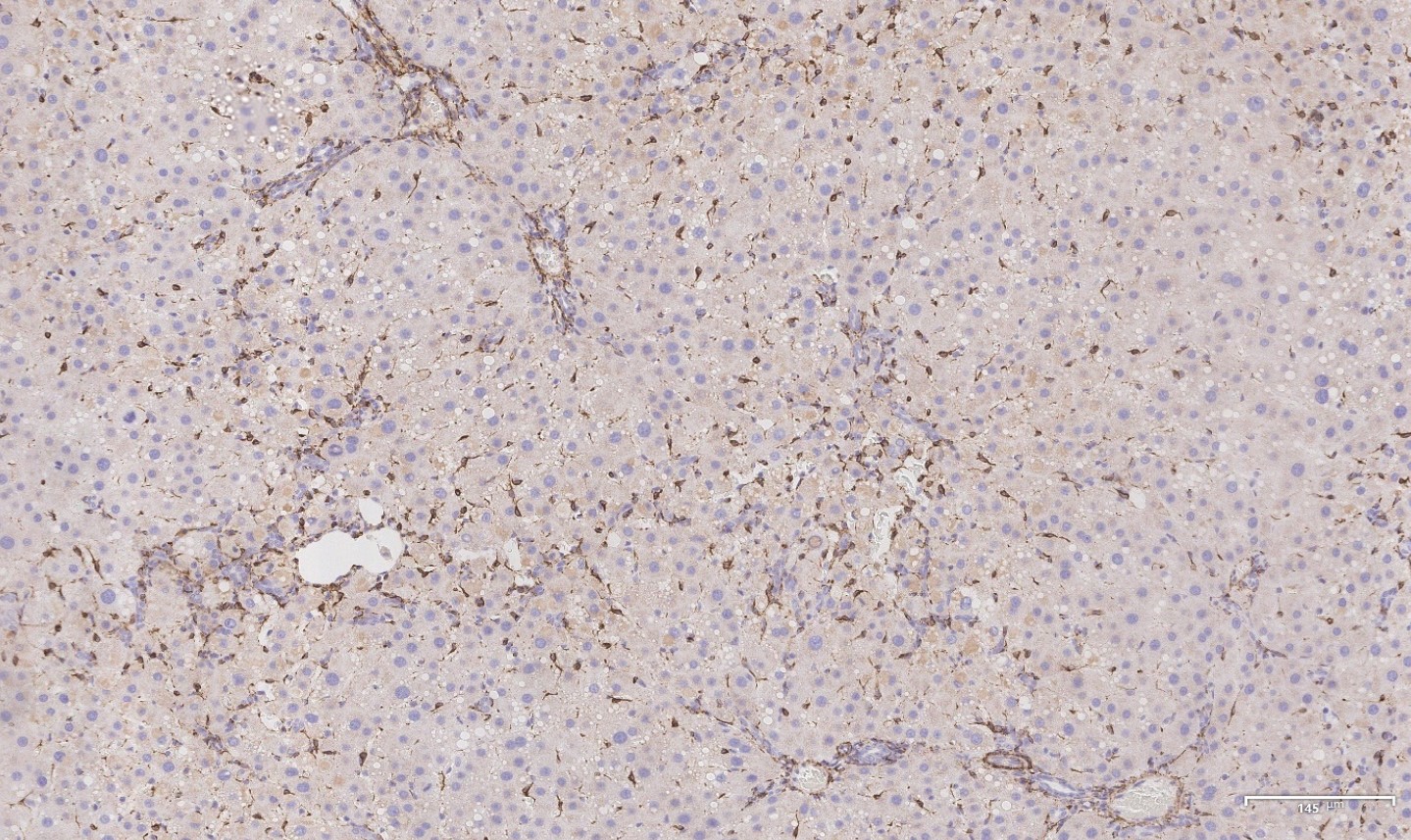

Supplement: Supplementary file 3 [file DataSheet4.ZIP › the original source data of Figures 5-7/Fig. 5/IHC/Fig. 5A (Rat-CCl4)/Desmin/SORA.jpg]

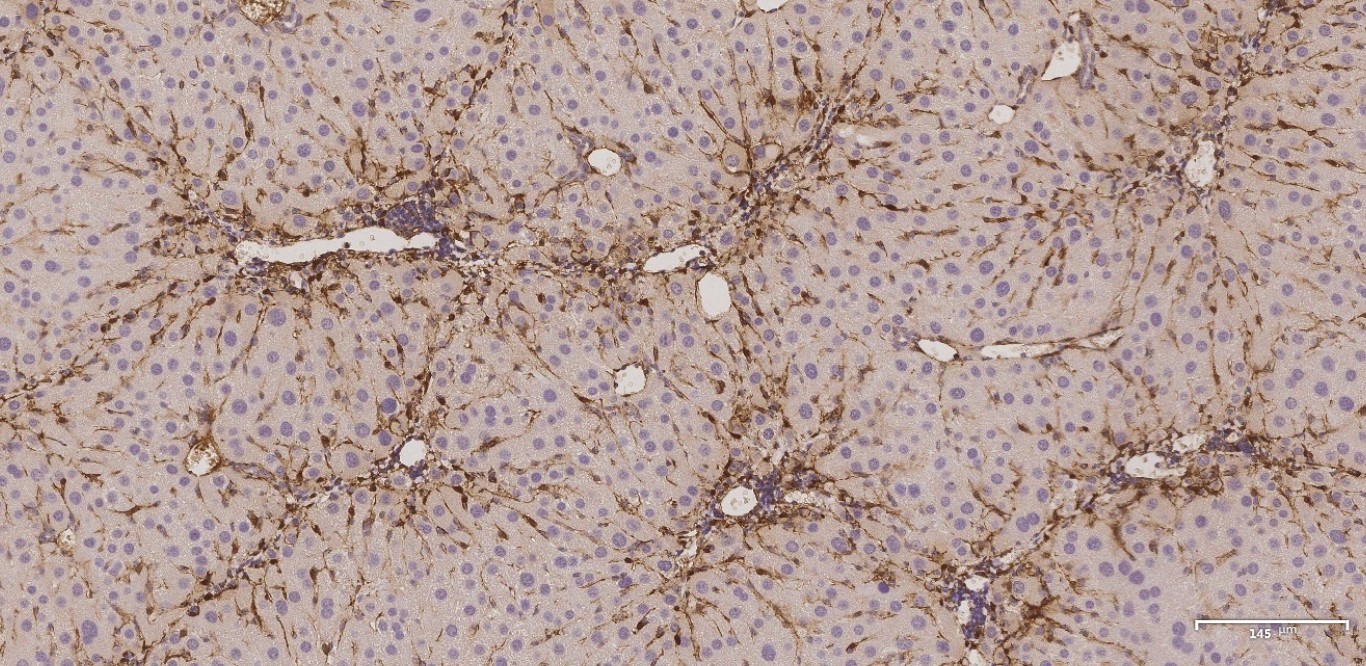

Supplement: Supplementary file 3 [file DataSheet4.ZIP › the original source data of Figures 5-7/Fig. 5/IHC/Fig. 5F (Mice-CCl4)/a-SMA/CCl4.jpg]

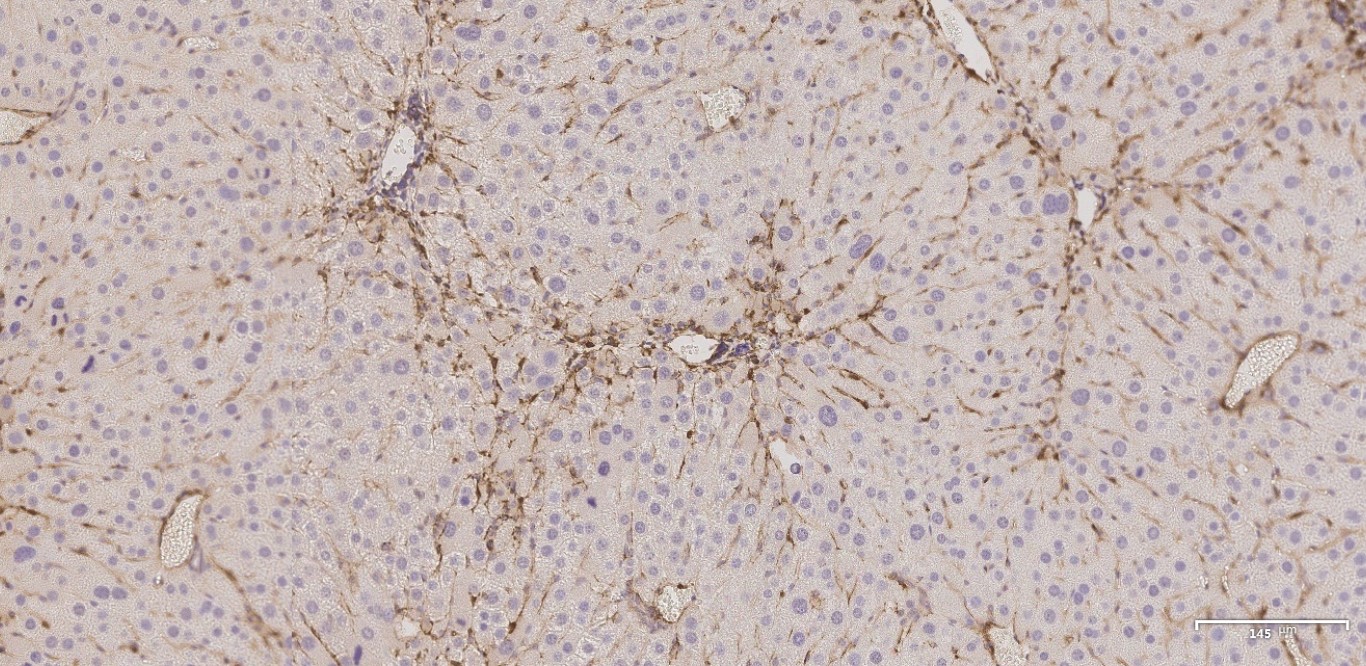

Supplement: Supplementary file 3 [file DataSheet4.ZIP › the original source data of Figures 5-7/Fig. 5/IHC/Fig. 5F (Mice-CCl4)/a-SMA/JY5.jpg]

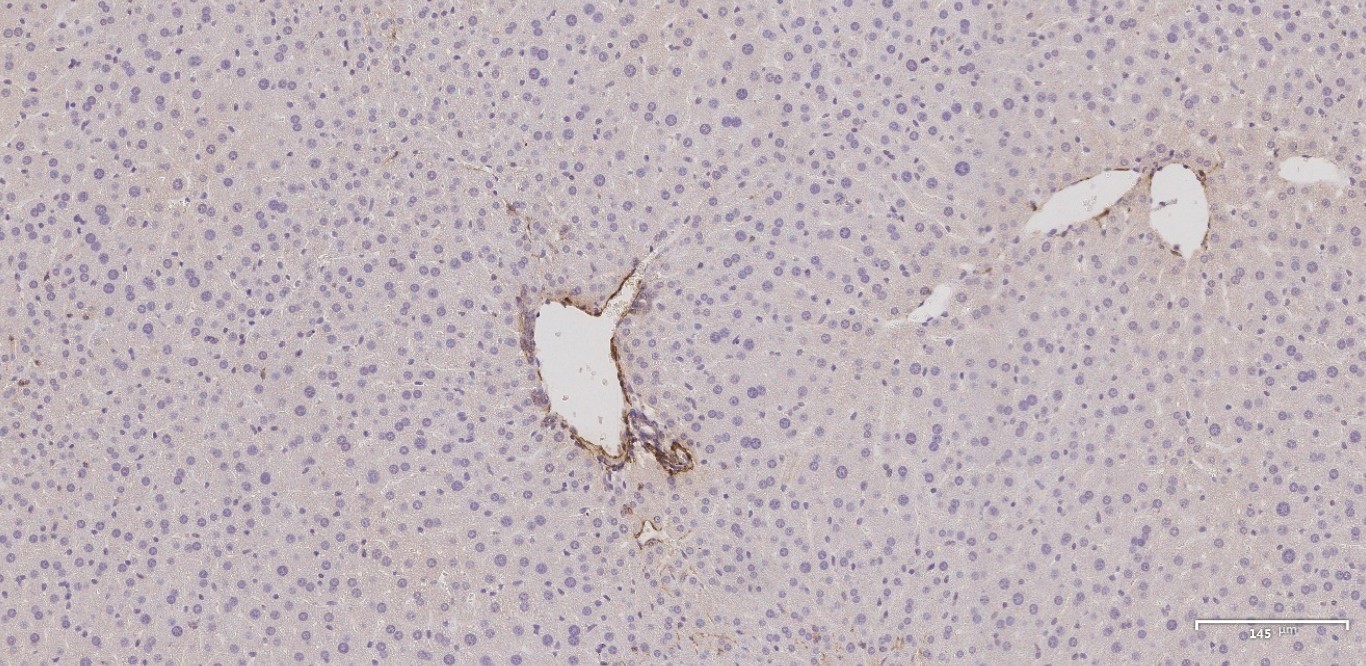

Supplement: Supplementary file 3 [file DataSheet4.ZIP › the original source data of Figures 5-7/Fig. 5/IHC/Fig. 5F (Mice-CCl4)/a-SMA/Oil.jpg]

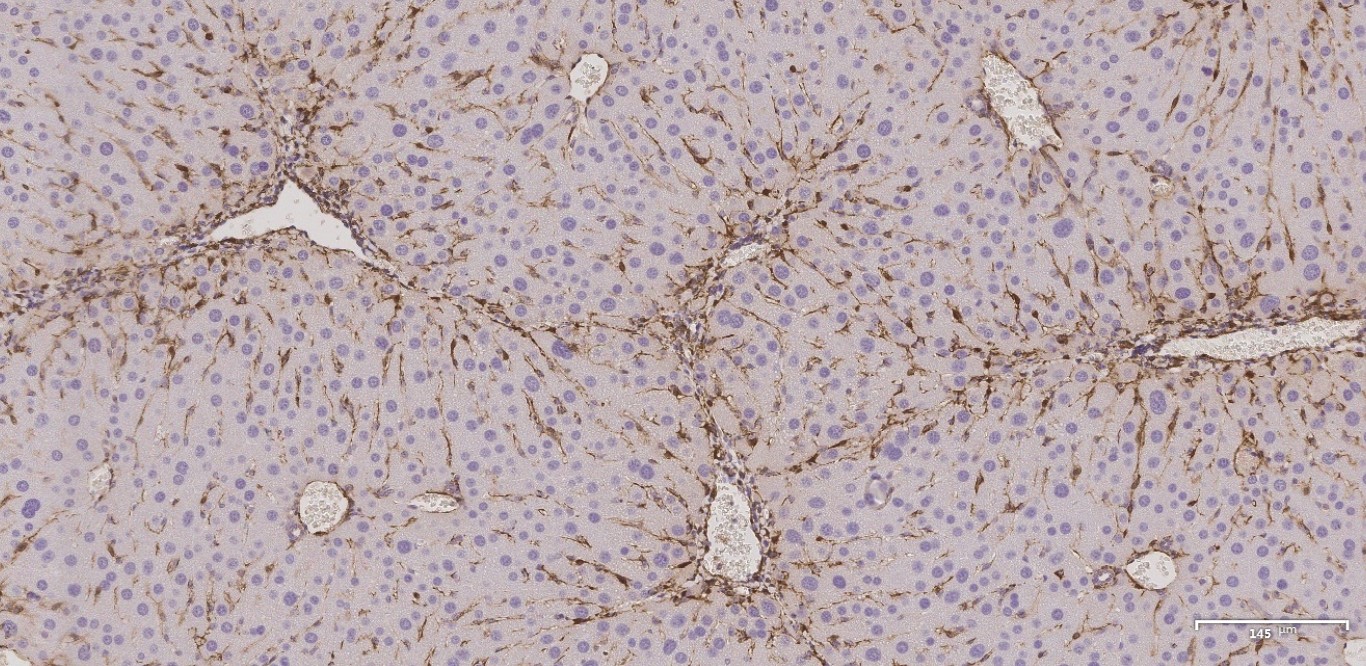

Supplement: Supplementary file 3 [file DataSheet4.ZIP › the original source data of Figures 5-7/Fig. 5/IHC/Fig. 5F (Mice-CCl4)/a-SMA/SORA.jpg]

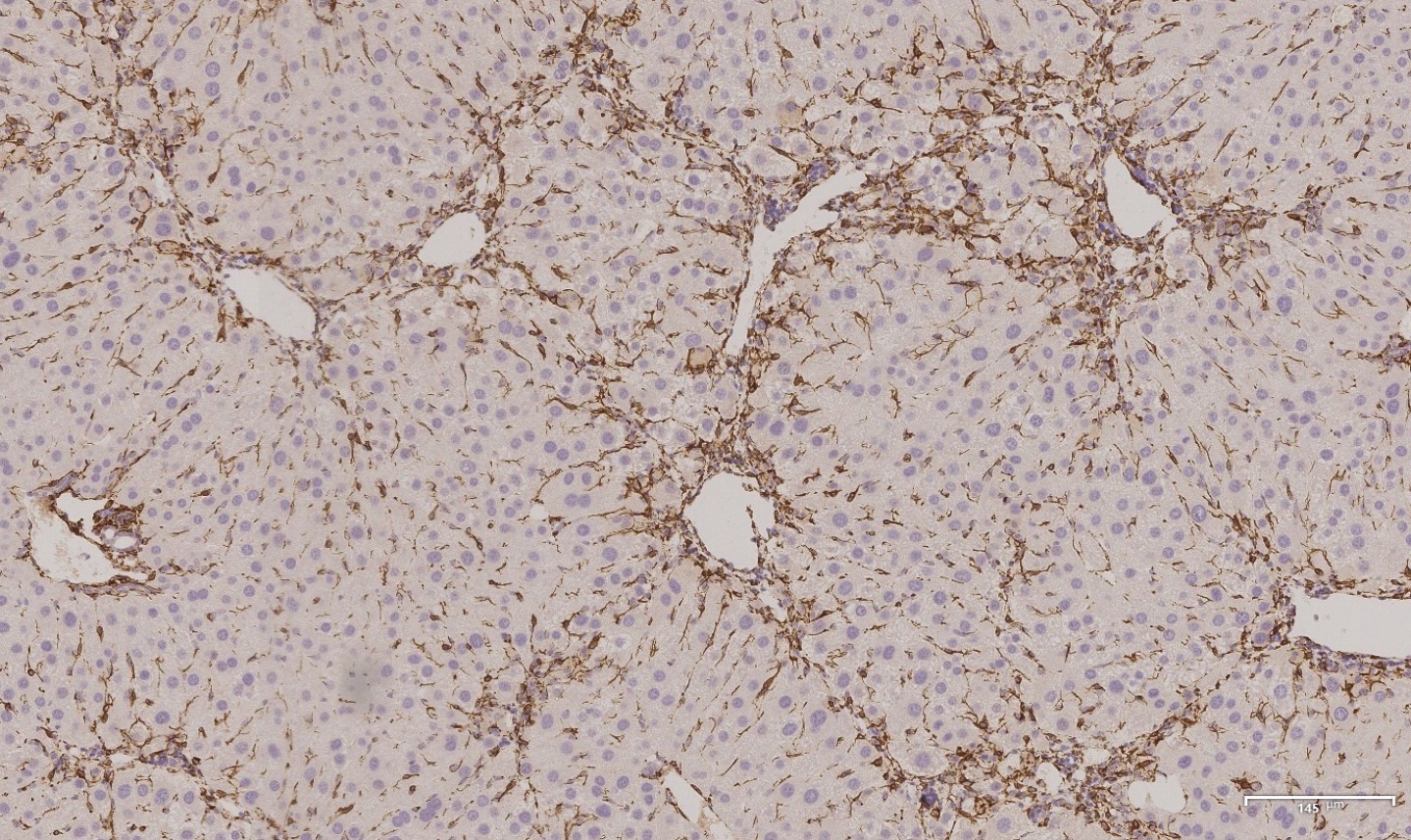

Supplement: Supplementary file 3 [file DataSheet4.ZIP › the original source data of Figures 5-7/Fig. 5/IHC/Fig. 5F (Mice-CCl4)/Desmin/CCl4.jpg]

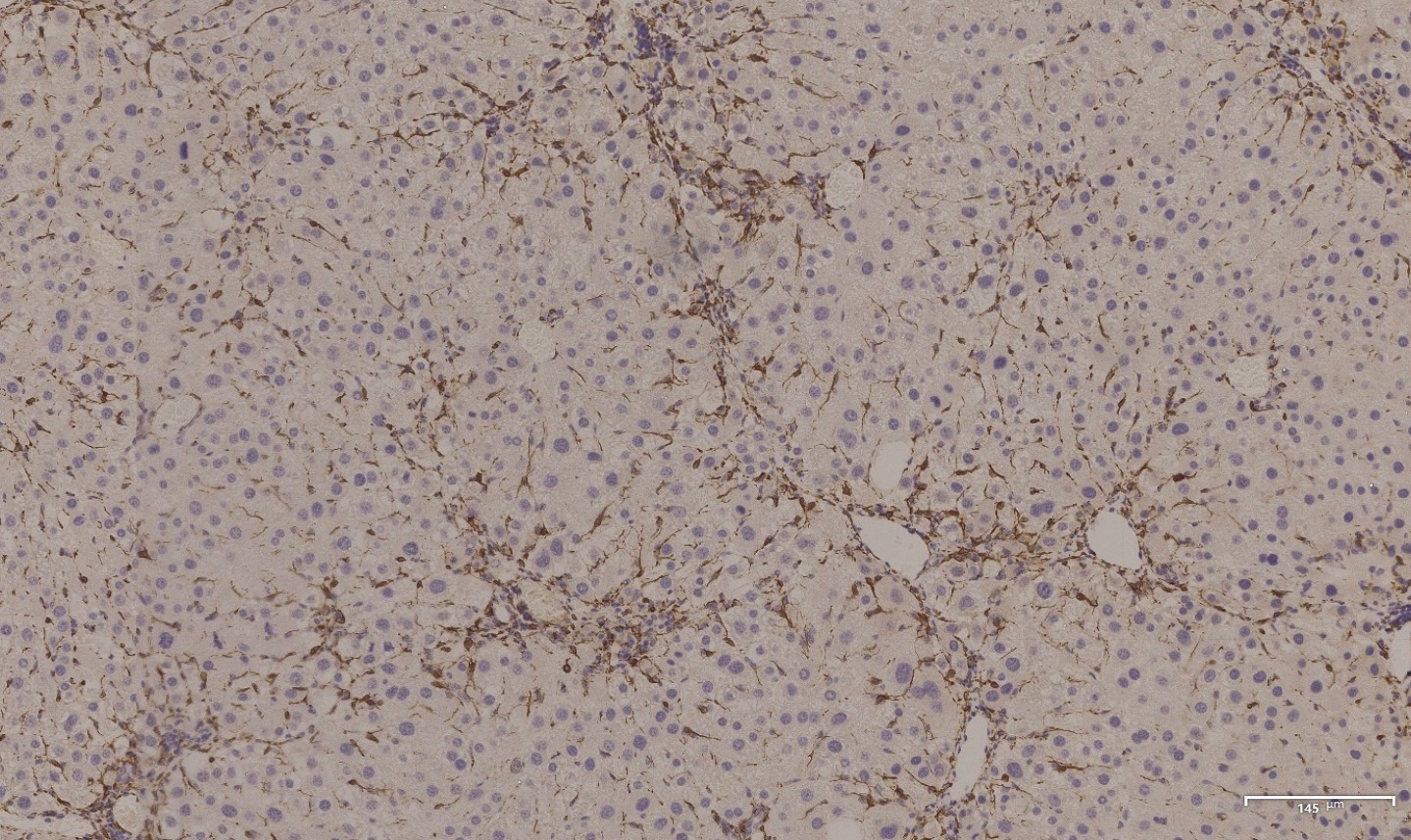

Supplement: Supplementary file 3 [file DataSheet4.ZIP › the original source data of Figures 5-7/Fig. 5/IHC/Fig. 5F (Mice-CCl4)/Desmin/JY5.jpg]

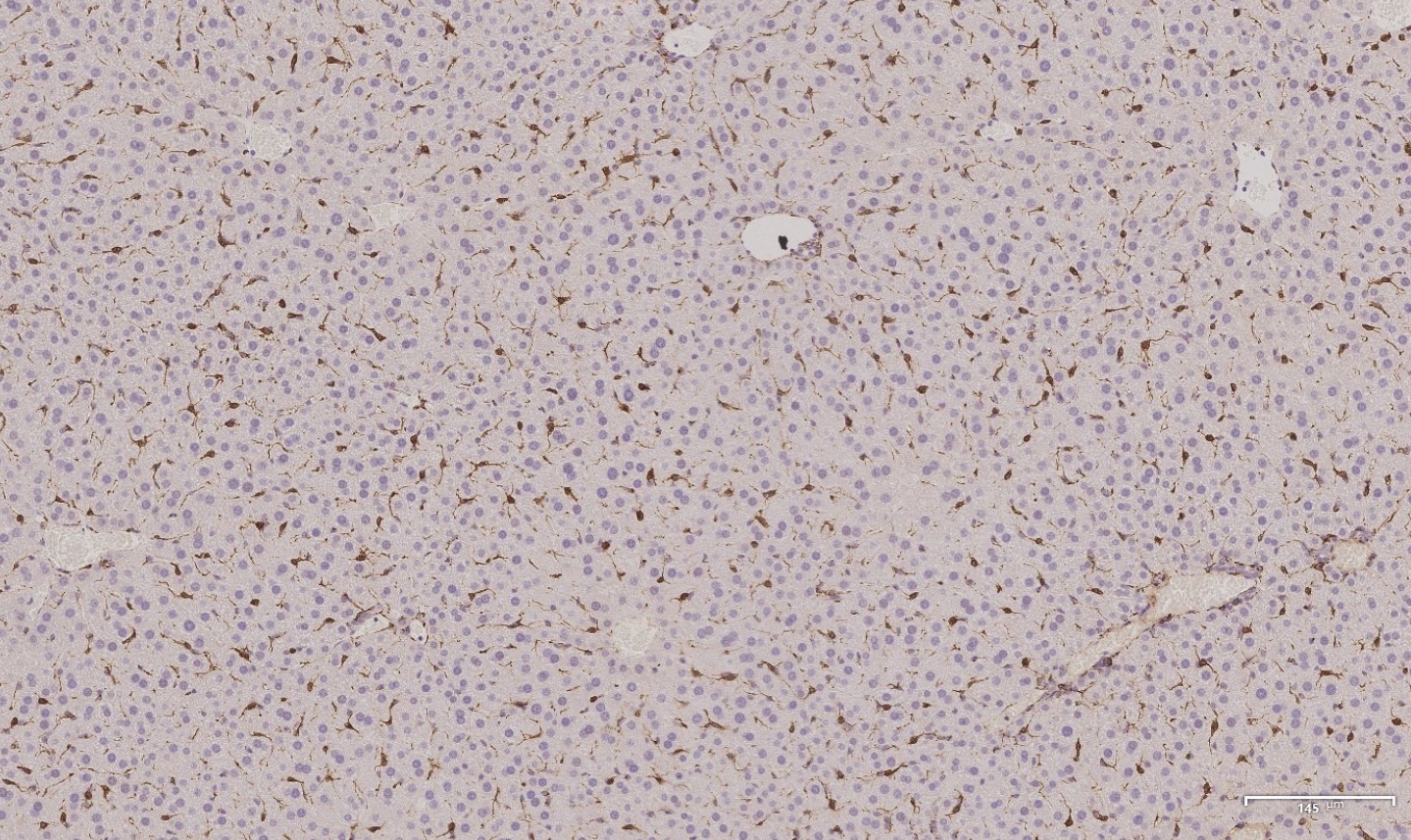

Supplement: Supplementary file 3 [file DataSheet4.ZIP › the original source data of Figures 5-7/Fig. 5/IHC/Fig. 5F (Mice-CCl4)/Desmin/Oil.jpg]

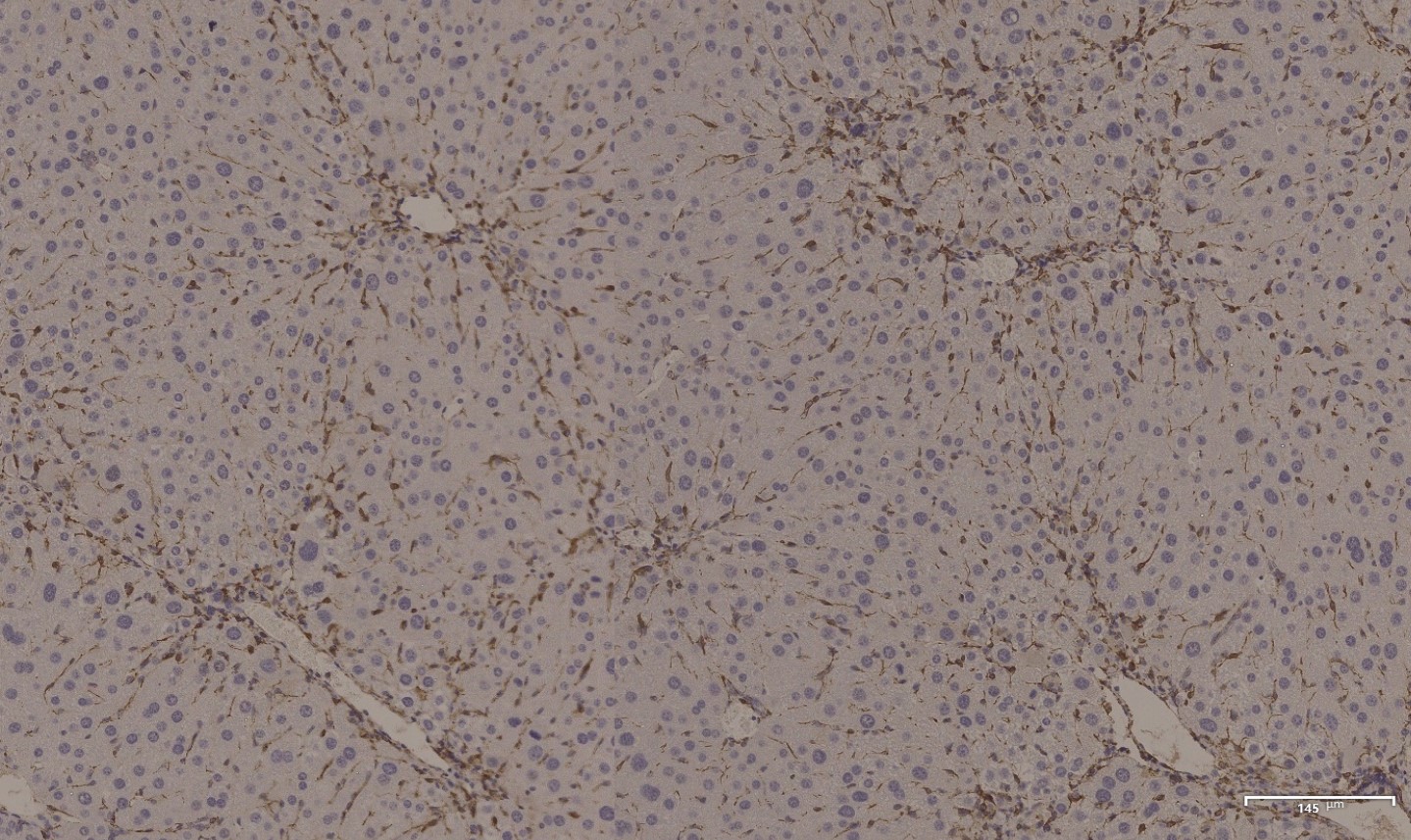

Supplement: Supplementary file 3 [file DataSheet4.ZIP › the original source data of Figures 5-7/Fig. 5/IHC/Fig. 5F (Mice-CCl4)/Desmin/SORA.jpg]

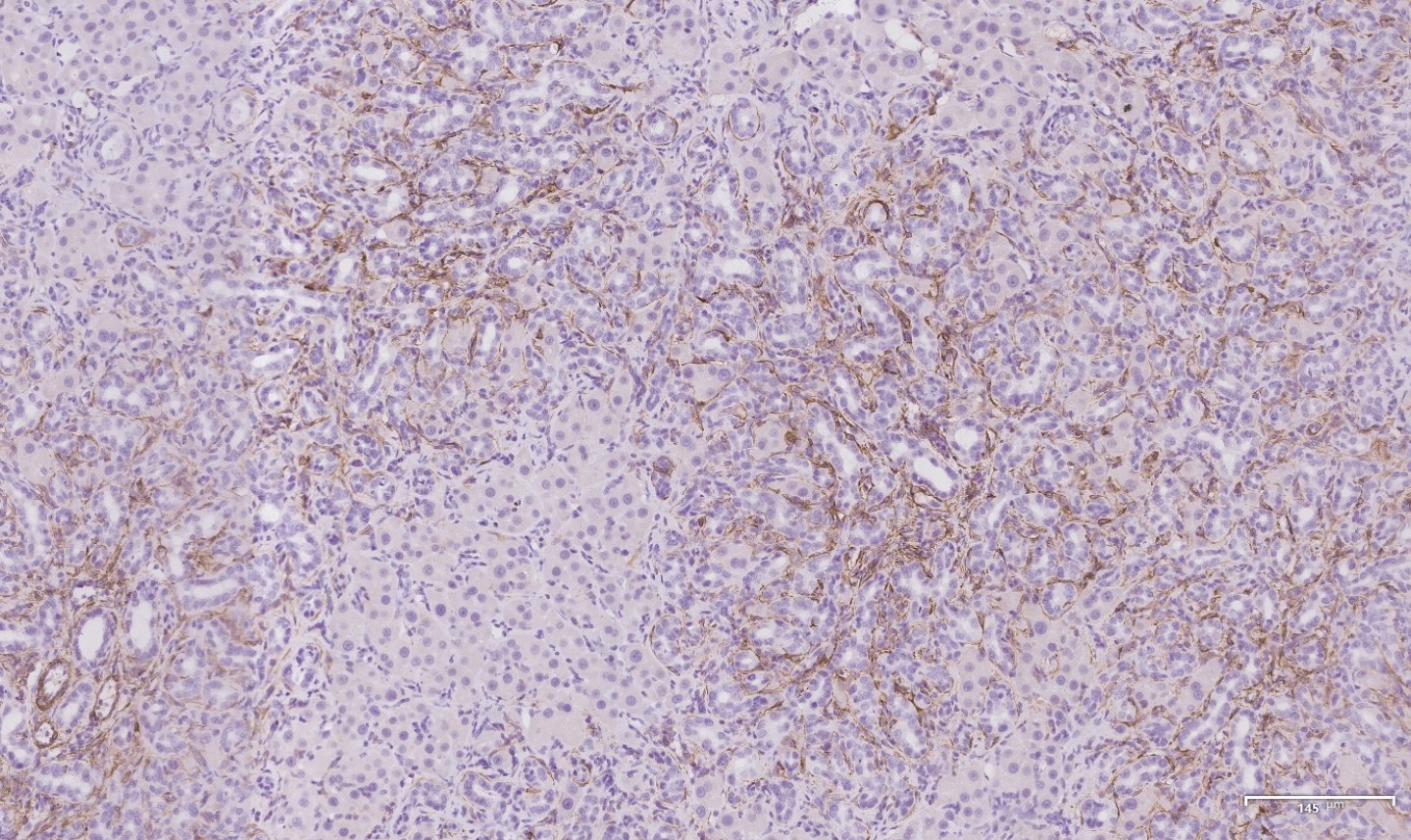

Supplement: Supplementary file 3 [file DataSheet4.ZIP › the original source data of Figures 5-7/Fig. 5/IHC/Fig. 5K (Rat-BDL)/a-SMA/BDL.jpg]

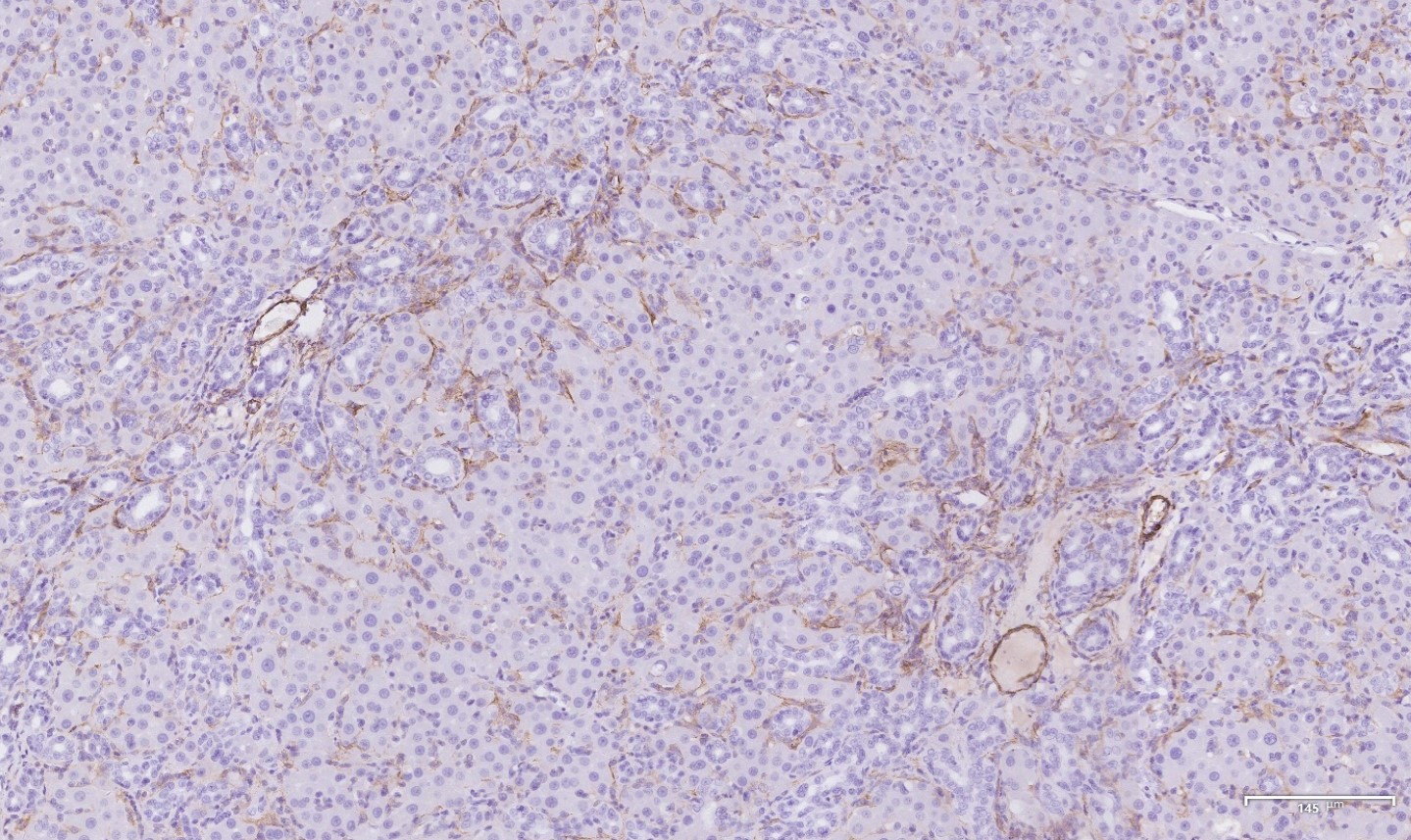

Supplement: Supplementary file 3 [file DataSheet4.ZIP › the original source data of Figures 5-7/Fig. 5/IHC/Fig. 5K (Rat-BDL)/a-SMA/DAPT.jpg]

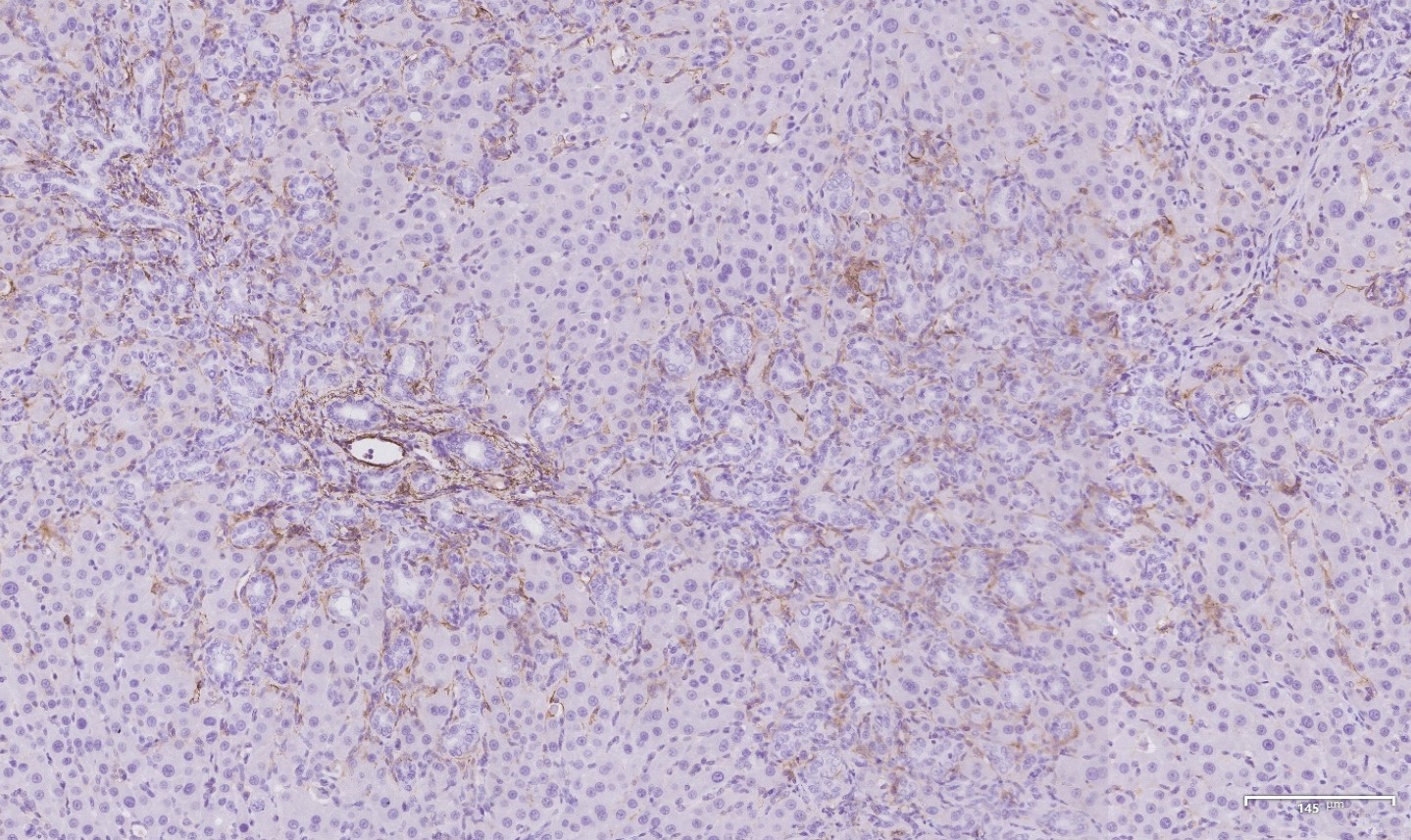

Supplement: Supplementary file 3 [file DataSheet4.ZIP › the original source data of Figures 5-7/Fig. 5/IHC/Fig. 5K (Rat-BDL)/a-SMA/JY5.jpg]

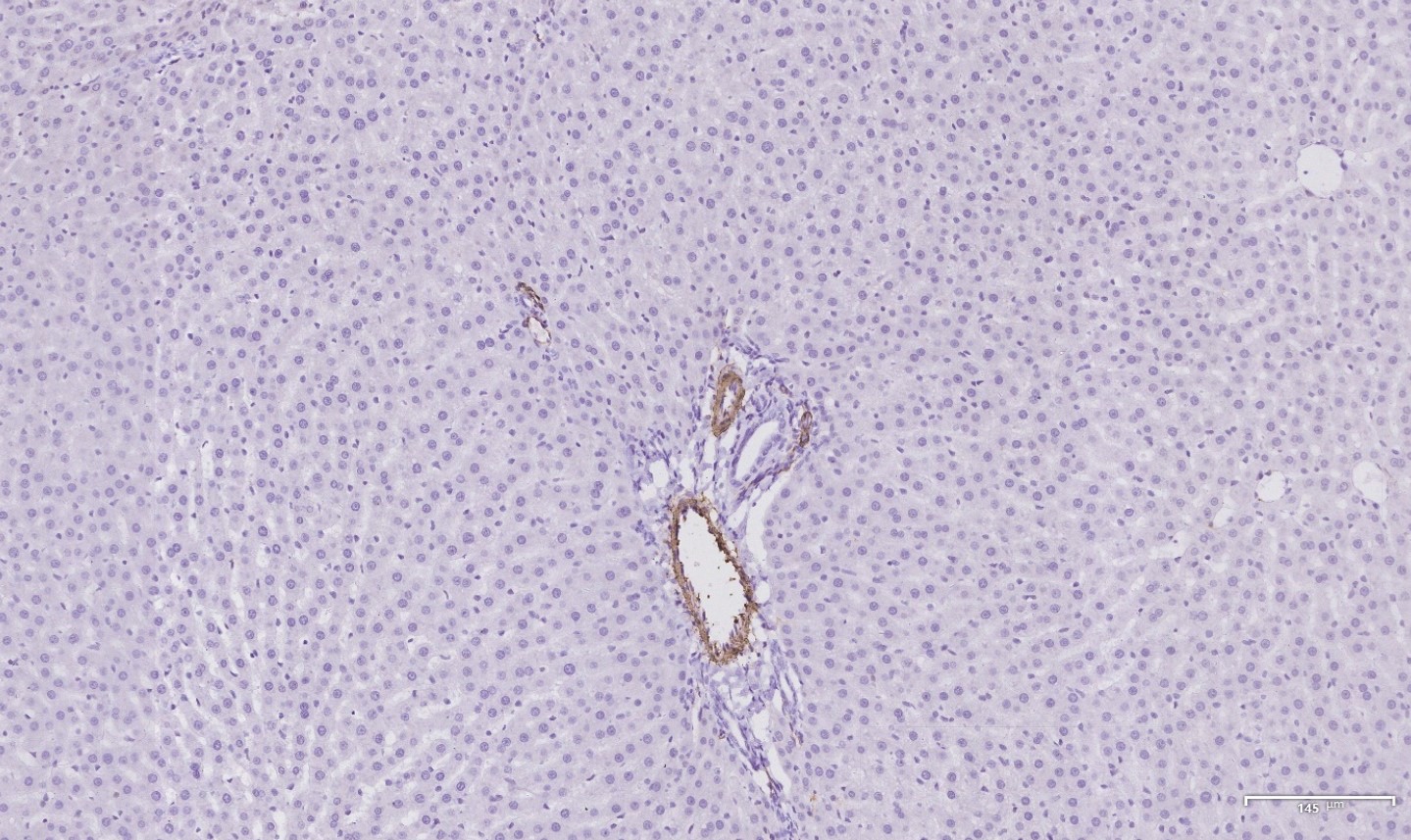

Supplement: Supplementary file 3 [file DataSheet4.ZIP › the original source data of Figures 5-7/Fig. 5/IHC/Fig. 5K (Rat-BDL)/a-SMA/Sham.jpg]

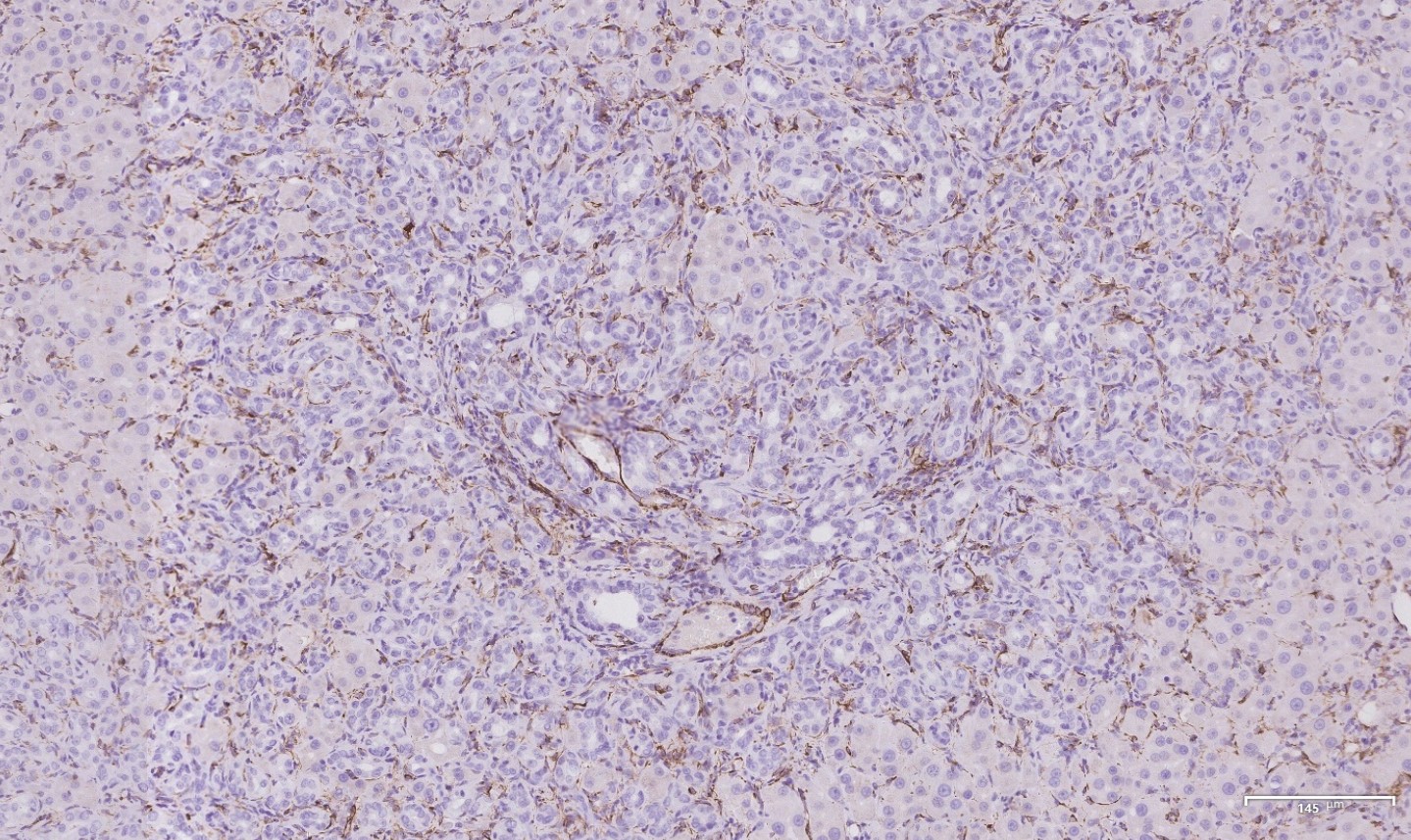

Supplement: Supplementary file 3 [file DataSheet4.ZIP › the original source data of Figures 5-7/Fig. 5/IHC/Fig. 5K (Rat-BDL)/Desmin/BDL.jpg]

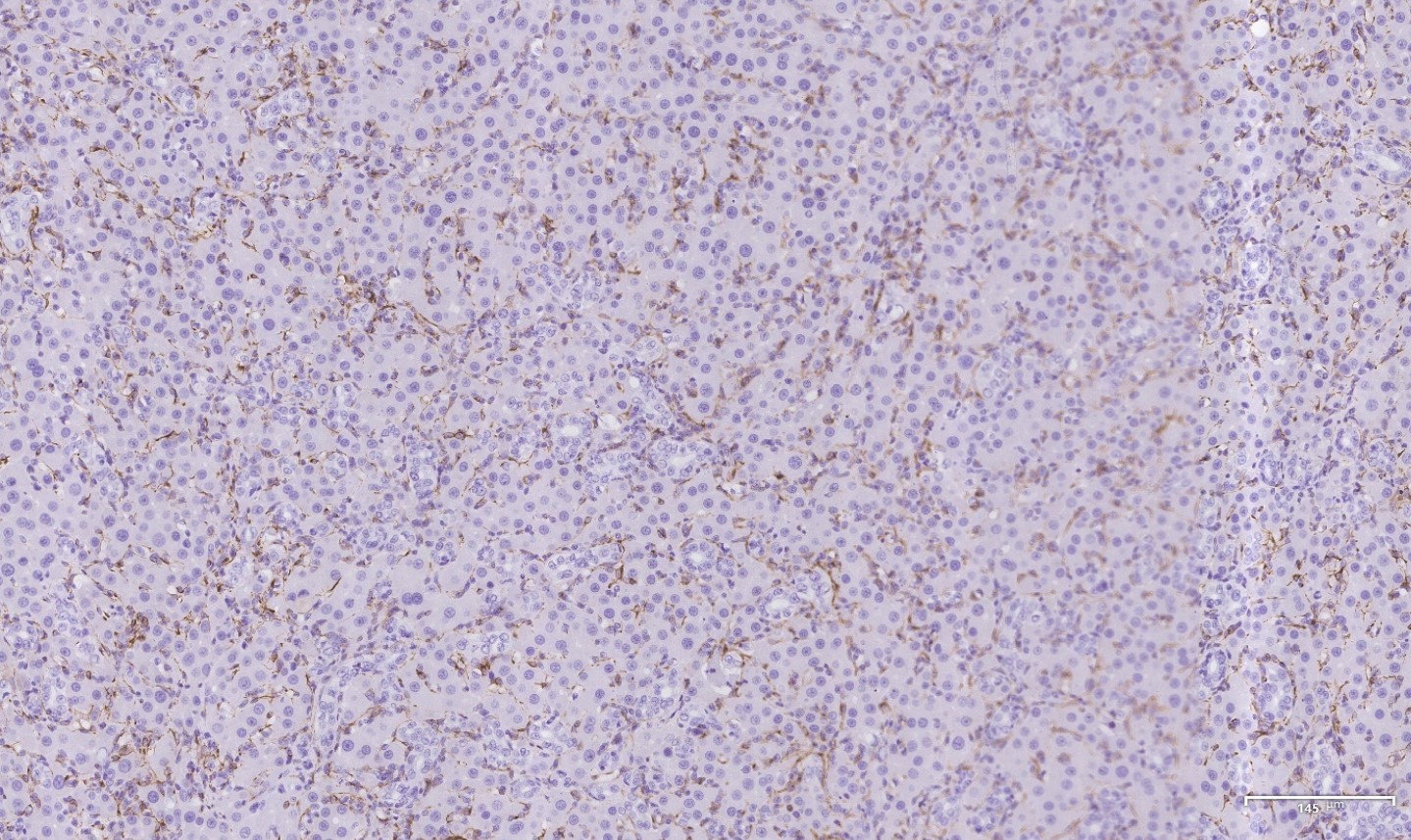

Supplement: Supplementary file 3 [file DataSheet4.ZIP › the original source data of Figures 5-7/Fig. 5/IHC/Fig. 5K (Rat-BDL)/Desmin/DAPT.jpg]

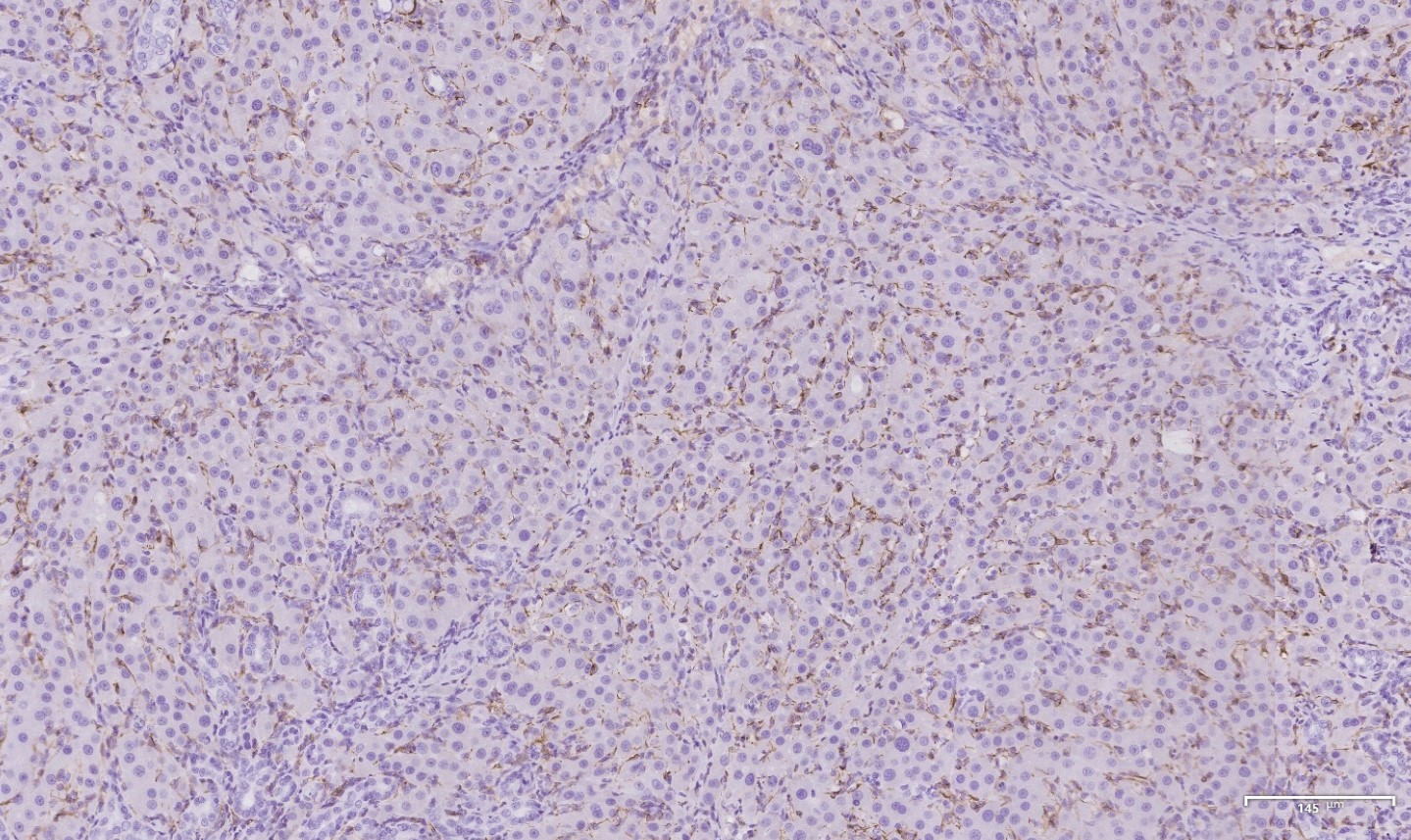

Supplement: Supplementary file 3 [file DataSheet4.ZIP › the original source data of Figures 5-7/Fig. 5/IHC/Fig. 5K (Rat-BDL)/Desmin/JY5.jpg]

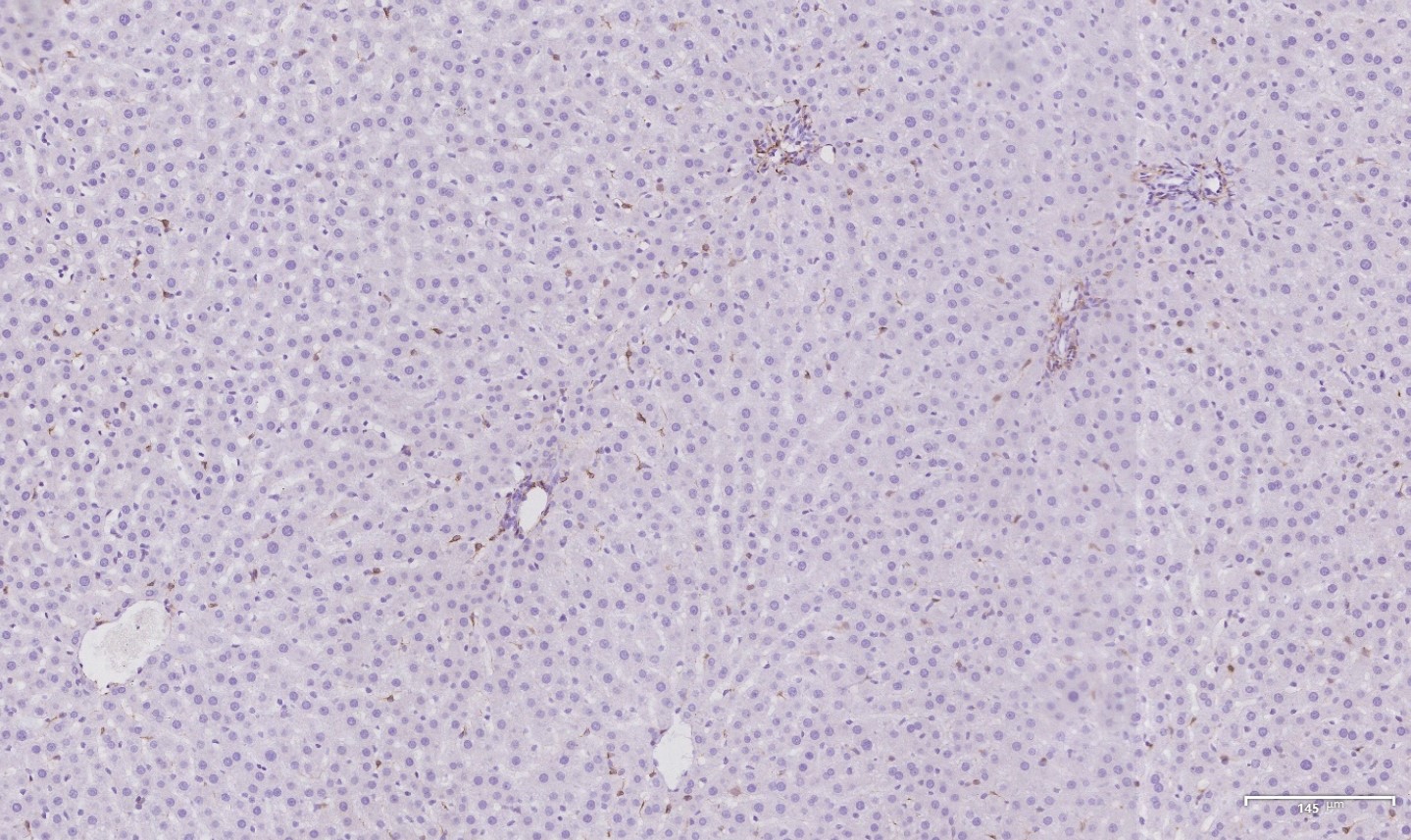

Supplement: Supplementary file 3 [file DataSheet4.ZIP › the original source data of Figures 5-7/Fig. 5/IHC/Fig. 5K (Rat-BDL)/Desmin/Sham.jpg]

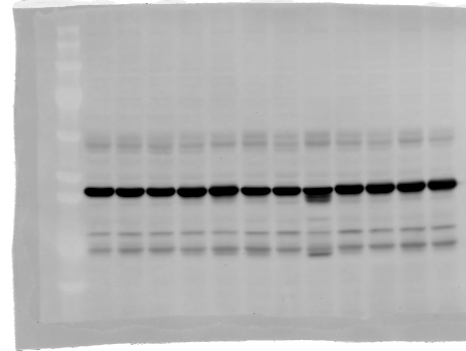

Supplement: Supplementary file 3 [file DataSheet4.ZIP › the original source data of Figures 5-7/Fig. 5/The original image file for the blots/Fig.5E Rat-CCl4-a-SMA-GAPDH.jpg]

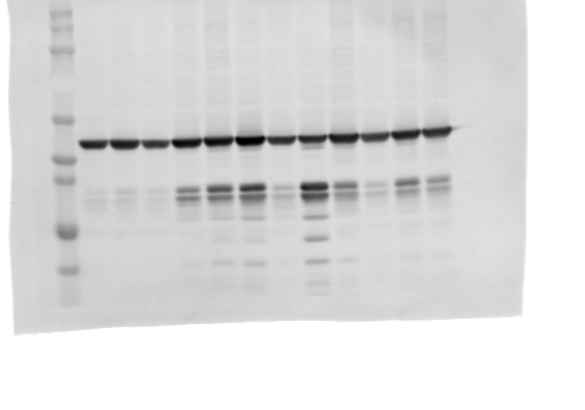

Supplement: Supplementary file 3 [file DataSheet4.ZIP › the original source data of Figures 5-7/Fig. 5/The original image file for the blots/Fig.5E Rat-CCl4-a-SMA.jpg]

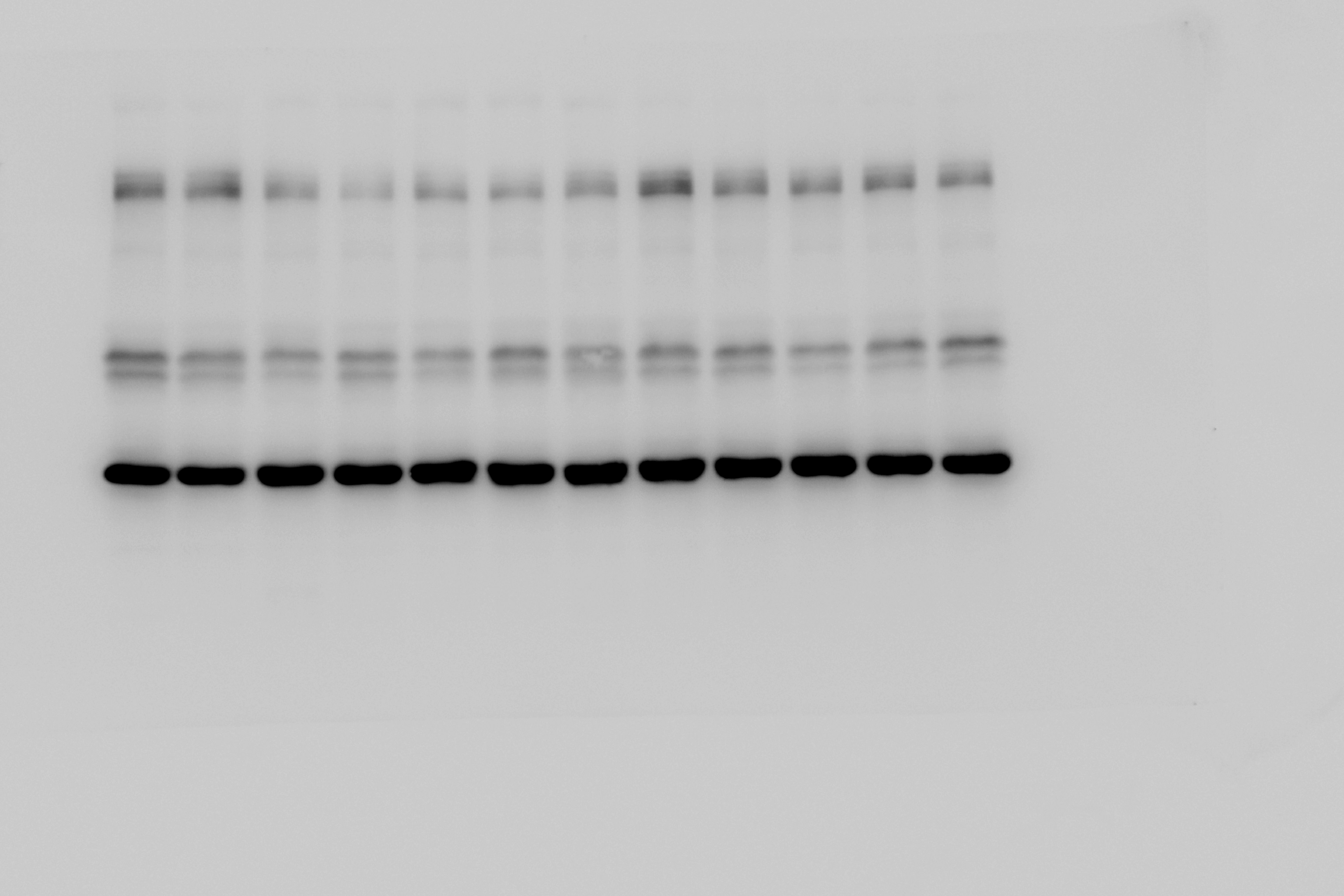

Supplement: Supplementary file 3 [file DataSheet4.ZIP › the original source data of Figures 5-7/Fig. 5/The original image file for the blots/Fig.5J Mice-CCl4-a-SMA-GAPDH.tif]

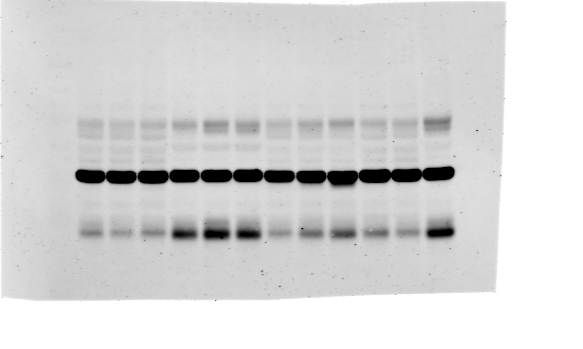

Supplement: Supplementary file 3 [file DataSheet4.ZIP › the original source data of Figures 5-7/Fig. 5/The original image file for the blots/Fig.5O Rat-BDL-a-SMA-GAPDH.jpg]

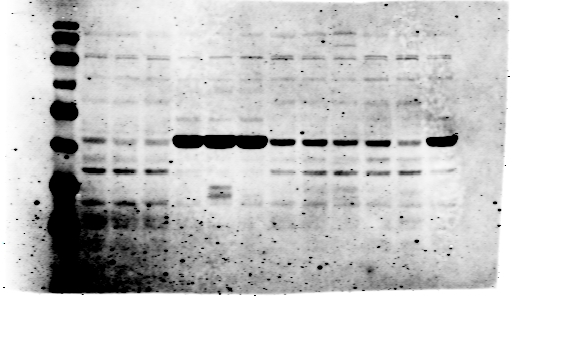

Supplement: Supplementary file 3 [file DataSheet4.ZIP › the original source data of Figures 5-7/Fig. 5/The original image file for the blots/Fig.5O Rat-BDL-a-SMA.jpg]

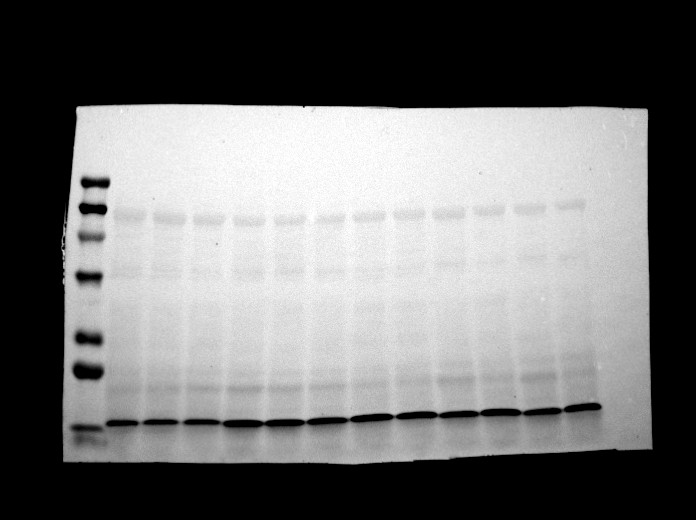

Supplement: Supplementary file 3 [file DataSheet4.ZIP › the original source data of Figures 5-7/Fig. 6/The original image file for the blots/Fig. 6B Rat-CCl4-Jagged1-GAPDH.jpg]

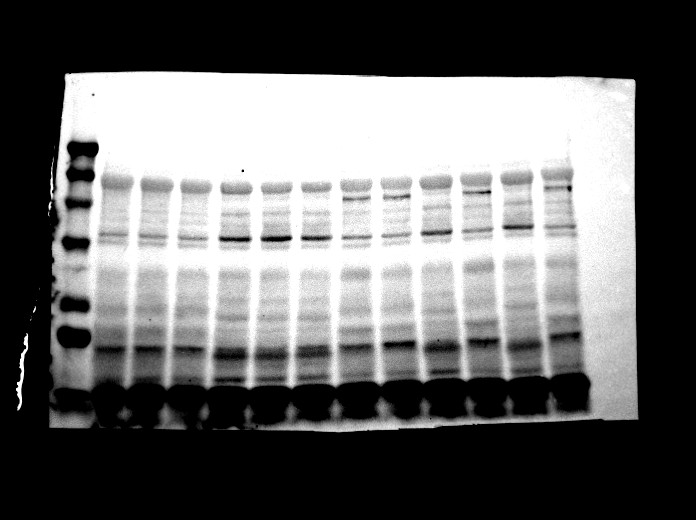

Supplement: Supplementary file 3 [file DataSheet4.ZIP › the original source data of Figures 5-7/Fig. 6/The original image file for the blots/Fig. 6B Rat-CCl4-Jagged1.jpg]

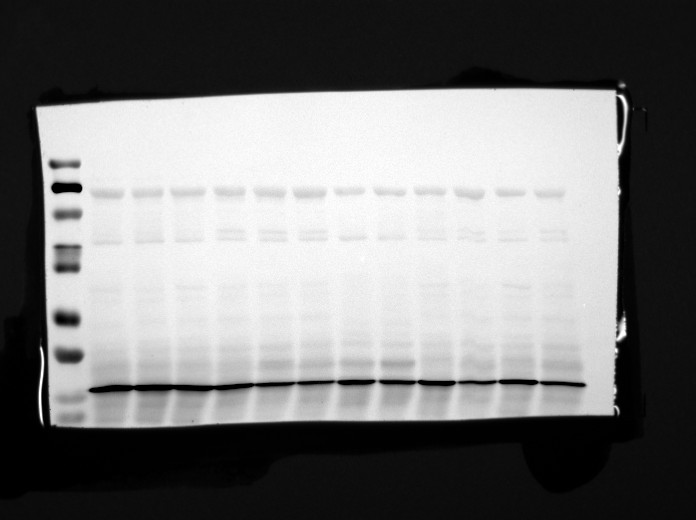

Supplement: Supplementary file 3 [file DataSheet4.ZIP › the original source data of Figures 5-7/Fig. 6/The original image file for the blots/Fig. 6B Rat-CCl4-Notch2-GAPDH.jpg]

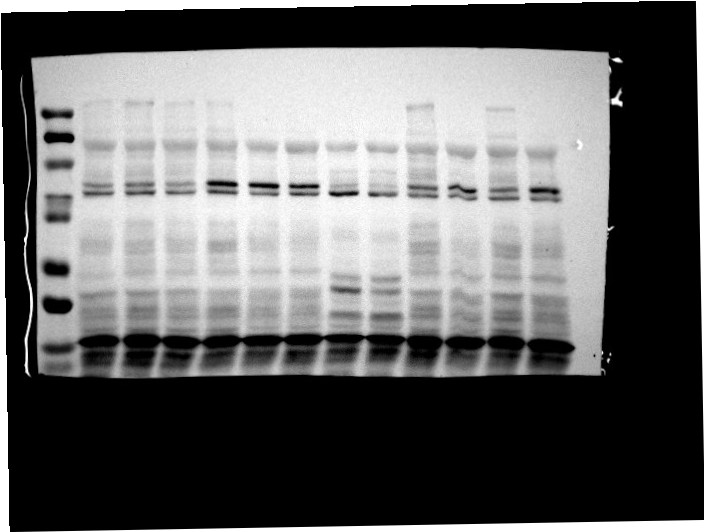

Supplement: Supplementary file 3 [file DataSheet4.ZIP › the original source data of Figures 5-7/Fig. 6/The original image file for the blots/Fig. 6B Rat-CCl4-Notch2.jpg]

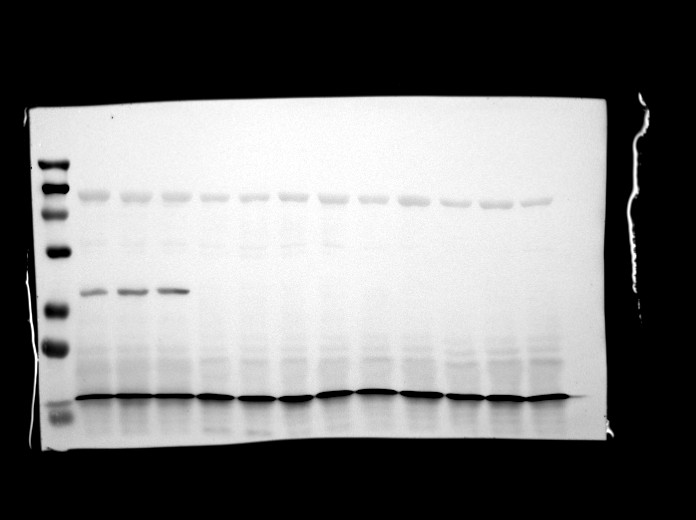

Supplement: Supplementary file 3 [file DataSheet4.ZIP › the original source data of Figures 5-7/Fig. 6/The original image file for the blots/Fig. 6B Rat-CCl4-Notch3-GAPDH.jpg]

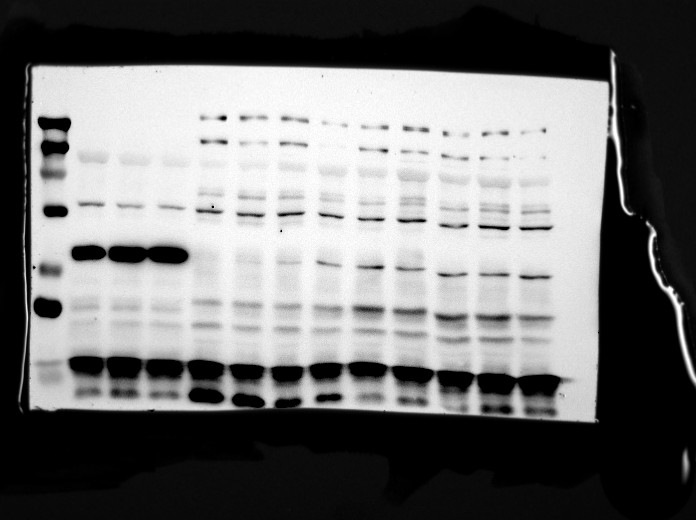

Supplement: Supplementary file 3 [file DataSheet4.ZIP › the original source data of Figures 5-7/Fig. 6/The original image file for the blots/Fig. 6B Rat-CCl4-Notch3.jpg]

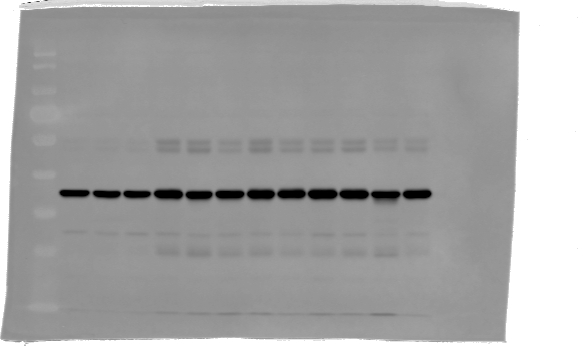

Supplement: Supplementary file 3 [file DataSheet4.ZIP › the original source data of Figures 5-7/Fig. 6/The original image file for the blots/Fig. 6B Rat-CCl4-RBP-kB-GAPDH.jpg]

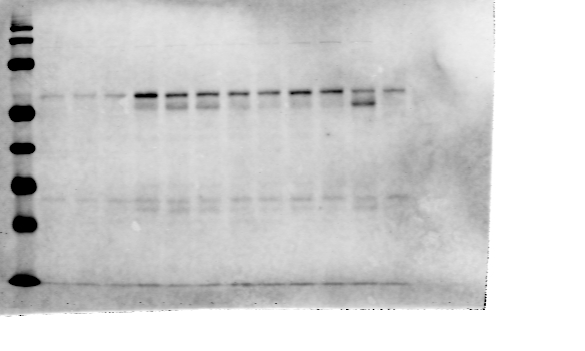

Supplement: Supplementary file 3 [file DataSheet4.ZIP › the original source data of Figures 5-7/Fig. 6/The original image file for the blots/Fig. 6B Rat-CCl4-RBP-kB.jpg]

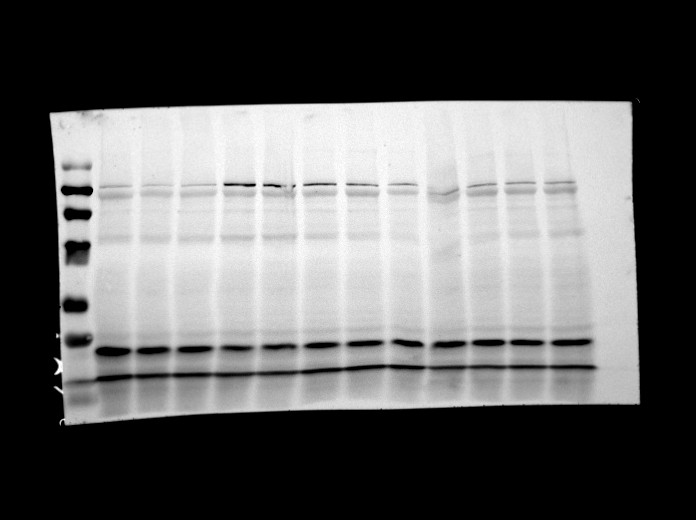

Supplement: Supplementary file 3 [file DataSheet4.ZIP › the original source data of Figures 5-7/Fig. 6/The original image file for the blots/Fig. 6E Mice-CCl4-Jagged1-GAPDH.jpg]

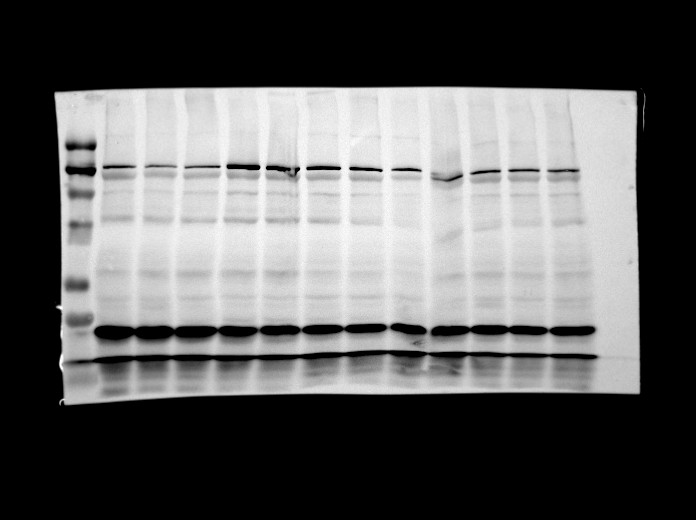

Supplement: Supplementary file 3 [file DataSheet4.ZIP › the original source data of Figures 5-7/Fig. 6/The original image file for the blots/Fig. 6E Mice-CCl4-Jagged1.jpg]

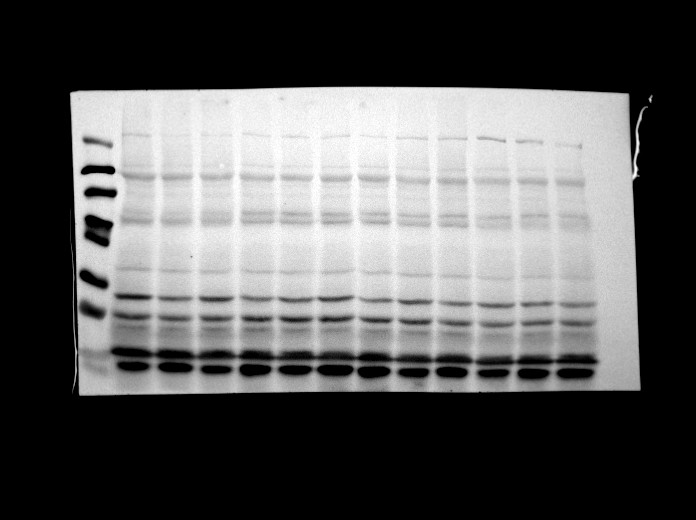

Supplement: Supplementary file 3 [file DataSheet4.ZIP › the original source data of Figures 5-7/Fig. 6/The original image file for the blots/Fig. 6E Mice-CCl4-Notch2-GAPDH.jpg]

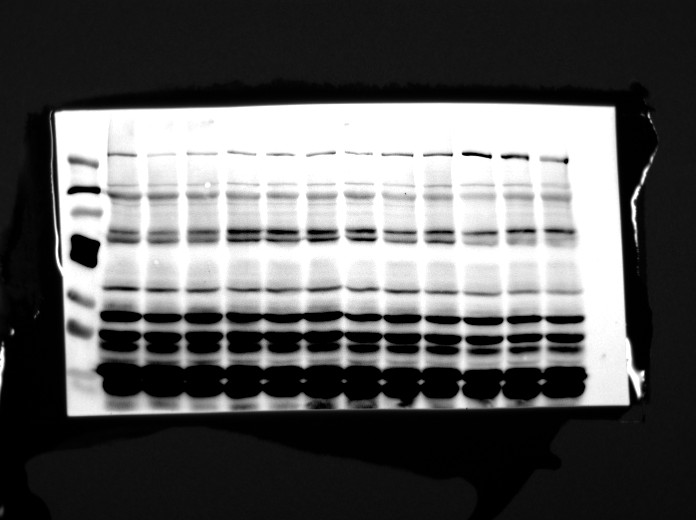

Supplement: Supplementary file 3 [file DataSheet4.ZIP › the original source data of Figures 5-7/Fig. 6/The original image file for the blots/Fig. 6E Mice-CCl4-Notch2.jpg]

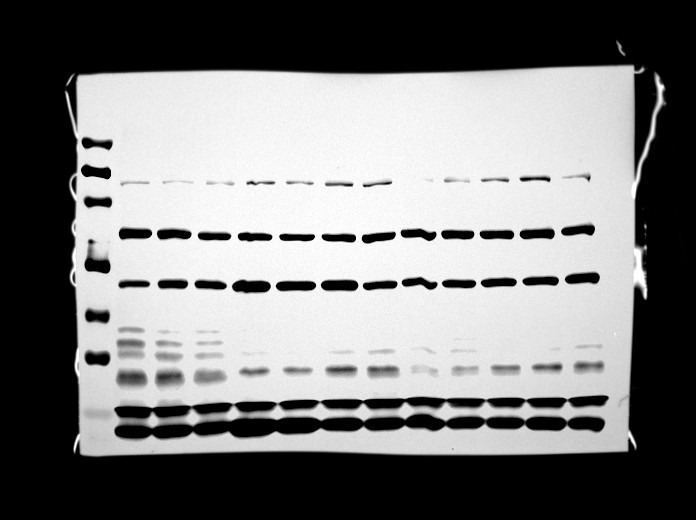

Supplement: Supplementary file 3 [file DataSheet4.ZIP › the original source data of Figures 5-7/Fig. 6/The original image file for the blots/Fig. 6E Mice-CCl4-Notch4-GAPDH.jpg]

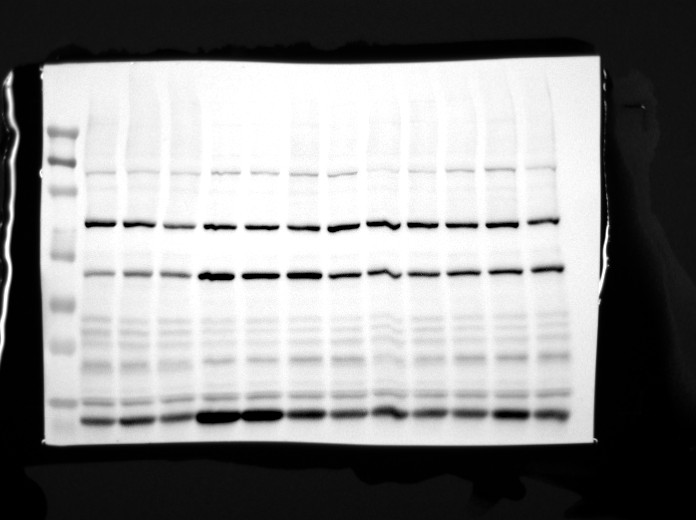

Supplement: Supplementary file 3 [file DataSheet4.ZIP › the original source data of Figures 5-7/Fig. 6/The original image file for the blots/Fig. 6E Mice-CCl4-Notch4.jpg]

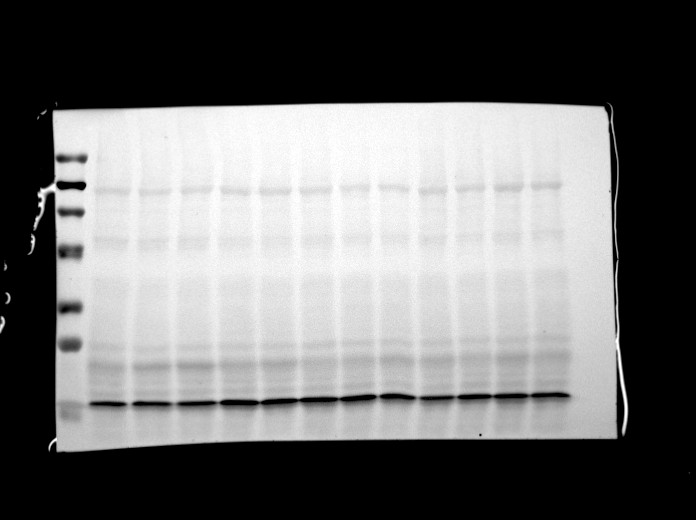

Supplement: Supplementary file 3 [file DataSheet4.ZIP › the original source data of Figures 5-7/Fig. 6/The original image file for the blots/Fig. 6E Mice-CCl4-RBP-kB-GAPDH.jpg]

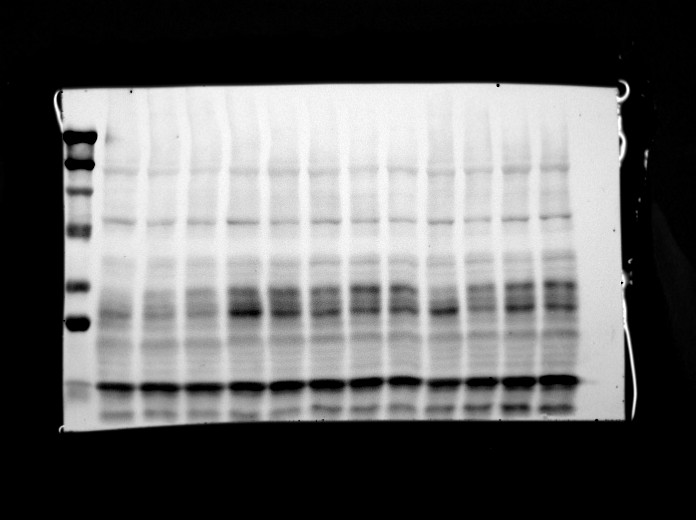

Supplement: Supplementary file 3 [file DataSheet4.ZIP › the original source data of Figures 5-7/Fig. 6/The original image file for the blots/Fig. 6E Mice-CCl4-RBP-kB.jpg]

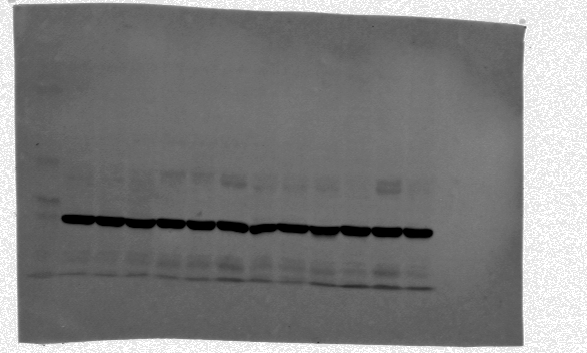

Supplement: Supplementary file 3 [file DataSheet4.ZIP › the original source data of Figures 5-7/Fig. 6/The original image file for the blots/Fig. 6H Rat-BDL-Jagged1-GAPDH.jpg]

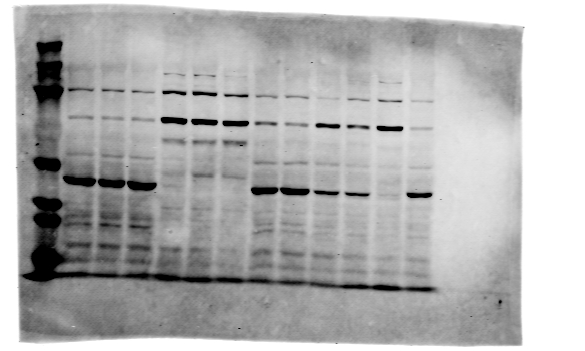

Supplement: Supplementary file 3 [file DataSheet4.ZIP › the original source data of Figures 5-7/Fig. 6/The original image file for the blots/Fig. 6H Rat-BDL-Jagged1.jpg]

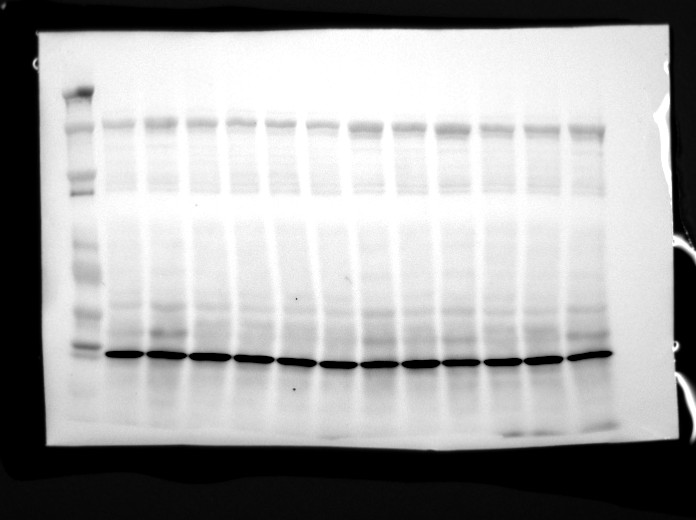

Supplement: Supplementary file 3 [file DataSheet4.ZIP › the original source data of Figures 5-7/Fig. 6/The original image file for the blots/Fig. 6H Rat-BDL-Notch2-GAPDH.jpg]

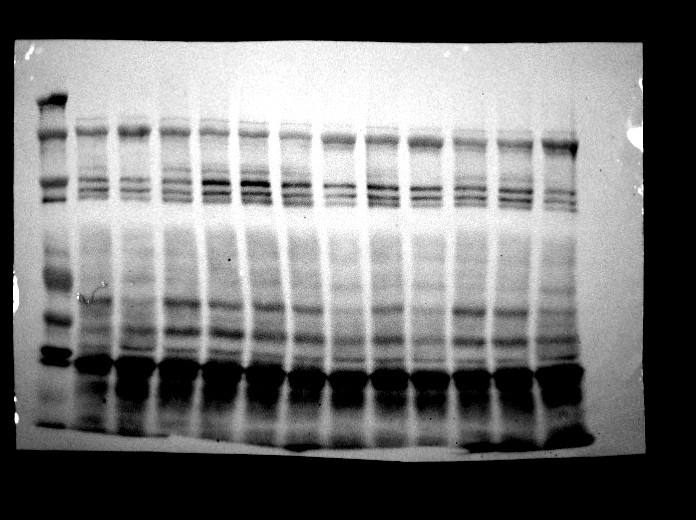

Supplement: Supplementary file 3 [file DataSheet4.ZIP › the original source data of Figures 5-7/Fig. 6/The original image file for the blots/Fig. 6H Rat-BDL-Notch2.jpg]

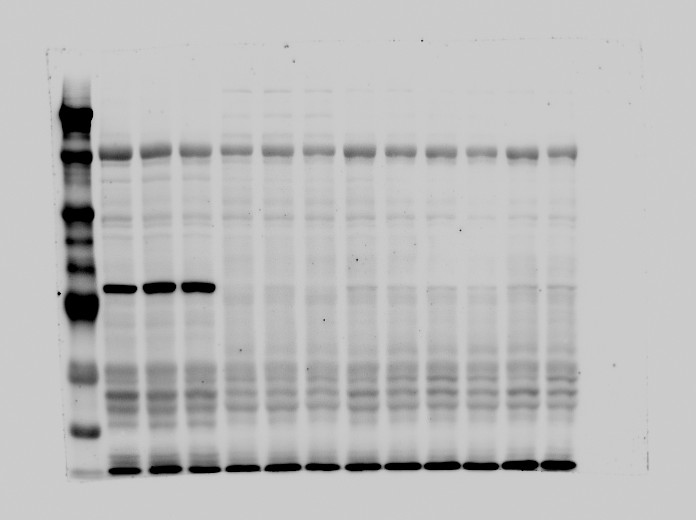

Supplement: Supplementary file 3 [file DataSheet4.ZIP › the original source data of Figures 5-7/Fig. 6/The original image file for the blots/Fig. 6H Rat-BDL-Notch3-GAPDH.jpg]

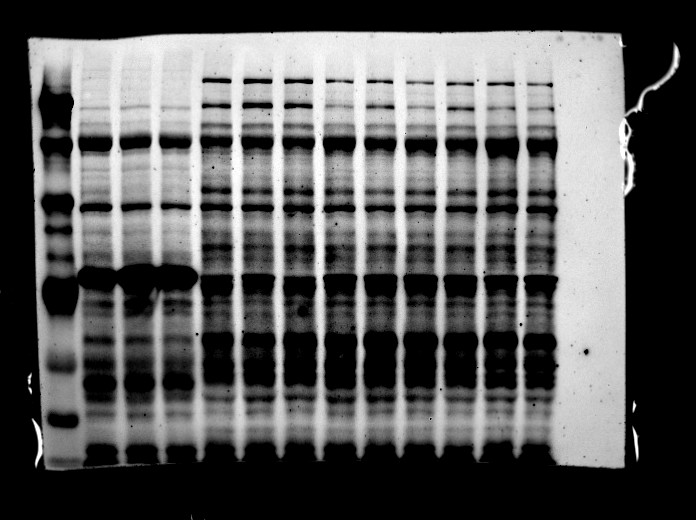

Supplement: Supplementary file 3 [file DataSheet4.ZIP › the original source data of Figures 5-7/Fig. 6/The original image file for the blots/Fig. 6H Rat-BDL-Notch3.jpg]

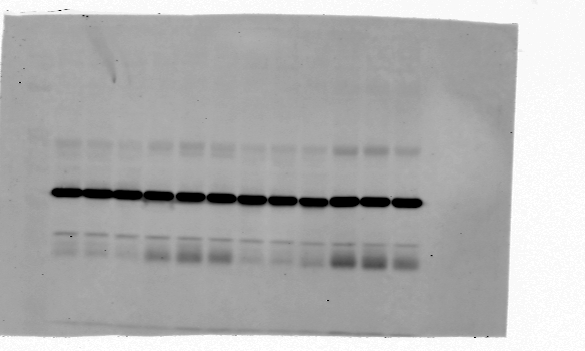

Supplement: Supplementary file 3 [file DataSheet4.ZIP › the original source data of Figures 5-7/Fig. 6/The original image file for the blots/Fig.6H Rat-BDL-RBPkB-GAPDH.jpg]
