# Supplementary material for: The Novel Chinese Medicine JY5 Formula Alleviates Hepatic Fibrosis by Inhibiting the Notch Signaling Pathway
Source: Front Pharmacol. 2021 Sep 22;12:671152. doi: 10.3389/fphar.2021.671152 (PMC8493219; doi:10.3389/fphar.2021.671152)
Supplement: Supplementary file 2 [file Presentation1.pdf]

# *Supplementary Material*

## 1. Supplementary Materials and Methods

### 1.1. Pharmacokinetic study of FZHY decoction in rats

Before the experiment, the rats were fasted overnight but with free access to water. After oral administration of FZHY decoction at 20g raw drug /kg, the rats (n=5 for each time point) were anesthetized and sampled at 0.167 h, 0.5 h, 1 h, 2 h, 4 h, 6 h, 8 h, 11 h, 24 h and 48 h (1.4 g/kg). The hepatic portal vein blood (2 mL) and systemic blood (6-8 mL) were obtained from the portal vein and abdominal aorta, respectively, and the right liver lobe was excised. After centrifuged for 5 min at 10000 rpm, the plasma was obtained. After acetonitrile precipitation, the processed plasma and liver samples were injected to the mass instrument for analysis.

### 1.2. Instrumentation

The operating parameters were set as follows: spray voltage of 2.5 kv, capillary temperature of 320 °C, source temperature of 350 °C, sheath gas of 35 arbitrary units, auxiliary air of 12 arbitrary units and capillary temperature of 320 °C. The analysis was conducted in full single ion monitoring (SIM) mode. The mass range of m/z 100-1200 was at 1.8-6.35 min in the negative mode and the quantitative ions included 717.14612 for salvianolic acid B [M-H]-, 197.04555 for salvianic acid [M-H]-, 493.11403 for salvianolic acid A [M-H]-, 343.15510 for rosmarinic acid [M-H]-, 1045.55889 for gypenoside XLIX [M-H]-, 1077.58511 for ginsenoside Rb3 [M-H]-. The mass range of m/z 150-400 was at 0-1.8min in the positive mode, and the quantitative ions (m/z) included 494.16567 for amygdalin [M+H]+, 537.21187 for schisantherin A [M+H]+, 433.22209 for schisandrol A [M+H]+, 417.19079 for schisandrol B [M+H]+, 417.22717 for deoxyschizandrin [M+H]+, 401.19587 for schisandrin B [M+H]+, 277.08591 for tanshinone [M+H]+, 297.14851 for cryptotanshinone [M+H]+, 268.10399 adenosine for [M+H]+, 252.10907 for cordycepin [M+H]+. Xcalibur 2.1 workstation was applied for the data analysis and system operation. Chromatographic separation was carried out on an Acquity UPLC BEH column (100 mm × 1.0 mm, 1.7μm, Thermo Scientific, US) at 40 °C. The sample injection and flow rate were set at 5μL and 0.3mL/min, respectively. The mobile phases consisting of solvent A (acetonitrile with 0.1% formic acid) and solvent B (water with 0.1% formic acid) were utilized the following gradient program: 0~1.5min，7% A；1.5~2min，7%~30% A；2~3min，30%~50% A；3~4min，50% A；4~4.5min，50%~70% A；4.5~5.5min，70% A; 5.5~6min，70%~90% A; 6~9min，90% A; 9~9.1min，90%~7% A; 9.1~10min，7% A.

### 1.3. Experimental liver fibrosis models

$CCl_4$-induced liver fibrosis rat model: Wistar rats were randomly divided into the control (Oil) group (n=8) and the $CCl_4$ group (n=24). The model group rats were injected subcutaneously with 50% $CCl_4$ solution in olive oil at a 1mL/kg dose, twice a week for 9 weeks. The control rats were injected subcutaneously with equivalent volume olive oil at a 1mL/kg dose. At the first day of the 7$^{th}$ week, the $CCl_4$ rats were randomly divided into 3 groups: the $CCl_4$ group (n=8), the $CCl_4$ plus JY5 group (n=8) and the $CCl_4$ plus SORA group (n=8). Rats were respectively administered intragastrically with JY5 (salvianolic acid B: 16mg/kg, amygdalin: 0.5mg/kg,

schisantherin A: 2mg/kg) and SORA (5mg/kg) added to 0.3% CMC-Na suspension at a 10mL/kg dose in drug-treated groups, once a day for 3 weeks. In the control group and $CCl_4$ group, rats were administered intragastrically with equivalent volume 0.3% CMC-Na suspension at a 10mL/kg dose.

BDL-induced liver fibrosis rat model: SD rats were randomly divided into the control (Sham) group (n=8) and the BDL group (n=24). BDL surgery was performed as previously described (Zhang et al., 2017). At the first day of the second week after BDL, the BDL rats were randomly divided into 3 groups: the BDL group (n=8), the JY5 group (n=8) and the DAPT group (n=8). Rats were administered intragastrically with JY5 (salvianolic acid B: 16mg/kg, amygdalin: 0.5mg/kg, schisantherin A: 2mg/kg) added to 0.3% CMC-Na suspension at a 10mL/kg dose in the JY5 group. The DAPT group rats were injected intraperitoneally with DAPT (30mg/kg) dissolved in DMSO at a dose of 30mg/kg. In Sham group and BDL group, rats were administered intragastrically with equivalent volume 0.3% CMC-Na suspension at a 10mL/kg dose. The rats were treated with drugs once a day for three weeks.

$CCl_4$-induced liver fibrosis mice model: C57/ BL6 mice were randomly divided into the control (Oil) group (n=8) and the $CCl_4$ group (n=24). The $CCl_4$ mice were injected intraperitoneally with 15% $CCl_4$ solution in olive oil at a 2mL/kg dose, three times a week for 6 weeks. The control mice were injected intraperitoneally with equivalent volume olive oil at a 2mL/kg dose. At the first day of the $4^{th}$ week, the $CCl_4$ mice were randomly divided into 3 groups: the $CCl_4$ group (n=8), the $CCl_4$ plus JY5 group (n=8) and the $CCl_4$ plus SORA group (n=8). Mice were respectively administered intragastrically with JY5 (salvianolic acid B: 22.4mg/kg, amygdalin: 0.7mg/kg, schisantherin A: 2.8mg/kg) and SORA (7mg/kg) added to 0.3% CMC-Na suspension at a 10mL/kg dose in drug-treated groups, once a day for 3 weeks. In the control group and $CCl_4$ group, mice were administered intragastrically with equivalent volume 0.3% CMC-Na suspension at a 10mL/kg dose.

All rats and mice were anaesthetized under sodium pentobarbital i.p. injection, then blood and liver samples were collected, and stored at −80°C for subsequent testing. Partial liver tissues were fixed in 10% neutral-buffered formalin for pathological analysis.

### 1.4. Histopathological and immunohistochemical analysis

According to corresponding standard protocol, liver sections were stained with Hematoxylin & Eosin (H&E, lot. 20161225, NJBI, Nanjing, China) and Sirius Red (SR). All slides were scanned using the Leica SCN400 slide scanner (Leica Microsystems Ltd., Mannheim, Germany). The whole SR-stained liver sections were analyzed using ImageScope software to calculate the percentage of collagen positive areas (Percent Total Positive × Total Stained Area / Total Analysis Area), which could semi-quantitatively evaluate liver fibrosis better.

IHC staining was performed using the GTVision TM III Detection Kit with peroxidase/DAB, rabbit/mouse (Cat No. GK500705, Gene Tech Co., Ltd, Shanghai, China). Paraffin-embedded sections were dewaxed and rehydrated, subsequently subjected to heat-mediated antigen retrieval in 0.01M sodium citrate buffer, and rinsed in diH2O prior to staining. After cooling to room temperature, the sections were incubated in 3% $H_2O_2$-methanol mixture solution for 10 minutes to block endogenous peroxidase. Blocking the samples was performed by incubation with 10% goat serum at room temperature for 30 min, before incubation with primary antibody overnight at

4°C in a wet box. The next day, incubation with the second antibody and chromogen detection was performed using the DAB chromogen by light microscopy. After nuclear counterstaining by hematoxylin, the sections were differentiated, dehydrated and transparentized. Finally, the sections were sealed with neutral gum and scanned using the Leica SCN400 slide scanner to further analysis.

### 1.5. Luciferase reporter assay

The transcriptional activity of Notch was measured using RBP-κB luciferase reporter plasmid constructed by Shanghai Jikai Gene Chemical Technology Co. Ltd. following the supplier's instructions for use. The transiently co-transfected LX-2 cells with firefly luciferase and renilla luciferase plasmid using Lip8000TM (C0533, Beyotime Biotechnology, China) were treated with TGF-β1(5ng/mL), and simultaneously treated with salvianolic acid B (32μM), amygdalin (1μM), schisantherin A (4μM) or JY5 (37μM) for 24 hours, respectively. RBP-κB luciferase activity was detected by Dual-Lumi™ luciferase reporter gene assay kit (RG088S, Beyotime Biotechnology, China) following the manufacturer's instructions. With renilla luciferase a the internal control in each transfection, the relative luciferase activity was calculated as the ratio of firefly-to-renilla luciferase activity.

## 2. Supplementary Figures and Tables

### 2.1 Supplementary Figures

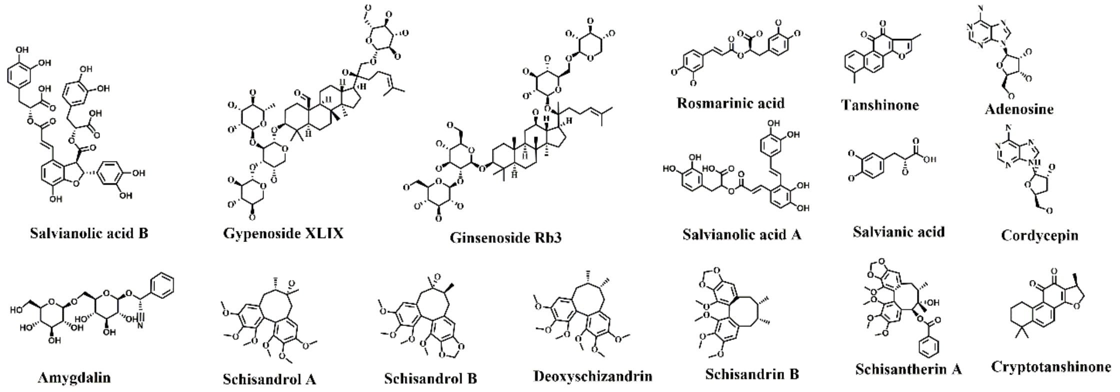


**Supplementary Figure 1. The chemicals structure of 16 compounds in FZHY decoction (2g/mL).**

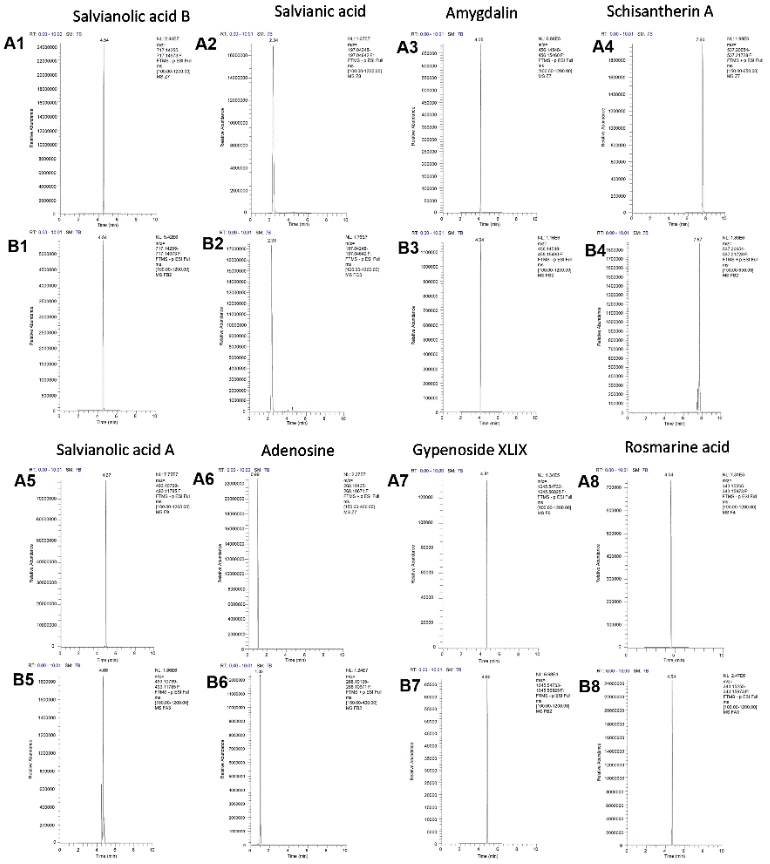
Salvianolic acid B
Salvianic acid
Amygdalin
Schisantherin A
A1
A2
A3
A4
B1
B2
B3
B4
Salvianolic acid A
Adenosine
Gypenoside XLIX
Rosmarine acid
A5
A6
A7
A8
B5
B6
B7
B8

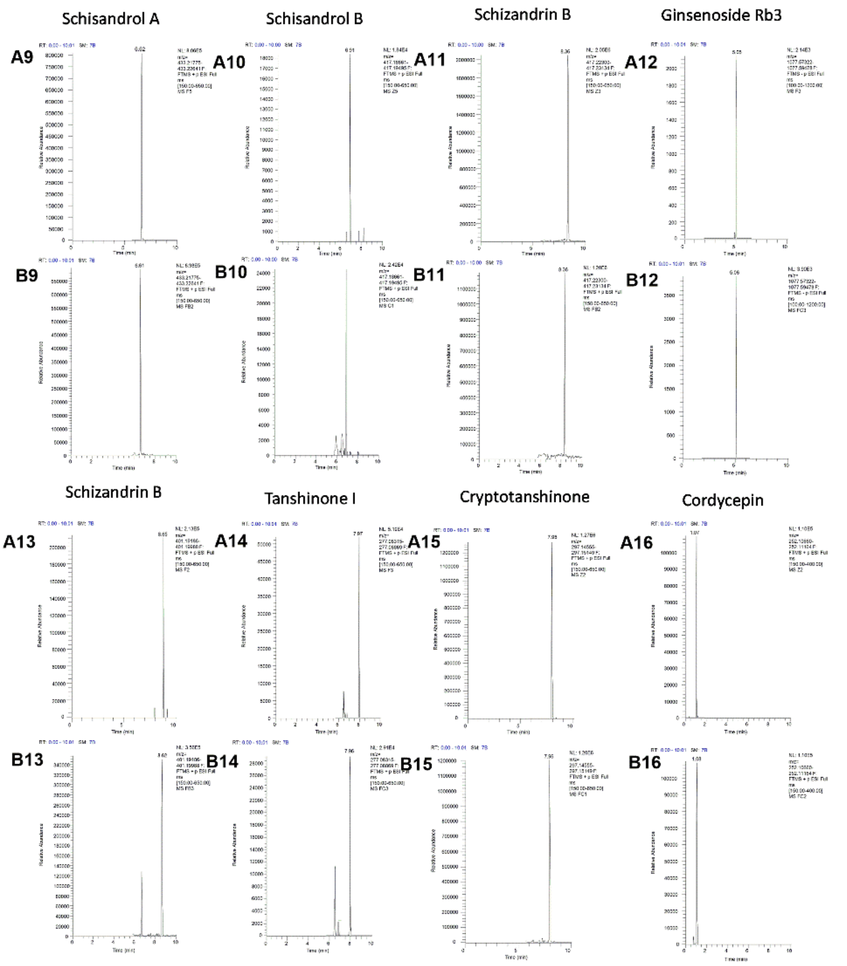


**Supplementary Figure 2. The SIM chromatograms of 16 constituents in the standard curves (A) and FZHY decoction by UPLC- Q- trap mass.**

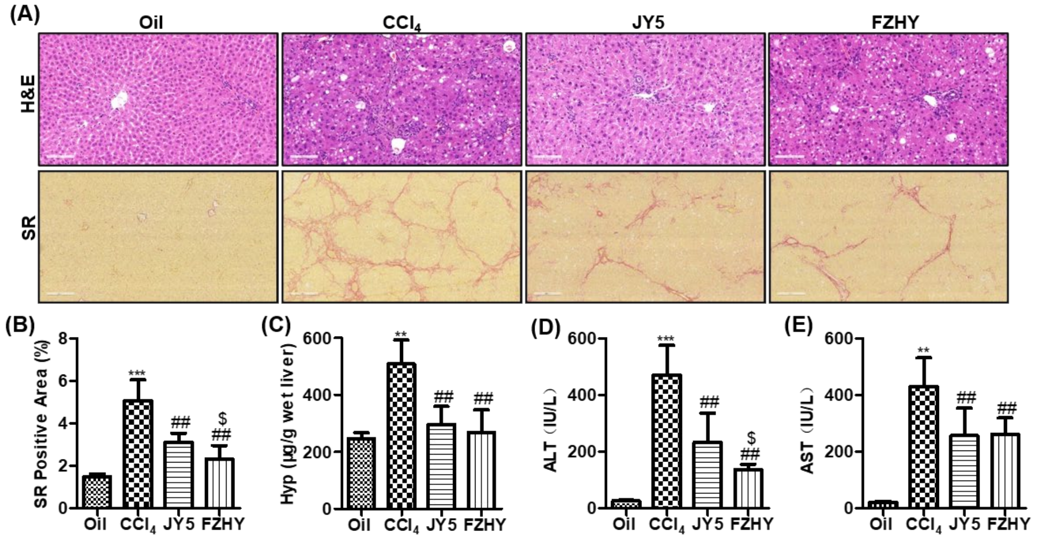


**Supplementary Figure 3. JY5 and FZHY significantly alleviates hepatic injury and collagen deposition in $CCl_4$-induced rat liver fibrosis.** H&E (100×) and SR (100×) staining(**A**), and semi-quantitative analysis (**B**) of collagen disposition (%) in

SR-stained liver sections. (**C**). Hydroxyproline content in wet liver tissue was detected. (**D, E**) The levels of serum ALT and AST were measured. **p* < 0.05, ***p*<0.01, and ****p*<0.001 vs. the control group, $^{\#\#}p<0.01$ vs. the $CCl_4$ group, $^{\$}p < 0.05$ vs. the FZHY treated group. Oil, control group.

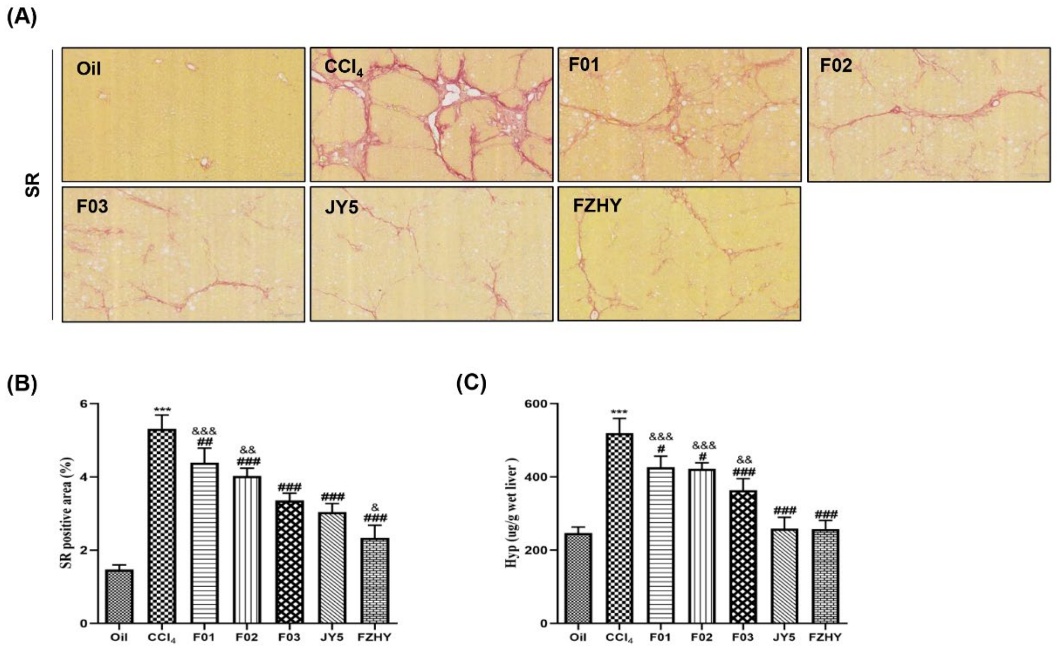


**Supplementary Figure 4. JY5 significantly alleviates liver tissue collagen deposition and Hyp content in $CCl_4$-induced rat liver fibrosis.** SR (100×) staining(**A**), and semi-quantitative analysis (**B**) of collagen disposition (%) in SR-stained liver sections. (**C**). Hyp content in wet liver tissue was detected. ****p*<0.001 vs. the control group, $^{\#}p<0.05$, $^{\#\#}p<0.01$ and $^{\#\#\#}p<0.001$ vs. the $CCl_4$ group, $^{\&}p < 0.05$, $^{\&\&}p < 0.01$ and $^{\&\&\&}p < 0.001$ vs. the JY5 treated group. Oil, control group; F01, salvianolic acid B; F02, amygdalin; F03, schisantherin A.

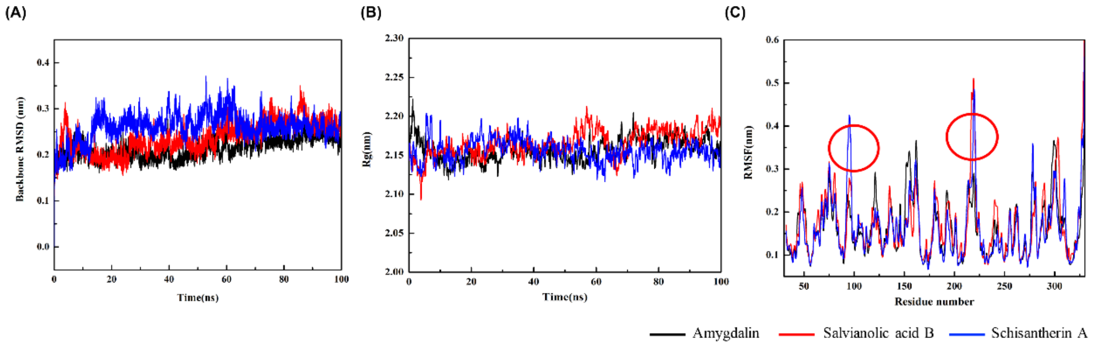


**Supplementary Figure 5. Molecular dynamics simulation findings.** (A) Stability analysis as inferred by RMSD. (B) Compactness of the protein from Rg. (C) Fluctuations rendered by the RMSF plots.

## 2.2 Supplementary Tables

## Supplementary Table 1. The antibodies used in this study.

| Antibody | Species | Company | Cat No | Concentration |
|---|---|---|---|---|
| Col-I | Rabbit pAb | abcam | ab34710 | IHC 1:200 |
| Col-IV | Rabbit pAb | abcam | ab6586 | IHC 1:250 |
| α-SMA | Rabbit pAb | abcam | ab5694 | IHC 1:200 |
| | | | | WB 1:1000 |
| Desmin | Rabbit pAb | abcam | ab15200 | IHC 1:400 |
| Jagged1 | Rabbit pAb | Epitomics | Epit3772S | WB 1:1000 |
| Notch2 | Rabbit mAb | CST | #4530S | WB 1:1000 |
| Notch3 | Rabbit pAb | ABclonal | A13522 | WB 1:1000 |
| Notch4 | Rabbit pAb | ABclonal | A8303 | WB 1:1000 |
| RBP-κB | Rabbit mAb | CST | #5313 | WB 1:1000 |
| GAPDH | Mouse mAb | Proteintech | 60004-1-Ig | WB 1:5000 |
| Anti-rabbit IgG | Goat | CST | #5366 | WB 1:10000 |
| Anti-mouse IgG | Goat | CST | #5257 | WB 1:10000 |

## Supplementary Table 2. Primers used in this study.

| Name | Rat (5’→3’) | Mouse (5’→3’) | Human (5’→3’) |
|---|---|---|---|
| α-SMA | | | |
| Forward | GAGGAGCATCCGACCTTGC | GTCCCAGACATCAGGGAGTAA | TGCAACATGGAAGGTATTGC |
| Reverse | TTTCTCCCGGTTGGCCTTA | TCGGATACTTCAGCGTCAGGA | TTCACACAGCACCAAGC |
| Col-I | | | |
| Forward | GCCTCCCAGAACATCACCTA | GCTCCTCTTAGGGGCCACT | CCAAGGTGGGATCGTGAGG |
| Reverse | CCTTCTTGAGGTTGCCAGTC | CCACGTCTCACCATTGGGG | TCGGAAGGATAAAACGCGGTC |
| Notch2 | | | |
| Forward | GAGGAAGAAGTGTCTCAA | CCACCTGCAATGACTTCATCGG | CCCAATGGGCAAGAAGTCTA |
| Reverse | GTGGCATCAGAAACATAT G | TCGATGCAGGTGCCTCCATTCT | CACAATGTGGTGGTGGTGGGATA |
| Notch3 | | | |
| Forward | GACAAGGACCACTCCCACTACT | GACTGCTCACTGAACGTGGA | GCATAGGCCAGTTCACCTGT |
| Reverse | ATCCACATCATCCTCACAACTG | CACACCGGCTGTTGTTGAAG | AATGTCCACCTCGCAATAGG |
| Notch4 | | | |
| Forward | TGTCAGGAACCAGTGTCAGAA C | CTCTTGCCACTCAATTTCCCT | TCTCCGGCACCCGATGT |
| Reverse | CCTGGGCTTCACATTCATCTAT | TTGCAGAGTTGGGTATCCCTG | TCAAAGCCTGGGAGACACTTG |
| Jagged1 | | | |
| Forward | CCATCAAGGATTATGAGAAC | CCTCGGGTCAGTTTGAGCTG | CAACCGTGCCAGTGACTATTTCTGC |
| Reverse | TGGTGCTTATCCATATCA | CCTTGAGGCACACTTTGAAGTA | TGTTCCCGTGAAGCCTTTGTTACAG |
| Jagged2 | | | |
| Forward | AAATGAGTGGTCCGTGGCAGA | CAATGACACCACTCCAGATGAG | AACGATACCCCGAATGAGG |
| Reverse | TGGTTGGAAGCCTTGTCTGCT | GGCCAAAGAAGTCGTTGCG | GCTGCCACAGTAGTTCAGGTCTTTG |
| Delta-like 1 | | | |
| Forward | GTGTGCAGATGGTCCTTGCTTC | GCTGGAAGTAGATGAGTGTGCTC | TCCTGATGACCTCGCAACAGA |
| Reverse | CTGACATCGGCACAGGTAGGAG | CACAGACCTTGCCATAGAAGCC | ACACACGAAGCGGTAGGAGT |
| Delta-like 3 | | | |
| Forward | CTGAGGTTACAAGACGGTGCT | CTGGTGTCTTCGAGCTACAAAT | CACTCCCGGATGCACTCAAC |
| Reverse | GTAAATGGAAGGGGCTGGTATG | TGCTCCGTATAGACCGGGAC | CCCGAGCGTAGATGGAAGGA |

| | | | |
|---|---|---|---|
| Delta-like 4 | | | |
| Forward | GCAGAACCACACACTGGACTAT | TTCCAGGCAACCTTCTCCGA | GACCACTTCGGCCACTATGT |
| Reverse | TGGCACCTTCTCTCCTAAACTC | ACTGCCGCTATTCTTGTCCC | CCTGTCCACTTTCTTCTCGC |
| RBP κB | | | |
| Forward | TTGCTTACCTTCAGGCGTGTG | TGGCTACATCCATTACGGGCAG | TCATGCCAGTTCACAGCAGTGG |
| Reverse | GCCCAATGAGTCTGCTGCAA | GTGGAGTTGTGATACAGGGTCG | TGGATGTAGCCATCTCGGACTG |
| GAPDH | | | |
| Forward | GGCACAGTCAAGGCTGAGAATG | AAGGTCATCCATGACAACTTTGGC | GAAATCCCATCACCATCTTCCAGG |
| Reverse | ATGGTGGTGAAGACGCCAGTA | ACAGTCTTCTGGGTGGCAGTGAT | GAGCCCCAGCCTTCTCCATG |

**Supplementary Table 3. The content and source of 16 compounds in FZHY decoction.**

| Compounds | Source | Concentration (μg/mL) |
|---|---|---|
| Salvianolic acid B | *Salvia miltiorrhiza* | 3067.86 |
| Danshensu | *Salvia miltiorrhiza* | 1939.75 |
| Amygdain | *Peach kernel* | 1926.57 |
| Schisantherin A | *Schisandra chinensis* | 1400.61 |
| Salvianolic acid A | *Salvia miltiorrhiza* | 1399.69 |
| Adenosine | *Cordyceps mycelium* | 855.73 |
| Gypenoside XLIX | *Gynostemma pentaphylla* | 346.19 |
| Rosmarinic acid | *Salvia miltiorrhiza* | 276.23 |
| Schisandrol A | *Schisandra chinensis* | 144.88 |
| Schisandrol B | *Schisandra chinensis* | 70.95 |
| Deoxyschizandrin | *Schisandra chinensis* | 8.65 |
| Ginsenoside Rb3 | *Gynostemma pentaphylla* | 4.66 |
| Schisandrin B | *Schisandra chinensis* | 4.59 |
| Tanshinone I | *Salvia miltiorrhiza* | 2.93 |
| Cryptotanshinone | *Salvia miltiorrhiza* | 1.39 |
| Cordycepin | *Cordyceps mycelium* | 1.21 |

**Supplementary Table 4. The related information of components docked with key targets.**

| **Protein** | **Component** | **Binding energy (kcal/mol)** |
|---|---|---|
| Jagged1 | Salvianolic acid B | 1.16 |
| Jagged1 | Amygdalin | -0.64 |
| Jagged1 | Schisantherin A | -2.88 |
| Notch2 | Salvianolic acid B | 0.19 |
| Notch2 | Amygdalin | -1.8 |
| Notch2 | Schisantherin A | -3.32 |
| RBP-κB | Salvianolic acid B | -5.7 |
| RBP-κB | Amygdalin | -3.84 |
| RBP-κB | Schisantherin A | -6.7 |

The binding energy refers to the strength of the binding between the receptor and the ligand; the lower the binding energy, the more stable the docking module.

## References

Zhang, X., Xu, Y., Chen, J., Liu, C., Du, G., Zhang, H., et al. (2017). Huang Qi Decoction Prevents BDL-Induced Liver Fibrosis Through Inhibition of Notch Signaling Activation. *Am J Chin Med* 45(1), 85-104. doi: 10.1142/S0192415X17500070.
