# Supplementary figures and images for: The Novel Chinese Medicine JY5 Formula Alleviates Hepatic Fibrosis by Inhibiting the Notch Signaling Pathway (part 2 of 2)
Source: Front Pharmacol. 2021 Sep 22;12:671152. doi: 10.3389/fphar.2021.671152 (PMC8493219; doi:10.3389/fphar.2021.671152)

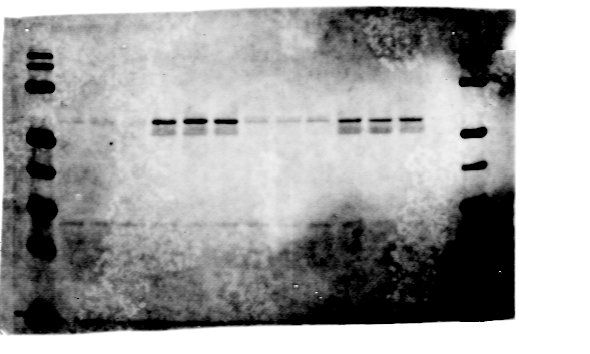

Supplement: Supplementary file 3 [file DataSheet4.ZIP › the original source data of Figures 5-7/Fig. 6/The original image file for the blots/Fig.6H Rat-BDL-RBPkB.jpg]

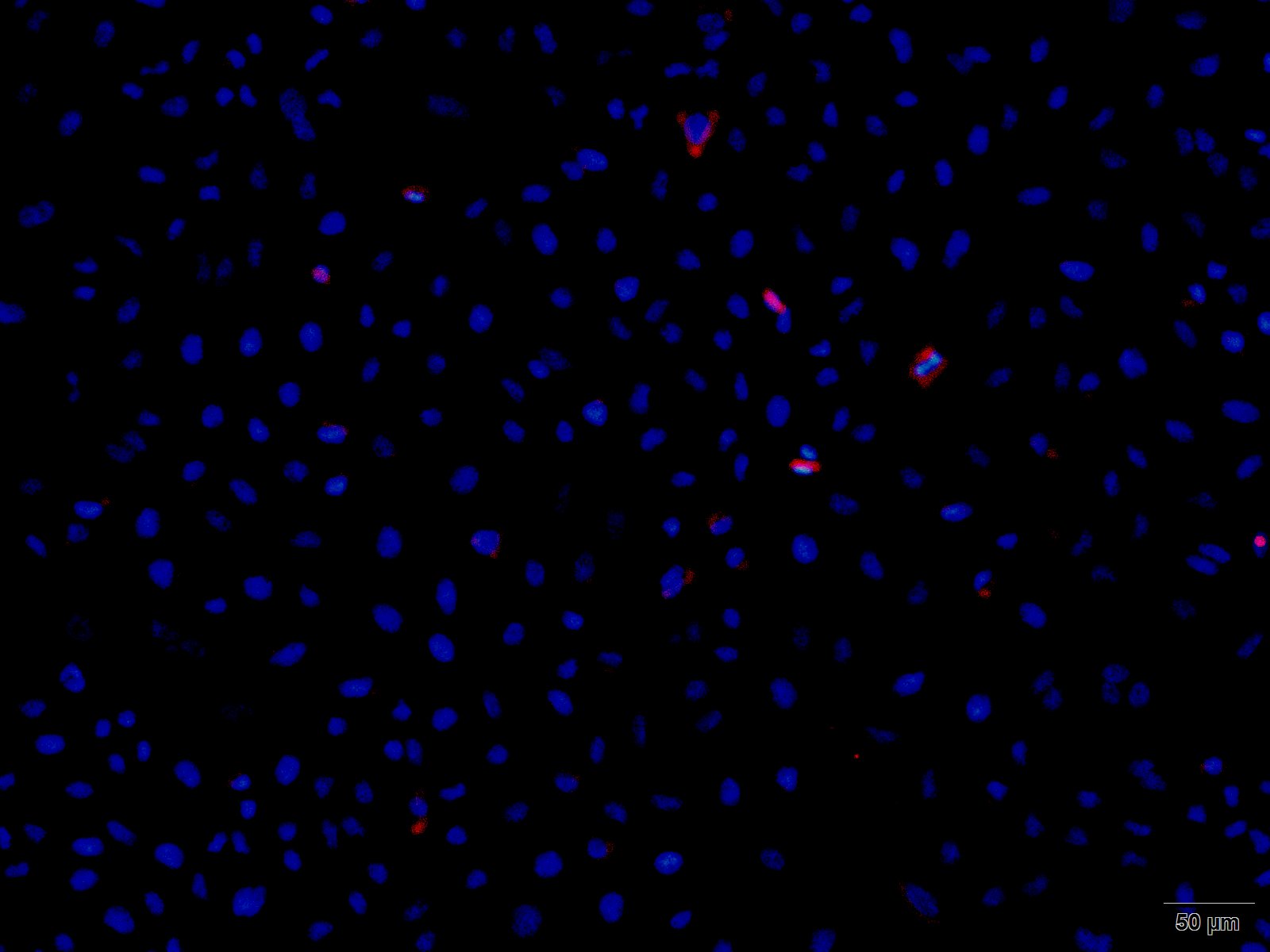

Supplement: Supplementary file 3 [file DataSheet4.ZIP › the original source data of Figures 5-7/Fig. 7/Fig. 7A (LX-2)/a-SMA/Control.jpg]

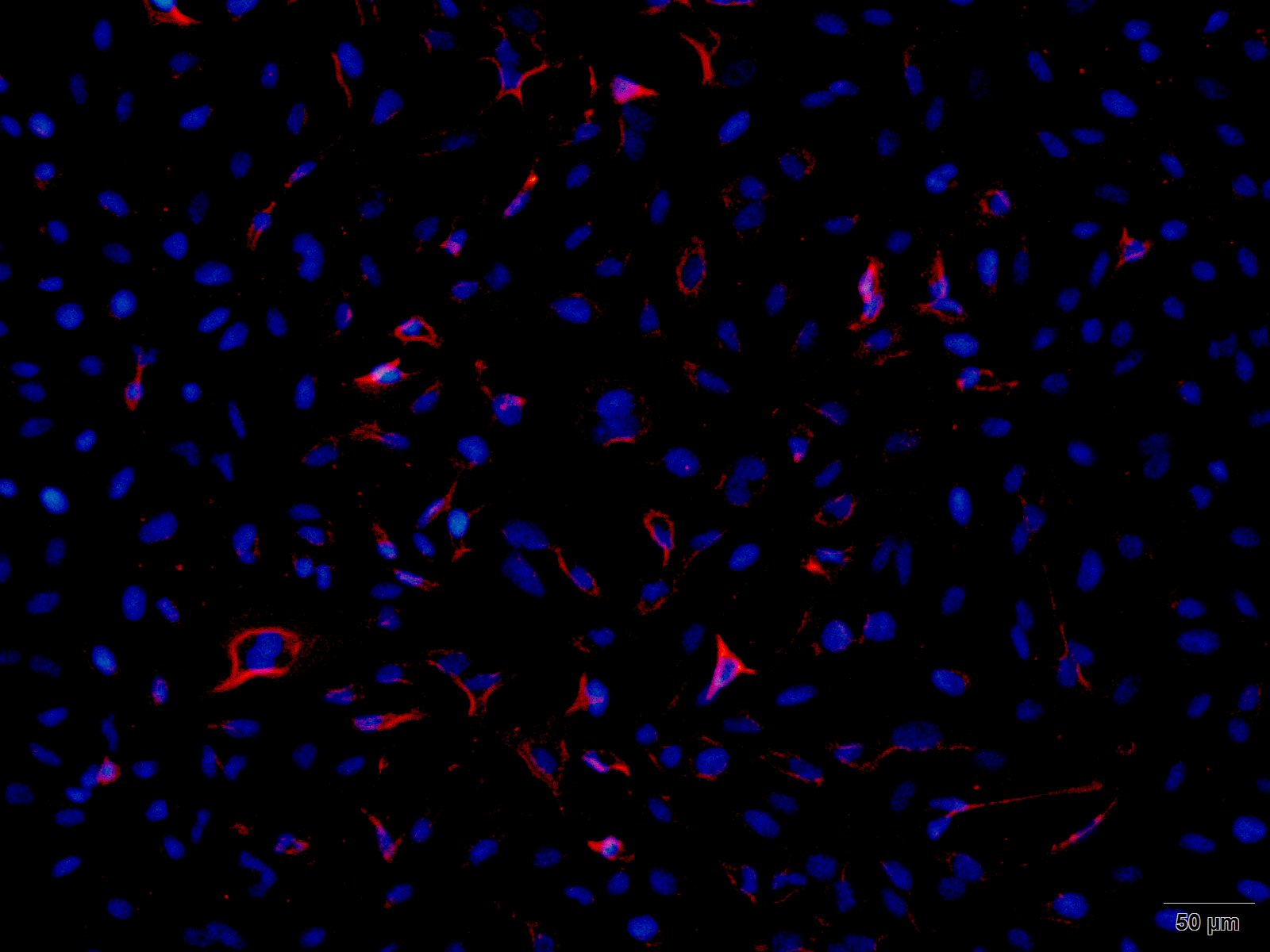

Supplement: Supplementary file 3 [file DataSheet4.ZIP › the original source data of Figures 5-7/Fig. 7/Fig. 7A (LX-2)/a-SMA/JY5(18.5uM).jpg]

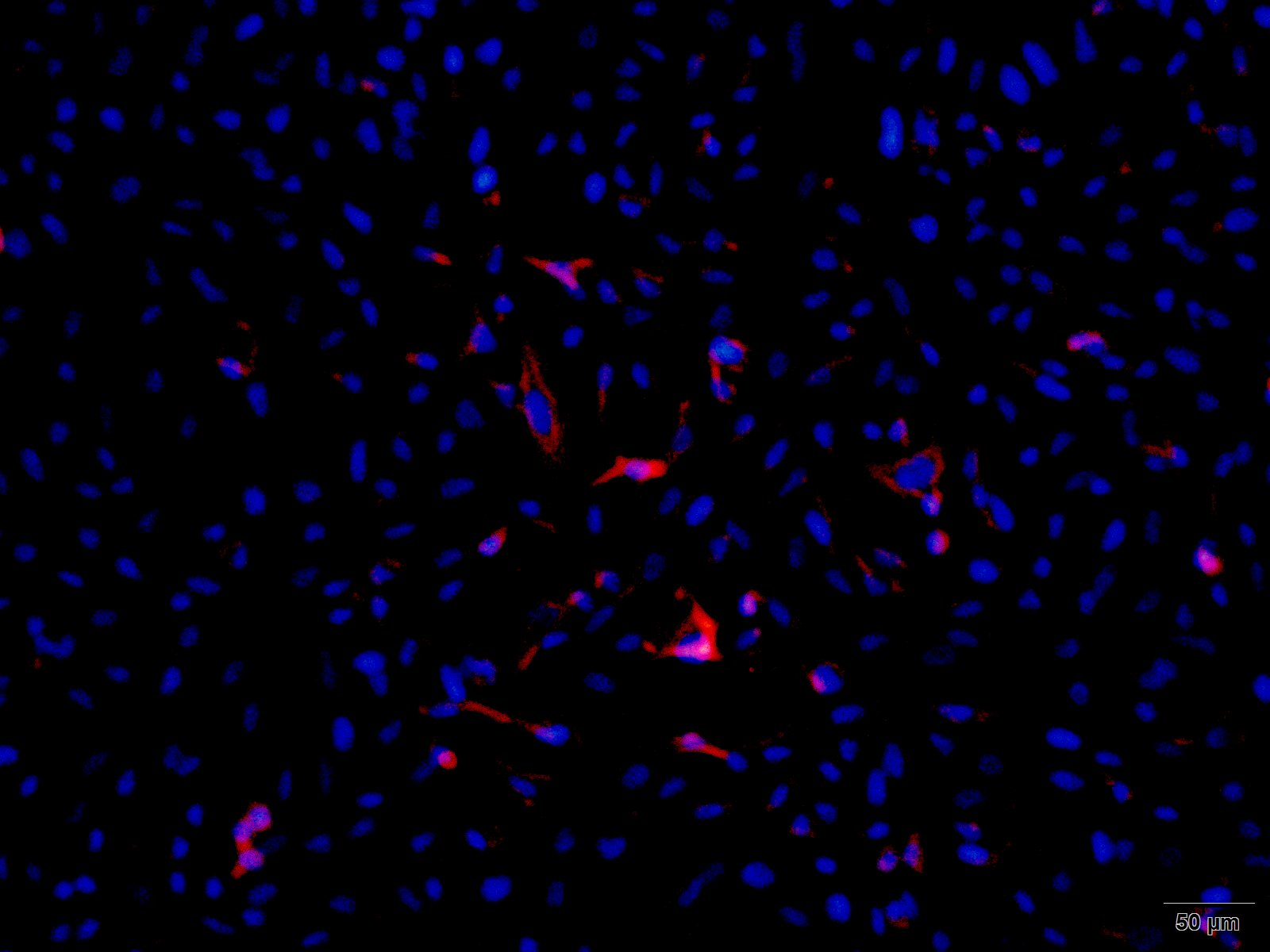

Supplement: Supplementary file 3 [file DataSheet4.ZIP › the original source data of Figures 5-7/Fig. 7/Fig. 7A (LX-2)/a-SMA/JY5(37uM).jpg]

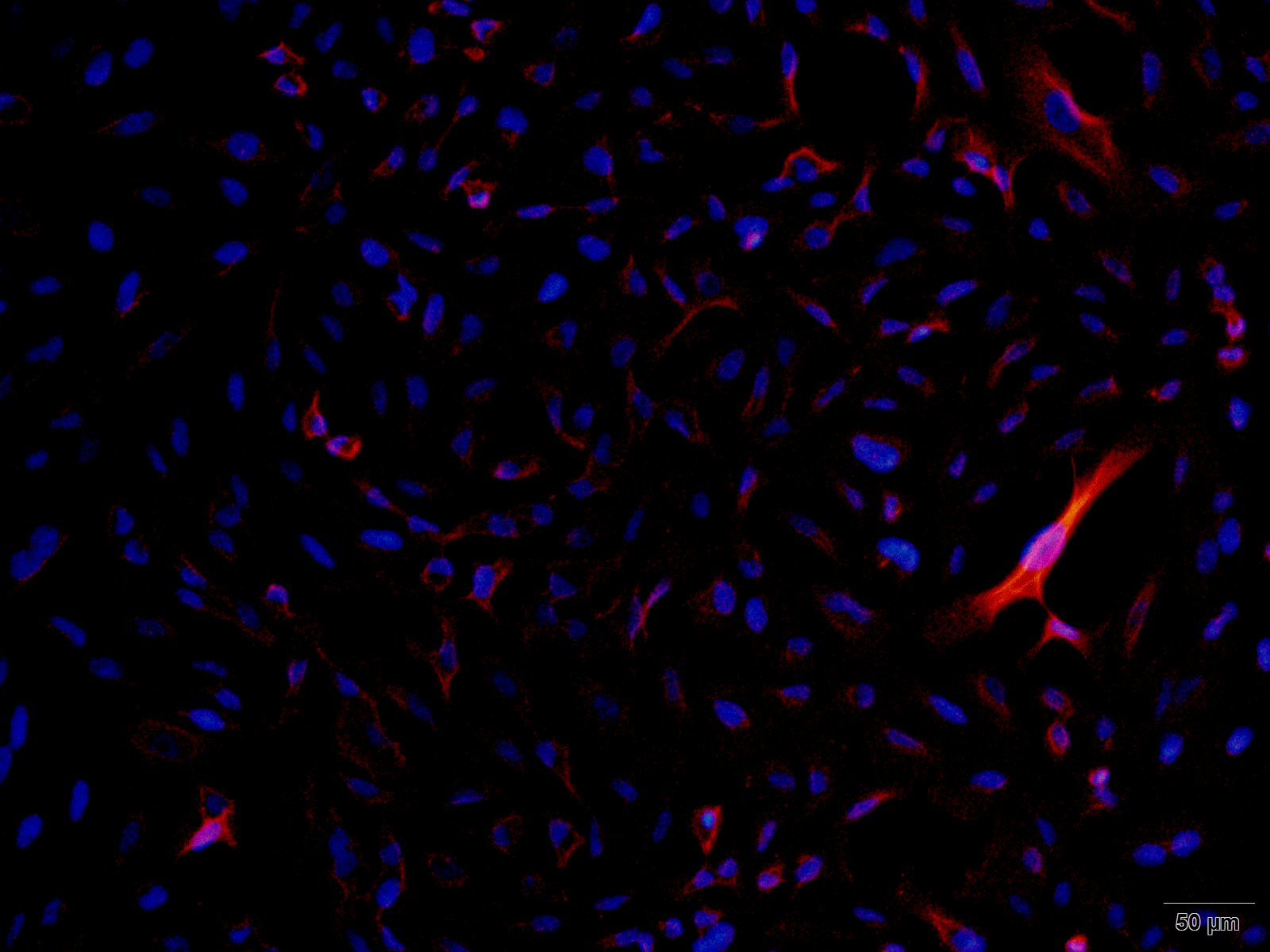

Supplement: Supplementary file 3 [file DataSheet4.ZIP › the original source data of Figures 5-7/Fig. 7/Fig. 7A (LX-2)/a-SMA/JY5(9.25uM).jpg]

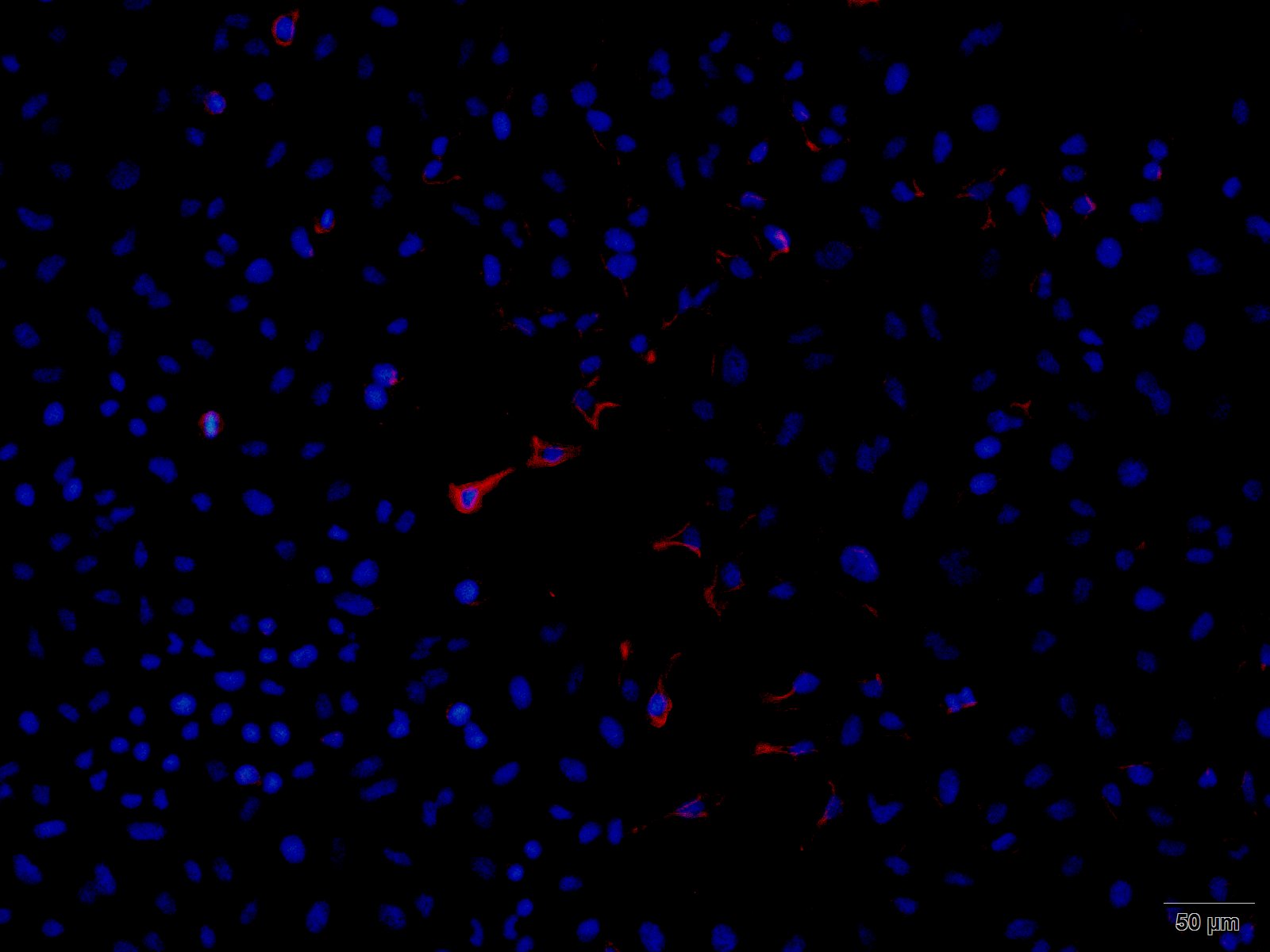

Supplement: Supplementary file 3 [file DataSheet4.ZIP › the original source data of Figures 5-7/Fig. 7/Fig. 7A (LX-2)/a-SMA/SB431542.jpg]

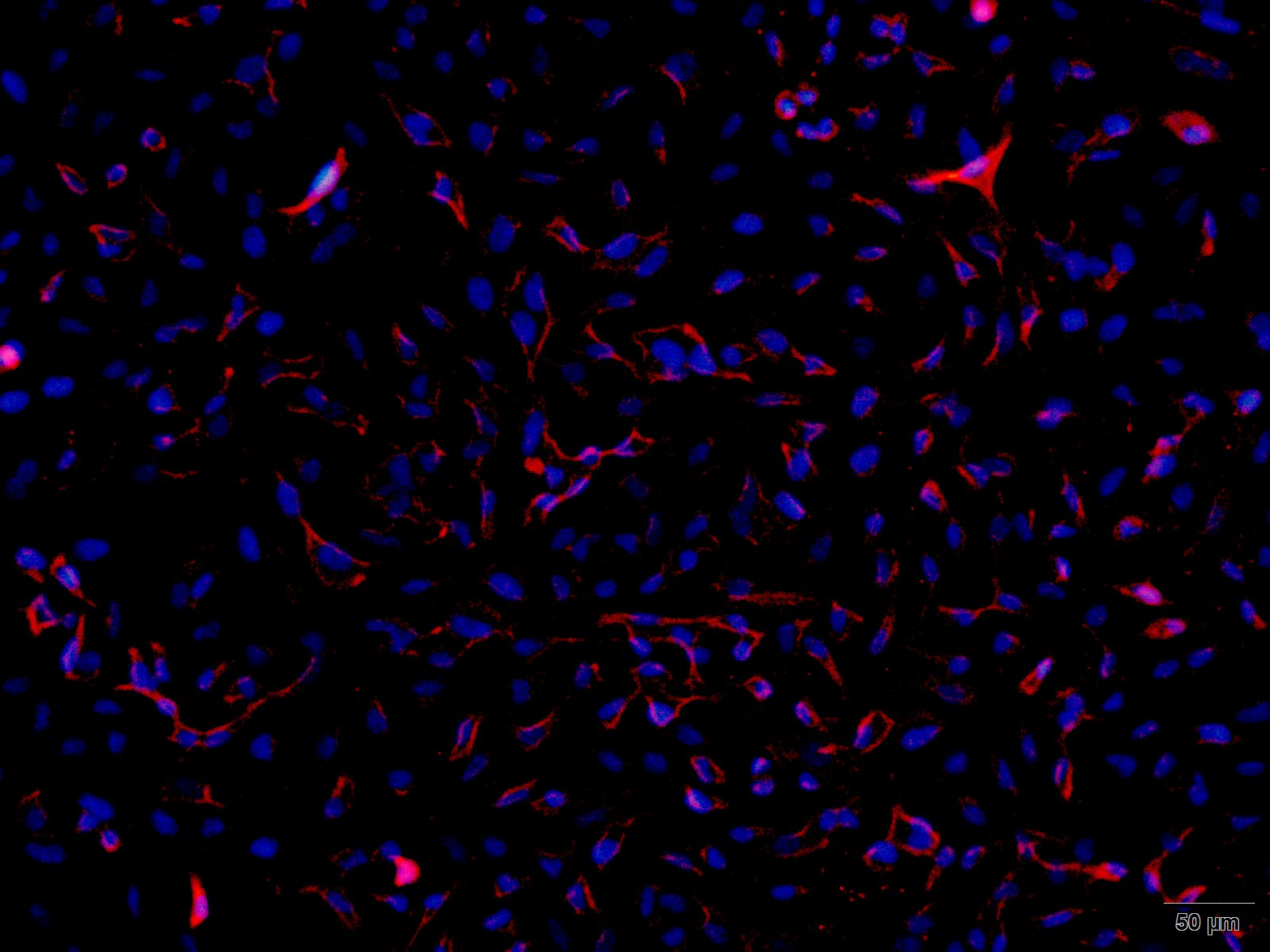

Supplement: Supplementary file 3 [file DataSheet4.ZIP › the original source data of Figures 5-7/Fig. 7/Fig. 7A (LX-2)/a-SMA/TGF-beta1.jpg]

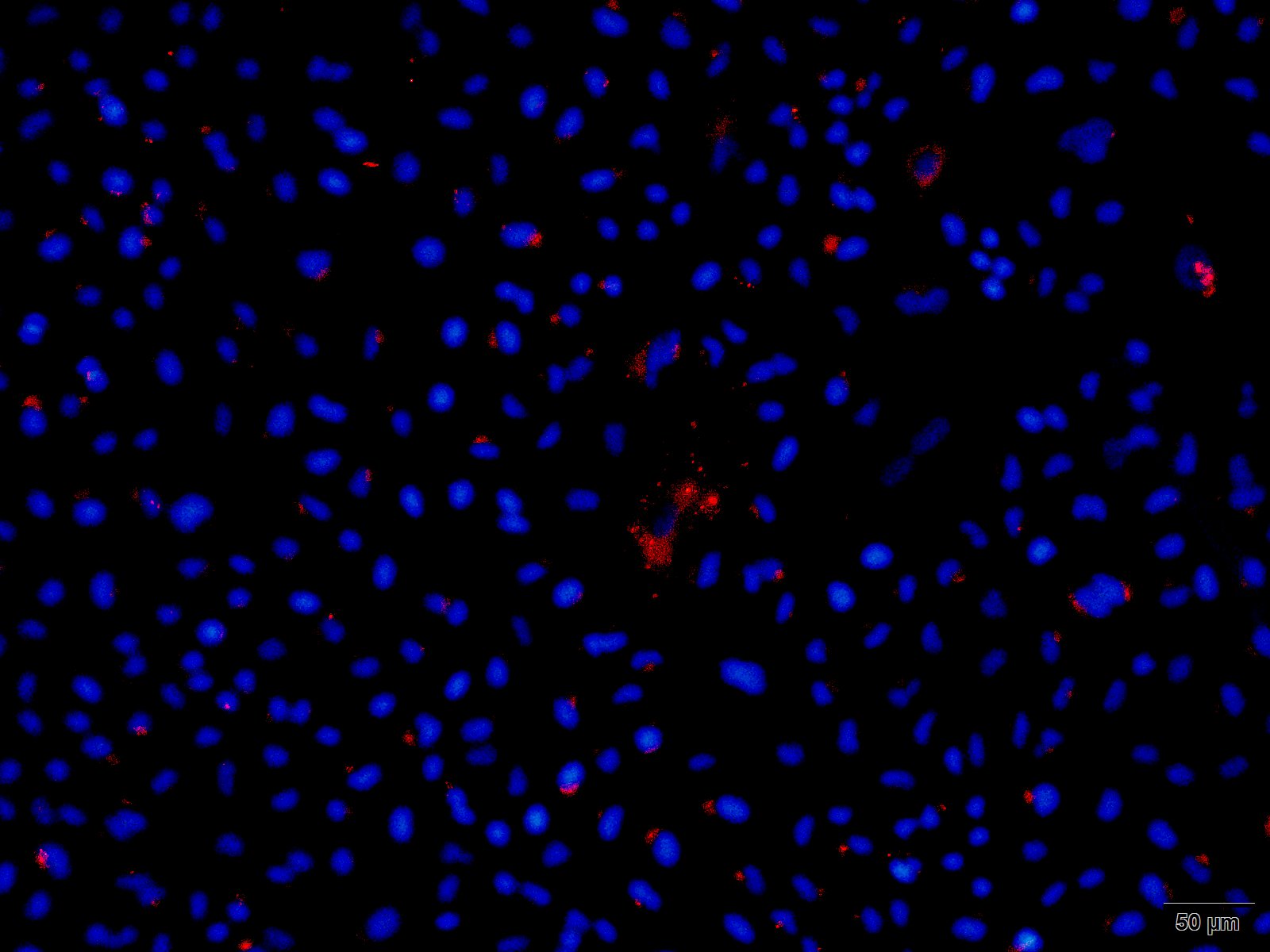

Supplement: Supplementary file 3 [file DataSheet4.ZIP › the original source data of Figures 5-7/Fig. 7/Fig. 7A (LX-2)/Col-I/Control.jpg]

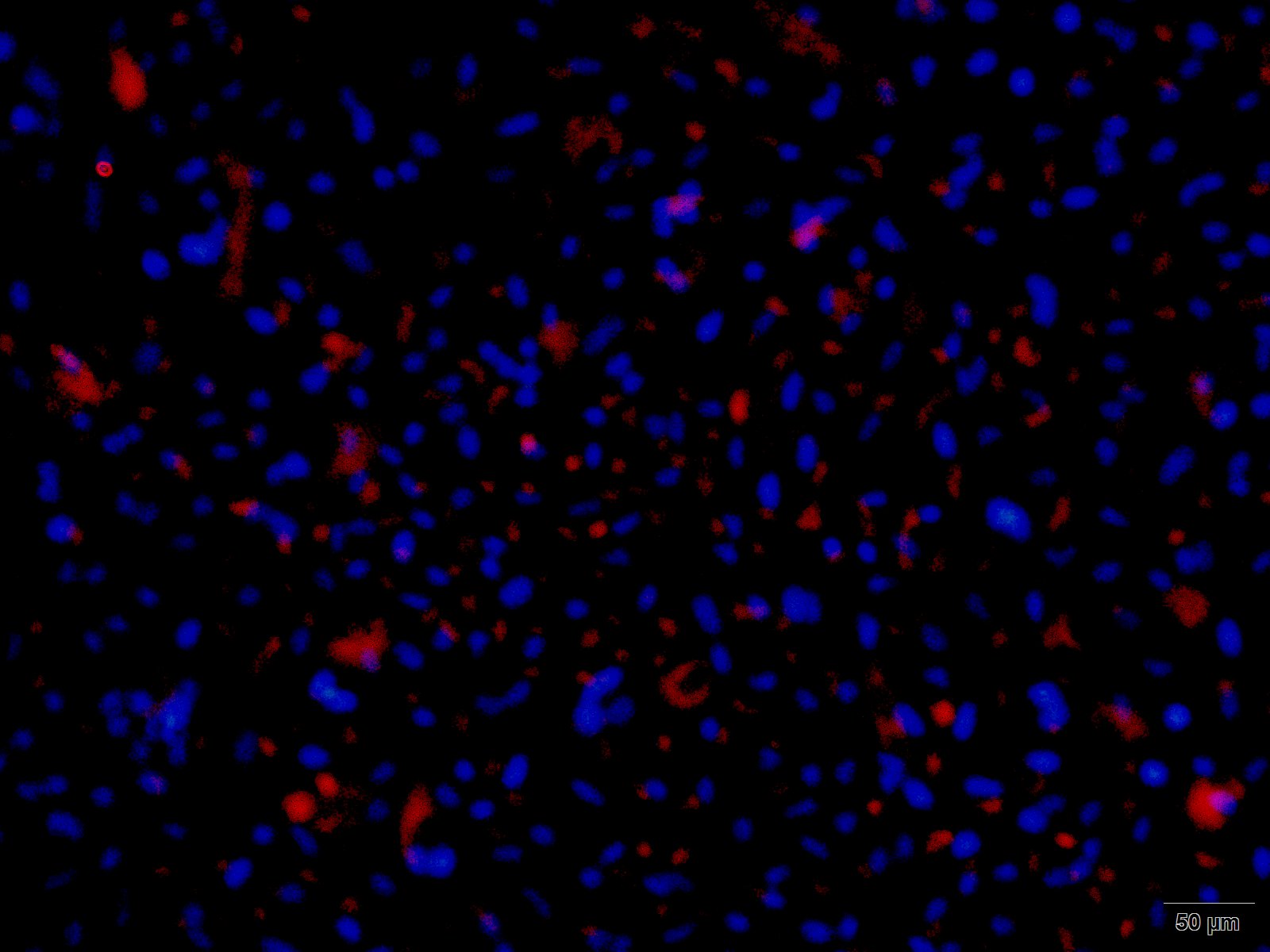

Supplement: Supplementary file 3 [file DataSheet4.ZIP › the original source data of Figures 5-7/Fig. 7/Fig. 7A (LX-2)/Col-I/JY5(18.5uM).jpg]

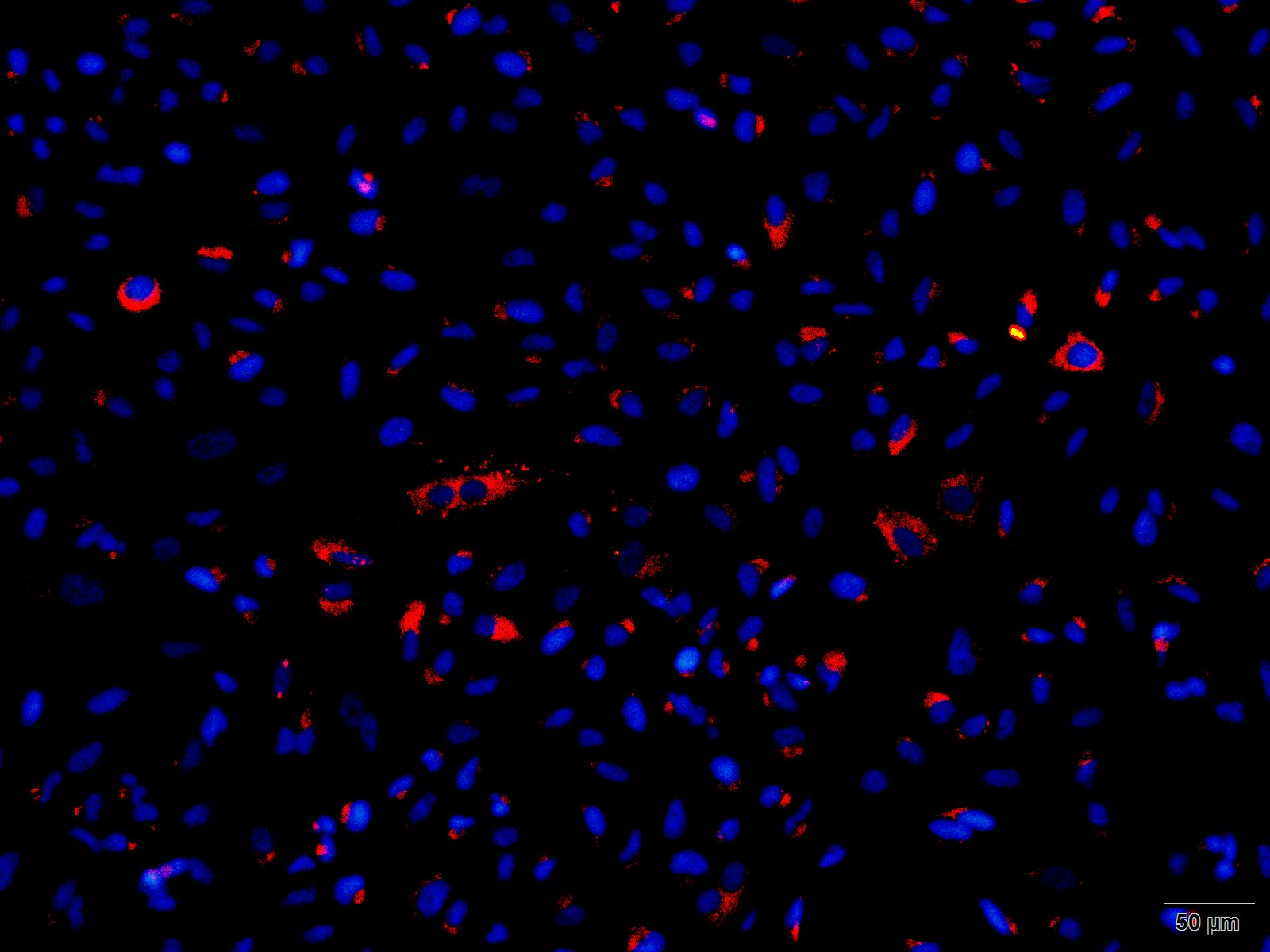

Supplement: Supplementary file 3 [file DataSheet4.ZIP › the original source data of Figures 5-7/Fig. 7/Fig. 7A (LX-2)/Col-I/JY5(37uM).jpg]

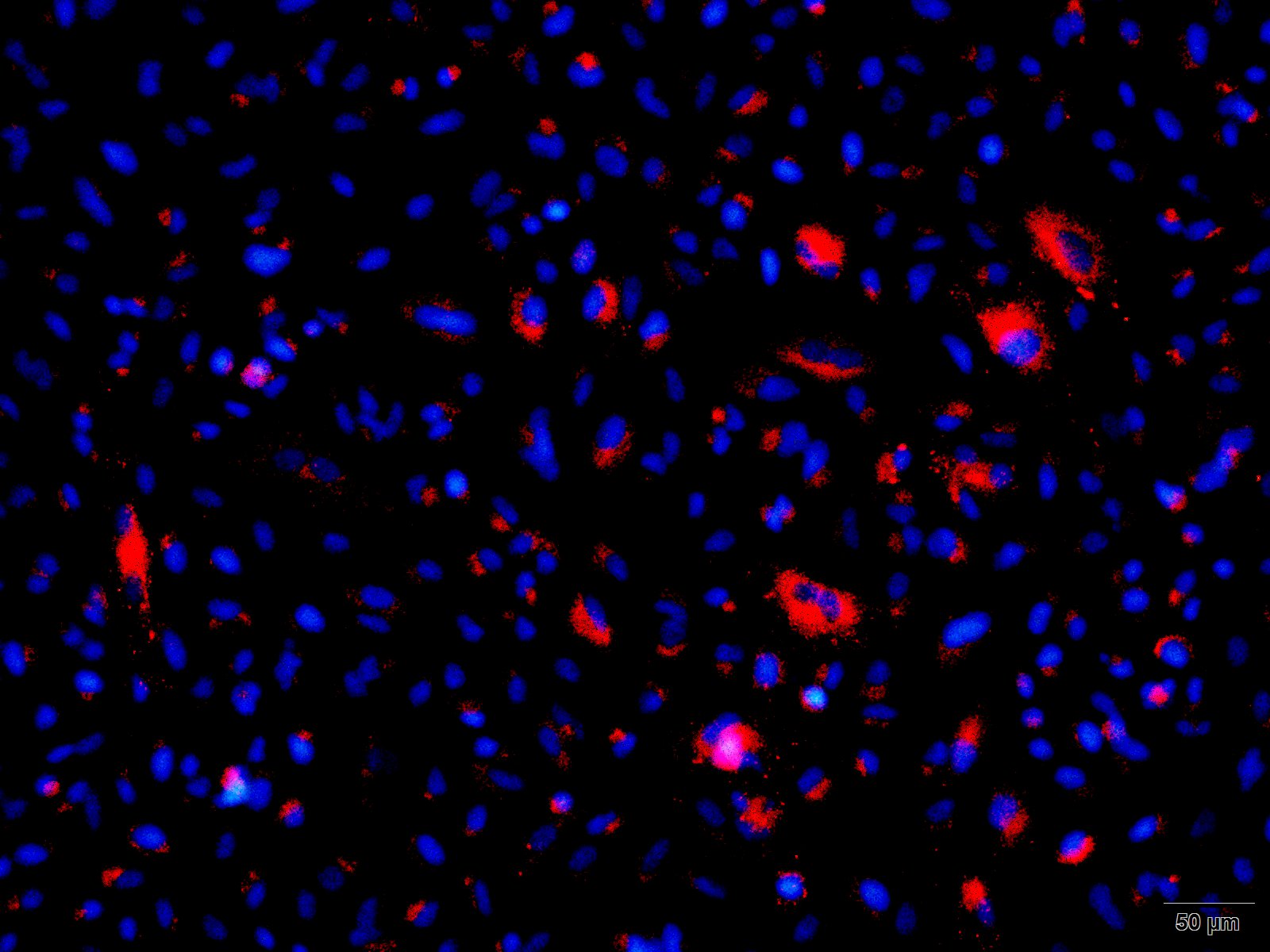

Supplement: Supplementary file 3 [file DataSheet4.ZIP › the original source data of Figures 5-7/Fig. 7/Fig. 7A (LX-2)/Col-I/JY5(9.25uM).jpg]

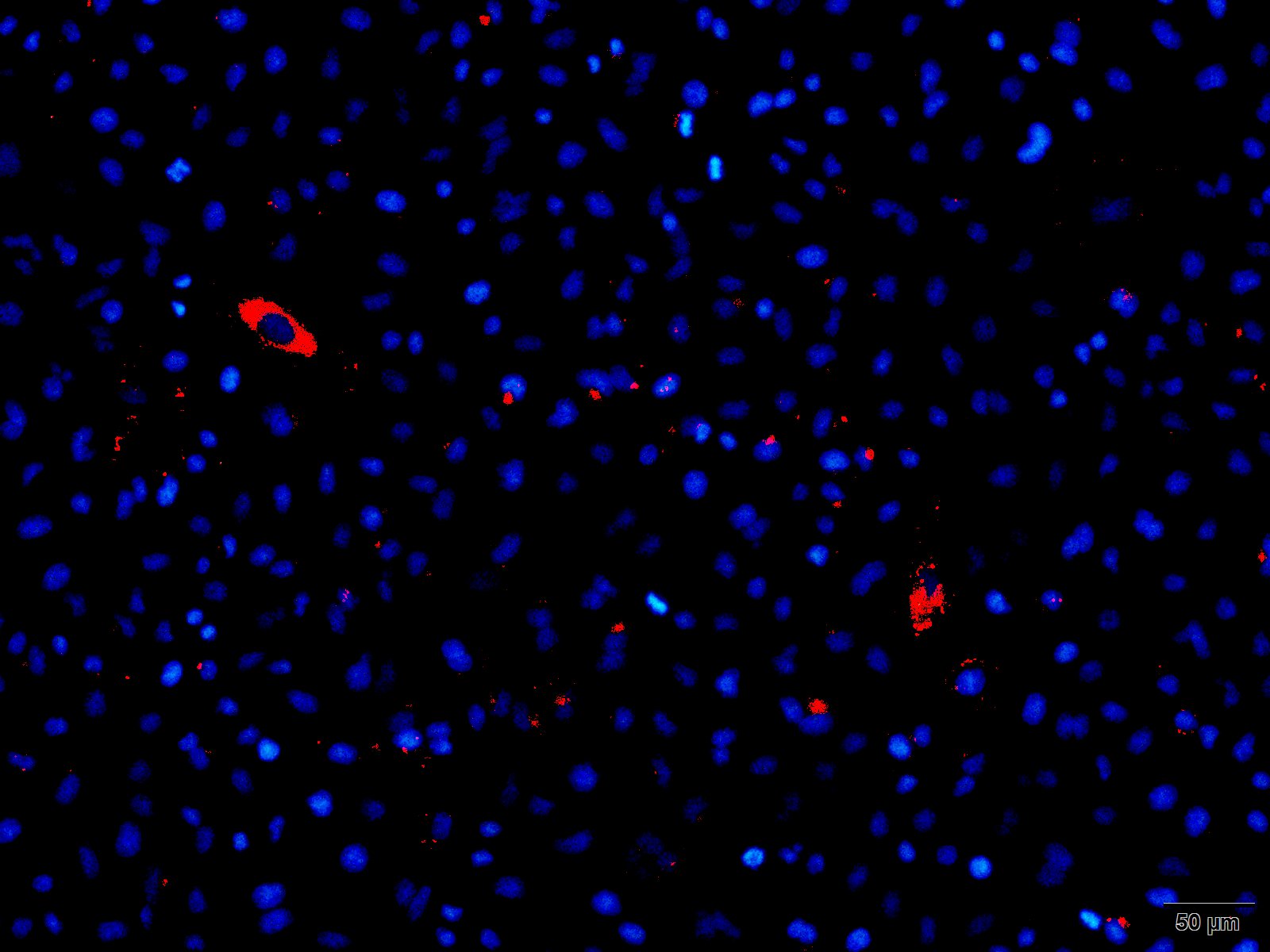

Supplement: Supplementary file 3 [file DataSheet4.ZIP › the original source data of Figures 5-7/Fig. 7/Fig. 7A (LX-2)/Col-I/SB431542.jpg]

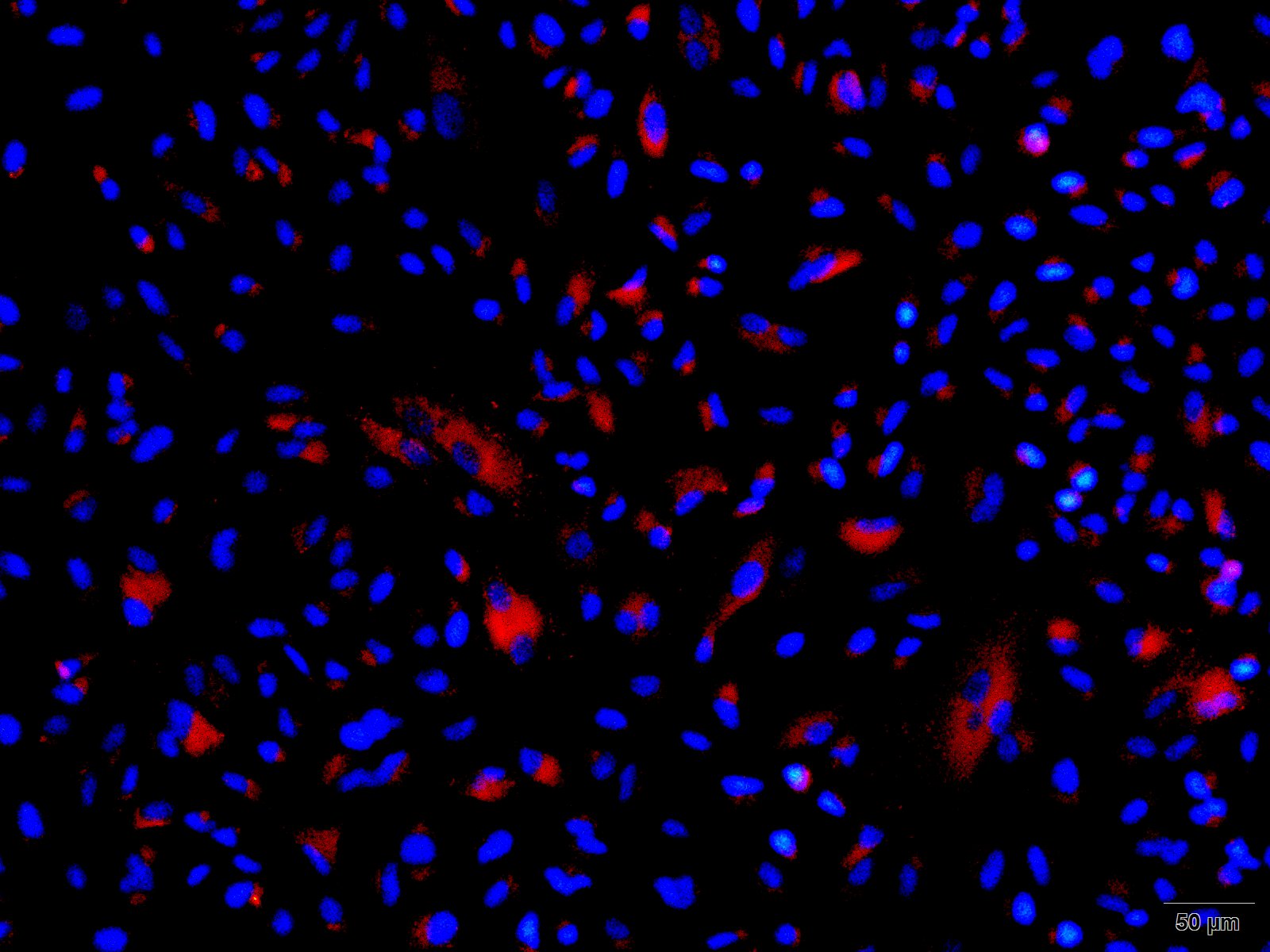

Supplement: Supplementary file 3 [file DataSheet4.ZIP › the original source data of Figures 5-7/Fig. 7/Fig. 7A (LX-2)/Col-I/TGF-beta1.jpg]

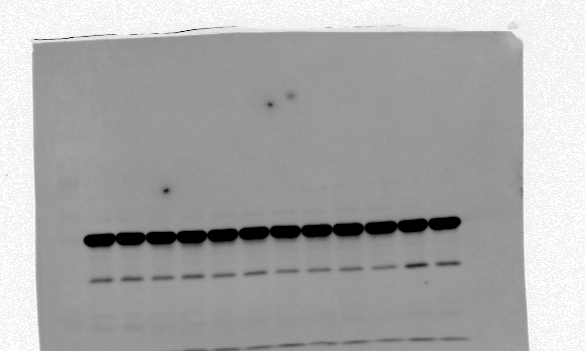

Supplement: Supplementary file 3 [file DataSheet4.ZIP › the original source data of Figures 5-7/Fig. 7/The original image file for the blots/Fig. 7C LX-2-a-SMA-GAPDH.jpg]

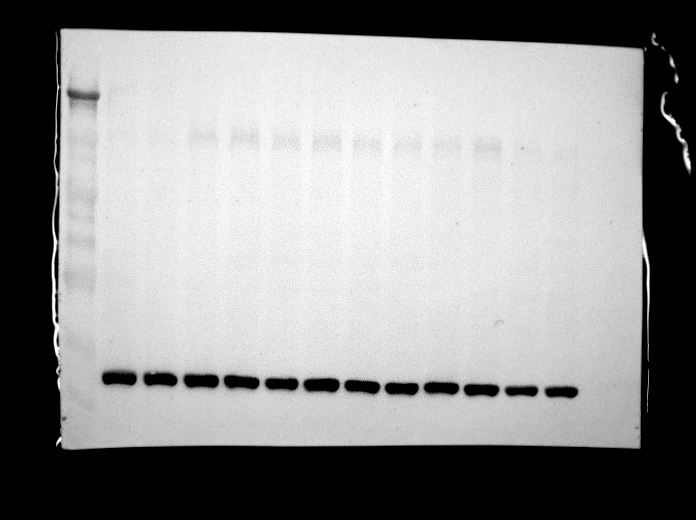

Supplement: Supplementary file 3 [file DataSheet4.ZIP › the original source data of Figures 5-7/Fig. 7/The original image file for the blots/Fig. 7F LX-2-Jagged1-beta-actin.tif]

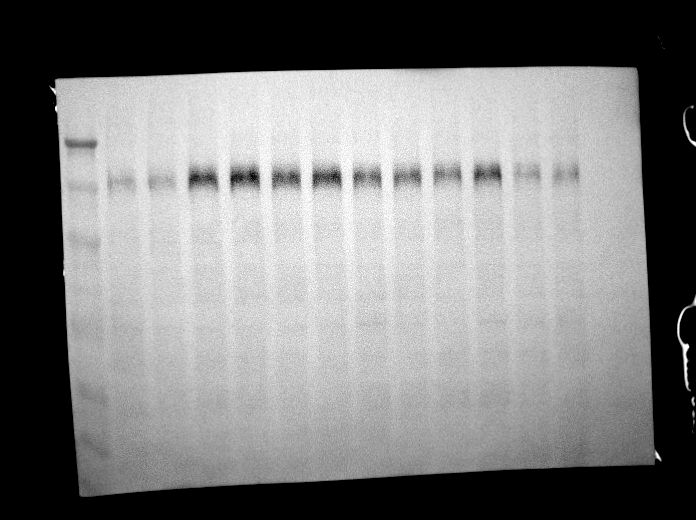

Supplement: Supplementary file 3 [file DataSheet4.ZIP › the original source data of Figures 5-7/Fig. 7/The original image file for the blots/Fig. 7F LX-2-Jagged1.tif]

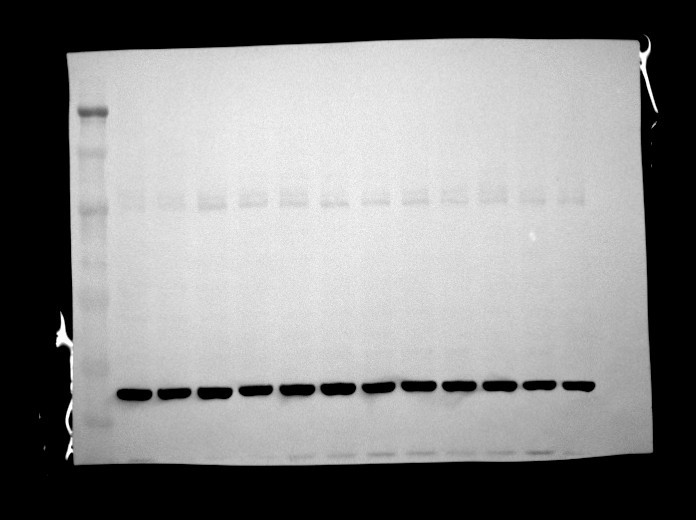

Supplement: Supplementary file 3 [file DataSheet4.ZIP › the original source data of Figures 5-7/Fig. 7/The original image file for the blots/Fig. 7H LX-2-Notch2-GAPDH.jpg]

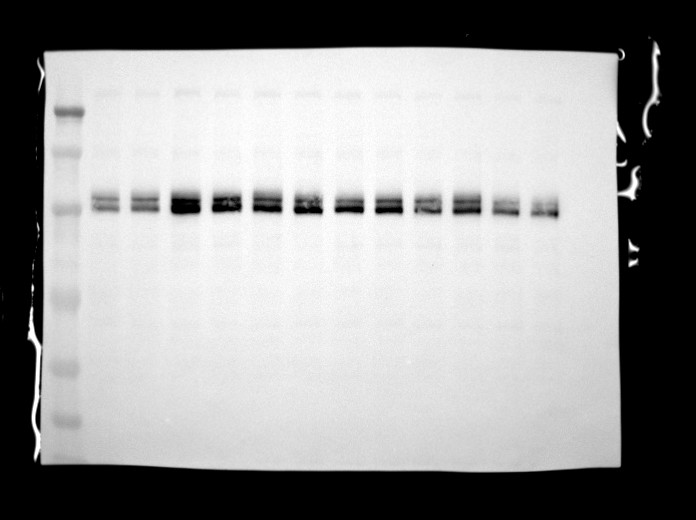

Supplement: Supplementary file 3 [file DataSheet4.ZIP › the original source data of Figures 5-7/Fig. 7/The original image file for the blots/Fig. 7H LX-2-Notch2.jpg]

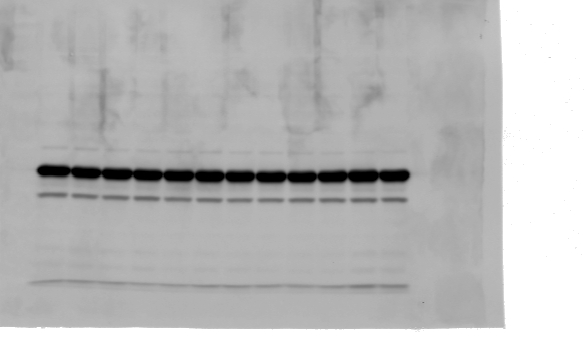

Supplement: Supplementary file 3 [file DataSheet4.ZIP › the original source data of Figures 5-7/Fig. 7/The original image file for the blots/Fig. 7K LX-2-RBP-kB-GAPDH.jpg]

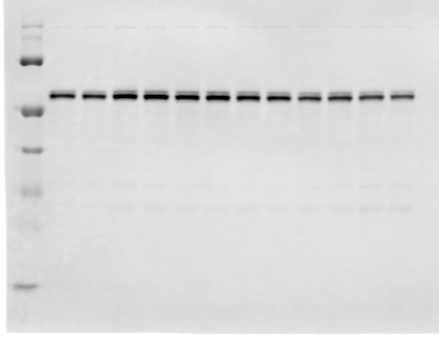

Supplement: Supplementary file 3 [file DataSheet4.ZIP › the original source data of Figures 5-7/Fig. 7/The original image file for the blots/Fig. 7K LX-2-RBP-kB.jpg]

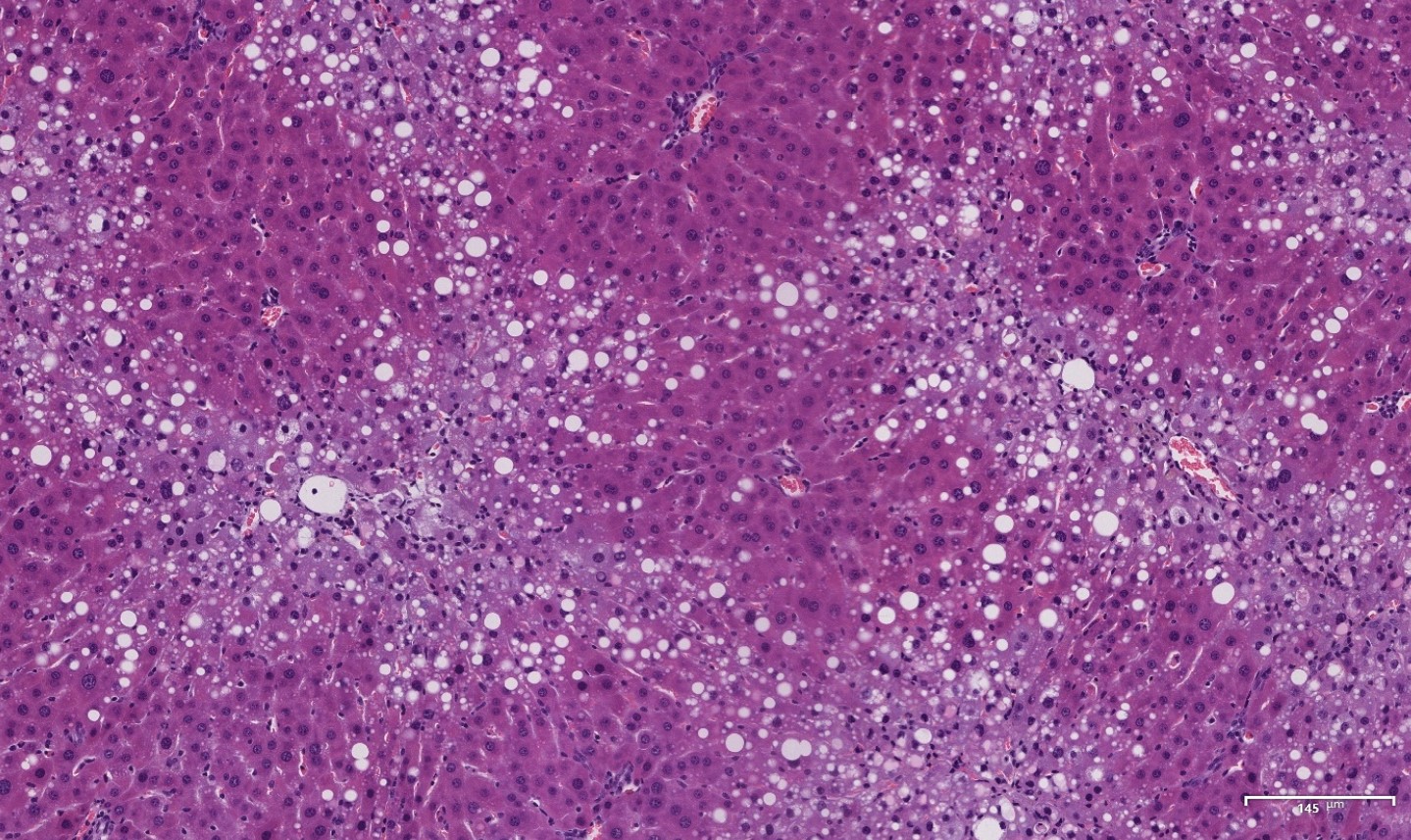

Supplement: Supplementary file 6 [file DataSheet5.ZIP › the original source data of Supplementary Figures /Fig. S3/Fig. S3A (Rat-CCl4)/H&E/CCl4.jpg]

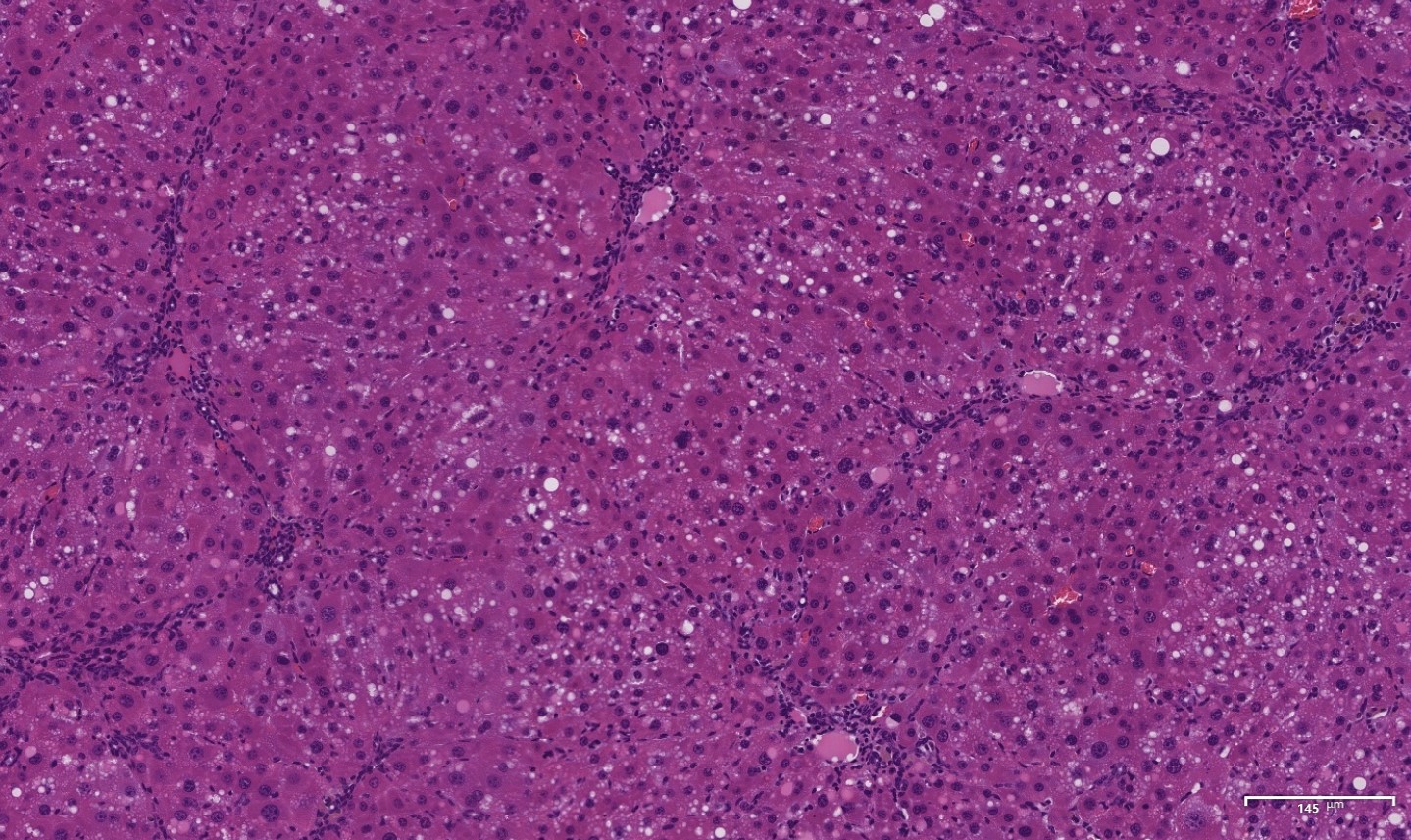

Supplement: Supplementary file 6 [file DataSheet5.ZIP › the original source data of Supplementary Figures /Fig. S3/Fig. S3A (Rat-CCl4)/H&E/FZHY.jpg]

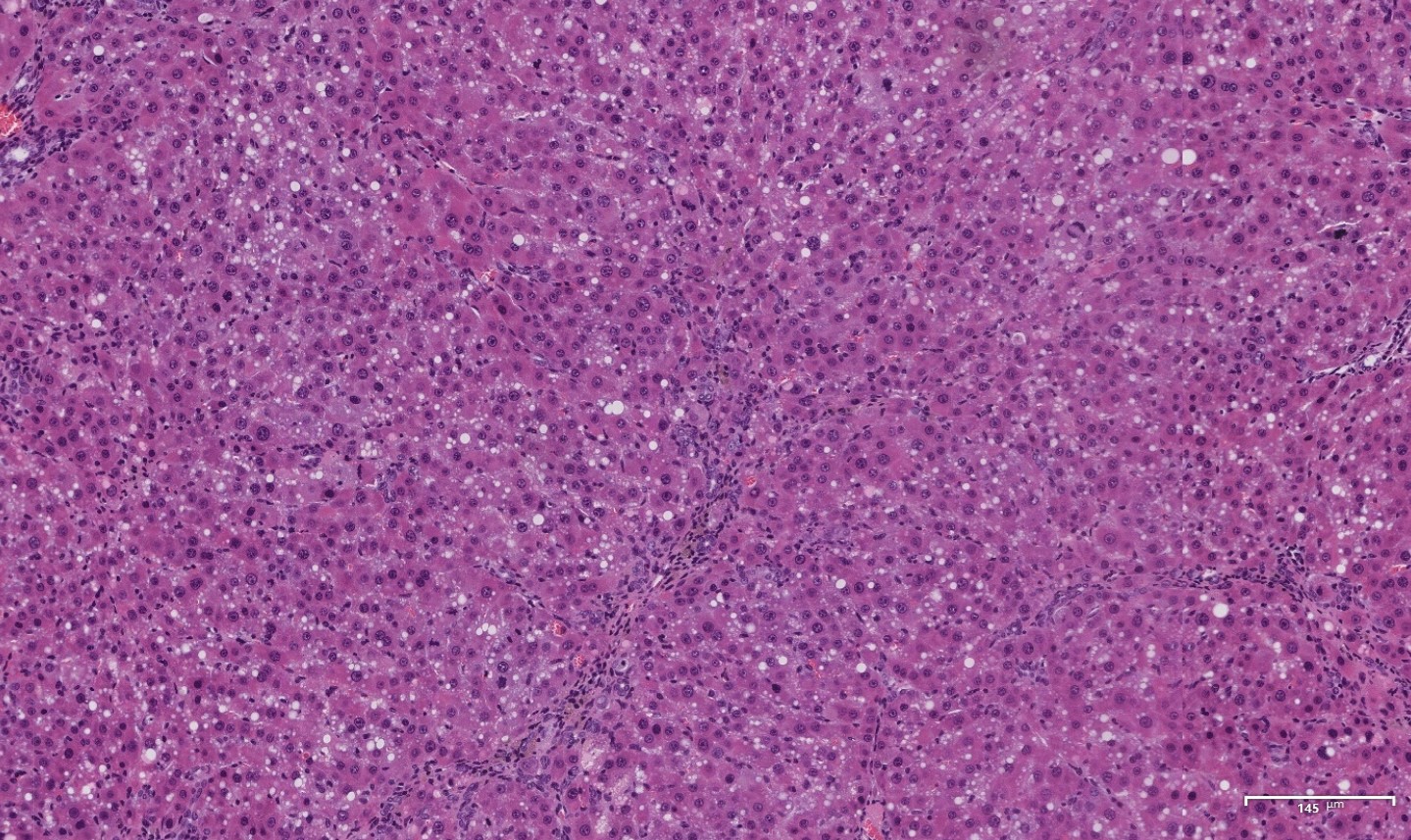

Supplement: Supplementary file 6 [file DataSheet5.ZIP › the original source data of Supplementary Figures /Fig. S3/Fig. S3A (Rat-CCl4)/H&E/JY5.jpg]

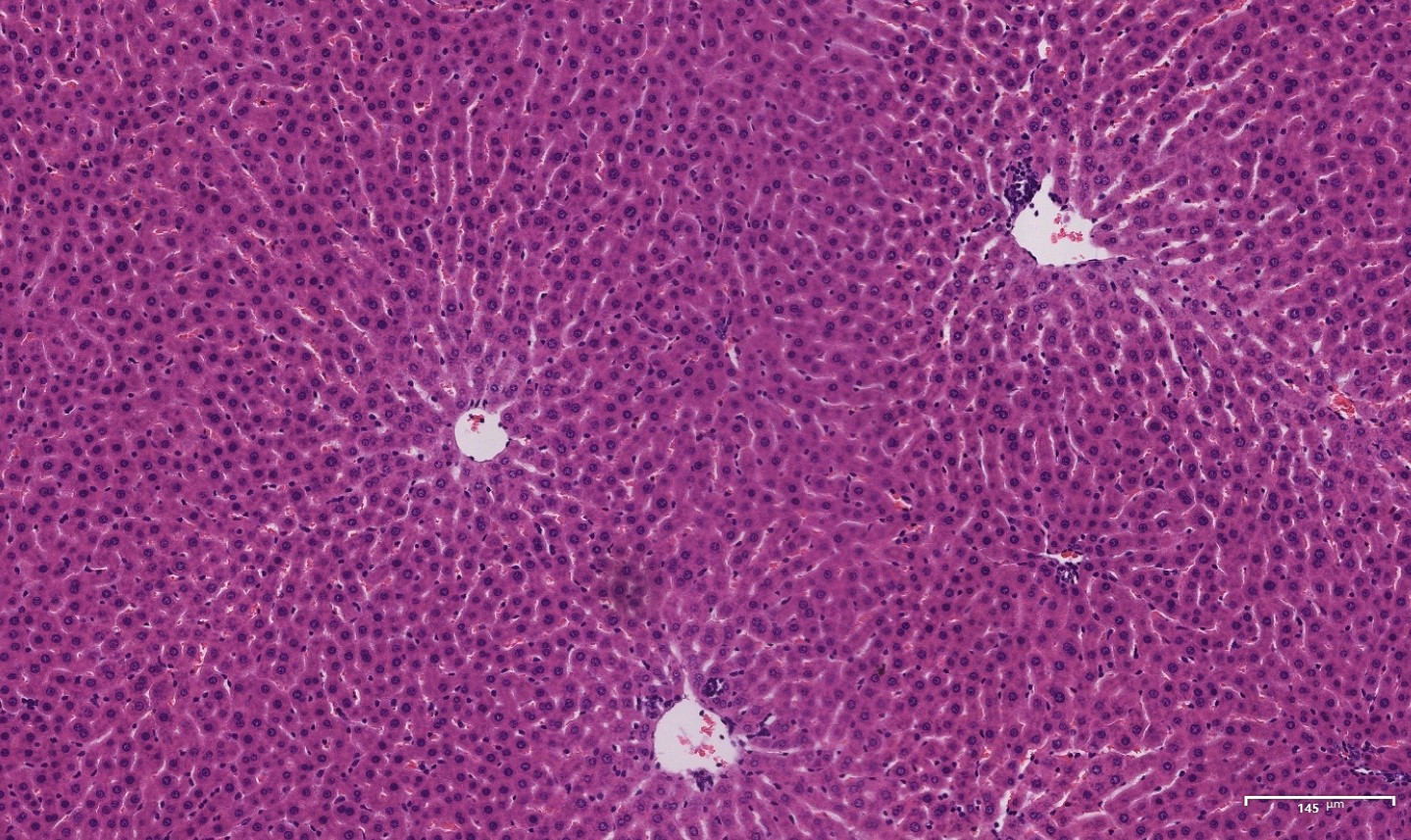

Supplement: Supplementary file 6 [file DataSheet5.ZIP › the original source data of Supplementary Figures /Fig. S3/Fig. S3A (Rat-CCl4)/H&E/Oil.jpg]

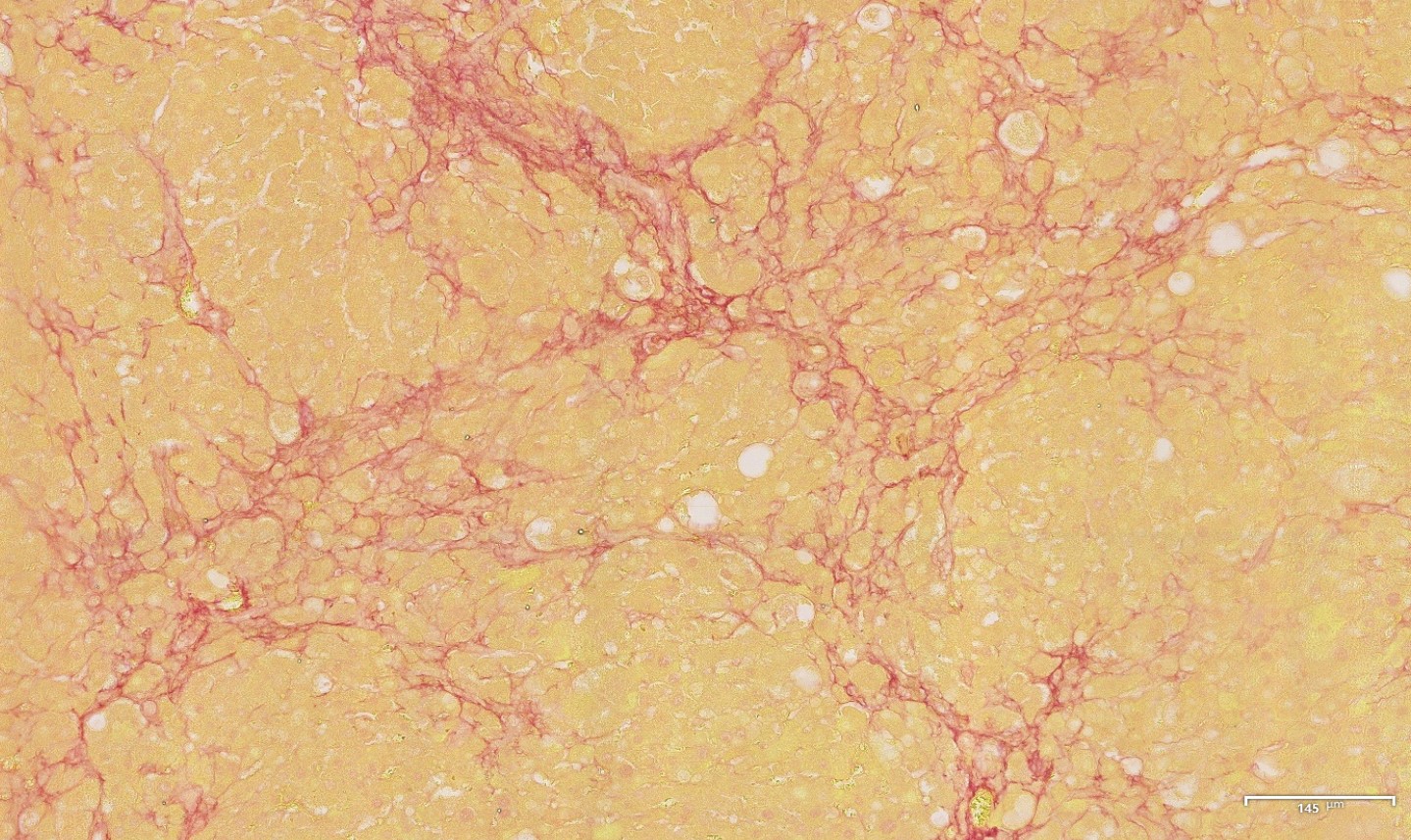

Supplement: Supplementary file 6 [file DataSheet5.ZIP › the original source data of Supplementary Figures /Fig. S3/Fig. S3A (Rat-CCl4)/SR/CCl4.jpg]

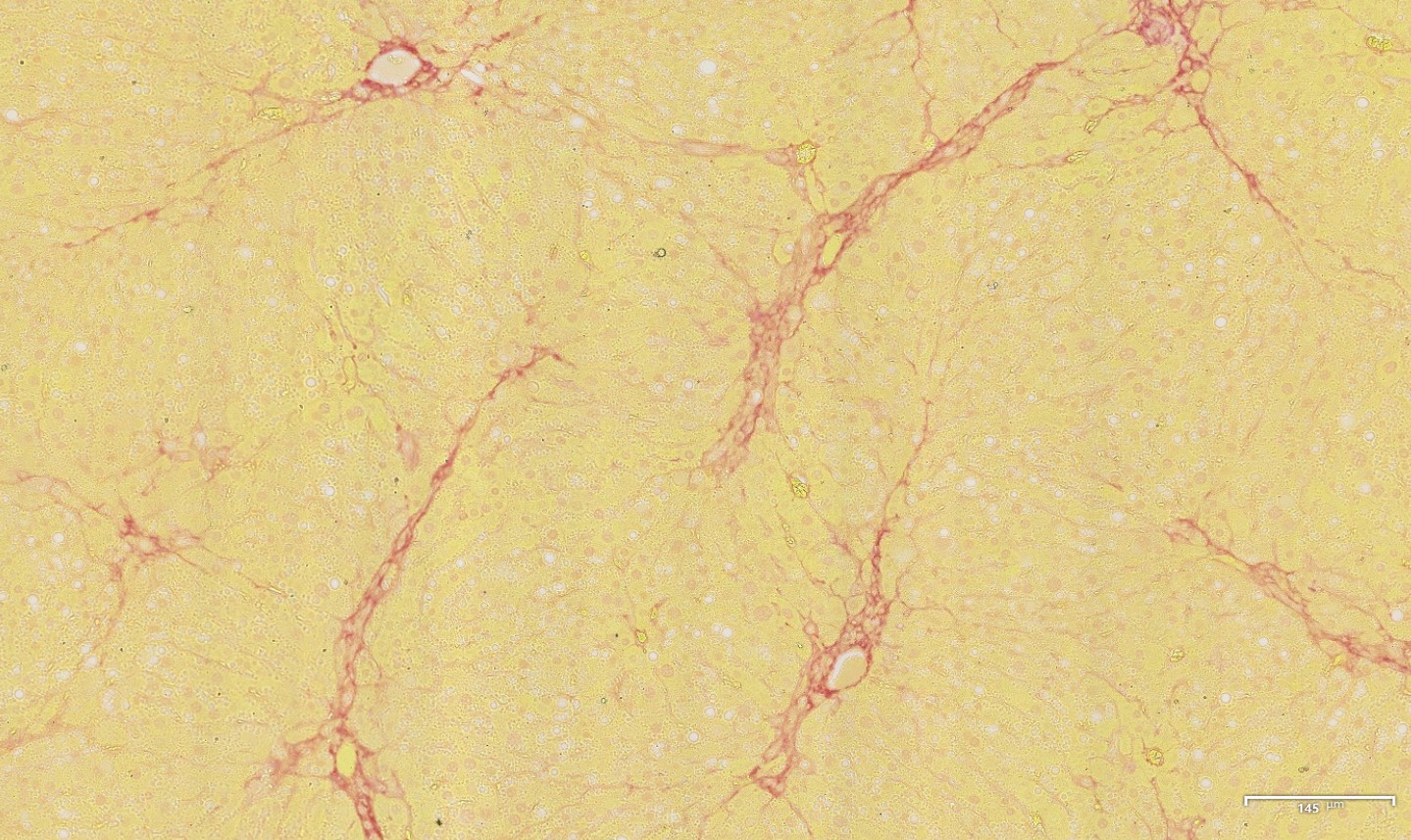

Supplement: Supplementary file 6 [file DataSheet5.ZIP › the original source data of Supplementary Figures /Fig. S3/Fig. S3A (Rat-CCl4)/SR/FZHY.jpg]

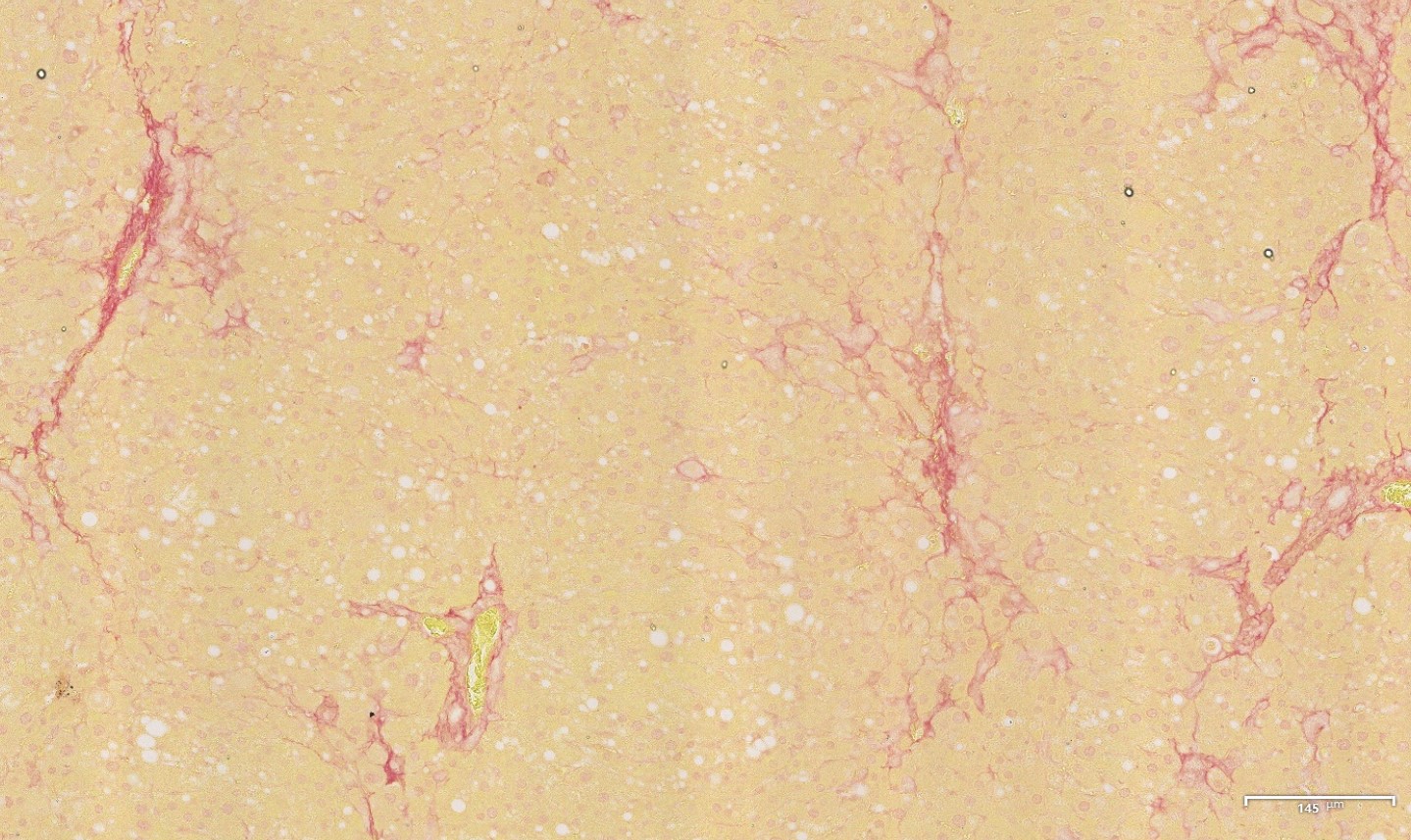

Supplement: Supplementary file 6 [file DataSheet5.ZIP › the original source data of Supplementary Figures /Fig. S3/Fig. S3A (Rat-CCl4)/SR/JY5.jpg]

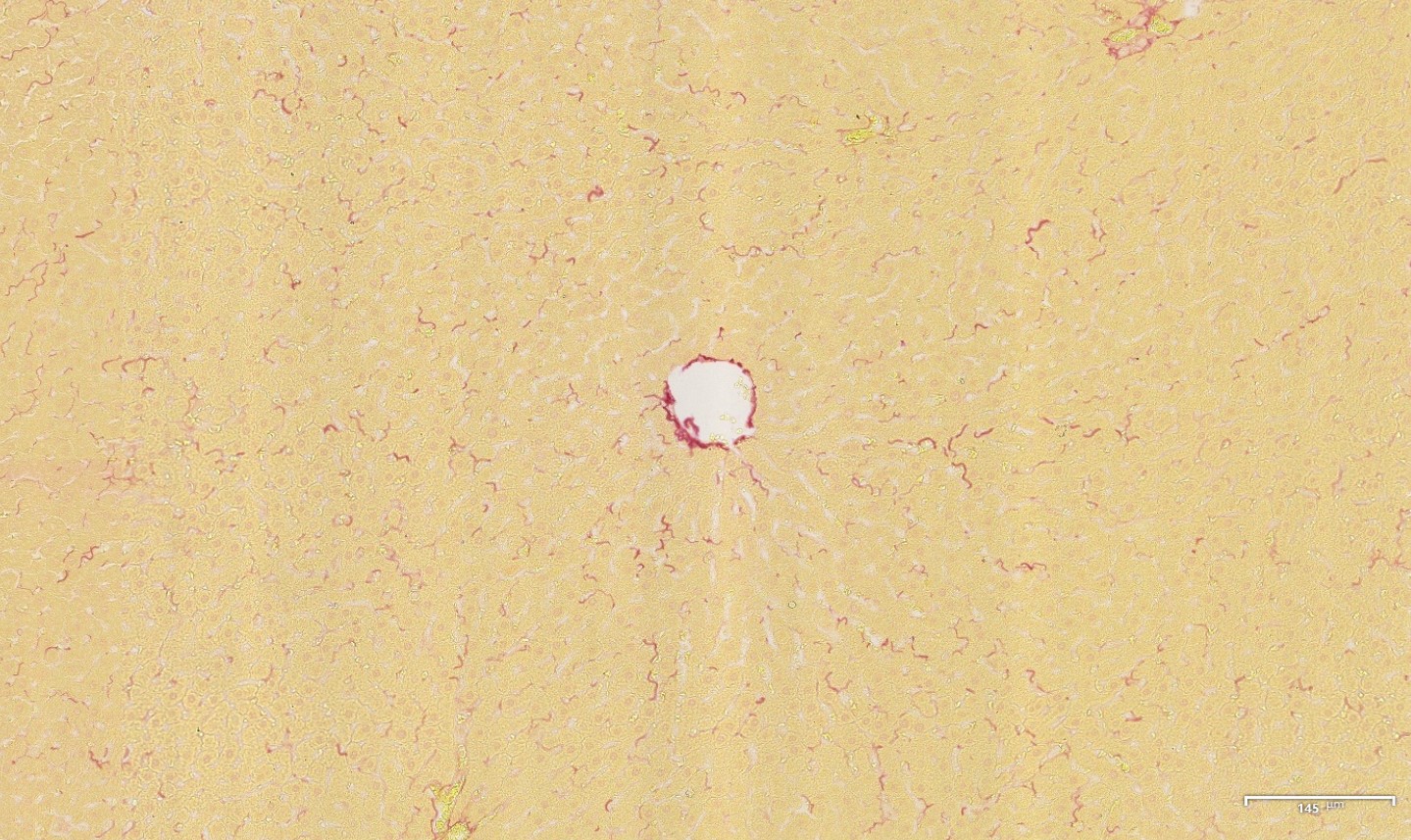

Supplement: Supplementary file 6 [file DataSheet5.ZIP › the original source data of Supplementary Figures /Fig. S3/Fig. S3A (Rat-CCl4)/SR/Oil.jpg]

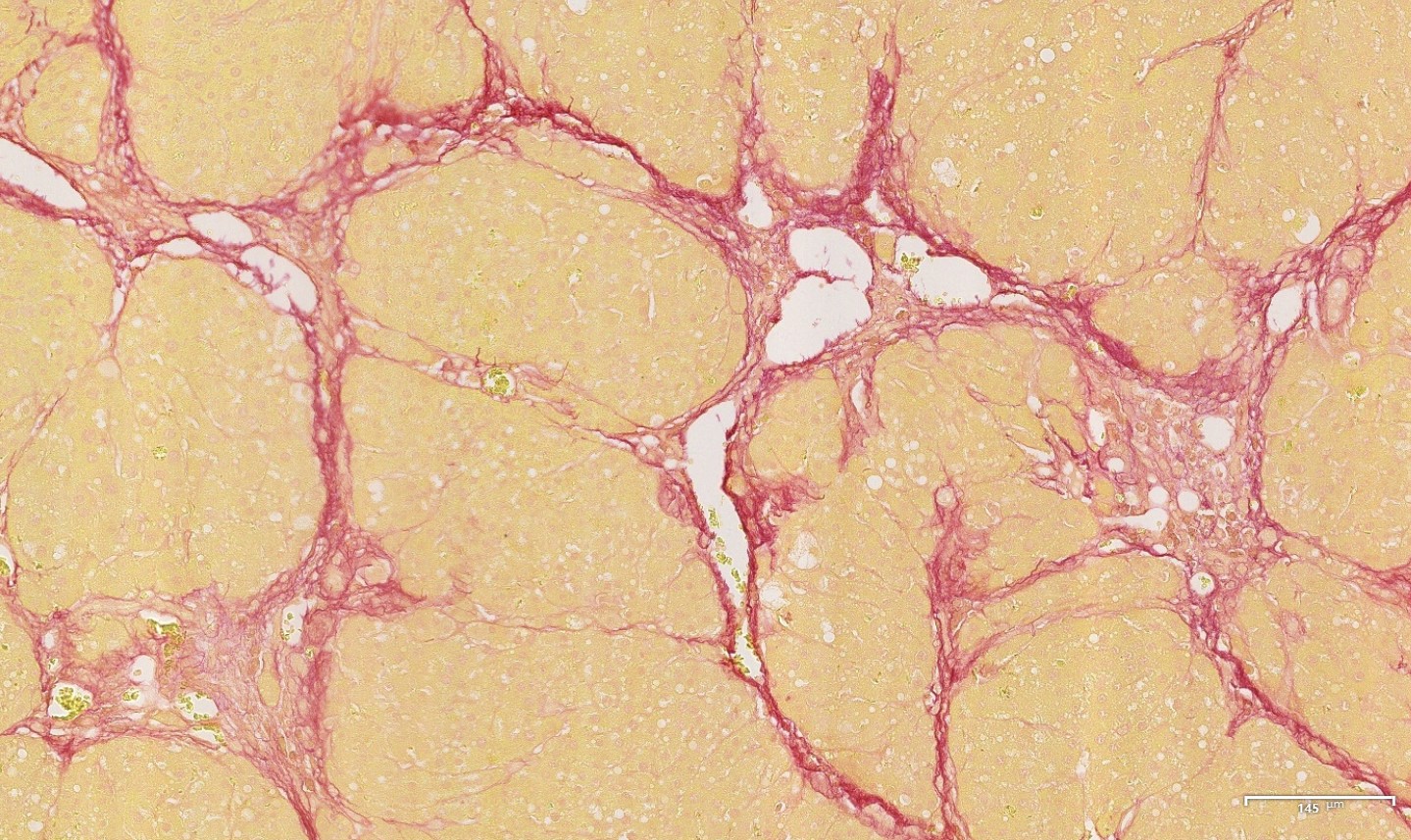

Supplement: Supplementary file 6 [file DataSheet5.ZIP › the original source data of Supplementary Figures /Fig. S4/Fig. S4A (Rat-CCl4)/CCl4.jpg]

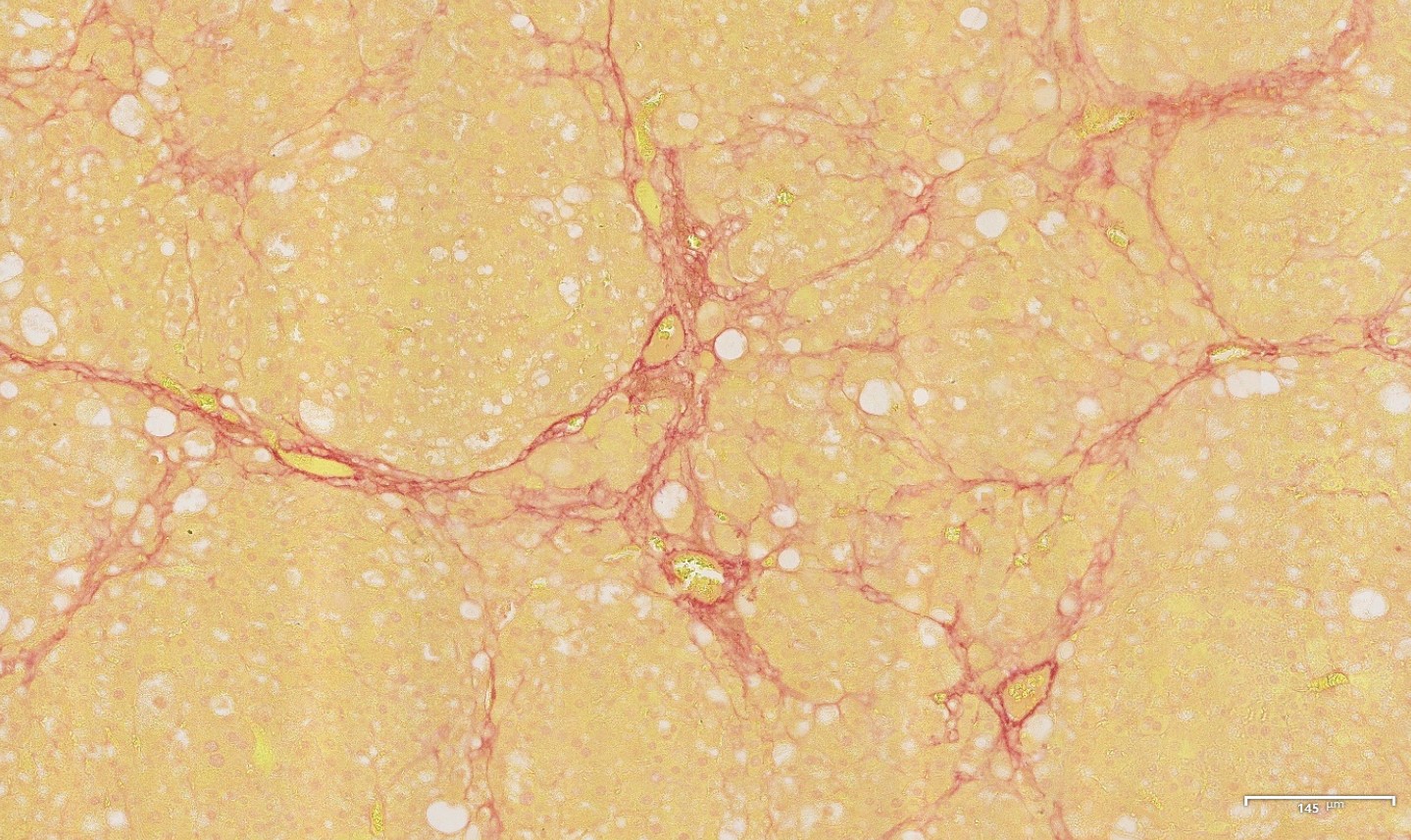

Supplement: Supplementary file 6 [file DataSheet5.ZIP › the original source data of Supplementary Figures /Fig. S4/Fig. S4A (Rat-CCl4)/F01.jpg]

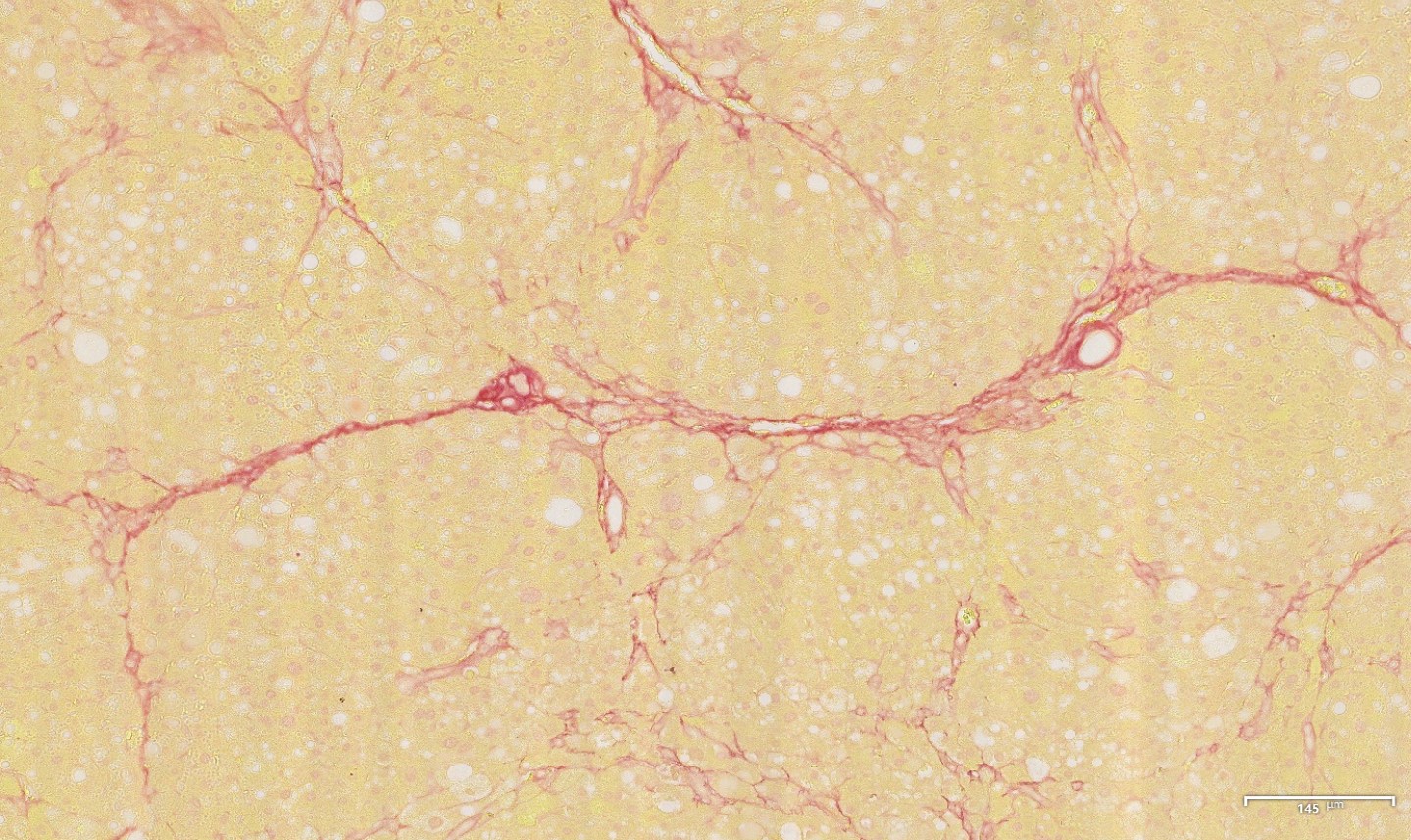

Supplement: Supplementary file 6 [file DataSheet5.ZIP › the original source data of Supplementary Figures /Fig. S4/Fig. S4A (Rat-CCl4)/F02.jpg]

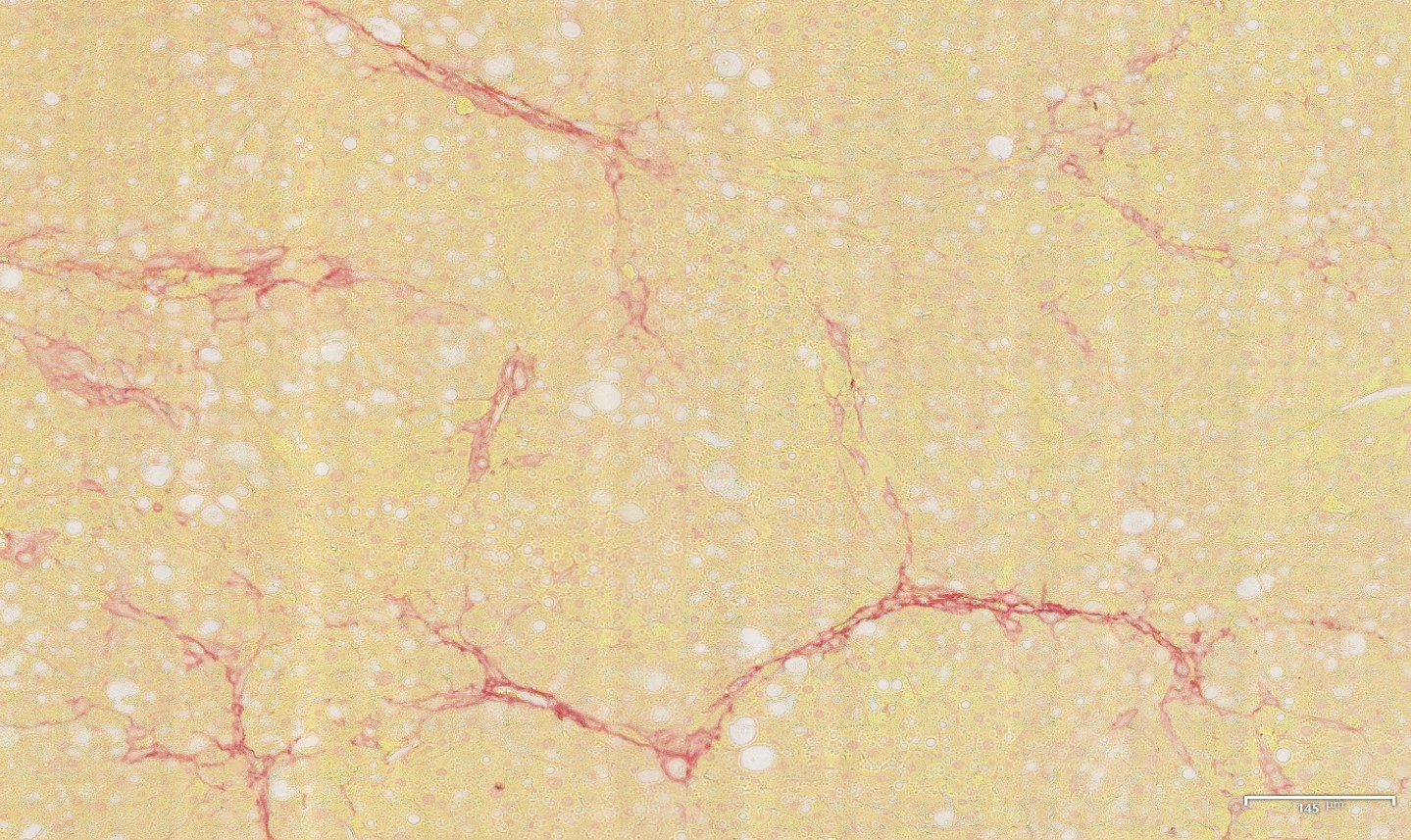

Supplement: Supplementary file 6 [file DataSheet5.ZIP › the original source data of Supplementary Figures /Fig. S4/Fig. S4A (Rat-CCl4)/F03.jpg]

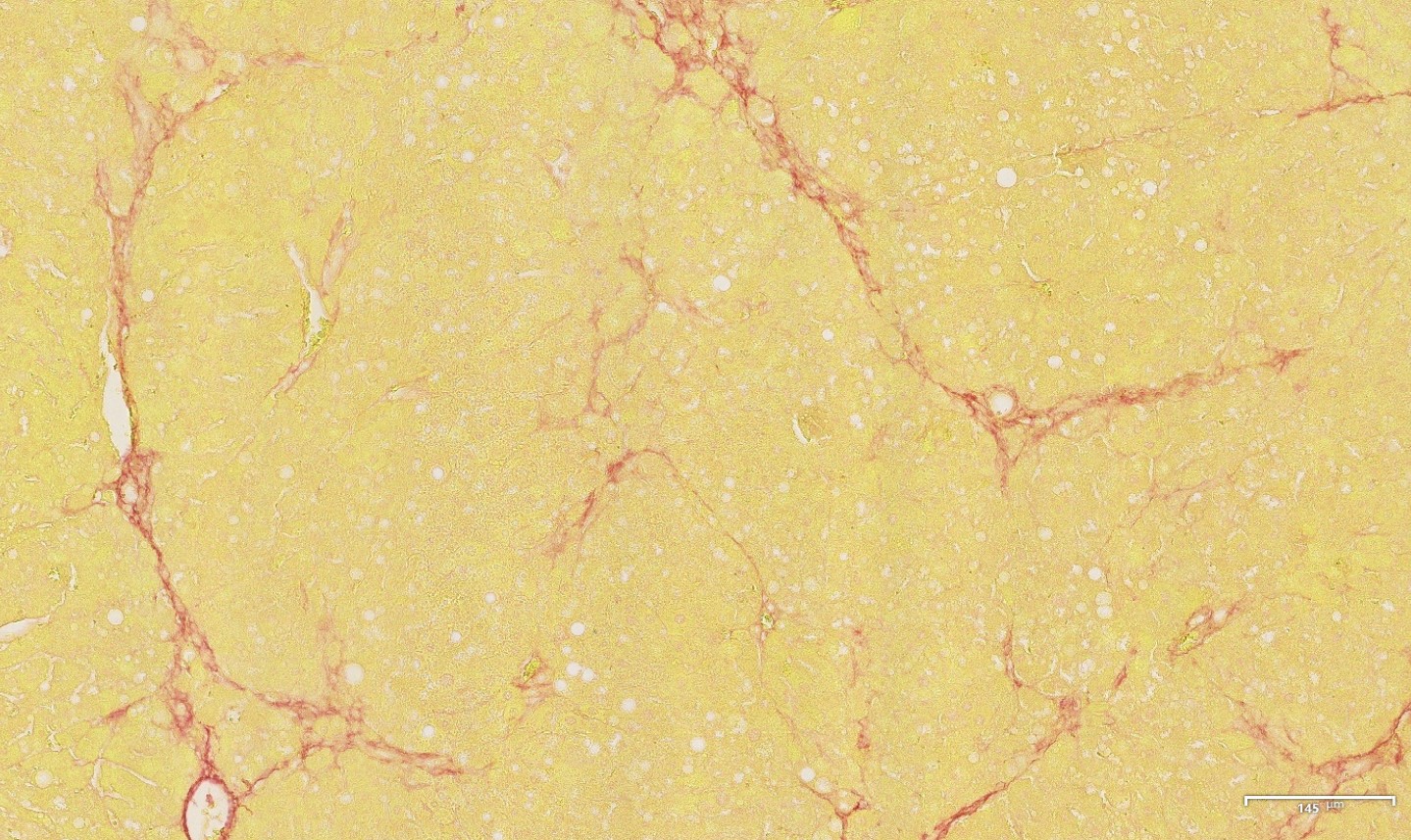

Supplement: Supplementary file 6 [file DataSheet5.ZIP › the original source data of Supplementary Figures /Fig. S4/Fig. S4A (Rat-CCl4)/FZHY.jpg]

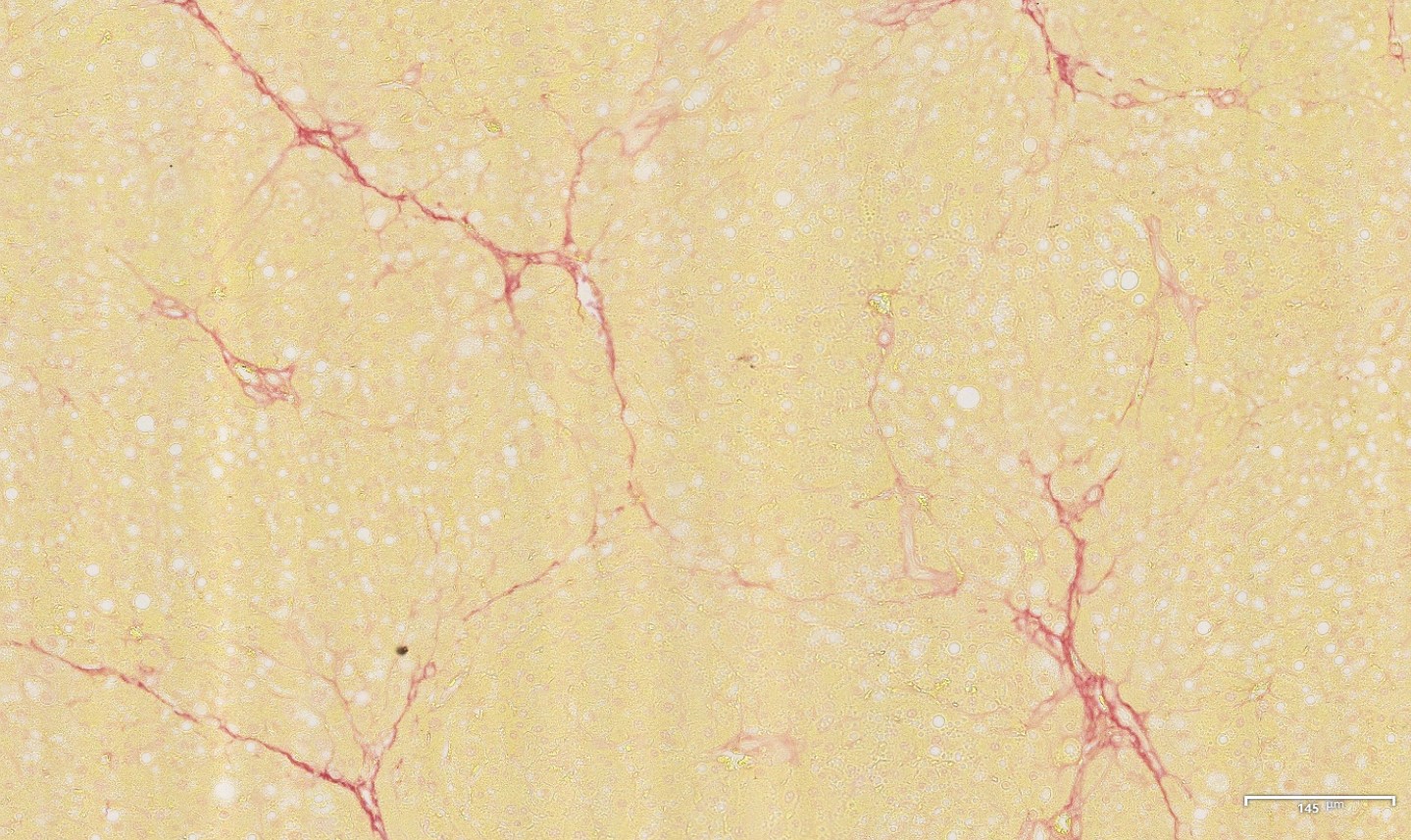

Supplement: Supplementary file 6 [file DataSheet5.ZIP › the original source data of Supplementary Figures /Fig. S4/Fig. S4A (Rat-CCl4)/JY5.jpg]

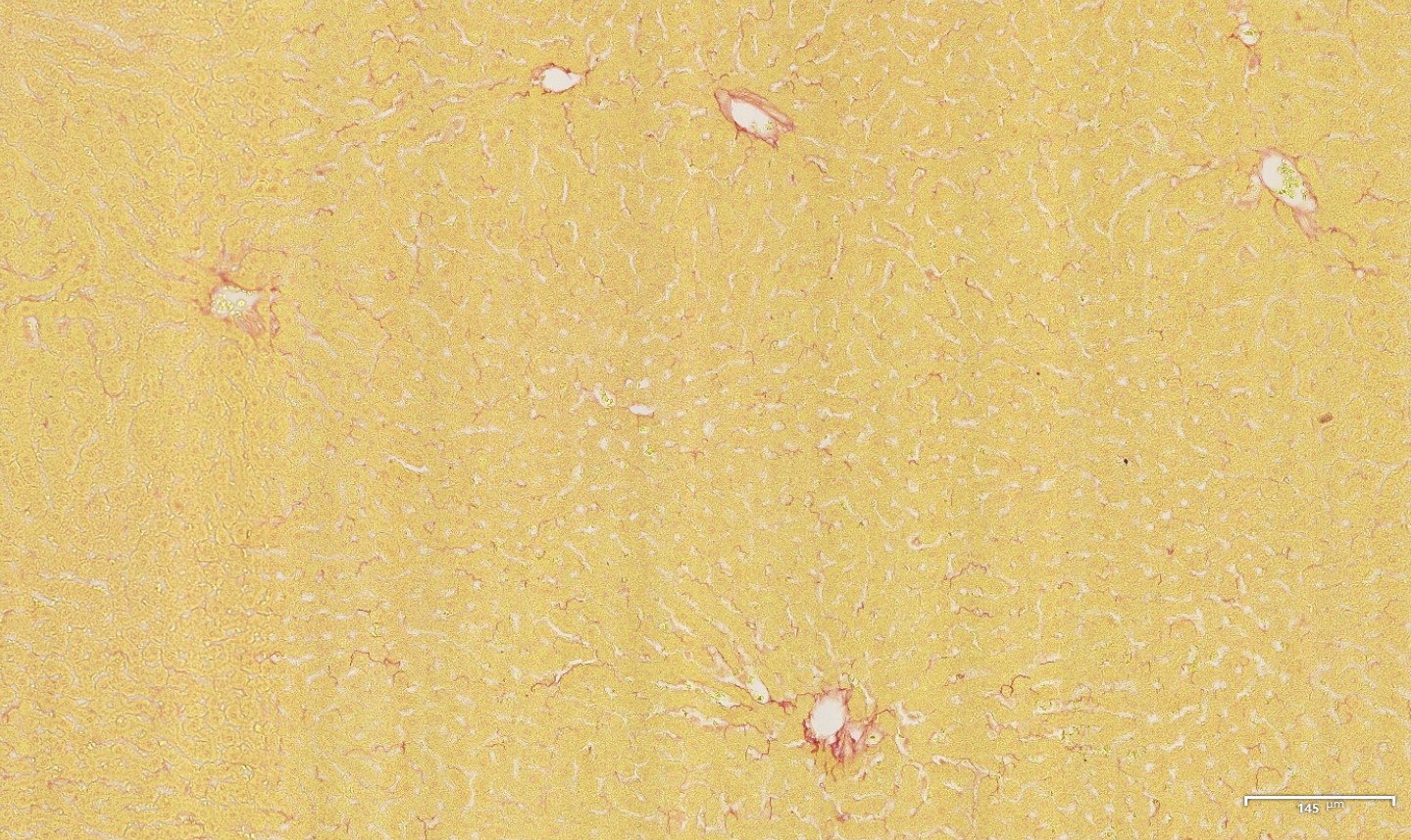

Supplement: Supplementary file 6 [file DataSheet5.ZIP › the original source data of Supplementary Figures /Fig. S4/Fig. S4A (Rat-CCl4)/Oil.jpg]

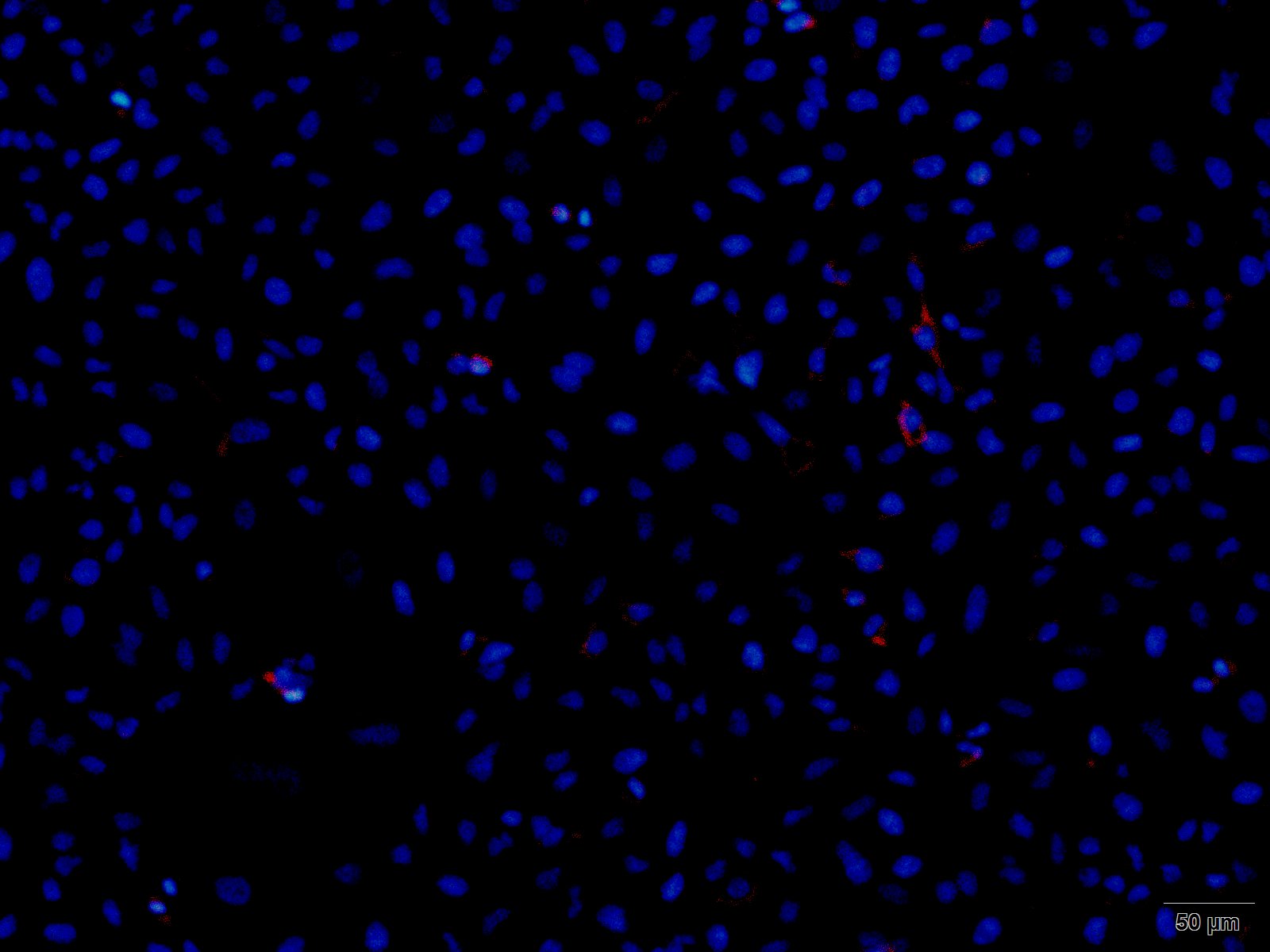

Supplement: Supplementary file 6 [file DataSheet5.ZIP › the original source data of Supplementary Figures /Review supporting file - 149956 comment 6/IF/a-SMA/Control.jpg]

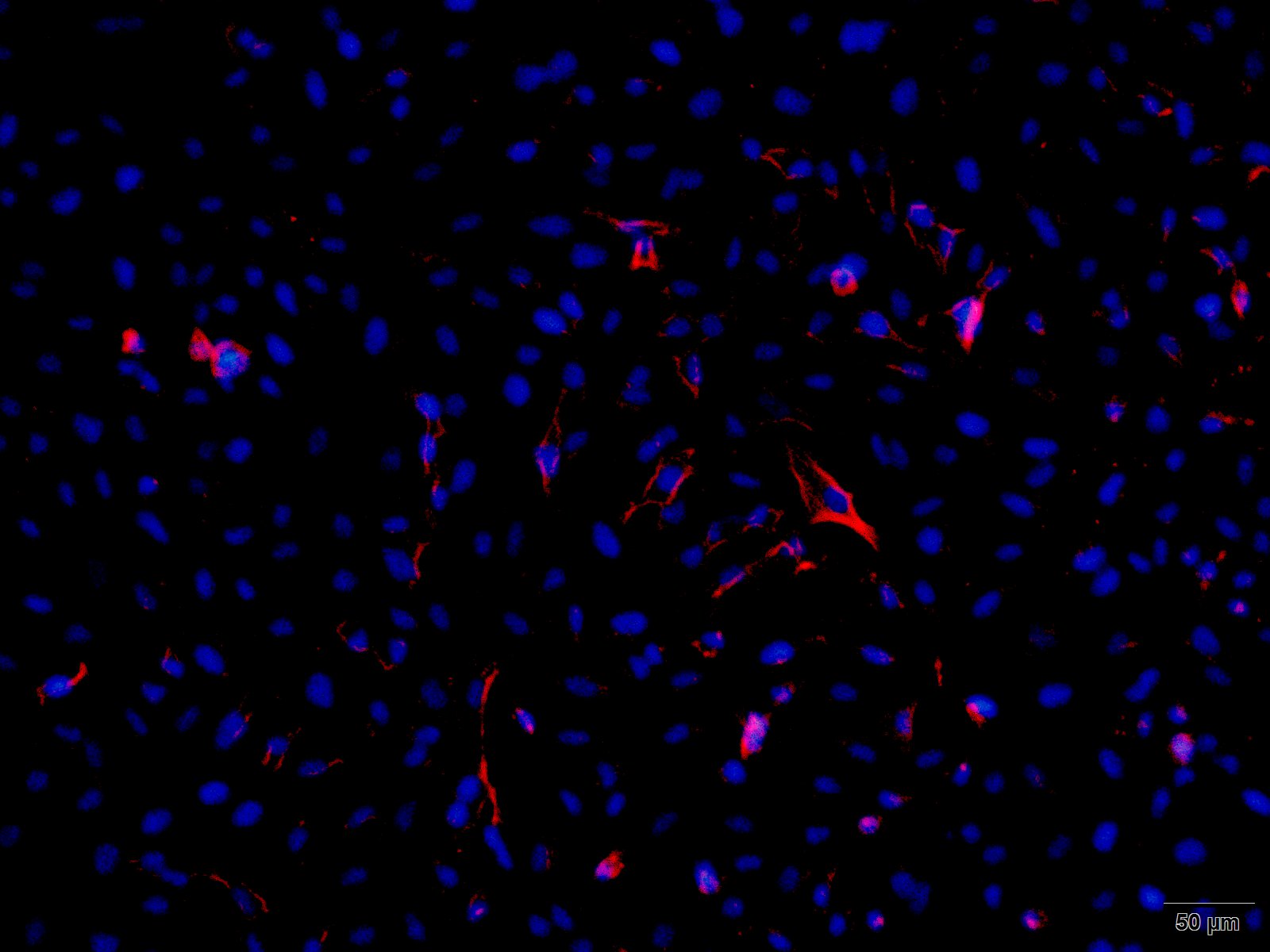

Supplement: Supplementary file 6 [file DataSheet5.ZIP › the original source data of Supplementary Figures /Review supporting file - 149956 comment 6/IF/a-SMA/DAPT(50uM).jpg]

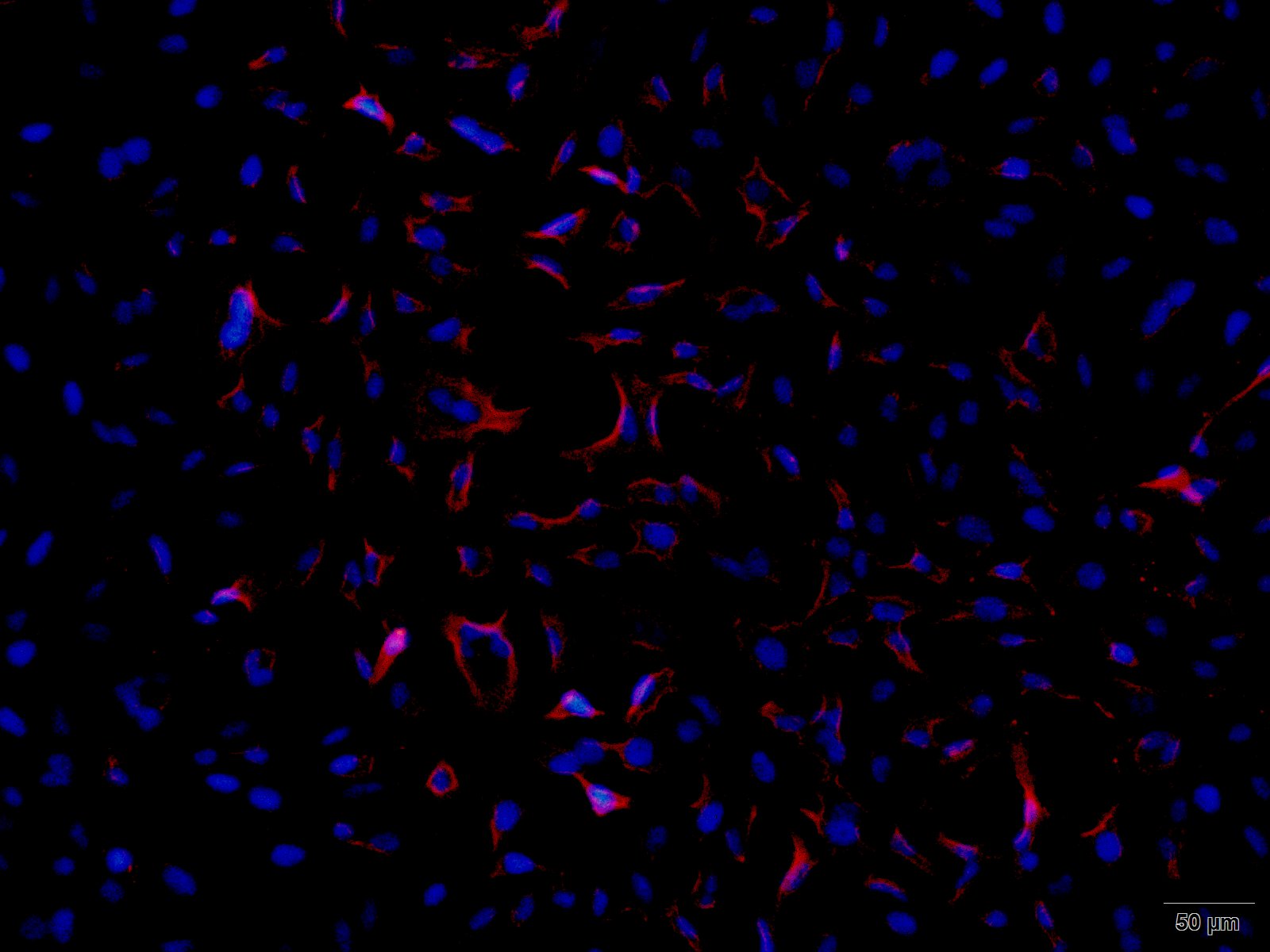

Supplement: Supplementary file 6 [file DataSheet5.ZIP › the original source data of Supplementary Figures /Review supporting file - 149956 comment 6/IF/a-SMA/JY5(37uM).jpg]

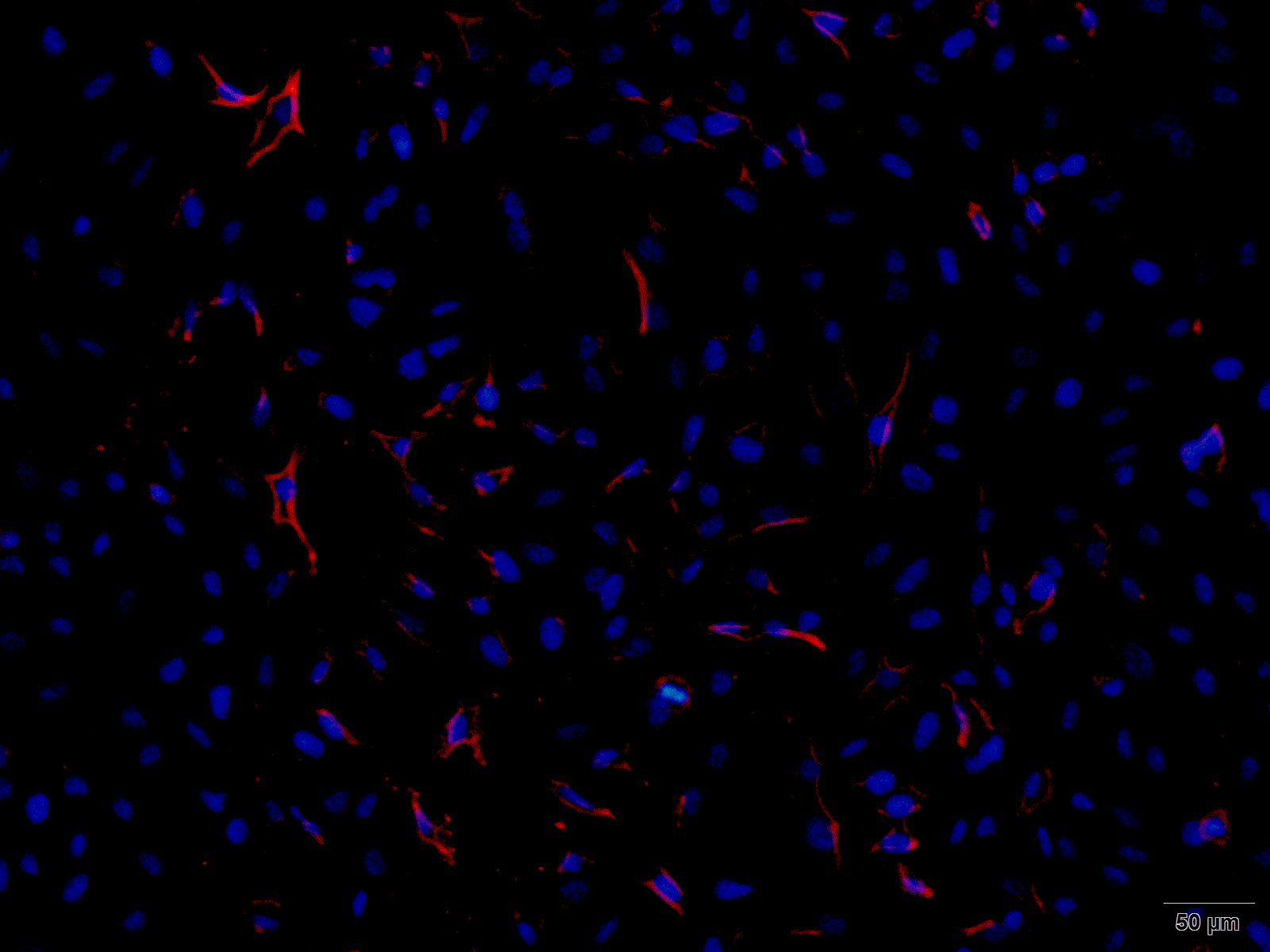

Supplement: Supplementary file 6 [file DataSheet5.ZIP › the original source data of Supplementary Figures /Review supporting file - 149956 comment 6/IF/a-SMA/JY5+DAPT.jpg]

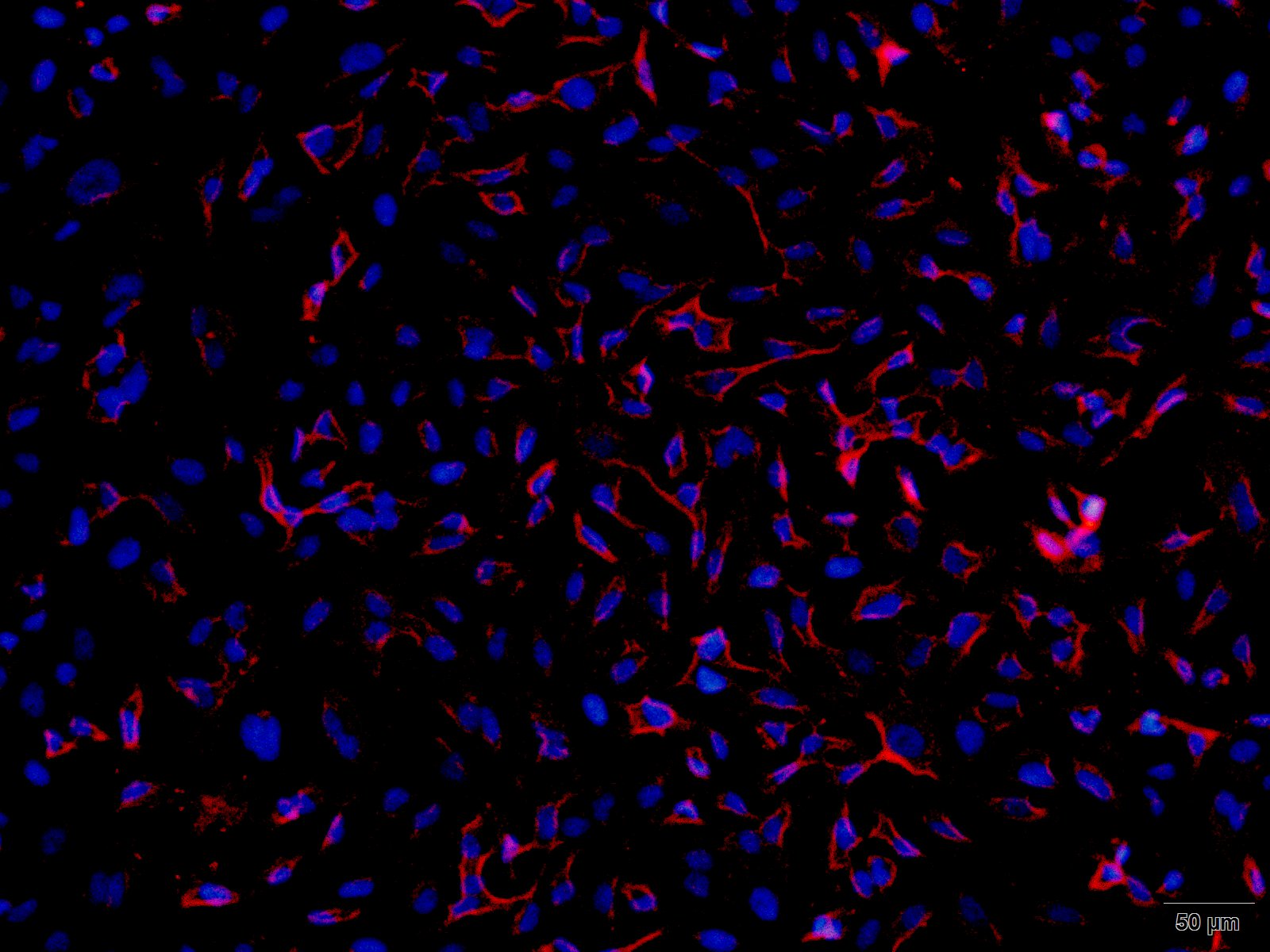

Supplement: Supplementary file 6 [file DataSheet5.ZIP › the original source data of Supplementary Figures /Review supporting file - 149956 comment 6/IF/a-SMA/TGF-beta1.jpg]

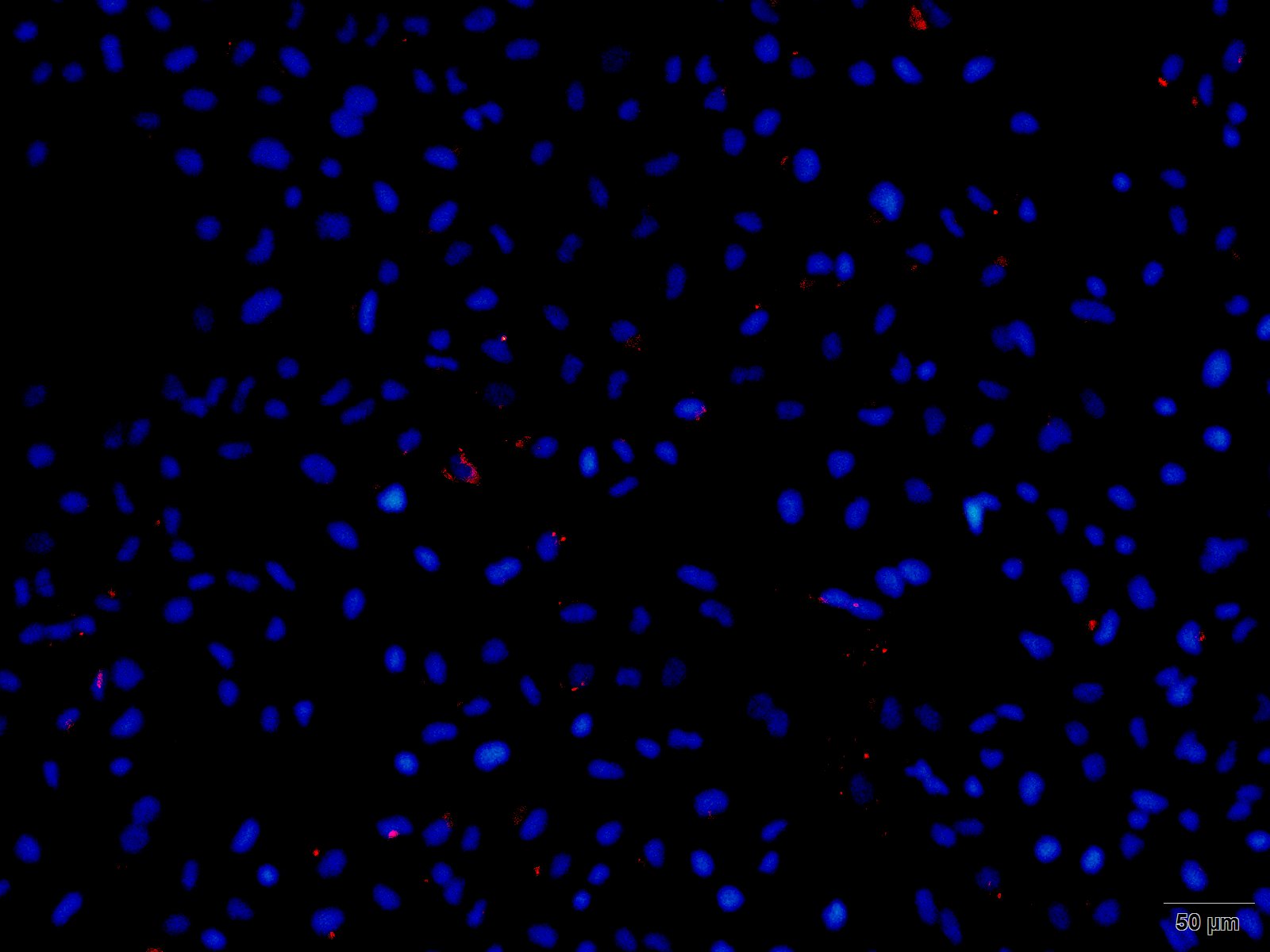

Supplement: Supplementary file 6 [file DataSheet5.ZIP › the original source data of Supplementary Figures /Review supporting file - 149956 comment 6/IF/Col-I/Control.jpg]

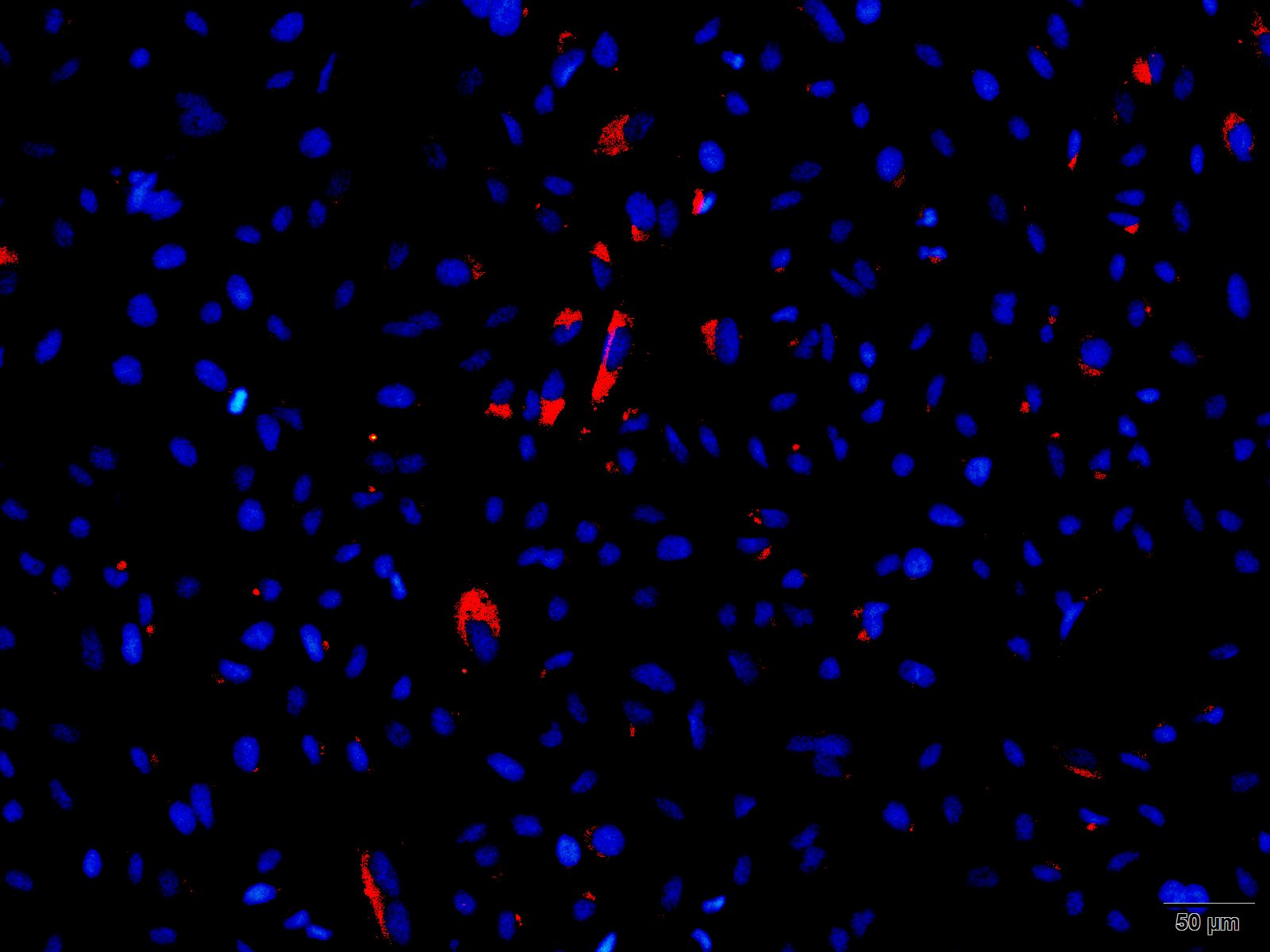

Supplement: Supplementary file 6 [file DataSheet5.ZIP › the original source data of Supplementary Figures /Review supporting file - 149956 comment 6/IF/Col-I/DAPT(50uM).jpg]

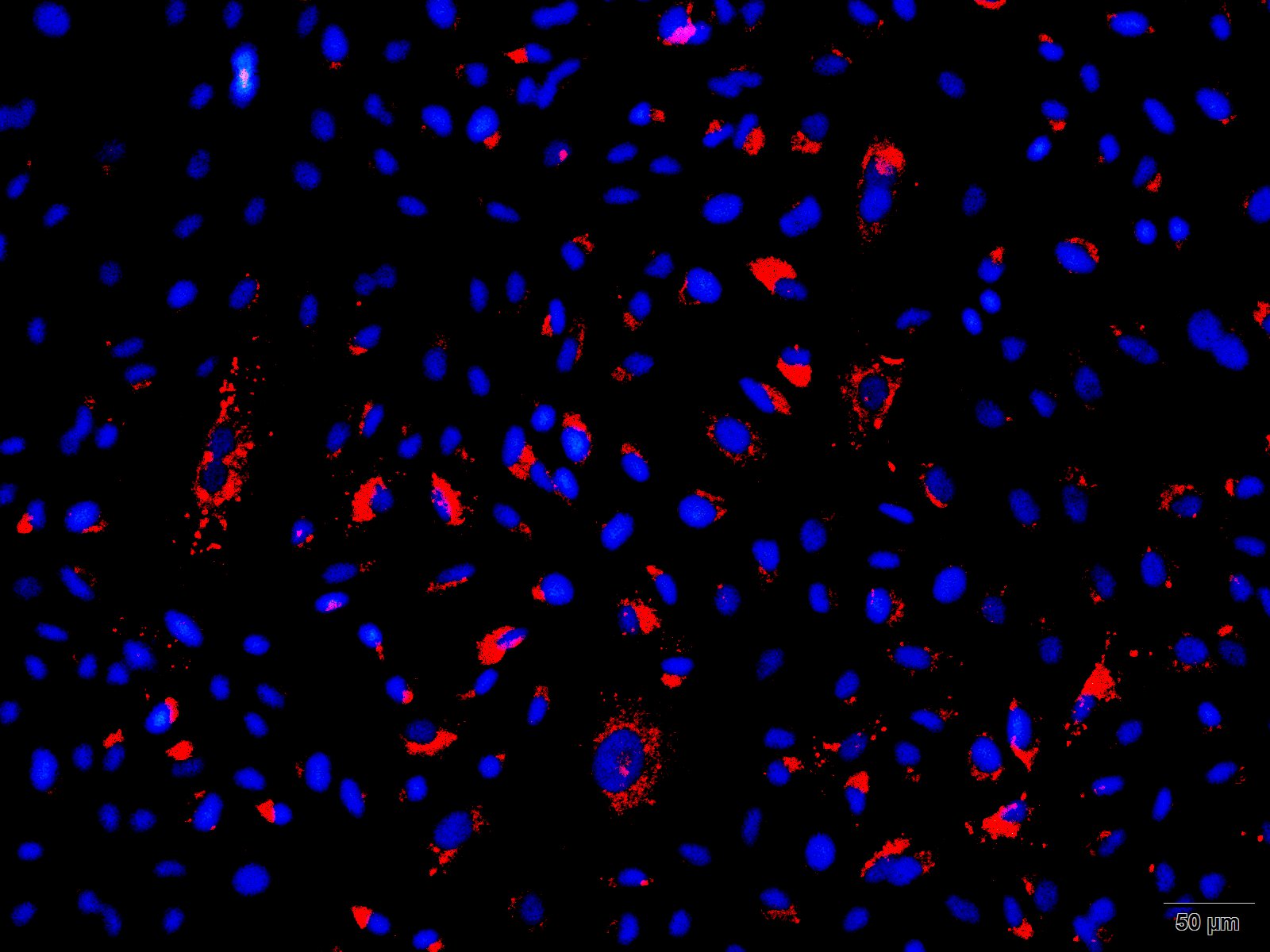

Supplement: Supplementary file 6 [file DataSheet5.ZIP › the original source data of Supplementary Figures /Review supporting file - 149956 comment 6/IF/Col-I/JY5(37uM).jpg]

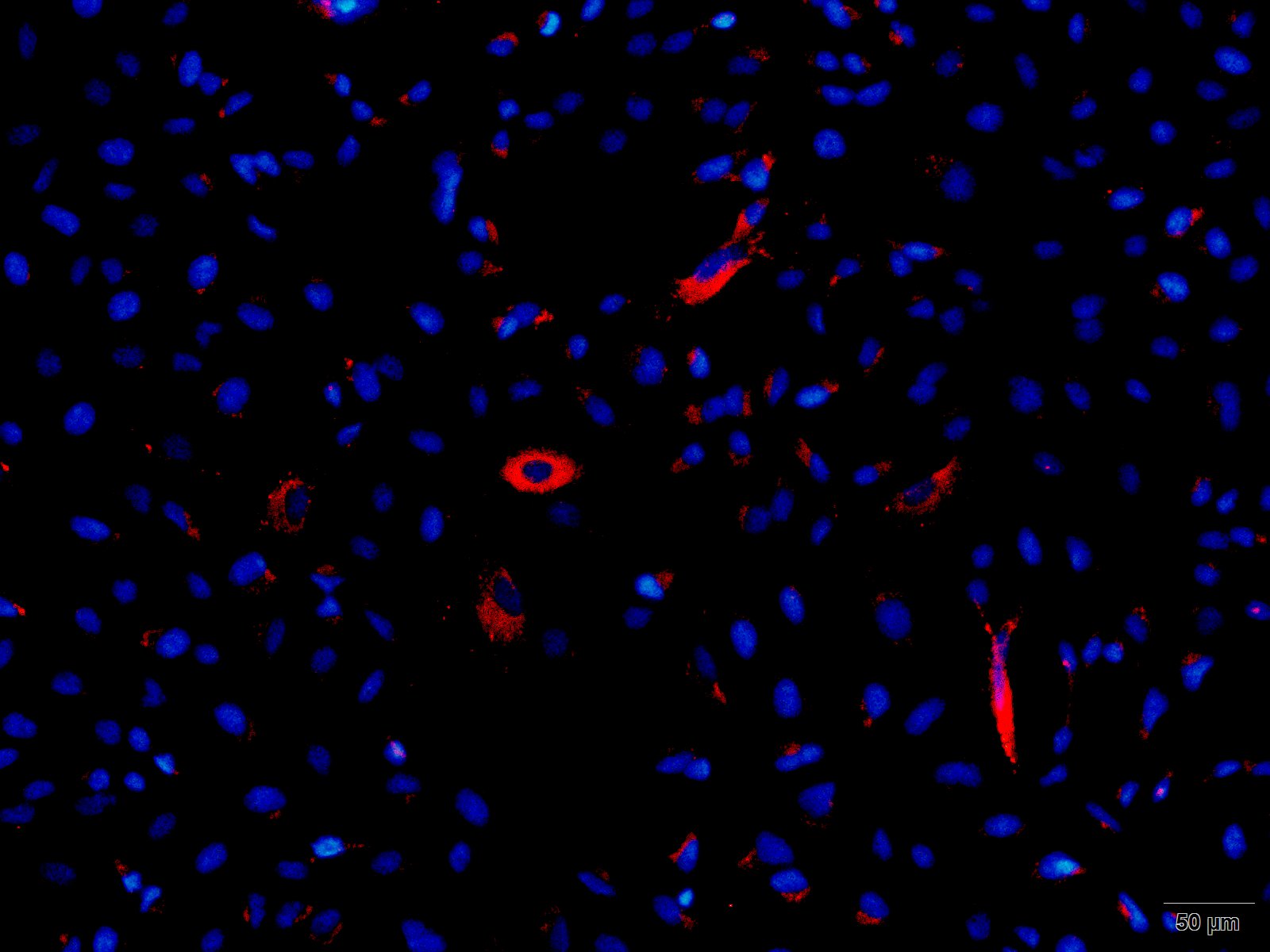

Supplement: Supplementary file 6 [file DataSheet5.ZIP › the original source data of Supplementary Figures /Review supporting file - 149956 comment 6/IF/Col-I/JY5+DAPT.jpg]

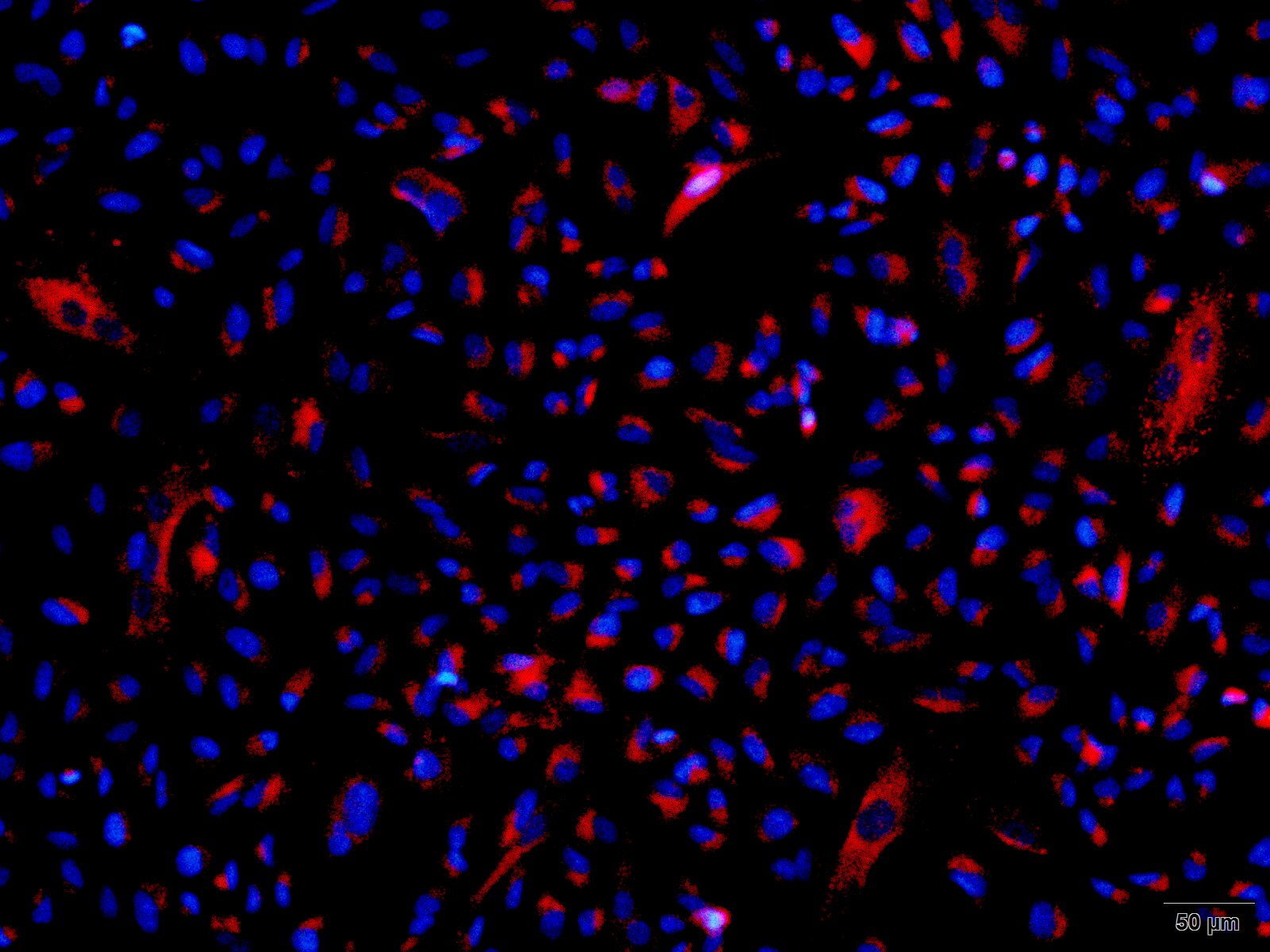

Supplement: Supplementary file 6 [file DataSheet5.ZIP › the original source data of Supplementary Figures /Review supporting file - 149956 comment 6/IF/Col-I/TGF-beta1.jpg]

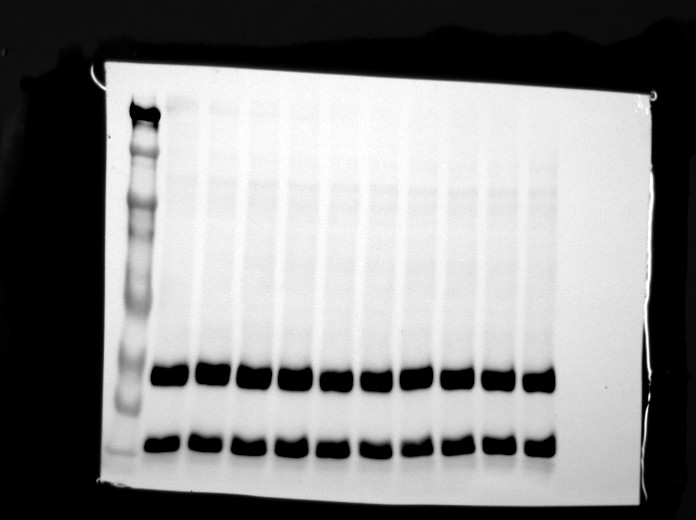

Supplement: Supplementary file 6 [file DataSheet5.ZIP › the original source data of Supplementary Figures /Review supporting file - 149956 comment 6/The original image file for the blots/LX-2-a-SMA-GAPDH.jpg]

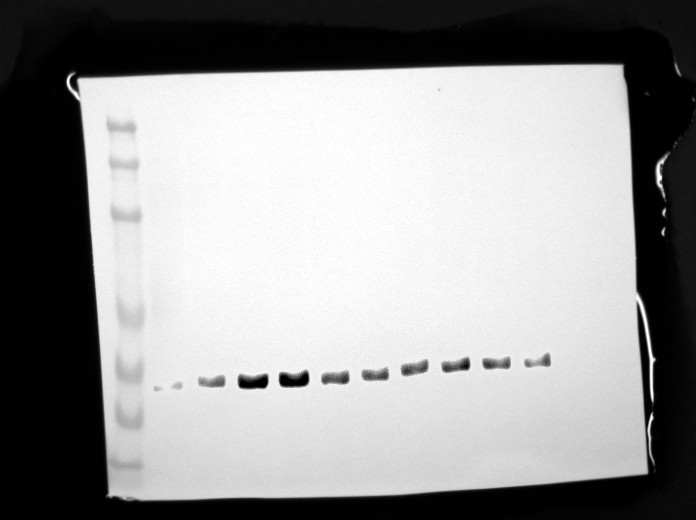

Supplement: Supplementary file 6 [file DataSheet5.ZIP › the original source data of Supplementary Figures /Review supporting file - 149956 comment 6/The original image file for the blots/LX-2-a-SMA.jpg]

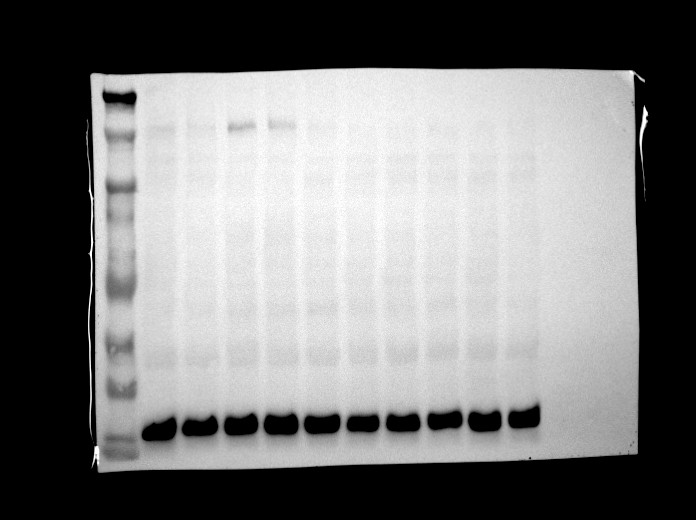

Supplement: Supplementary file 6 [file DataSheet5.ZIP › the original source data of Supplementary Figures /Review supporting file - 149956 comment 6/The original image file for the blots/LX-2-Jagged1-GAPDH.jpg]

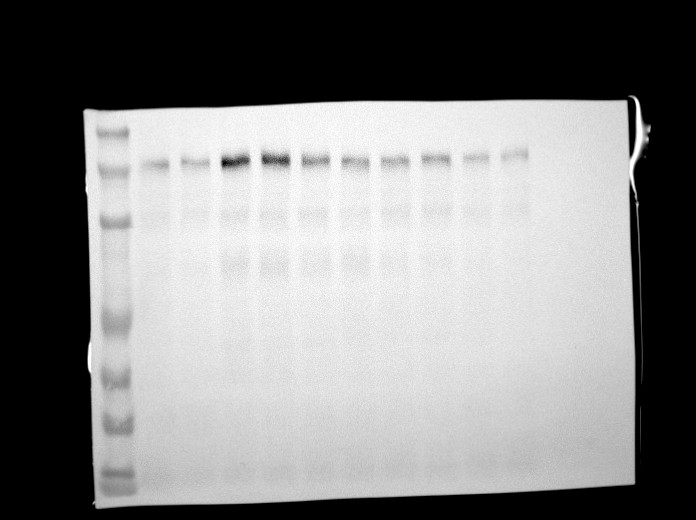

Supplement: Supplementary file 6 [file DataSheet5.ZIP › the original source data of Supplementary Figures /Review supporting file - 149956 comment 6/The original image file for the blots/LX-2-Jagged1.jpg]

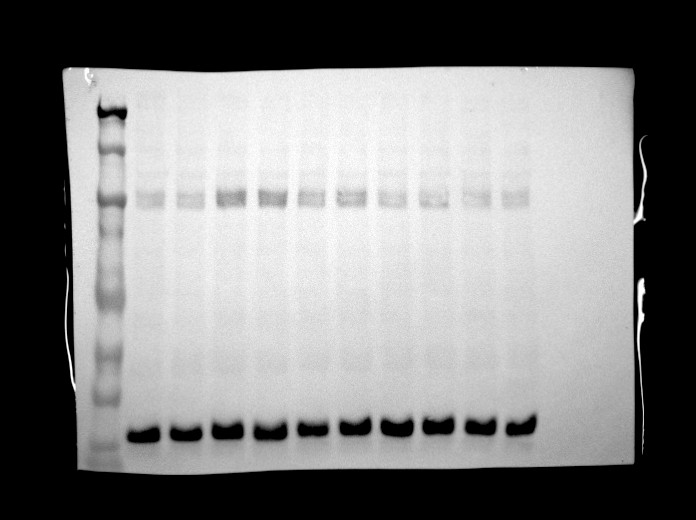

Supplement: Supplementary file 6 [file DataSheet5.ZIP › the original source data of Supplementary Figures /Review supporting file - 149956 comment 6/The original image file for the blots/LX-2-Notch2-GAPDH.jpg]

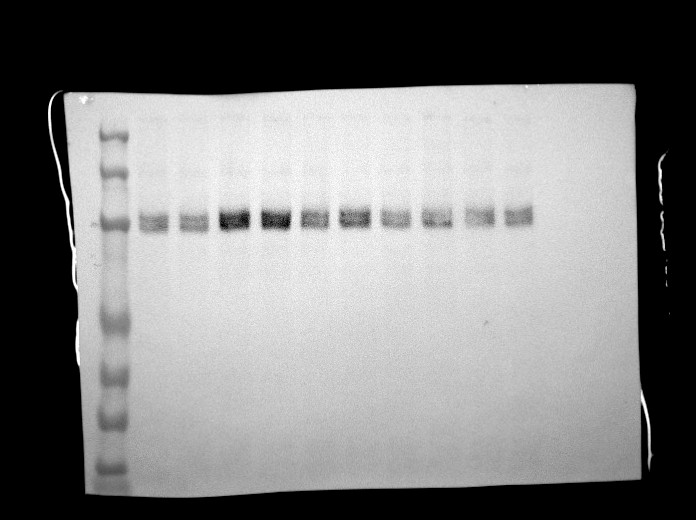

Supplement: Supplementary file 6 [file DataSheet5.ZIP › the original source data of Supplementary Figures /Review supporting file - 149956 comment 6/The original image file for the blots/LX-2-Notch2.jpg]

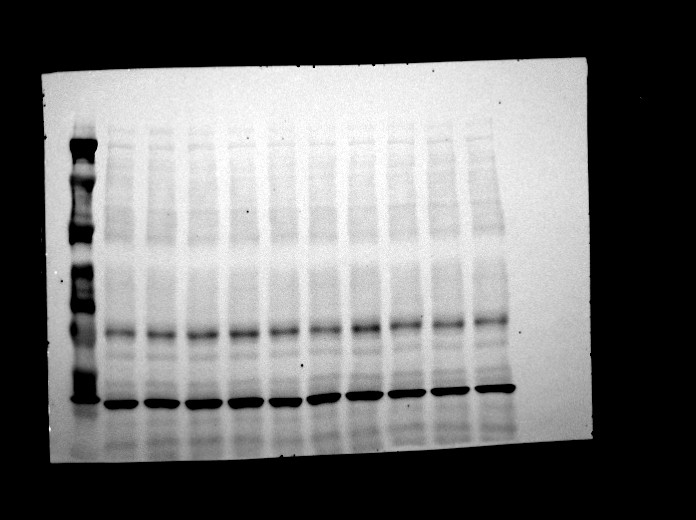

Supplement: Supplementary file 6 [file DataSheet5.ZIP › the original source data of Supplementary Figures /Review supporting file - 149956 comment 6/The original image file for the blots/LX-2-RBP-kB-GAPDH.jpg]

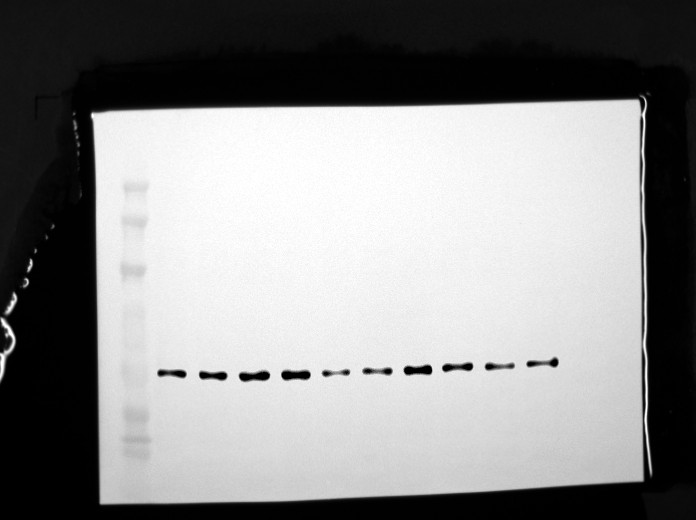

Supplement: Supplementary file 6 [file DataSheet5.ZIP › the original source data of Supplementary Figures /Review supporting file - 149956 comment 6/The original image file for the blots/LX-2-RBP-kB.jpg]
