# Supplementary material for: The Novel Chinese Medicine JY5 Formula Alleviates Hepatic Fibrosis by Inhibiting the Notch Signaling Pathway
Source: Front Pharmacol. 2021 Sep 22;12:671152. doi: 10.3389/fphar.2021.671152 (PMC8493219; doi:10.3389/fphar.2021.671152)
Supplement: Supplementary file 4 [file DataSheet1.ZIP › Ethical file/2019-0031.pdf]

上海南方模式生物科技股份有限公司（上海南方模式生物研究中心）动物看护与使用委员会

（*SRCMO IACUC*）

项目名称（***project name***）：基于细胞间相互作用解析扶正化瘀方抗肝纤维化的效应基础

项目负责人（***PI name***）：刘平　　递交日期（***application date***）2019-08-14

IACUC 编号：***IACUC NO.*** 2019-0031

项目负责人签名（***Signature***）

作为实验主要负责人，本人承诺人道地对待实验动物，严格按国家的相关法律法规以及 3R 的原则进行研究和使用实验动物，我向上海南方模式生物科技股份有限公司(上海南方模式生物研究中心)的 IACUC 承诺尽可能减少实验动物的使用数量，在每项实验中会减少动物的疼痛与不安。我经过详细的研究和考虑认为这项实验除了使用本实验动物的方法以外没有其他方法可以替代。我承诺每年会对该实验进行审核，如果实验中有相关改动，我会及时上报 IACUC 会议进行审核。

As Principal Investigator, I am aware that I have the ultimate responsibility, on a day-to-day basis, for the proper care and treatment of the laboratory animals. I agree to adhere to state and local laws and3R principle governing the use of animals in teaching and research. I further assure the Shanghai Research Centre （company）for Model Organisms (SRCMO) IACUC that the minimal number of animals will be used for the project and that every possible step will be taken to minimize stress or pain to the animals. I have carefully considered and concluded that no reasonable alternatives to the use of animals could be applied to this project, and that this project is not an unnecessary duplication of any previously published work.I will submit appropriate annual review forms for this project, and obtain formal approval of the Committee prior to implementation of any changes in this protocol.

主要负责人签名 *PI signature:* 刘平　日期 *Date:* 2019.08.14
