# Supplementary material for: The Novel Chinese Medicine JY5 Formula Alleviates Hepatic Fibrosis by Inhibiting the Notch Signaling Pathway
Source: Front Pharmacol. 2021 Sep 22;12:671152. doi: 10.3389/fphar.2021.671152 (PMC8493219; doi:10.3389/fphar.2021.671152)
Supplement: Supplementary file 4 [file DataSheet1.ZIP › Ethical file/Chinese edition of 2018-07-SZYD-LP-01.pdf]

复旦大学药学院实验动物伦理委员会

研究项目伦理审批件

伦药批第（2018-07-SZYD-LP-01）号

项目名称	基于细胞间相互作用解析扶正化瘀方抗肝纤维化的效应基础				
项目类别	基础 √ 临床 ___ 药物 ___				
项目来源	国家自然科学基金重点项目				
申办单位	上海中医药大学			单位负责人	徐建光
研究部门	附属曙光医院	项目负责人	刘平	职 称	教授
伦理审查意见					
Δ 同意				√	
Δ 修改后同意					
Δ 不同意（项目终止或暂停）					

审批意见

本项目已通过实验动物伦理审查，同意实施。

主任委员（签名）

复旦大学药学院实验动物伦理委员会

（盖章）

2018 年 07 月 13 日
