# Supplementary material for: The Novel Chinese Medicine JY5 Formula Alleviates Hepatic Fibrosis by Inhibiting the Notch Signaling Pathway
Source: Front Pharmacol. 2021 Sep 22;12:671152. doi: 10.3389/fphar.2021.671152 (PMC8493219; doi:10.3389/fphar.2021.671152)
Supplement: Supplementary file 4 [file DataSheet1.ZIP › Ethical file/English edition of 2018-07-SZYD-LP-01.pdf]

The Ethical Approval of the Research Project of Experimental Animal Ethics Committee of School of Pharmacy, Fudan University

Approval No.2018-07-SZYD-LP-01

Project name	Investigation the effect mechanism of Fuzheng Huayu formula on liver fibrosis based on the interaction between cells				
Project category	Basic √ Clinical Drug				
Project source	the Key Program of the National Natural Science Foundation of China				
Insitute	Shanghai University of Traditional Chinese Medicine			Sponsor	Jianguang Xu
Research department	Shuguang Hospital	Project manager	Ping Liu	Positional titles	Professor

Ethics review opinion

Δ **agree** √

Δ agree after modification

Δ disagree（Project termination or suspension）

Approval comments

This project was reviewed and approved by the Experimental Animal Ethics Committee of School of Pharmacy, Fudan University.

Chairman: Nengneng Cheng

Experimental Animal Ethics Committee of School of Pharmacy

Fudan University

July 13, 2018
